# Supplementary material for: Choice of assembly software has a critical impact on virome characterisation
Source: Microbiome. 2019 Jan 28;7:12. doi: 10.1186/s40168-019-0626-5 (PMC6350398; doi:10.1186/s40168-019-0626-5)
Supplement: Supplementary file 1 — Simulated virome MetaQUAST output. (HTML 6369 kb) [file 40168_2019_626_MOESM1_ESM.html]

|  |
| --- |
| QUAST **Quality Assessment Tool for Genome Assemblies** by CAB |

Loading...

Aligned to
""

Combined reference
Estimated reference size:
 bp
|
 bp
|
 references
|
 fragments
|
 % G+C
|
 chromosomes
  
 reads
|
 mapped
|
 properly paired
  
 genes
|
 operons

Unfortunately, JavaScript in your
browser is disabled or is not supported.
We need JavaScript to build report and plots.

Worst
Median
Best

Show heatmap

|  |  |
| --- | --- |
|  |  |
|  | |  |
| Contigs are ordered from largest (contig #1) to smallest.  Contigs are broken into nonoverlapping 100 bp windows. Plot shows numbers of windows for each GC percentage. |

[{"assembliesWithNs":["ABySS\_127","ABySS\_63","Ray\_Meta","SOAPdenovo2","SPAdes","SPAdes\_meta","SPAdes\_sc","SPAdes\_sc\_careful","Velvet","Geneious"],"minContig":500,"report":[["Genome statistics",[{"values":["49.166","45.196","68.601","66.146","69.665","65.873","25.520","38.068","72.273","68.138","68.854","68.645","47.738","0.004","41.262"],"quality":"More is better","isMain":true,"metricName":"Genome fraction (%)"},{"values":["1.025","1.010","1.008","1.006","1.010","2.694","1.029","1.034","1.011","1.005","1.005","1.006","1.002","1.082","1.048"],"quality":"Less is better","isMain":true,"metricName":"Duplication ratio"},{"values":[228547,212531,245567,244933,257479,257331,134892,134904,260428,260153,257672,257672,206050,562,434153],"quality":"More is better","isMain":true,"metricName":"Largest alignment"},{"values":[23674881,21425692,32442333,31244550,33018109,83297496,12328551,17984472,34285840,32128868,32454082,32393887,22451711,2023,20277960],"quality":"More is better","isMain":true,"metricName":"Total aligned length"},{"values":[9253,7426,16476,15678,14625,4305,13868,1876,29967,29468,14329,14453,11310,491,37621],"quality":"More is better","isMain":false,"metricName":"NA50"},{"values":[2263,2002,2863,2596,2447,2202,1996,679,3734,4000,2501,2392,3073,457,15111],"quality":"More is better","isMain":false,"metricName":"NA75"},{"values":[417,451,357,336,358,3580,169,1861,260,253,352,346,412,3,143],"quality":"Less is better","isMain":false,"metricName":"LA50"},{"values":[1793,1938,1688,1812,2007,10604,911,6849,1268,1105,1957,1988,1429,4,346],"quality":"Less is better","isMain":false,"metricName":"LA75"}]],["Misassemblies",[{"values":[47,24,117,106,69,361,21,33,165,95,133,129,21,0,471],"quality":"Less is better","isMain":true,"metricName":"# misassemblies"},{"values":[17,6,26,21,14,104,14,6,49,20,29,30,8,0,87],"quality":"Less is better","isMain":false,"metricName":" # relocations"},{"values":[0,0,0,0,0,0,0,0,0,0,0,0,0,0,0],"quality":"Less is better","isMain":false,"metricName":" # translocations"},{"values":[1,0,3,1,2,6,0,1,3,1,3,3,1,0,4],"quality":"Less is better","isMain":false,"metricName":" # inversions"},{"values":[29,18,88,84,53,251,7,26,113,74,101,96,12,0,380],"quality":"Less is better","isMain":false,"metricName":" # interspecies translocations"},{"values":[37,22,113,106,67,273,19,31,151,73,128,124,20,0,243],"quality":"Less is better","isMain":false,"metricName":"# misassembled contigs"},{"values":[1185158,483198,442144,439028,368452,5922378,838732,91438,1666997,1236945,1450687,1436341,55036,0,9289166],"quality":"Less is better","isMain":true,"metricName":"Misassembled contigs length"},{"values":[2,0,11,13,7,9,2,201,22,14,14,16,0,0,39],"quality":"Less is better","isMain":false,"metricName":"# possibly misassembled contigs"},{"values":[4,0,12,13,7,15,4,408,24,23,15,16,0,0,69],"quality":"Less is better","isMain":false,"metricName":" # possible misassemblies"},{"values":[37,13,34,123,166,280,67,208,231,98,135,128,34,0,212],"quality":"Less is better","isMain":false,"metricName":"# local misassemblies"},{"values":[0,0,3,1,0,0,0,542,0,0,0,0,0,0,0],"quality":"Less is better","isMain":false,"metricName":"# unaligned mis. contigs"}]],["Unaligned",[{"values":[0,1,34,25,21,4,0,536,29,5,38,40,1,0,9],"quality":"Less is better","isMain":false,"metricName":"# fully unaligned contigs"},{"values":[0,825,32945,21695,18765,5250,0,590096,34376,4567,43665,45195,806,0,23824],"quality":"Less is better","isMain":false,"metricName":"Fully unaligned length"},{"values":[2,1,19,22,12,11,3,925,42,28,49,52,1,0,42],"quality":"Less is better","isMain":false,"metricName":"# partially unaligned contigs"},{"values":[1304,1537,18315,17620,11111,10076,40039,1881407,48334,34530,44069,48589,736,0,73178],"quality":"Less is better","isMain":false,"metricName":"Partially unaligned length"}]],["Mismatches",[{"values":[6759,5747,25043,19644,24709,37700,3440,6572,33197,36945,30302,24586,5382,19,33734],"quality":"Less is better","isMain":false,"metricName":"# mismatches"},{"values":[303,185,968,846,928,1806,242,5257,1329,1487,1291,1127,208,6,2731],"quality":"Less is better","isMain":false,"metricName":"# indels"},{"values":[2295,1159,3206,4734,4518,6687,3291,250676,6173,12931,14888,13986,2642,6,9240],"quality":"Less is better","isMain":false,"metricName":"Indels length"},{"values":["29.27","27.07","77.72","63.23","75.52","121.85","28.70","36.76","97.80","115.45","93.70","76.26","24.00","939.20","174.07"],"quality":"Less is better","isMain":true,"metricName":"# mismatches per 100 kbp"},{"values":["1.31","0.87","3.00","2.72","2.84","5.84","2.02","29.40","3.92","4.65","3.99","3.50","0.93","296.59","14.09"],"quality":"Less is better","isMain":true,"metricName":"# indels per 100 kbp"},{"values":[225,144,876,680,785,1587,154,45,1115,1145,909,776,125,6,2447],"quality":"Less is better","isMain":false,"metricName":" # indels (<= 5 bp)"},{"values":[78,41,92,166,143,219,88,5212,214,342,382,351,83,0,284],"quality":"Less is better","isMain":false,"metricName":" # indels (> 5 bp)"},{"values":[2087,10174,70,0,0,31371,38470,1202313,16835,19343,37748,37921,561,0,58221],"quality":"Less is better","isMain":false,"metricName":"# N's"},{"values":["8.81","47.47","0.22","0.00","0.00","37.63","310.92","5734.93","48.93","60.09","115.85","116.57","2.50","0.00","285.24"],"quality":"Less is better","isMain":true,"metricName":"# N's per 100 kbp"}]],["Statistics without reference",[{"values":[7957,7732,9152,8999,10083,27600,4224,11548,8230,7419,9506,9724,6343,4,958],"quality":"Equal","isMain":true,"metricName":"# contigs"},{"values":[370491,365479,13713,28327,133775,28198,33995,51732,14948,12769,18136,24689,37706,58,958],"quality":"Equal","isMain":false,"metricName":"# contigs (>= 0 bp)"},{"values":[3819,3722,4786,4927,5079,19809,1984,5026,4475,4126,5189,5164,3617,0,954],"quality":"Equal","isMain":false,"metricName":"# contigs (>= 1000 bp)"},{"values":[795,723,1003,902,942,2706,351,686,973,900,939,918,895,0,566],"quality":"Equal","isMain":false,"metricName":"# contigs (>= 5000 bp)"},{"values":[373,313,545,467,485,870,194,236,506,520,472,461,466,0,360],"quality":"Equal","isMain":false,"metricName":"# contigs (>= 10000 bp)"},{"values":[149,129,270,244,250,311,111,64,279,291,231,234,160,0,229],"quality":"Equal","isMain":false,"metricName":"# contigs (>= 25000 bp)"},{"values":[46,35,86,81,88,107,22,8,111,115,84,86,34,0,107],"quality":"Equal","isMain":false,"metricName":"# contigs (>= 50000 bp)"},{"values":[254220,212531,245567,244933,257479,257331,168755,143833,260428,260153,257672,257672,206050,575,493731],"quality":"More is better","isMain":true,"metricName":"Largest contig"},{"values":[23681087,21432785,32520957,31301066,33063967,83362307,12372995,20964737,34403483,32191509,32583190,32531234,22456974,2189,20411466],"quality":"More is better","isMain":true,"metricName":"Total length"},{"values":[93104463,59887345,34080536,35290817,65789049,83623020,17261783,29532819,36698861,34069084,35239336,36159364,27134710,15629,20411466],"quality":"More is better","isMain":false,"metricName":"Total length (>= 0 bp)"},{"values":[20800608,18620360,29438093,28401161,29537783,77880567,10823994,16446893,31728463,29803343,29481300,29259287,20558915,0,20407831],"quality":"More is better","isMain":true,"metricName":"Total length (>= 1000 bp)"},{"values":[14587695,12424905,21868488,20293423,21202095,38464194,7670699,8166787,24731787,23426530,21004194,20839174,14756571,0,19256390],"quality":"More is better","isMain":false,"metricName":"Total length (>= 5000 bp)"},{"values":[11616775,9592773,18720616,17300896,18060662,26066031,6604341,5081779,21481760,20792976,17792033,17713386,11805214,0,17812757],"quality":"More is better","isMain":true,"metricName":"Total length (>= 10000 bp)"},{"values":[8108365,6795598,14580506,13894703,14553640,18199348,5281051,2486355,18006271,17361350,14192549,14305769,7061500,0,15726390],"quality":"More is better","isMain":false,"metricName":"Total length (>= 25000 bp)"},{"values":[4335830,3342681,7723720,7755862,8368074,10612165,1923336,594195,11519040,10848548,8526821,8602205,2580830,0,11104104],"quality":"More is better","isMain":true,"metricName":"Total length (>= 50000 bp)"},{"values":[9466,7528,16694,15712,14657,4365,14676,3018,31536,31099,14816,14847,11310,553,56961],"quality":"More is better","isMain":false,"metricName":"N50"},{"values":[2272,2012,2912,2634,2463,2215,2050,1125,3906,4156,2570,2460,3081,548,28523],"quality":"More is better","isMain":false,"metricName":"N75"},{"values":[397,444,354,334,356,3396,160,1291,251,246,345,340,412,2,91],"quality":"Less is better","isMain":false,"metricName":"L50"},{"values":[1752,1917,1664,1794,1985,10356,873,4343,1215,1057,1902,1935,1426,3,214],"quality":"Less is better","isMain":false,"metricName":"L75"}]],["Predicted genes",[]],["Similarity statistics",[{"values":[34,33,51,54,57,21,11,4,55,50,51,50,36,0,14],"quality":"Equal","isMain":false,"metricName":"# similar correct contigs"},{"values":[0,0,0,0,0,0,0,0,0,0,0,0,0,0,0],"quality":"Equal","isMain":false,"metricName":"# similar misassembled blocks"}]],["Reference statistics",[{"values":[46967092,46967092,46967092,46967092,46967092,46967092,46967092,46967092,46967092,46967092,46967092,46967092,46967092,46967092,46967092],"quality":"Equal","isMain":false,"metricName":"Reference length"},{"values":[572,572,572,572,572,572,572,572,572,572,572,572,572,572,572],"quality":"Equal","isMain":false,"metricName":"Reference fragments"}]]],"referenceName":"combined\_reference","date":"18 July 2018, Wednesday, 16:04:27","order":[0,1,2,3,4,5,6,7,8,9,10,11,12,13,14],"assembliesNames":["ABySS\_127","ABySS\_63","CLC","IDBA\_UD","MEGAHIT","MIRA","Ray\_Meta","SOAPdenovo2","SPAdes","SPAdes\_meta","SPAdes\_sc","SPAdes\_sc\_careful","Velvet","VICUNA","Geneious"]},{"assembliesWithNs":null,"minContig":500,"report":[["Genome statistics",[{"values":["98.731","97.587","98.149","98.343","98.558","99.118","80.547","66.126","98.892","98.500","98.558","98.549","96.347","99.194"],"quality":"More is better","isMain":true,"metricName":"Genome fraction (%)"},{"values":["1.000","1.000","1.000","1.000","1.001","1.000","1.023","1.062","1.000","1.000","1.001","1.001","1.000","1.000"],"quality":"Less is better","isMain":true,"metricName":"Duplication ratio"},{"values":[116413,124904,82194,116359,116438,129456,4525,7835,129161,125482,125772,125772,70038,129555],"quality":"More is better","isMain":true,"metricName":"Largest alignment"},{"values":[128978,127500,128163,128444,128849,129456,107580,87507,129161,128655,128761,128765,125867,129555],"quality":"More is better","isMain":true,"metricName":"Total aligned length"},{"values":[116413,124904,82194,116359,116438,129456,1135,4490,129161,127023,127297,127297,70038,129608],"quality":"More is better","isMain":false,"metricName":"NG50"},{"values":[116413,124904,34031,116359,116438,129456,672,897,129161,127023,127297,127297,45092,129608],"quality":"More is better","isMain":false,"metricName":"NG75"},{"values":[116413,124904,82194,116359,116438,129456,1266,3154,129161,125482,125772,125772,70038,129555],"quality":"More is better","isMain":false,"metricName":"NA50"},{"values":[116413,124904,34031,116359,116438,129456,955,897,129161,125482,125772,125772,45092,129555],"quality":"More is better","isMain":false,"metricName":"NA75"},{"values":[116413,124904,82194,116359,116438,129456,1135,1889,129161,125482,125772,125772,70038,129555],"quality":"More is better","isMain":true,"metricName":"NGA50"},{"values":[116413,124904,34031,116359,116438,129456,672,null,129161,125482,125772,125772,45092,129555],"quality":"More is better","isMain":false,"metricName":"NGA75"},{"values":[1,1,1,1,1,1,38,11,1,1,1,1,1,1],"quality":"Less is better","isMain":false,"metricName":"LG50"},{"values":[1,1,2,1,1,1,74,29,1,1,1,1,2,1],"quality":"Less is better","isMain":false,"metricName":"LG75"},{"values":[1,1,1,1,1,1,28,10,1,1,1,1,1,1],"quality":"Less is better","isMain":false,"metricName":"LA50"},{"values":[1,1,2,1,1,1,52,26,1,1,1,1,2,1],"quality":"Less is better","isMain":false,"metricName":"LA75"},{"values":[1,1,1,1,1,1,38,15,1,1,1,1,1,1],"quality":"Less is better","isMain":true,"metricName":"LGA50"},{"values":[1,1,2,1,1,1,74,null,1,1,1,1,2,1],"quality":"Less is better","isMain":false,"metricName":"LGA75"}]],["Misassemblies",[{"values":[0,0,0,0,0,0,0,0,0,1,1,1,0,0],"quality":"Less is better","isMain":true,"metricName":"# misassemblies"},{"values":[0,0,0,0,0,0,0,0,0,1,1,1,0,0],"quality":"Less is better","isMain":false,"metricName":" # relocations"},{"values":[0,0,0,0,0,0,0,0,0,0,0,0,0,0],"quality":"Less is better","isMain":false,"metricName":" # translocations"},{"values":[0,0,0,0,0,0,0,0,0,0,0,0,0,0],"quality":"Less is better","isMain":false,"metricName":" # inversions"},{"values":[0,0,0,0,0,0,0,0,0,1,1,1,0,0],"quality":"Less is better","isMain":false,"metricName":"# misassembled contigs"},{"values":[0,0,0,0,0,0,0,0,0,127023,127297,127297,0,0],"quality":"Less is better","isMain":true,"metricName":"Misassembled contigs length"},{"values":[0,0,1,0,0,0,0,2,0,1,1,1,0,0],"quality":"Less is better","isMain":false,"metricName":"# local misassemblies"},{"values":[0,0,0,0,0,0,0,0,0,0,0,0,0,0],"quality":"Less is better","isMain":false,"metricName":"# unaligned mis. contigs"}]],["Unaligned",[{"values":[0,0,0,0,0,0,0,0,0,0,0,0,0,0],"quality":"Less is better","isMain":false,"metricName":"# fully unaligned contigs"},{"values":[0,0,0,0,0,0,0,0,0,0,0,0,0,0],"quality":"Less is better","isMain":false,"metricName":"Fully unaligned length"},{"values":[0,0,0,0,0,0,0,6,0,0,0,0,0,0],"quality":"Less is better","isMain":false,"metricName":"# partially unaligned contigs"},{"values":[0,0,0,0,0,0,0,13499,0,0,0,0,0,0],"quality":"Less is better","isMain":false,"metricName":"Partially unaligned length"}]],["Mismatches",[{"values":[0,0,20,0,0,0,0,2,0,15,16,3,0,0],"quality":"Less is better","isMain":false,"metricName":"# mismatches"},{"values":[1,0,11,2,1,1,1,66,1,4,4,2,0,3],"quality":"Less is better","isMain":false,"metricName":"# indels"},{"values":[29,0,39,50,29,29,18,3117,29,41,27,50,0,50],"quality":"Less is better","isMain":false,"metricName":"Indels length"},{"values":["0.00","0.00","15.60","0.00","0.00","0.00","0.00","2.32","0.00","11.66","12.43","2.33","0.00","0.00"],"quality":"Less is better","isMain":true,"metricName":"# mismatches per 100 kbp"},{"values":["0.78","0.00","8.58","1.56","0.78","0.77","0.95","76.42","0.77","3.11","3.11","1.55","0.00","2.32"],"quality":"Less is better","isMain":true,"metricName":"# indels per 100 kbp"},{"values":[0,0,8,0,0,0,0,0,0,2,3,0,0,1],"quality":"Less is better","isMain":false,"metricName":" # indels (<= 5 bp)"},{"values":[1,0,3,2,1,1,1,66,1,2,1,2,0,2],"quality":"Less is better","isMain":false,"metricName":" # indels (> 5 bp)"},{"values":[0,0,0,0,0,0,0,9025,0,0,10,0,0,2],"quality":"Less is better","isMain":false,"metricName":"# N's"},{"values":["0.00","0.00","0.00","0.00","0.00","0.00","0.00","8574.82","0.00","0.00","7.76","0.00","0.00","1.54"],"quality":"Less is better","isMain":true,"metricName":"# N's per 100 kbp"}]],["Statistics without reference",[{"values":[3,4,5,4,4,1,90,39,1,2,2,2,5,1],"quality":"Equal","isMain":true,"metricName":"# contigs"},{"values":[3,2,5,3,3,1,47,25,1,2,2,2,5,1],"quality":"Equal","isMain":false,"metricName":"# contigs (>= 1000 bp)"},{"values":[2,1,3,2,2,1,0,7,1,1,1,1,3,1],"quality":"Equal","isMain":false,"metricName":"# contigs (>= 5000 bp)"},{"values":[1,1,2,1,1,1,0,0,1,1,1,1,2,1],"quality":"Equal","isMain":false,"metricName":"# contigs (>= 10000 bp)"},{"values":[1,1,2,1,1,1,0,0,1,1,1,1,2,1],"quality":"Equal","isMain":false,"metricName":"# contigs (>= 25000 bp)"},{"values":[1,1,1,1,1,1,0,0,1,1,1,1,1,1],"quality":"Equal","isMain":false,"metricName":"# contigs (>= 50000 bp)"},{"values":[116413,124904,82194,116359,116438,129456,4525,9504,129161,127023,127297,127297,70038,129608],"quality":"More is better","isMain":true,"metricName":"Largest contig"},{"values":[128978,127500,128163,128444,128849,129456,107628,105250,129161,128655,128791,128791,125867,129608],"quality":"More is better","isMain":true,"metricName":"Total length"},{"values":[128978,126431,128163,127848,128227,129456,75860,94813,129161,128655,128791,128791,125867,129608],"quality":"More is better","isMain":true,"metricName":"Total length (>= 1000 bp)"},{"values":[125955,124904,124529,125847,125063,129456,0,50268,129161,127023,127297,127297,123395,129608],"quality":"More is better","isMain":false,"metricName":"Total length (>= 5000 bp)"},{"values":[116413,124904,116225,116359,116438,129456,0,0,129161,127023,127297,127297,115130,129608],"quality":"More is better","isMain":true,"metricName":"Total length (>= 10000 bp)"},{"values":[116413,124904,116225,116359,116438,129456,0,0,129161,127023,127297,127297,115130,129608],"quality":"More is better","isMain":false,"metricName":"Total length (>= 25000 bp)"},{"values":[116413,124904,82194,116359,116438,129456,0,0,129161,127023,127297,127297,70038,129608],"quality":"More is better","isMain":true,"metricName":"Total length (>= 50000 bp)"},{"values":[116413,124904,82194,116359,116438,129456,1266,4691,129161,127023,127297,127297,70038,129608],"quality":"More is better","isMain":false,"metricName":"N50"},{"values":[116413,124904,34031,116359,116438,129456,955,2300,129161,127023,127297,127297,45092,129608],"quality":"More is better","isMain":false,"metricName":"N75"},{"values":[1,1,1,1,1,1,28,8,1,1,1,1,1,1],"quality":"Less is better","isMain":false,"metricName":"L50"},{"values":[1,1,2,1,1,1,52,15,1,1,1,1,2,1],"quality":"Less is better","isMain":false,"metricName":"L75"},{"values":["45.77","45.64","45.66","45.73","45.76","45.88","45.77","45.48","45.80","45.72","45.74","45.74","45.44","45.90"],"quality":"Equal","isMain":false,"metricName":"GC (%)"}]],["Predicted genes",[]],["Similarity statistics",[{"values":[0,0,0,0,0,0,0,0,0,0,0,0,0,0],"quality":"Equal","isMain":false,"metricName":"# similar correct contigs"},{"values":[0,0,0,0,0,0,0,0,0,0,0,0,0,0],"quality":"Equal","isMain":false,"metricName":"# similar misassembled blocks"}]],["Reference statistics",[{"values":[130608,130608,130608,130608,130608,130608,130608,130608,130608,130608,130608,130608,130608,130608],"quality":"Equal","isMain":false,"metricName":"Reference length"},{"values":[1,1,1,1,1,1,1,1,1,1,1,1,1,1],"quality":"Equal","isMain":false,"metricName":"Reference fragments"},{"values":["46.17","46.17","46.17","46.17","46.17","46.17","46.17","46.17","46.17","46.17","46.17","46.17","46.17","46.17"],"quality":"Equal","isMain":false,"metricName":"Reference GC (%)"}]]],"referenceName":"gi\_10140926\_ref\_NC","date":"18 July 2018, Wednesday, 16:04:50","order":[0,1,2,3,4,5,6,7,8,9,10,11,12,13],"assembliesNames":["ABySS\_127","ABySS\_63","CLC","IDBA\_UD","MEGAHIT","MIRA","Ray\_Meta","SOAPdenovo2","SPAdes","SPAdes\_meta","SPAdes\_sc","SPAdes\_sc\_careful","Velvet","Geneious"]},{"assembliesWithNs":null,"minContig":500,"report":[["Genome statistics",[{"values":["69.825","56.488","88.359","72.029","88.359","100.000","1.169","25.144","88.070","77.745","57.447","57.758","22.093","100.000"],"quality":"More is better","isMain":true,"metricName":"Genome fraction (%)"},{"values":["1.027","1.019","1.000","1.069","1.043","1.488","1.000","1.456","1.013","1.006","1.005","1.009","1.000","1.488"],"quality":"Less is better","isMain":true,"metricName":"Duplication ratio"},{"values":[7566,2623,11497,4437,6258,32865,519,1993,12908,10422,2774,2774,2035,38902],"quality":"More is better","isMain":true,"metricName":"Largest alignment"},{"values":[31823,25547,39224,34186,40887,66062,519,11160,39598,34675,25616,25864,9806,66035],"quality":"More is better","isMain":true,"metricName":"Total aligned length"},{"values":[2002,644,7271,1497,3370,44529,null,590,6082,4710,912,912,null,45094],"quality":"More is better","isMain":false,"metricName":"NG50"},{"values":[null,null,4396,794,1776,44529,null,null,4384,889,null,null,null,45094],"quality":"More is better","isMain":false,"metricName":"NG75"},{"values":[4618,1168,7271,1497,3370,27133,519,null,7568,9749,1289,1883,777,27133],"quality":"More is better","isMain":false,"metricName":"NA50"},{"values":[1746,928,5387,828,1776,null,519,null,5112,4099,1198,1198,627,null],"quality":"More is better","isMain":false,"metricName":"NA75"},{"values":[2002,644,7271,1237,3370,32865,null,null,6082,4657,912,912,null,38902],"quality":"More is better","isMain":true,"metricName":"NGA50"},{"values":[null,null,4396,588,1748,27133,null,null,4384,889,null,null,null,38902],"quality":"More is better","isMain":false,"metricName":"NGA75"},{"values":[5,18,3,7,5,1,null,17,3,3,14,13,null,1],"quality":"Less is better","isMain":false,"metricName":"LG50"},{"values":[null,null,5,17,9,1,null,null,5,7,null,null,null,1],"quality":"Less is better","isMain":false,"metricName":"LG75"},{"values":[3,8,3,7,5,2,1,null,2,2,6,6,5,2],"quality":"Less is better","isMain":false,"metricName":"LA50"},{"values":[6,14,4,16,9,null,1,null,4,4,11,10,8,null],"quality":"Less is better","isMain":false,"metricName":"LA75"},{"values":[5,18,3,10,5,1,null,null,3,3,14,13,null,1],"quality":"Less is better","isMain":true,"metricName":"LGA50"},{"values":[null,null,5,23,10,2,null,null,5,7,null,null,null,1],"quality":"Less is better","isMain":false,"metricName":"LGA75"}]],["Misassemblies",[{"values":[0,0,0,0,0,0,0,0,0,0,0,0,0,0],"quality":"Less is better","isMain":true,"metricName":"# misassemblies"},{"values":[0,0,0,0,0,0,0,0,0,0,0,0,0,0],"quality":"Less is better","isMain":false,"metricName":" # relocations"},{"values":[0,0,0,0,0,0,0,0,0,0,0,0,0,0],"quality":"Less is better","isMain":false,"metricName":" # translocations"},{"values":[0,0,0,0,0,0,0,0,0,0,0,0,0,0],"quality":"Less is better","isMain":false,"metricName":" # inversions"},{"values":[0,0,0,0,0,0,0,0,0,0,0,0,0,0],"quality":"Less is better","isMain":false,"metricName":"# misassembled contigs"},{"values":[0,0,0,0,0,0,0,0,0,0,0,0,0,0],"quality":"Less is better","isMain":true,"metricName":"Misassembled contigs length"},{"values":[0,0,0,0,0,4,0,0,0,0,0,0,0,4],"quality":"Less is better","isMain":false,"metricName":"# local misassemblies"},{"values":[0,0,0,0,0,0,0,8,0,0,0,0,0,0],"quality":"Less is better","isMain":false,"metricName":"# unaligned mis. contigs"}]],["Unaligned",[{"values":[0,0,0,0,0,0,0,0,0,0,0,0,0,0],"quality":"Less is better","isMain":false,"metricName":"# fully unaligned contigs"},{"values":[0,0,0,0,0,0,0,0,0,0,0,0,0,0],"quality":"Less is better","isMain":false,"metricName":"Fully unaligned length"},{"values":[0,0,0,1,1,2,0,7,0,0,0,0,0,2],"quality":"Less is better","isMain":false,"metricName":"# partially unaligned contigs"},{"values":[0,0,0,3839,1338,23588,0,8149,0,0,0,0,0,23588],"quality":"Less is better","isMain":false,"metricName":"Partially unaligned length"}]],["Mismatches",[{"values":[1,24,2,101,30,637,0,2,0,270,1,8,3,579],"quality":"Less is better","isMain":false,"metricName":"# mismatches"},{"values":[0,0,0,2,0,18,0,11,0,10,0,0,0,12],"quality":"Less is better","isMain":false,"metricName":"# indels"},{"values":[0,0,0,3,0,22,0,527,0,11,0,0,0,14],"quality":"Less is better","isMain":false,"metricName":"Indels length"},{"values":["3.23","95.72","5.10","315.92","76.50","1435.17","0.00","17.92","0.00","782.45","3.92","31.21","30.59","1304.49"],"quality":"Less is better","isMain":true,"metricName":"# mismatches per 100 kbp"},{"values":["0.00","0.00","0.00","6.26","0.00","40.55","0.00","98.57","0.00","28.98","0.00","0.00","0.00","27.04"],"quality":"Less is better","isMain":true,"metricName":"# indels per 100 kbp"},{"values":[0,0,0,2,0,18,0,0,0,10,0,0,0,12],"quality":"Less is better","isMain":false,"metricName":" # indels (<= 5 bp)"},{"values":[0,0,0,0,0,0,0,11,0,0,0,0,0,0],"quality":"Less is better","isMain":false,"metricName":" # indels (> 5 bp)"},{"values":[0,0,0,0,0,2,0,8685,0,0,0,0,0,2],"quality":"Less is better","isMain":false,"metricName":"# N's"},{"values":["0.00","0.00","0.00","0.00","0.00","2.23","0.00","35594.26","0.00","0.00","0.00","0.00","0.00","2.23"],"quality":"Less is better","isMain":true,"metricName":"# N's per 100 kbp"}]],["Statistics without reference",[{"values":[14,23,8,24,16,3,1,21,9,9,19,18,12,2],"quality":"Equal","isMain":true,"metricName":"# contigs"},{"values":[9,12,7,13,12,3,0,8,6,6,12,11,2,2],"quality":"Equal","isMain":false,"metricName":"# contigs (>= 1000 bp)"},{"values":[2,0,4,1,2,3,0,0,4,2,0,0,0,2],"quality":"Equal","isMain":false,"metricName":"# contigs (>= 5000 bp)"},{"values":[0,0,1,0,0,2,0,0,1,1,0,0,0,2],"quality":"Equal","isMain":false,"metricName":"# contigs (>= 10000 bp)"},{"values":[0,0,0,0,0,2,0,0,0,0,0,0,0,2],"quality":"Equal","isMain":false,"metricName":"# contigs (>= 25000 bp)"},{"values":[0,0,0,0,0,0,0,0,0,0,0,0,0,0],"quality":"Equal","isMain":false,"metricName":"# contigs (>= 50000 bp)"},{"values":[7566,2623,11497,8276,7596,44529,519,4789,12908,10422,2774,2774,2035,45094],"quality":"More is better","isMain":true,"metricName":"Largest contig"},{"values":[31823,25547,39224,38025,42225,89650,519,24400,39598,34728,25616,25864,9806,89623],"quality":"More is better","isMain":true,"metricName":"Total length"},{"values":[28509,17796,38584,30288,38914,89650,0,15218,37467,32681,20749,20708,3038,89623],"quality":"More is better","isMain":true,"metricName":"Total length (>= 1000 bp)"},{"values":[13362,0,31512,8276,13066,89650,0,0,31670,20171,0,0,0,89623],"quality":"More is better","isMain":false,"metricName":"Total length (>= 5000 bp)"},{"values":[0,0,11497,0,0,83586,0,0,12908,10422,0,0,0,89623],"quality":"More is better","isMain":true,"metricName":"Total length (>= 10000 bp)"},{"values":[0,0,0,0,0,83586,0,0,0,0,0,0,0,89623],"quality":"More is better","isMain":false,"metricName":"Total length (>= 25000 bp)"},{"values":[0,0,0,0,0,0,0,0,0,0,0,0,0,0],"quality":"More is better","isMain":true,"metricName":"Total length (>= 50000 bp)"},{"values":[4618,1168,7271,1948,4301,39057,519,1215,7568,9749,1289,1883,777,45094],"quality":"More is better","isMain":false,"metricName":"N50"},{"values":[1746,928,5387,1173,1927,39057,519,852,5112,4099,1198,1198,627,44529],"quality":"More is better","isMain":false,"metricName":"N75"},{"values":[3,8,3,5,4,2,1,6,2,2,6,6,5,1],"quality":"Less is better","isMain":false,"metricName":"L50"},{"values":[6,14,4,12,8,2,1,12,4,4,11,10,8,2],"quality":"Less is better","isMain":false,"metricName":"L75"},{"values":["45.07","44.85","45.20","44.97","45.06","45.15","44.89","45.87","45.16","45.15","44.92","45.40","44.43","45.14"],"quality":"Equal","isMain":false,"metricName":"GC (%)"}]],["Predicted genes",[]],["Similarity statistics",[{"values":[0,0,0,0,0,0,0,0,0,0,0,0,0,0],"quality":"Equal","isMain":false,"metricName":"# similar correct contigs"},{"values":[0,0,0,0,0,0,0,0,0,0,0,0,0,0],"quality":"Equal","isMain":false,"metricName":"# similar misassembled blocks"}]],["Reference statistics",[{"values":[44385,44385,44385,44385,44385,44385,44385,44385,44385,44385,44385,44385,44385,44385],"quality":"Equal","isMain":false,"metricName":"Reference length"},{"values":[1,1,1,1,1,1,1,1,1,1,1,1,1,1],"quality":"Equal","isMain":false,"metricName":"Reference fragments"},{"values":["45.25","45.25","45.25","45.25","45.25","45.25","45.25","45.25","45.25","45.25","45.25","45.25","45.25","45.25"],"quality":"Equal","isMain":false,"metricName":"Reference GC (%)"}]]],"referenceName":"gi\_108861984\_ref\_NC","date":"18 July 2018, Wednesday, 16:05:01","order":[0,1,2,3,4,5,6,7,8,9,10,11,12,13],"assembliesNames":["ABySS\_127","ABySS\_63","CLC","IDBA\_UD","MEGAHIT","MIRA","Ray\_Meta","SOAPdenovo2","SPAdes","SPAdes\_meta","SPAdes\_sc","SPAdes\_sc\_careful","Velvet","Geneious"]},{"assembliesWithNs":null,"minContig":500,"report":[["Genome statistics",[{"values":["93.904","87.920","97.369","98.333","98.267","98.005","96.698","98.605","99.012","98.246","98.246","97.905","46.200"],"quality":"More is better","isMain":true,"metricName":"Genome fraction (%)"},{"values":["1.003","1.002","1.009","1.001","1.000","1.004","1.015","1.000","1.001","1.000","1.000","1.001","1.000"],"quality":"Less is better","isMain":true,"metricName":"Duplication ratio"},{"values":[9098,5767,47123,42239,69688,22384,41759,69798,65942,69809,69798,33833,20359],"quality":"More is better","isMain":true,"metricName":"Largest alignment"},{"values":[98596,92272,102862,103054,102907,103026,101854,103262,103792,102903,102892,102591,48376],"quality":"More is better","isMain":true,"metricName":"Total aligned length"},{"values":[3036,1954,22975,20500,69688,10632,7921,69798,65942,69809,69798,11670,null],"quality":"More is better","isMain":false,"metricName":"NG50"},{"values":[1378,965,11459,18965,19007,8379,5138,33464,33839,33094,33094,6800,null],"quality":"More is better","isMain":false,"metricName":"NG75"},{"values":[3173,2331,22975,20500,69688,10632,7680,69798,65942,69809,69798,11670,16549],"quality":"More is better","isMain":false,"metricName":"NA50"},{"values":[1735,1308,11459,18965,19007,8379,5023,33464,33839,33094,33094,6800,16549],"quality":"More is better","isMain":false,"metricName":"NA75"},{"values":[3036,1954,22975,20500,69688,10632,7680,69798,65942,69809,69798,11670,null],"quality":"More is better","isMain":true,"metricName":"NGA50"},{"values":[1378,965,11459,18965,19007,8379,5023,33464,33839,33094,33094,6800,null],"quality":"More is better","isMain":false,"metricName":"NGA75"},{"values":[11,15,2,2,1,3,3,1,1,1,1,3,null],"quality":"Less is better","isMain":false,"metricName":"LG50"},{"values":[24,34,3,3,2,6,7,2,2,2,2,5,null],"quality":"Less is better","isMain":false,"metricName":"LG75"},{"values":[10,12,2,2,1,3,3,1,1,1,1,3,2],"quality":"Less is better","isMain":false,"metricName":"LA50"},{"values":[21,26,3,3,2,6,7,2,2,2,2,5,2],"quality":"Less is better","isMain":false,"metricName":"LA75"},{"values":[11,15,2,2,1,3,3,1,1,1,1,3,null],"quality":"Less is better","isMain":true,"metricName":"LGA50"},{"values":[24,34,3,3,2,6,7,2,2,2,2,5,null],"quality":"Less is better","isMain":false,"metricName":"LGA75"}]],["Misassemblies",[{"values":[0,0,0,0,0,0,0,0,0,0,0,0,0],"quality":"Less is better","isMain":true,"metricName":"# misassemblies"},{"values":[0,0,0,0,0,0,0,0,0,0,0,0,0],"quality":"Less is better","isMain":false,"metricName":" # relocations"},{"values":[0,0,0,0,0,0,0,0,0,0,0,0,0],"quality":"Less is better","isMain":false,"metricName":" # translocations"},{"values":[0,0,0,0,0,0,0,0,0,0,0,0,0],"quality":"Less is better","isMain":false,"metricName":" # inversions"},{"values":[0,0,0,0,0,0,0,0,0,0,0,0,0],"quality":"Less is better","isMain":false,"metricName":"# misassembled contigs"},{"values":[0,0,0,0,0,0,0,0,0,0,0,0,0],"quality":"Less is better","isMain":true,"metricName":"Misassembled contigs length"},{"values":[0,0,0,0,0,0,0,0,0,0,0,0,0],"quality":"Less is better","isMain":false,"metricName":"# local misassemblies"},{"values":[0,0,0,0,0,0,0,0,0,0,0,0,0],"quality":"Less is better","isMain":false,"metricName":"# unaligned mis. contigs"}]],["Unaligned",[{"values":[0,0,0,0,0,0,0,0,0,0,0,0,0],"quality":"Less is better","isMain":false,"metricName":"# fully unaligned contigs"},{"values":[0,0,0,0,0,0,0,0,0,0,0,0,0],"quality":"Less is better","isMain":false,"metricName":"Fully unaligned length"},{"values":[0,0,0,0,0,0,1,0,0,0,0,0,0],"quality":"Less is better","isMain":false,"metricName":"# partially unaligned contigs"},{"values":[0,0,0,0,0,0,616,0,0,0,0,0,0],"quality":"Less is better","isMain":false,"metricName":"Partially unaligned length"}]],["Mismatches",[{"values":[9,1,12,8,9,5,10,17,20,13,7,11,4],"quality":"Less is better","isMain":false,"metricName":"# mismatches"},{"values":[0,0,3,1,0,0,19,0,5,3,2,0,0],"quality":"Less is better","isMain":false,"metricName":"# indels"},{"values":[0,0,29,42,0,0,888,0,141,119,108,0,0],"quality":"Less is better","isMain":false,"metricName":"Indels length"},{"values":["9.15","1.09","11.77","7.77","8.75","4.87","9.88","16.47","19.29","12.64","6.80","10.73","8.27"],"quality":"Less is better","isMain":true,"metricName":"# mismatches per 100 kbp"},{"values":["0.00","0.00","2.94","0.97","0.00","0.00","18.77","0.00","4.82","2.92","1.94","0.00","0.00"],"quality":"Less is better","isMain":true,"metricName":"# indels per 100 kbp"},{"values":[0,0,2,0,0,0,0,0,1,0,0,0,0],"quality":"Less is better","isMain":false,"metricName":" # indels (<= 5 bp)"},{"values":[0,0,1,1,0,0,19,0,4,3,2,0,0],"quality":"Less is better","isMain":false,"metricName":" # indels (> 5 bp)"},{"values":[0,0,0,0,0,27,1673,0,0,10,0,0,14],"quality":"Less is better","isMain":false,"metricName":"# N's"},{"values":["0.00","0.00","0.00","0.00","0.00","26.21","1618.16","0.00","0.00","9.72","0.00","0.00","28.94"],"quality":"Less is better","isMain":true,"metricName":"# N's per 100 kbp"}]],["Statistics without reference",[{"values":[44,52,7,6,3,12,16,2,3,2,2,12,3],"quality":"Equal","isMain":true,"metricName":"# contigs"},{"values":[33,33,7,6,3,12,15,2,3,2,2,12,3],"quality":"Equal","isMain":false,"metricName":"# contigs (>= 1000 bp)"},{"values":[3,2,5,4,3,8,7,2,2,2,2,6,3],"quality":"Equal","isMain":false,"metricName":"# contigs (>= 5000 bp)"},{"values":[0,0,3,4,3,4,1,2,2,2,2,4,3],"quality":"Equal","isMain":false,"metricName":"# contigs (>= 10000 bp)"},{"values":[0,0,1,1,1,0,1,2,2,2,2,1,0],"quality":"Equal","isMain":false,"metricName":"# contigs (>= 25000 bp)"},{"values":[0,0,0,0,1,0,0,1,1,1,1,0,0],"quality":"Equal","isMain":false,"metricName":"# contigs (>= 50000 bp)"},{"values":[9098,5767,47123,42239,69688,22384,41951,69798,65942,69809,69798,33833,20359],"quality":"More is better","isMain":true,"metricName":"Largest contig"},{"values":[98596,92272,102862,103054,102907,103026,103389,103262,103792,102903,102892,102591,48376],"quality":"More is better","isMain":true,"metricName":"Total length"},{"values":[89999,77813,102862,103054,102907,103026,102797,103262,103792,102903,102892,102591,48376],"quality":"More is better","isMain":true,"metricName":"Total length (>= 1000 bp)"},{"values":[23025,11371,97613,96046,102907,94179,81888,103262,99781,102903,102892,84688,48376],"quality":"More is better","isMain":false,"metricName":"Total length (>= 5000 bp)"},{"values":[0,0,81557,96046,102907,63987,41951,103262,99781,102903,102892,72822,48376],"quality":"More is better","isMain":true,"metricName":"Total length (>= 10000 bp)"},{"values":[0,0,47123,42239,69688,0,41951,103262,99781,102903,102892,33833,0],"quality":"More is better","isMain":false,"metricName":"Total length (>= 25000 bp)"},{"values":[0,0,0,0,69688,0,0,69798,65942,69809,69798,0,0],"quality":"More is better","isMain":true,"metricName":"Total length (>= 50000 bp)"},{"values":[3173,2331,22975,20500,69688,10632,7921,69798,65942,69809,69798,11670,16549],"quality":"More is better","isMain":false,"metricName":"N50"},{"values":[1735,1308,11459,18965,19007,8379,5138,33464,33839,33094,33094,6800,16549],"quality":"More is better","isMain":false,"metricName":"N75"},{"values":[10,12,2,2,1,3,3,1,1,1,1,3,2],"quality":"Less is better","isMain":false,"metricName":"L50"},{"values":[21,26,3,3,2,6,7,2,2,2,2,5,2],"quality":"Less is better","isMain":false,"metricName":"L75"},{"values":["32.64","32.86","32.85","32.85","32.86","32.67","32.91","32.82","32.78","32.87","32.87","32.89","33.20"],"quality":"Equal","isMain":false,"metricName":"GC (%)"}]],["Predicted genes",[]],["Similarity statistics",[{"values":[0,0,0,0,0,0,0,0,0,0,0,0,0],"quality":"Equal","isMain":false,"metricName":"# similar correct contigs"},{"values":[0,0,0,0,0,0,0,0,0,0,0,0,0],"quality":"Equal","isMain":false,"metricName":"# similar misassembled blocks"}]],["Reference statistics",[{"values":[104710,104710,104710,104710,104710,104710,104710,104710,104710,104710,104710,104710,104710],"quality":"Equal","isMain":false,"metricName":"Reference length"},{"values":[1,1,1,1,1,1,1,1,1,1,1,1,1],"quality":"Equal","isMain":false,"metricName":"Reference fragments"},{"values":["32.67","32.67","32.67","32.67","32.67","32.67","32.67","32.67","32.67","32.67","32.67","32.67","32.67"],"quality":"Equal","isMain":false,"metricName":"Reference GC (%)"}]]],"referenceName":"gi\_109255272\_ref\_NC","date":"18 July 2018, Wednesday, 16:05:11","order":[0,1,2,3,4,5,6,7,8,9,10,11,12],"assembliesNames":["ABySS\_127","ABySS\_63","CLC","IDBA\_UD","MEGAHIT","MIRA","SOAPdenovo2","SPAdes","SPAdes\_meta","SPAdes\_sc","SPAdes\_sc\_careful","Velvet","Geneious"]},{"assembliesWithNs":null,"minContig":500,"report":[["Genome statistics",[{"values":["95.332","90.004","90.241","97.586","95.361","98.803","0.285","79.773","99.101","98.347","89.821","89.788","82.239","81.986"],"quality":"More is better","isMain":true,"metricName":"Genome fraction (%)"},{"values":["1.004","1.006","1.008","1.003","1.002","1.032","1.000","1.044","1.005","1.002","1.003","1.003","1.001","1.010"],"quality":"Less is better","isMain":true,"metricName":"Duplication ratio"},{"values":[17617,7345,22772,43111,23802,25140,544,18347,78360,74238,22035,22035,18370,37817],"quality":"More is better","isMain":true,"metricName":"Largest alignment"},{"values":[182964,173011,172599,187067,182667,194553,544,153228,190222,188242,171925,171923,157252,157960],"quality":"More is better","isMain":true,"metricName":"Total aligned length"},{"values":[4022,2874,15405,28370,15064,24294,null,7491,181334,51932,14477,14475,7558,30458],"quality":"More is better","isMain":false,"metricName":"NG50"},{"values":[2476,1504,6419,21396,9343,8751,null,2851,181334,25133,8053,8053,2097,8751],"quality":"More is better","isMain":false,"metricName":"NG75"},{"values":[4248,3133,15405,28370,15064,12922,544,8460,72104,51932,15084,15084,9724,23441],"quality":"More is better","isMain":false,"metricName":"NA50"},{"values":[2852,1758,7579,21396,10356,6777,544,3904,72104,25133,9674,9674,4368,10989],"quality":"More is better","isMain":false,"metricName":"NA75"},{"values":[4022,2874,10833,28370,15064,12922,null,6223,72104,51932,14218,14216,7558,12922],"quality":"More is better","isMain":true,"metricName":"NGA50"},{"values":[2476,1504,5312,21396,9343,7474,null,1919,72104,25133,8053,8053,2097,6153],"quality":"More is better","isMain":false,"metricName":"NGA75"},{"values":[14,20,5,3,5,3,null,8,1,2,6,6,8,3],"quality":"Less is better","isMain":false,"metricName":"LG50"},{"values":[28,43,11,5,10,7,null,19,1,3,10,10,20,5],"quality":"Less is better","isMain":false,"metricName":"LG75"},{"values":[13,17,5,3,5,5,1,7,2,2,5,5,6,3],"quality":"Less is better","isMain":false,"metricName":"LA50"},{"values":[26,35,10,5,9,11,1,14,2,3,9,9,12,6],"quality":"Less is better","isMain":false,"metricName":"LA75"},{"values":[14,20,6,3,5,5,null,9,2,2,6,6,8,4],"quality":"Less is better","isMain":true,"metricName":"LGA50"},{"values":[28,43,12,5,10,10,null,22,2,3,10,10,20,9],"quality":"Less is better","isMain":false,"metricName":"LGA75"}]],["Misassemblies",[{"values":[0,0,3,0,0,5,0,0,2,0,0,0,0,5],"quality":"Less is better","isMain":true,"metricName":"# misassemblies"},{"values":[0,0,3,0,0,5,0,0,2,0,0,0,0,5],"quality":"Less is better","isMain":false,"metricName":" # relocations"},{"values":[0,0,0,0,0,0,0,0,0,0,0,0,0,0],"quality":"Less is better","isMain":false,"metricName":" # translocations"},{"values":[0,0,0,0,0,0,0,0,0,0,0,0,0,0],"quality":"Less is better","isMain":false,"metricName":" # inversions"},{"values":[0,0,3,0,0,2,0,0,1,0,0,0,0,2],"quality":"Less is better","isMain":false,"metricName":"# misassembled contigs"},{"values":[0,0,40812,0,0,63908,0,0,181334,0,0,0,0,95370],"quality":"Less is better","isMain":true,"metricName":"Misassembled contigs length"},{"values":[0,0,0,0,1,2,0,2,3,0,0,0,0,2],"quality":"Less is better","isMain":false,"metricName":"# local misassemblies"},{"values":[0,0,0,0,0,0,0,1,0,0,0,0,0,0],"quality":"Less is better","isMain":false,"metricName":"# unaligned mis. contigs"}]],["Unaligned",[{"values":[0,0,0,0,0,0,0,0,0,0,0,0,0,0],"quality":"Less is better","isMain":false,"metricName":"# fully unaligned contigs"},{"values":[0,0,0,0,0,0,0,0,0,0,0,0,0,0],"quality":"Less is better","isMain":false,"metricName":"Fully unaligned length"},{"values":[0,0,0,0,0,0,0,1,1,0,0,0,0,0],"quality":"Less is better","isMain":false,"metricName":"# partially unaligned contigs"},{"values":[0,0,0,0,0,0,0,2325,903,0,0,0,0,0],"quality":"Less is better","isMain":false,"metricName":"Partially unaligned length"}]],["Mismatches",[{"values":[14,5,42,4,9,84,0,4,29,14,13,7,12,78],"quality":"Less is better","isMain":false,"metricName":"# mismatches"},{"values":[2,0,13,2,0,33,0,50,3,0,6,6,2,34],"quality":"Less is better","isMain":false,"metricName":"# indels"},{"values":[46,0,98,46,0,128,0,2439,3,0,164,166,16,91],"quality":"Less is better","isMain":false,"metricName":"Indels length"},{"values":["7.68","2.91","24.35","2.14","4.94","44.49","0.00","2.62","15.31","7.45","7.57","4.08","7.64","49.78"],"quality":"Less is better","isMain":true,"metricName":"# mismatches per 100 kbp"},{"values":["1.10","0.00","7.54","1.07","0.00","17.48","0.00","32.80","1.58","0.00","3.50","3.50","1.27","21.70"],"quality":"Less is better","isMain":true,"metricName":"# indels per 100 kbp"},{"values":[0,0,11,0,0,29,0,0,3,0,2,2,1,31],"quality":"Less is better","isMain":false,"metricName":" # indels (<= 5 bp)"},{"values":[2,0,2,2,0,4,0,50,0,0,4,4,1,3],"quality":"Less is better","isMain":false,"metricName":" # indels (> 5 bp)"},{"values":[0,0,0,0,0,47,0,7534,1003,0,10,0,0,44],"quality":"Less is better","isMain":false,"metricName":"# N's"},{"values":["0.00","0.00","0.00","0.00","0.00","24.12","0.00","4665.62","524.51","0.00","5.81","0.00","0.00","27.82"],"quality":"Less is better","isMain":true,"metricName":"# N's per 100 kbp"}]],["Statistics without reference",[{"values":[58,77,24,16,26,21,1,31,4,7,27,27,31,7],"quality":"Equal","isMain":true,"metricName":"# contigs"},{"values":[47,52,20,10,17,16,0,27,3,5,16,16,26,7],"quality":"Equal","isMain":false,"metricName":"# contigs (>= 1000 bp)"},{"values":[10,10,12,7,12,13,0,10,2,5,10,10,10,7],"quality":"Equal","isMain":false,"metricName":"# contigs (>= 5000 bp)"},{"values":[1,0,6,7,9,5,0,5,1,5,8,8,5,4],"quality":"Equal","isMain":false,"metricName":"# contigs (>= 10000 bp)"},{"values":[0,0,1,3,0,2,0,0,1,4,0,0,0,3],"quality":"Equal","isMain":false,"metricName":"# contigs (>= 25000 bp)"},{"values":[0,0,0,0,0,1,0,0,1,2,0,0,0,1],"quality":"Equal","isMain":false,"metricName":"# contigs (>= 50000 bp)"},{"values":[17617,7345,27071,43111,23802,51400,544,18991,181334,74238,22035,22035,18370,51400],"quality":"More is better","isMain":true,"metricName":"Largest contig"},{"values":[182964,173011,173748,187067,182667,194841,544,161479,191225,188242,172184,172182,157252,158182],"quality":"More is better","isMain":true,"metricName":"Total length"},{"values":[175252,155664,170859,182584,176379,191047,0,158624,190268,186795,163819,163817,153621,158182],"quality":"More is better","isMain":true,"metricName":"Total length (>= 1000 bp)"},{"values":[81386,61031,154930,174508,165958,185331,0,108371,189110,186795,145179,145177,109872,158182],"quality":"More is better","isMain":false,"metricName":"Total length (>= 5000 bp)"},{"values":[17617,0,109726,174508,143114,129854,0,71400,181334,186795,127452,127450,73222,136817],"quality":"More is better","isMain":true,"metricName":"Total length (>= 10000 bp)"},{"values":[0,0,27071,100641,0,76540,0,0,181334,176356,0,0,0,125828],"quality":"More is better","isMain":false,"metricName":"Total length (>= 25000 bp)"},{"values":[0,0,0,0,0,51400,0,0,181334,126170,0,0,0,51400],"quality":"More is better","isMain":true,"metricName":"Total length (>= 50000 bp)"},{"values":[4248,3133,15405,28370,15064,24294,544,8460,181334,51932,15084,15084,9724,43970],"quality":"More is better","isMain":false,"metricName":"N50"},{"values":[2852,1758,7946,21396,10356,8751,544,4641,181334,25133,9674,9674,4368,30458],"quality":"More is better","isMain":false,"metricName":"N75"},{"values":[13,17,5,3,5,3,1,7,1,2,5,5,6,2],"quality":"Less is better","isMain":false,"metricName":"L50"},{"values":[26,35,9,5,9,7,1,13,1,3,9,9,12,3],"quality":"Less is better","isMain":false,"metricName":"L75"},{"values":["47.99","48.19","48.41","48.01","48.14","47.79","49.82","48.98","47.92","47.97","48.47","48.47","48.89","47.88"],"quality":"Equal","isMain":false,"metricName":"GC (%)"}]],["Predicted genes",[]],["Similarity statistics",[{"values":[0,0,0,0,0,0,0,0,0,0,0,0,0,0],"quality":"Equal","isMain":false,"metricName":"# similar correct contigs"},{"values":[0,0,0,0,0,0,0,0,0,0,0,0,0,0],"quality":"Equal","isMain":false,"metricName":"# similar misassembled blocks"}]],["Reference statistics",[{"values":[191100,191100,191100,191100,191100,191100,191100,191100,191100,191100,191100,191100,191100,191100],"quality":"Equal","isMain":false,"metricName":"Reference length"},{"values":[1,1,1,1,1,1,1,1,1,1,1,1,1,1],"quality":"Equal","isMain":false,"metricName":"Reference fragments"},{"values":["47.89","47.89","47.89","47.89","47.89","47.89","47.89","47.89","47.89","47.89","47.89","47.89","47.89","47.89"],"quality":"Equal","isMain":false,"metricName":"Reference GC (%)"}]]],"referenceName":"gi\_109287878\_ref\_NC","date":"18 July 2018, Wednesday, 16:05:23","order":[0,1,2,3,4,5,6,7,8,9,10,11,12,13],"assembliesNames":["ABySS\_127","ABySS\_63","CLC","IDBA\_UD","MEGAHIT","MIRA","Ray\_Meta","SOAPdenovo2","SPAdes","SPAdes\_meta","SPAdes\_sc","SPAdes\_sc\_careful","Velvet","Geneious"]},{"assembliesWithNs":null,"minContig":500,"report":[["Genome statistics",[{"values":["93.486","85.497","100.000","100.000","100.000","98.786","98.841","100.000","100.000","100.000","100.000","99.968","80.258"],"quality":"More is better","isMain":true,"metricName":"Genome fraction (%)"},{"values":["1.002","1.003","1.001","1.003","1.004","1.036","1.016","1.004","1.002","1.002","1.002","1.000","1.000"],"quality":"Less is better","isMain":true,"metricName":"Duplication ratio"},{"values":[8101,4507,34543,34624,34666,16683,15551,34652,34580,34580,34580,34514,16683],"quality":"More is better","isMain":true,"metricName":"Largest alignment"},{"values":[32329,29599,34543,34624,34666,35328,34199,34652,34580,34580,34580,34514,27709],"quality":"More is better","isMain":true,"metricName":"Total aligned length"},{"values":[1796,1451,34543,34624,34666,11026,9199,34652,34580,34580,34580,34514,11026],"quality":"More is better","isMain":false,"metricName":"NG50"},{"values":[1367,817,34543,34624,34666,11026,4330,34652,34580,34580,34580,34514,11026],"quality":"More is better","isMain":false,"metricName":"NG75"},{"values":[1989,1674,34543,34624,34666,11026,9199,34652,34580,34580,34580,34514,16683],"quality":"More is better","isMain":false,"metricName":"NA50"},{"values":[1469,1317,34543,34624,34666,11026,4237,34652,34580,34580,34580,34514,11026],"quality":"More is better","isMain":false,"metricName":"NA75"},{"values":[1796,1451,34543,34624,34666,11026,9199,34652,34580,34580,34580,34514,11026],"quality":"More is better","isMain":true,"metricName":"NGA50"},{"values":[1367,817,34543,34624,34666,11026,4237,34652,34580,34580,34580,34514,11026],"quality":"More is better","isMain":false,"metricName":"NGA75"},{"values":[6,8,1,1,1,2,2,1,1,1,1,1,2],"quality":"Less is better","isMain":false,"metricName":"LG50"},{"values":[11,15,1,1,1,2,3,1,1,1,1,1,2],"quality":"Less is better","isMain":false,"metricName":"LG75"},{"values":[5,6,1,1,1,2,2,1,1,1,1,1,1],"quality":"Less is better","isMain":false,"metricName":"LA50"},{"values":[10,11,1,1,1,2,3,1,1,1,1,1,2],"quality":"Less is better","isMain":false,"metricName":"LA75"},{"values":[6,8,1,1,1,2,2,1,1,1,1,1,2],"quality":"Less is better","isMain":true,"metricName":"LGA50"},{"values":[11,15,1,1,1,2,3,1,1,1,1,1,2],"quality":"Less is better","isMain":false,"metricName":"LGA75"}]],["Misassemblies",[{"values":[0,0,0,0,0,0,0,0,0,0,0,0,0],"quality":"Less is better","isMain":true,"metricName":"# misassemblies"},{"values":[0,0,0,0,0,0,0,0,0,0,0,0,0],"quality":"Less is better","isMain":false,"metricName":" # relocations"},{"values":[0,0,0,0,0,0,0,0,0,0,0,0,0],"quality":"Less is better","isMain":false,"metricName":" # translocations"},{"values":[0,0,0,0,0,0,0,0,0,0,0,0,0],"quality":"Less is better","isMain":false,"metricName":" # inversions"},{"values":[0,0,0,0,0,0,0,0,0,0,0,0,0],"quality":"Less is better","isMain":false,"metricName":"# misassembled contigs"},{"values":[0,0,0,0,0,0,0,0,0,0,0,0,0],"quality":"Less is better","isMain":true,"metricName":"Misassembled contigs length"},{"values":[0,0,0,1,1,0,0,1,0,0,0,0,0],"quality":"Less is better","isMain":false,"metricName":"# local misassemblies"},{"values":[0,0,0,0,0,0,0,0,0,0,0,0,0],"quality":"Less is better","isMain":false,"metricName":"# unaligned mis. contigs"}]],["Unaligned",[{"values":[0,0,0,0,0,0,0,0,0,0,0,0,0],"quality":"Less is better","isMain":false,"metricName":"# fully unaligned contigs"},{"values":[0,0,0,0,0,0,0,0,0,0,0,0,0],"quality":"Less is better","isMain":false,"metricName":"Fully unaligned length"},{"values":[0,0,0,0,0,0,0,0,0,0,0,0,0],"quality":"Less is better","isMain":false,"metricName":"# partially unaligned contigs"},{"values":[0,0,0,0,0,0,0,0,0,0,0,0,0],"quality":"Less is better","isMain":false,"metricName":"Partially unaligned length"}]],["Mismatches",[{"values":[3,1,3,0,0,0,1,9,5,2,0,1,0],"quality":"Less is better","isMain":false,"metricName":"# mismatches"},{"values":[0,0,1,0,0,0,5,0,1,1,1,0,0],"quality":"Less is better","isMain":false,"metricName":"# indels"},{"values":[0,0,18,0,0,0,227,0,55,55,55,0,0],"quality":"Less is better","isMain":false,"metricName":"Indels length"},{"values":["9.29","3.39","8.69","0.00","0.00","0.00","2.93","26.07","14.48","5.79","0.00","2.90","0.00"],"quality":"Less is better","isMain":true,"metricName":"# mismatches per 100 kbp"},{"values":["0.00","0.00","2.90","0.00","0.00","0.00","14.65","0.00","2.90","2.90","2.90","0.00","0.00"],"quality":"Less is better","isMain":true,"metricName":"# indels per 100 kbp"},{"values":[0,0,0,0,0,0,0,0,0,0,0,0,0],"quality":"Less is better","isMain":false,"metricName":" # indels (<= 5 bp)"},{"values":[0,0,1,0,0,0,5,0,1,1,1,0,0],"quality":"Less is better","isMain":false,"metricName":" # indels (> 5 bp)"},{"values":[0,0,0,0,0,6,526,0,0,0,0,0,3],"quality":"Less is better","isMain":false,"metricName":"# N's"},{"values":["0.00","0.00","0.00","0.00","0.00","16.98","1516.64","0.00","0.00","0.00","0.00","0.00","10.83"],"quality":"Less is better","isMain":true,"metricName":"# N's per 100 kbp"}]],["Statistics without reference",[{"values":[17,20,1,1,1,5,5,1,1,1,1,1,2],"quality":"Equal","isMain":true,"metricName":"# contigs"},{"values":[13,12,1,1,1,3,5,1,1,1,1,1,2],"quality":"Equal","isMain":false,"metricName":"# contigs (>= 1000 bp)"},{"values":[1,0,1,1,1,3,2,1,1,1,1,1,2],"quality":"Equal","isMain":false,"metricName":"# contigs (>= 5000 bp)"},{"values":[0,0,1,1,1,2,1,1,1,1,1,1,2],"quality":"Equal","isMain":false,"metricName":"# contigs (>= 10000 bp)"},{"values":[0,0,1,1,1,0,0,1,1,1,1,1,0],"quality":"Equal","isMain":false,"metricName":"# contigs (>= 25000 bp)"},{"values":[0,0,0,0,0,0,0,0,0,0,0,0,0],"quality":"Equal","isMain":false,"metricName":"# contigs (>= 50000 bp)"},{"values":[8101,4507,34543,34624,34666,16683,15941,34652,34580,34580,34580,34514,16683],"quality":"More is better","isMain":true,"metricName":"Largest contig"},{"values":[32329,29599,34543,34624,34666,35328,34682,34652,34580,34580,34580,34514,27709],"quality":"More is better","isMain":true,"metricName":"Total length"},{"values":[29114,23589,34543,34624,34666,34106,34682,34652,34580,34580,34580,34514,27709],"quality":"More is better","isMain":true,"metricName":"Total length (>= 1000 bp)"},{"values":[8101,0,34543,34624,34666,34106,25140,34652,34580,34580,34580,34514,27709],"quality":"More is better","isMain":false,"metricName":"Total length (>= 5000 bp)"},{"values":[0,0,34543,34624,34666,27709,15941,34652,34580,34580,34580,34514,27709],"quality":"More is better","isMain":true,"metricName":"Total length (>= 10000 bp)"},{"values":[0,0,34543,34624,34666,0,0,34652,34580,34580,34580,34514,0],"quality":"More is better","isMain":false,"metricName":"Total length (>= 25000 bp)"},{"values":[0,0,0,0,0,0,0,0,0,0,0,0,0],"quality":"More is better","isMain":true,"metricName":"Total length (>= 50000 bp)"},{"values":[1989,1674,34543,34624,34666,11026,9199,34652,34580,34580,34580,34514,16683],"quality":"More is better","isMain":false,"metricName":"N50"},{"values":[1469,1317,34543,34624,34666,11026,4330,34652,34580,34580,34580,34514,11026],"quality":"More is better","isMain":false,"metricName":"N75"},{"values":[5,6,1,1,1,2,2,1,1,1,1,1,1],"quality":"Less is better","isMain":false,"metricName":"L50"},{"values":[10,11,1,1,1,2,3,1,1,1,1,1,2],"quality":"Less is better","isMain":false,"metricName":"L75"},{"values":["41.43","41.57","41.58","41.56","41.54","41.73","41.59","41.52","41.56","41.57","41.57","41.57","41.81"],"quality":"Equal","isMain":false,"metricName":"GC (%)"}]],["Predicted genes",[]],["Similarity statistics",[{"values":[0,0,0,0,0,0,0,0,0,0,0,0,0],"quality":"Equal","isMain":false,"metricName":"# similar correct contigs"},{"values":[0,0,0,0,0,0,0,0,0,0,0,0,0],"quality":"Equal","isMain":false,"metricName":"# similar misassembled blocks"}]],["Reference statistics",[{"values":[34525,34525,34525,34525,34525,34525,34525,34525,34525,34525,34525,34525,34525],"quality":"Equal","isMain":false,"metricName":"Reference length"},{"values":[1,1,1,1,1,1,1,1,1,1,1,1,1],"quality":"Equal","isMain":false,"metricName":"Reference fragments"},{"values":["41.57","41.57","41.57","41.57","41.57","41.57","41.57","41.57","41.57","41.57","41.57","41.57","41.57"],"quality":"Equal","isMain":false,"metricName":"Reference GC (%)"}]]],"referenceName":"gi\_109289936\_ref\_NC","date":"18 July 2018, Wednesday, 16:05:33","order":[0,1,2,3,4,5,6,7,8,9,10,11,12],"assembliesNames":["ABySS\_127","ABySS\_63","CLC","IDBA\_UD","MEGAHIT","MIRA","SOAPdenovo2","SPAdes","SPAdes\_meta","SPAdes\_sc","SPAdes\_sc\_careful","Velvet","Geneious"]},{"assembliesWithNs":null,"minContig":500,"report":[["Genome statistics",[{"values":["67.039","59.392","92.833","94.784","94.567","83.171","89.604","97.501","96.220","96.797","96.622","67.313","21.707"],"quality":"More is better","isMain":true,"metricName":"Genome fraction (%)"},{"values":["1.001","1.001","1.016","1.002","1.000","1.027","1.017","1.001","1.003","1.003","1.002","1.000","1.000"],"quality":"Less is better","isMain":true,"metricName":"Duplication ratio"},{"values":[3135,3305,9377,13715,13879,6185,7925,13879,13879,13879,13879,12739,7220],"quality":"More is better","isMain":true,"metricName":"Largest alignment"},{"values":[108374,95975,152006,153284,152721,137876,145498,157611,155833,156736,156400,108719,35056],"quality":"More is better","isMain":true,"metricName":"Total aligned length"},{"values":[821,692,4084,7434,5625,2473,2239,6493,5667,6305,6305,2688,null],"quality":"More is better","isMain":false,"metricName":"NG50"},{"values":[null,null,1810,4013,3337,1100,1197,4013,3176,3739,3403,null,null],"quality":"More is better","isMain":false,"metricName":"NG75"},{"values":[1238,1067,4482,5058,5056,2608,2293,5843,5667,5233,5233,4049,3480],"quality":"More is better","isMain":false,"metricName":"NA50"},{"values":[821,761,2081,2400,3166,1510,1418,3176,3461,2402,2402,2322,1708],"quality":"More is better","isMain":false,"metricName":"NA75"},{"values":[821,692,4084,5711,5056,2335,2132,5843,5667,5667,5667,2405,null],"quality":"More is better","isMain":true,"metricName":"NGA50"},{"values":[null,null,1670,2880,3166,1088,1187,3583,3176,2880,2880,null,null],"quality":"More is better","isMain":false,"metricName":"NGA75"},{"values":[54,69,14,7,9,21,21,8,9,9,9,16,null],"quality":"Less is better","isMain":false,"metricName":"LG50"},{"values":[null,null,29,15,18,46,46,15,18,17,17,null,null],"quality":"Less is better","isMain":false,"metricName":"LG75"},{"values":[26,29,13,10,10,18,18,9,9,10,10,9,5],"quality":"Less is better","isMain":false,"metricName":"LA50"},{"values":[55,57,26,21,20,35,38,19,17,21,21,19,9],"quality":"Less is better","isMain":false,"metricName":"LA75"},{"values":[54,69,14,9,10,22,21,9,9,9,9,17,null],"quality":"Less is better","isMain":true,"metricName":"LGA50"},{"values":[null,null,30,19,20,48,46,17,18,19,19,null,null],"quality":"Less is better","isMain":false,"metricName":"LGA75"}]],["Misassemblies",[{"values":[0,0,0,0,0,0,0,0,0,0,0,0,0],"quality":"Less is better","isMain":true,"metricName":"# misassemblies"},{"values":[0,0,0,0,0,0,0,0,0,0,0,0,0],"quality":"Less is better","isMain":false,"metricName":" # relocations"},{"values":[0,0,0,0,0,0,0,0,0,0,0,0,0],"quality":"Less is better","isMain":false,"metricName":" # translocations"},{"values":[0,0,0,0,0,0,0,0,0,0,0,0,0],"quality":"Less is better","isMain":false,"metricName":" # inversions"},{"values":[0,0,0,0,0,0,0,0,0,0,0,0,0],"quality":"Less is better","isMain":false,"metricName":"# misassembled contigs"},{"values":[0,0,0,0,0,0,0,0,0,0,0,0,0],"quality":"Less is better","isMain":true,"metricName":"Misassembled contigs length"},{"values":[0,0,0,0,0,0,0,0,0,0,0,0,0],"quality":"Less is better","isMain":false,"metricName":"# local misassemblies"},{"values":[0,0,0,0,0,0,0,0,0,0,0,0,0],"quality":"Less is better","isMain":false,"metricName":"# unaligned mis. contigs"}]],["Unaligned",[{"values":[0,0,0,0,0,0,0,0,0,0,0,0,0],"quality":"Less is better","isMain":false,"metricName":"# fully unaligned contigs"},{"values":[0,0,0,0,0,0,0,0,0,0,0,0,0],"quality":"Less is better","isMain":false,"metricName":"Fully unaligned length"},{"values":[0,0,0,2,1,1,0,2,0,1,1,2,3],"quality":"Less is better","isMain":false,"metricName":"# partially unaligned contigs"},{"values":[0,0,0,15607,6147,2697,0,8329,0,9833,9833,3391,10451],"quality":"Less is better","isMain":false,"metricName":"Partially unaligned length"}]],["Mismatches",[{"values":[15,6,99,57,50,24,47,102,133,77,69,57,31],"quality":"Less is better","isMain":false,"metricName":"# mismatches"},{"values":[0,0,1,1,1,1,22,1,0,0,0,1,9],"quality":"Less is better","isMain":false,"metricName":"# indels"},{"values":[0,0,1,1,1,1,1030,1,0,0,0,1,11],"quality":"Less is better","isMain":false,"metricName":"Indels length"},{"values":["13.86","6.26","66.04","37.24","32.74","17.87","32.48","64.79","85.60","49.26","44.22","52.44","88.44"],"quality":"Less is better","isMain":true,"metricName":"# mismatches per 100 kbp"},{"values":["0.00","0.00","0.67","0.65","0.65","0.74","15.21","0.64","0.00","0.00","0.00","0.92","25.68"],"quality":"Less is better","isMain":true,"metricName":"# indels per 100 kbp"},{"values":[0,0,1,1,1,1,0,1,0,0,0,1,9],"quality":"Less is better","isMain":false,"metricName":" # indels (<= 5 bp)"},{"values":[0,0,0,0,0,0,22,0,0,0,0,0,0],"quality":"Less is better","isMain":false,"metricName":" # indels (> 5 bp)"},{"values":[0,0,0,0,0,92,2148,0,0,0,112,0,335],"quality":"Less is better","isMain":false,"metricName":"# N's"},{"values":["0.00","0.00","0.00","0.00","0.00","65.45","1460.13","0.00","0.00","0.00","67.37","0.00","736.15"],"quality":"Less is better","isMain":true,"metricName":"# N's per 100 kbp"}]],["Statistics without reference",[{"values":[94,94,61,38,37,70,75,35,38,40,40,36,9],"quality":"Equal","isMain":true,"metricName":"# contigs"},{"values":[37,31,41,35,35,49,55,34,36,38,38,32,9],"quality":"Equal","isMain":false,"metricName":"# contigs (>= 1000 bp)"},{"values":[0,0,8,12,10,4,5,11,9,11,11,7,3],"quality":"Equal","isMain":false,"metricName":"# contigs (>= 5000 bp)"},{"values":[0,0,0,4,4,0,0,4,4,5,5,1,0],"quality":"Equal","isMain":false,"metricName":"# contigs (>= 10000 bp)"},{"values":[0,0,0,0,0,0,0,0,0,0,0,0,0],"quality":"Equal","isMain":false,"metricName":"# contigs (>= 25000 bp)"},{"values":[0,0,0,0,0,0,0,0,0,0,0,0,0],"quality":"Equal","isMain":false,"metricName":"# contigs (>= 50000 bp)"},{"values":[3135,3305,9377,19021,13879,6185,7925,19179,13879,13879,13879,12739,9897],"quality":"More is better","isMain":true,"metricName":"Largest contig"},{"values":[108374,95975,152234,168894,158868,140573,147110,165943,155845,166572,166236,112113,45507],"quality":"More is better","isMain":true,"metricName":"Total length"},{"values":[66032,50066,137226,166642,157383,125021,132066,165147,154527,165086,164750,108984,45507],"quality":"More is better","isMain":true,"metricName":"Total length (>= 1000 bp)"},{"values":[0,0,54484,110104,89022,23758,31857,103787,83553,97833,97833,47954,25851],"quality":"More is better","isMain":false,"metricName":"Total length (>= 5000 bp)"},{"values":[0,0,0,56031,49672,0,0,56716,49367,58414,58414,12739,0],"quality":"More is better","isMain":true,"metricName":"Total length (>= 10000 bp)"},{"values":[0,0,0,0,0,0,0,0,0,0,0,0,0],"quality":"More is better","isMain":false,"metricName":"Total length (>= 25000 bp)"},{"values":[0,0,0,0,0,0,0,0,0,0,0,0,0],"quality":"More is better","isMain":true,"metricName":"Total length (>= 50000 bp)"},{"values":[1238,1067,4482,6344,5625,2683,2293,6493,5667,6305,6305,4843,6107],"quality":"More is better","isMain":false,"metricName":"N50"},{"values":[821,761,2081,3586,3337,1517,1443,3795,3461,3176,3176,2365,4104],"quality":"More is better","isMain":false,"metricName":"N75"},{"values":[26,29,13,8,9,17,18,8,9,9,9,9,3],"quality":"Less is better","isMain":false,"metricName":"L50"},{"values":[55,57,26,16,18,34,37,16,17,18,18,18,5],"quality":"Less is better","isMain":false,"metricName":"L75"},{"values":["40.95","41.00","40.92","41.28","41.15","41.17","40.92","41.20","40.94","41.15","41.14","41.21","41.92"],"quality":"Equal","isMain":false,"metricName":"GC (%)"}]],["Predicted genes",[]],["Similarity statistics",[{"values":[0,0,0,1,1,0,0,1,1,1,1,1,0],"quality":"Equal","isMain":false,"metricName":"# similar correct contigs"},{"values":[0,0,0,0,0,0,0,0,0,0,0,0,0],"quality":"Equal","isMain":false,"metricName":"# similar misassembled blocks"}]],["Reference statistics",[{"values":[161475,161475,161475,161475,161475,161475,161475,161475,161475,161475,161475,161475,161475],"quality":"Equal","isMain":false,"metricName":"Reference length"},{"values":[1,1,1,1,1,1,1,1,1,1,1,1,1],"quality":"Equal","isMain":false,"metricName":"Reference fragments"},{"values":["41.04","41.04","41.04","41.04","41.04","41.04","41.04","41.04","41.04","41.04","41.04","41.04","41.04"],"quality":"Equal","isMain":false,"metricName":"Reference GC (%)"}]]],"referenceName":"gi\_109289986\_ref\_NC","date":"18 July 2018, Wednesday, 16:05:44","order":[0,1,2,3,4,5,6,7,8,9,10,11,12],"assembliesNames":["ABySS\_127","ABySS\_63","CLC","IDBA\_UD","MEGAHIT","MIRA","SOAPdenovo2","SPAdes","SPAdes\_meta","SPAdes\_sc","SPAdes\_sc\_careful","Velvet","Geneious"]},{"assembliesWithNs":null,"minContig":500,"report":[["Genome statistics",[{"values":["9.421","6.474","0.708","4.198","4.792","23.560","2.879","2.405","12.168","2.192","9.473","9.548","4.243","5.123"],"quality":"More is better","isMain":true,"metricName":"Genome fraction (%)"},{"values":["1.000","1.000","1.000","1.000","1.000","1.015","1.000","1.000","1.000","0.999","1.008","1.008","1.000","1.000"],"quality":"Less is better","isMain":true,"metricName":"Duplication ratio"},{"values":[1094,955,527,1069,1003,1938,865,877,5225,882,4314,4314,891,3816],"quality":"More is better","isMain":true,"metricName":"Largest alignment"},{"values":[7017,4822,527,3126,3568,17808,2144,1791,9063,1632,7110,7166,3160,3816],"quality":"More is better","isMain":true,"metricName":"Total aligned length"},{"values":[908,911,527,911,995,1272,665,177,5225,882,4314,4314,794,3816],"quality":"More is better","isMain":false,"metricName":"NA50"},{"values":[638,837,527,593,988,1053,614,null,967,750,823,823,773,3816],"quality":"More is better","isMain":false,"metricName":"NA75"},{"values":[null,null,null,null,null,null,null,null,null,null,null,null,null,null],"quality":"More is better","isMain":true,"metricName":"NGA50"},{"values":[4,3,1,2,2,6,2,3,1,1,1,1,2,1],"quality":"Less is better","isMain":false,"metricName":"LA50"},{"values":[7,4,1,3,3,10,3,null,3,2,3,3,3,1],"quality":"Less is better","isMain":false,"metricName":"LA75"}]],["Misassemblies",[{"values":[0,0,0,0,0,0,0,0,0,0,0,0,0,0],"quality":"Less is better","isMain":true,"metricName":"# misassemblies"},{"values":[0,0,0,0,0,0,0,0,0,0,0,0,0,0],"quality":"Less is better","isMain":false,"metricName":" # relocations"},{"values":[0,0,0,0,0,0,0,0,0,0,0,0,0,0],"quality":"Less is better","isMain":false,"metricName":" # translocations"},{"values":[0,0,0,0,0,0,0,0,0,0,0,0,0,0],"quality":"Less is better","isMain":false,"metricName":" # inversions"},{"values":[0,0,0,0,0,0,0,0,0,0,0,0,0,0],"quality":"Less is better","isMain":false,"metricName":"# misassembled contigs"},{"values":[0,0,0,0,0,0,0,0,0,0,0,0,0,0],"quality":"Less is better","isMain":true,"metricName":"Misassembled contigs length"},{"values":[0,0,0,0,0,0,0,0,0,0,0,0,0,1],"quality":"Less is better","isMain":false,"metricName":"# local misassemblies"},{"values":[0,0,0,0,0,0,0,0,0,0,0,0,0,0],"quality":"Less is better","isMain":false,"metricName":"# unaligned mis. contigs"}]],["Unaligned",[{"values":[0,0,0,0,0,0,0,0,0,0,0,0,0,0],"quality":"Less is better","isMain":false,"metricName":"# fully unaligned contigs"},{"values":[0,0,0,0,0,0,0,0,0,0,0,0,0,0],"quality":"Less is better","isMain":false,"metricName":"Fully unaligned length"},{"values":[0,0,0,0,0,0,0,1,1,0,0,0,0,0],"quality":"Less is better","isMain":false,"metricName":"# partially unaligned contigs"},{"values":[0,0,0,0,0,0,0,1754,593,0,0,0,0,0],"quality":"Less is better","isMain":false,"metricName":"Partially unaligned length"}]],["Mismatches",[{"values":[0,3,2,5,7,39,1,1,24,6,30,30,5,29],"quality":"Less is better","isMain":false,"metricName":"# mismatches"},{"values":[0,0,0,1,1,3,0,0,0,1,3,3,0,6],"quality":"Less is better","isMain":false,"metricName":"# indels"},{"values":[0,0,0,1,1,18,0,0,0,1,3,3,0,8],"quality":"Less is better","isMain":false,"metricName":"Indels length"},{"values":["0.00","62.21","379.51","159.90","196.13","222.25","46.64","55.83","264.81","367.42","425.17","421.82","158.23","759.96"],"quality":"Less is better","isMain":true,"metricName":"# mismatches per 100 kbp"},{"values":["0.00","0.00","0.00","31.98","28.02","17.10","0.00","0.00","0.00","61.24","42.52","42.18","0.00","157.23"],"quality":"Less is better","isMain":true,"metricName":"# indels per 100 kbp"},{"values":[0,0,0,1,1,2,0,0,0,1,3,3,0,6],"quality":"Less is better","isMain":false,"metricName":" # indels (<= 5 bp)"},{"values":[0,0,0,0,0,1,0,0,0,0,0,0,0,0],"quality":"Less is better","isMain":false,"metricName":" # indels (> 5 bp)"},{"values":[0,0,0,0,0,12,0,448,593,0,0,0,0,71],"quality":"Less is better","isMain":false,"metricName":"# N's"},{"values":["0.00","0.00","0.00","0.00","0.00","67.39","0.00","12637.52","6141.26","0.00","0.00","0.00","0.00","1860.59"],"quality":"Less is better","isMain":true,"metricName":"# N's per 100 kbp"}]],["Statistics without reference",[{"values":[9,6,1,4,4,15,3,3,6,2,5,5,4,1],"quality":"Equal","isMain":true,"metricName":"# contigs"},{"values":[2,0,0,1,1,11,0,1,2,0,1,1,0,1],"quality":"Equal","isMain":false,"metricName":"# contigs (>= 1000 bp)"},{"values":[0,0,0,0,0,0,0,0,1,0,0,0,0,0],"quality":"Equal","isMain":false,"metricName":"# contigs (>= 5000 bp)"},{"values":[0,0,0,0,0,0,0,0,0,0,0,0,0,0],"quality":"Equal","isMain":false,"metricName":"# contigs (>= 10000 bp)"},{"values":[0,0,0,0,0,0,0,0,0,0,0,0,0,0],"quality":"Equal","isMain":false,"metricName":"# contigs (>= 25000 bp)"},{"values":[0,0,0,0,0,0,0,0,0,0,0,0,0,0],"quality":"Equal","isMain":false,"metricName":"# contigs (>= 50000 bp)"},{"values":[1094,955,527,1069,1003,1938,865,1931,5818,882,4319,4319,891,3816],"quality":"More is better","isMain":true,"metricName":"Largest contig"},{"values":[7017,4822,527,3126,3568,17808,2144,3545,9656,1632,7115,7171,3160,3816],"quality":"More is better","isMain":true,"metricName":"Total length"},{"values":[2094,0,0,1069,1003,14672,0,1931,6952,0,4319,4319,0,3816],"quality":"More is better","isMain":true,"metricName":"Total length (>= 1000 bp)"},{"values":[0,0,0,0,0,0,0,0,5818,0,0,0,0,0],"quality":"More is better","isMain":false,"metricName":"Total length (>= 5000 bp)"},{"values":[0,0,0,0,0,0,0,0,0,0,0,0,0,0],"quality":"More is better","isMain":true,"metricName":"Total length (>= 10000 bp)"},{"values":[0,0,0,0,0,0,0,0,0,0,0,0,0,0],"quality":"More is better","isMain":false,"metricName":"Total length (>= 25000 bp)"},{"values":[0,0,0,0,0,0,0,0,0,0,0,0,0,0],"quality":"More is better","isMain":true,"metricName":"Total length (>= 50000 bp)"},{"values":[908,911,527,911,995,1272,665,1931,5818,882,4319,4319,794,3816],"quality":"More is better","isMain":false,"metricName":"N50"},{"values":[638,837,527,593,988,1053,614,877,967,750,823,823,773,3816],"quality":"More is better","isMain":false,"metricName":"N75"},{"values":[4,3,1,2,2,6,2,1,1,1,1,1,2,1],"quality":"Less is better","isMain":false,"metricName":"L50"},{"values":[7,4,1,3,3,10,3,2,3,2,3,3,3,1],"quality":"Less is better","isMain":false,"metricName":"L75"},{"values":["63.18","63.36","70.40","63.15","63.09","63.65","65.44","58.22","63.41","63.79","63.61","63.65","61.11","63.68"],"quality":"Equal","isMain":false,"metricName":"GC (%)"}]],["Predicted genes",[]],["Similarity statistics",[{"values":[0,0,0,0,0,0,0,0,0,0,0,0,0,0],"quality":"Equal","isMain":false,"metricName":"# similar correct contigs"},{"values":[0,0,0,0,0,0,0,0,0,0,0,0,0,0],"quality":"Equal","isMain":false,"metricName":"# similar misassembled blocks"}]],["Reference statistics",[{"values":[74483,74483,74483,74483,74483,74483,74483,74483,74483,74483,74483,74483,74483,74483],"quality":"Equal","isMain":false,"metricName":"Reference length"},{"values":[1,1,1,1,1,1,1,1,1,1,1,1,1,1],"quality":"Equal","isMain":false,"metricName":"Reference fragments"},{"values":["62.94","62.94","62.94","62.94","62.94","62.94","62.94","62.94","62.94","62.94","62.94","62.94","62.94","62.94"],"quality":"Equal","isMain":false,"metricName":"Reference GC (%)"}]]],"referenceName":"gi\_109302753\_ref\_NC","date":"18 July 2018, Wednesday, 16:05:55","order":[0,1,2,3,4,5,6,7,8,9,10,11,12,13],"assembliesNames":["ABySS\_127","ABySS\_63","CLC","IDBA\_UD","MEGAHIT","MIRA","Ray\_Meta","SOAPdenovo2","SPAdes","SPAdes\_meta","SPAdes\_sc","SPAdes\_sc\_careful","Velvet","Geneious"]},{"assembliesWithNs":null,"minContig":500,"report":[["Genome statistics",[{"values":["24.314","3.805","21.030","31.193","29.216","67.785","13.298","25.985","17.178","19.720","21.677","11.577","26.381"],"quality":"More is better","isMain":true,"metricName":"Genome fraction (%)"},{"values":["1.005","1.000","1.011","1.011","1.013","1.132","1.135","1.000","1.031","1.005","1.004","1.004","1.001"],"quality":"Less is better","isMain":true,"metricName":"Duplication ratio"},{"values":[1288,954,1277,1429,1772,4100,1200,3101,4049,1221,1194,1484,5259],"quality":"More is better","isMain":true,"metricName":"Largest alignment"},{"values":[13888,2163,12019,17931,16674,42684,7604,14770,9805,11211,12324,6582,15009],"quality":"More is better","isMain":true,"metricName":"Total aligned length"},{"values":[null,null,null,null,null,1703,null,null,null,null,null,null,null],"quality":"More is better","isMain":false,"metricName":"NG50"},{"values":[null,null,null,null,null,1192,null,null,null,null,null,null,null],"quality":"More is better","isMain":false,"metricName":"NG75"},{"values":[726,639,711,912,813,1410,760,1641,566,970,970,1019,5172],"quality":"More is better","isMain":false,"metricName":"NA50"},{"values":[646,570,609,633,591,1018,608,755,null,732,620,719,2058],"quality":"More is better","isMain":false,"metricName":"NA75"},{"values":[null,null,null,null,null,1357,null,null,null,null,null,null,null],"quality":"More is better","isMain":true,"metricName":"NGA50"},{"values":[null,null,null,null,null,339,null,null,null,null,null,null,null],"quality":"More is better","isMain":false,"metricName":"NGA75"},{"values":[null,null,null,null,null,11,null,null,null,null,null,null,null],"quality":"Less is better","isMain":false,"metricName":"LG50"},{"values":[null,null,null,null,null,21,null,null,null,null,null,null,null],"quality":"Less is better","isMain":false,"metricName":"LG75"},{"values":[8,2,7,8,8,13,5,4,6,6,6,3,2],"quality":"Less is better","isMain":false,"metricName":"LA50"},{"values":[13,3,11,14,14,24,9,8,null,9,10,5,4],"quality":"Less is better","isMain":false,"metricName":"LA75"},{"values":[null,null,null,null,null,15,null,null,null,null,null,null,null],"quality":"Less is better","isMain":true,"metricName":"LGA50"},{"values":[null,null,null,null,null,29,null,null,null,null,null,null,null],"quality":"Less is better","isMain":false,"metricName":"LGA75"}]],["Misassemblies",[{"values":[0,0,0,0,0,0,0,0,0,0,0,0,0],"quality":"Less is better","isMain":true,"metricName":"# misassemblies"},{"values":[0,0,0,0,0,0,0,0,0,0,0,0,0],"quality":"Less is better","isMain":false,"metricName":" # relocations"},{"values":[0,0,0,0,0,0,0,0,0,0,0,0,0],"quality":"Less is better","isMain":false,"metricName":" # translocations"},{"values":[0,0,0,0,0,0,0,0,0,0,0,0,0],"quality":"Less is better","isMain":false,"metricName":" # inversions"},{"values":[0,0,0,0,0,0,0,0,0,0,0,0,0],"quality":"Less is better","isMain":false,"metricName":"# misassembled contigs"},{"values":[0,0,0,0,0,0,0,0,0,0,0,0,0],"quality":"Less is better","isMain":true,"metricName":"Misassembled contigs length"},{"values":[0,0,0,0,0,1,0,1,0,0,0,0,2],"quality":"Less is better","isMain":false,"metricName":"# local misassemblies"},{"values":[0,0,0,0,0,0,0,0,1,0,0,0,0],"quality":"Less is better","isMain":false,"metricName":"# unaligned mis. contigs"}]],["Unaligned",[{"values":[0,0,0,0,0,0,0,0,0,0,0,0,0],"quality":"Less is better","isMain":false,"metricName":"# fully unaligned contigs"},{"values":[0,0,0,0,0,0,0,0,0,0,0,0,0],"quality":"Less is better","isMain":false,"metricName":"Fully unaligned length"},{"values":[0,0,0,0,1,4,1,1,1,0,0,0,3],"quality":"Less is better","isMain":false,"metricName":"# partially unaligned contigs"},{"values":[0,0,0,0,937,7488,576,1613,7876,0,0,0,3896],"quality":"Less is better","isMain":false,"metricName":"Partially unaligned length"}]],["Mismatches",[{"values":[9,0,34,8,54,323,0,82,83,12,13,4,159],"quality":"Less is better","isMain":false,"metricName":"# mismatches"},{"values":[0,0,0,0,2,2,1,10,4,0,0,0,10],"quality":"Less is better","isMain":false,"metricName":"# indels"},{"values":[0,0,0,0,2,2,44,17,10,0,0,0,13],"quality":"Less is better","isMain":false,"metricName":"Indels length"},{"values":["65.11","0.00","284.38","45.11","325.11","838.16","0.00","555.07","849.89","107.04","105.49","60.77","1060.14"],"quality":"Less is better","isMain":true,"metricName":"# mismatches per 100 kbp"},{"values":["0.00","0.00","0.00","0.00","12.04","5.19","13.23","67.69","40.96","0.00","0.00","0.00","66.68"],"quality":"Less is better","isMain":true,"metricName":"# indels per 100 kbp"},{"values":[0,0,0,0,2,2,0,9,4,0,0,0,10],"quality":"Less is better","isMain":false,"metricName":" # indels (<= 5 bp)"},{"values":[0,0,0,0,0,0,1,1,0,0,0,0,0],"quality":"Less is better","isMain":false,"metricName":" # indels (> 5 bp)"},{"values":[0,0,0,0,0,65,886,0,0,0,0,0,372],"quality":"Less is better","isMain":false,"metricName":"# N's"},{"values":["0.00","0.00","0.00","0.00","0.00","127.21","9674.60","0.00","0.00","0.00","0.00","0.00","1967.73"],"quality":"Less is better","isMain":true,"metricName":"# N's per 100 kbp"}]],["Statistics without reference",[{"values":[19,3,16,21,20,29,10,12,6,13,15,7,4],"quality":"Equal","isMain":true,"metricName":"# contigs"},{"values":[3,0,3,7,8,27,2,6,4,5,5,3,4],"quality":"Equal","isMain":false,"metricName":"# contigs (>= 1000 bp)"},{"values":[0,0,0,0,0,0,0,0,1,0,0,0,2],"quality":"Equal","isMain":false,"metricName":"# contigs (>= 5000 bp)"},{"values":[0,0,0,0,0,0,0,0,1,0,0,0,0],"quality":"Equal","isMain":false,"metricName":"# contigs (>= 10000 bp)"},{"values":[0,0,0,0,0,0,0,0,0,0,0,0,0],"quality":"Equal","isMain":false,"metricName":"# contigs (>= 25000 bp)"},{"values":[0,0,0,0,0,0,0,0,0,0,0,0,0],"quality":"Equal","isMain":false,"metricName":"# contigs (>= 50000 bp)"},{"values":[1288,954,1298,1429,1772,4100,1515,4714,10052,1275,1248,1484,6718],"quality":"More is better","isMain":true,"metricName":"Largest contig"},{"values":[13888,2163,12091,17931,17765,51097,9158,16383,17940,11265,12378,6611,18905],"quality":"More is better","isMain":true,"metricName":"Total length"},{"values":[3373,0,3474,8285,10058,49374,2737,12417,16491,5599,5572,3701,18905],"quality":"More is better","isMain":true,"metricName":"Total length (>= 1000 bp)"},{"values":[0,0,0,0,0,0,0,0,10052,0,0,0,12699],"quality":"More is better","isMain":false,"metricName":"Total length (>= 5000 bp)"},{"values":[0,0,0,0,0,0,0,0,10052,0,0,0,0],"quality":"More is better","isMain":true,"metricName":"Total length (>= 10000 bp)"},{"values":[0,0,0,0,0,0,0,0,0,0,0,0,0],"quality":"More is better","isMain":false,"metricName":"Total length (>= 25000 bp)"},{"values":[0,0,0,0,0,0,0,0,0,0,0,0,0],"quality":"More is better","isMain":true,"metricName":"Total length (>= 50000 bp)"},{"values":[726,639,765,912,1064,1834,935,1671,10052,970,970,1019,5981],"quality":"More is better","isMain":false,"metricName":"N50"},{"values":[646,570,609,633,623,1357,760,1054,4049,732,620,719,4148],"quality":"More is better","isMain":false,"metricName":"N75"},{"values":[8,2,6,8,7,10,4,3,1,6,6,3,2],"quality":"Less is better","isMain":false,"metricName":"L50"},{"values":[13,3,11,14,13,18,7,6,2,9,10,5,3],"quality":"Less is better","isMain":false,"metricName":"L75"},{"values":["60.60","61.40","61.20","61.01","61.18","61.63","61.00","61.16","61.32","61.43","61.43","61.90","61.84"],"quality":"Equal","isMain":false,"metricName":"GC (%)"}]],["Predicted genes",[]],["Similarity statistics",[{"values":[0,0,0,0,0,0,0,0,0,0,0,0,0],"quality":"Equal","isMain":false,"metricName":"# similar correct contigs"},{"values":[0,0,0,0,0,0,0,0,0,0,0,0,0],"quality":"Equal","isMain":false,"metricName":"# similar misassembled blocks"}]],["Reference statistics",[{"values":[56852,56852,56852,56852,56852,56852,56852,56852,56852,56852,56852,56852,56852],"quality":"Equal","isMain":false,"metricName":"Reference length"},{"values":[1,1,1,1,1,1,1,1,1,1,1,1,1],"quality":"Equal","isMain":false,"metricName":"Reference fragments"},{"values":["61.53","61.53","61.53","61.53","61.53","61.53","61.53","61.53","61.53","61.53","61.53","61.53","61.53"],"quality":"Equal","isMain":false,"metricName":"Reference GC (%)"}]]],"referenceName":"gi\_109392186\_ref\_NC","date":"18 July 2018, Wednesday, 16:06:05","order":[0,1,2,3,4,5,6,7,8,9,10,11,12],"assembliesNames":["ABySS\_127","ABySS\_63","CLC","IDBA\_UD","MEGAHIT","MIRA","SOAPdenovo2","SPAdes","SPAdes\_meta","SPAdes\_sc","SPAdes\_sc\_careful","Velvet","Geneious"]},{"assembliesWithNs":null,"minContig":500,"report":[["Genome statistics",[{"values":["100.000","93.298","99.604","99.438","95.280","100.000","39.475","11.171","100.000","22.218","88.652","91.882","22.212","100.000"],"quality":"More is better","isMain":true,"metricName":"Genome fraction (%)"},{"values":["1.181","1.012","1.015","1.011","1.011","1.020","1.023","1.196","1.002","1.007","1.001","1.000","1.003","1.004"],"quality":"Less is better","isMain":true,"metricName":"Duplication ratio"},{"values":[67655,9532,12657,17344,11959,70925,1609,566,70781,11333,30166,30684,2168,70925],"quality":"More is better","isMain":true,"metricName":"Largest alignment"},{"values":[83474,66736,71408,70996,68049,72090,28529,7893,70781,15710,62638,64918,15736,70925],"quality":"More is better","isMain":true,"metricName":"Total aligned length"},{"values":[83474,4532,11699,8740,8013,70925,null,null,70781,null,11212,30059,null,70925],"quality":"More is better","isMain":false,"metricName":"NG50"},{"values":[83474,1778,9347,4792,2023,70925,null,null,70781,null,3606,30059,null,70925],"quality":"More is better","isMain":false,"metricName":"NG75"},{"values":[67655,4920,11699,8740,8013,70925,781,null,70781,11333,11212,30059,758,70925],"quality":"More is better","isMain":false,"metricName":"NA50"},{"values":[67655,1782,9347,4792,2061,70925,640,null,70781,2735,6122,30059,593,70925],"quality":"More is better","isMain":false,"metricName":"NA75"},{"values":[67655,4532,11699,8740,8013,70925,null,null,70781,null,11212,30059,null,70925],"quality":"More is better","isMain":true,"metricName":"NGA50"},{"values":[67655,1778,9347,4792,2023,70925,null,null,70781,null,3606,30059,null,70925],"quality":"More is better","isMain":false,"metricName":"NGA75"},{"values":[1,6,3,3,4,1,null,null,1,null,2,2,null,1],"quality":"Less is better","isMain":false,"metricName":"LG50"},{"values":[1,13,5,7,11,1,null,null,1,null,5,2,null,1],"quality":"Less is better","isMain":false,"metricName":"LG75"},{"values":[1,5,3,3,4,1,12,null,1,1,2,2,8,1],"quality":"Less is better","isMain":false,"metricName":"LA50"},{"values":[1,12,5,7,10,1,22,null,1,2,3,2,14,1],"quality":"Less is better","isMain":false,"metricName":"LA75"},{"values":[1,6,3,3,4,1,null,null,1,null,2,2,null,1],"quality":"Less is better","isMain":true,"metricName":"LGA50"},{"values":[1,13,5,7,11,1,null,null,1,null,5,2,null,1],"quality":"Less is better","isMain":false,"metricName":"LGA75"}]],["Misassemblies",[{"values":[1,0,0,0,0,0,0,0,0,0,0,0,0,0],"quality":"Less is better","isMain":true,"metricName":"# misassemblies"},{"values":[1,0,0,0,0,0,0,0,0,0,0,0,0,0],"quality":"Less is better","isMain":false,"metricName":" # relocations"},{"values":[0,0,0,0,0,0,0,0,0,0,0,0,0,0],"quality":"Less is better","isMain":false,"metricName":" # translocations"},{"values":[0,0,0,0,0,0,0,0,0,0,0,0,0,0],"quality":"Less is better","isMain":false,"metricName":" # inversions"},{"values":[1,0,0,0,0,0,0,0,0,0,0,0,0,0],"quality":"Less is better","isMain":false,"metricName":"# misassembled contigs"},{"values":[83474,0,0,0,0,0,0,0,0,0,0,0,0,0],"quality":"Less is better","isMain":true,"metricName":"Misassembled contigs length"},{"values":[0,0,0,0,0,1,0,0,1,1,0,0,0,1],"quality":"Less is better","isMain":false,"metricName":"# local misassemblies"},{"values":[0,0,0,0,0,0,0,8,0,0,0,0,0,0],"quality":"Less is better","isMain":false,"metricName":"# unaligned mis. contigs"}]],["Unaligned",[{"values":[0,0,0,0,0,0,0,0,0,0,0,0,0,0],"quality":"Less is better","isMain":false,"metricName":"# fully unaligned contigs"},{"values":[0,0,0,0,0,0,0,0,0,0,0,0,0,0],"quality":"Less is better","isMain":false,"metricName":"Fully unaligned length"},{"values":[0,0,0,0,0,0,0,10,0,1,0,0,0,0],"quality":"Less is better","isMain":false,"metricName":"# partially unaligned contigs"},{"values":[0,0,0,0,0,0,0,13384,0,2236,0,0,0,0],"quality":"Less is better","isMain":false,"metricName":"Partially unaligned length"}]],["Mismatches",[{"values":[0,400,140,0,14,3,1,3,21,113,216,0,0,0],"quality":"Less is better","isMain":false,"metricName":"# mismatches"},{"values":[0,10,3,0,0,0,0,4,0,15,10,0,0,0],"quality":"Less is better","isMain":false,"metricName":"# indels"},{"values":[0,14,3,0,0,0,0,193,0,23,14,0,0,0],"quality":"Less is better","isMain":false,"metricName":"Indels length"},{"values":["0.00","606.81","198.94","0.00","20.80","4.25","3.59","38.01","29.72","719.84","344.85","0.00","0.00","0.00"],"quality":"Less is better","isMain":true,"metricName":"# mismatches per 100 kbp"},{"values":["0.00","15.17","4.26","0.00","0.00","0.00","0.00","50.68","0.00","95.55","15.97","0.00","0.00","0.00"],"quality":"Less is better","isMain":true,"metricName":"# indels per 100 kbp"},{"values":[0,10,3,0,0,0,0,0,0,15,10,0,0,0],"quality":"Less is better","isMain":false,"metricName":" # indels (<= 5 bp)"},{"values":[0,0,0,0,0,0,0,4,0,0,0,0,0,0],"quality":"Less is better","isMain":false,"metricName":" # indels (> 5 bp)"},{"values":[0,5,0,0,0,0,0,7800,0,104,0,0,0,3],"quality":"Less is better","isMain":false,"metricName":"# N's"},{"values":["0.00","7.49","0.00","0.00","0.00","0.00","0.00","34170.06","0.00","576.18","0.00","0.00","0.00","4.23"],"quality":"Less is better","isMain":true,"metricName":"# N's per 100 kbp"}]],["Statistics without reference",[{"values":[1,27,11,13,23,3,33,18,1,3,9,3,21,1],"quality":"Equal","isMain":true,"metricName":"# contigs"},{"values":[1,18,11,12,18,1,10,10,1,3,9,3,1,1],"quality":"Equal","isMain":false,"metricName":"# contigs (>= 1000 bp)"},{"values":[1,4,6,5,4,1,0,0,1,1,3,2,0,1],"quality":"Equal","isMain":false,"metricName":"# contigs (>= 5000 bp)"},{"values":[1,0,3,2,1,1,0,0,1,1,2,2,0,1],"quality":"Equal","isMain":false,"metricName":"# contigs (>= 10000 bp)"},{"values":[1,0,0,0,0,1,0,0,1,0,1,2,0,1],"quality":"Equal","isMain":false,"metricName":"# contigs (>= 25000 bp)"},{"values":[1,0,0,0,0,1,0,0,1,0,0,0,0,1],"quality":"Equal","isMain":false,"metricName":"# contigs (>= 50000 bp)"},{"values":[83474,9532,12657,17344,11959,70925,1609,3173,70781,13569,30166,30684,2168,70925],"quality":"More is better","isMain":true,"metricName":"Largest contig"},{"values":[83474,66736,71408,70996,68049,72090,28536,22827,70781,18050,62672,64918,15736,70925],"quality":"More is better","isMain":true,"metricName":"Total length"},{"values":[83474,60127,71408,70146,64515,70925,13275,18385,70781,18050,62672,64918,2168,70925],"quality":"More is better","isMain":true,"metricName":"Total length (>= 1000 bp)"},{"values":[83474,28451,61992,47156,36176,70925,0,0,70781,13569,47508,60743,0,70925],"quality":"More is better","isMain":false,"metricName":"Total length (>= 5000 bp)"},{"values":[83474,0,36905,27887,11959,70925,0,0,70781,13569,41378,60743,0,70925],"quality":"More is better","isMain":true,"metricName":"Total length (>= 10000 bp)"},{"values":[83474,0,0,0,0,70925,0,0,70781,0,30166,60743,0,70925],"quality":"More is better","isMain":false,"metricName":"Total length (>= 25000 bp)"},{"values":[83474,0,0,0,0,70925,0,0,70781,0,0,0,0,70925],"quality":"More is better","isMain":true,"metricName":"Total length (>= 50000 bp)"},{"values":[83474,4920,11699,8740,8013,70925,781,1601,70781,13569,11212,30059,758,70925],"quality":"More is better","isMain":false,"metricName":"N50"},{"values":[83474,1782,9347,4792,2061,70925,640,1405,70781,13569,6130,30059,593,70925],"quality":"More is better","isMain":false,"metricName":"N75"},{"values":[1,5,3,3,4,1,12,6,1,1,2,2,8,1],"quality":"Less is better","isMain":false,"metricName":"L50"},{"values":[1,12,5,7,10,1,22,9,1,1,3,2,14,1],"quality":"Less is better","isMain":false,"metricName":"L75"},{"values":["69.06","69.05","69.09","69.10","69.10","69.10","69.12","69.90","69.10","69.56","69.22","69.13","69.27","69.09"],"quality":"Equal","isMain":false,"metricName":"GC (%)"}]],["Predicted genes",[]],["Similarity statistics",[{"values":[0,0,0,0,0,0,0,0,0,0,0,0,0,0],"quality":"Equal","isMain":false,"metricName":"# similar correct contigs"},{"values":[0,0,0,0,0,0,0,0,0,0,0,0,0,0],"quality":"Equal","isMain":false,"metricName":"# similar misassembled blocks"}]],["Reference statistics",[{"values":[70654,70654,70654,70654,70654,70654,70654,70654,70654,70654,70654,70654,70654,70654],"quality":"Equal","isMain":false,"metricName":"Reference length"},{"values":[1,1,1,1,1,1,1,1,1,1,1,1,1,1],"quality":"Equal","isMain":false,"metricName":"Reference fragments"},{"values":["69.11","69.11","69.11","69.11","69.11","69.11","69.11","69.11","69.11","69.11","69.11","69.11","69.11","69.11"],"quality":"Equal","isMain":false,"metricName":"Reference GC (%)"}]]],"referenceName":"gi\_109392353\_ref\_NC","date":"18 July 2018, Wednesday, 16:06:16","order":[0,1,2,3,4,5,6,7,8,9,10,11,12,13],"assembliesNames":["ABySS\_127","ABySS\_63","CLC","IDBA\_UD","MEGAHIT","MIRA","Ray\_Meta","SOAPdenovo2","SPAdes","SPAdes\_meta","SPAdes\_sc","SPAdes\_sc\_careful","Velvet","Geneious"]},{"assembliesWithNs":null,"minContig":500,"report":[["Genome statistics",[{"values":["98.286","100.000","100.000","99.871","100.000","94.503","7.334","100.000","99.318","99.303","98.611","63.216","100.000"],"quality":"More is better","isMain":true,"metricName":"Genome fraction (%)"},{"values":["1.000","1.011","1.006","1.000","1.023","1.058","1.000","1.002","1.005","1.002","1.000","1.008","1.006"],"quality":"Less is better","isMain":true,"metricName":"Duplication ratio"},{"values":[39636,29441,47448,51980,52342,16781,801,52174,51745,40665,40860,3212,52362],"quality":"More is better","isMain":true,"metricName":"Largest alignment"},{"values":[51155,52636,52344,51980,53242,51553,3817,52174,51745,51794,51324,33139,52362],"quality":"More is better","isMain":true,"metricName":"Total aligned length"},{"values":[39636,29441,47448,51980,52342,8017,null,52174,51942,40665,40860,772,52364],"quality":"More is better","isMain":false,"metricName":"NG50"},{"values":[39636,12158,47448,51980,52342,3644,null,52174,51942,40665,40860,null,52364],"quality":"More is better","isMain":false,"metricName":"NG75"},{"values":[39636,29441,47448,51980,52342,8017,627,52174,51745,40665,40860,1107,52362],"quality":"More is better","isMain":false,"metricName":"NA50"},{"values":[39636,12158,47448,51980,52342,3165,591,52174,51745,40665,40860,776,52362],"quality":"More is better","isMain":false,"metricName":"NA75"},{"values":[39636,29441,47448,51980,52342,8017,null,52174,51745,40665,40860,772,52362],"quality":"More is better","isMain":true,"metricName":"NGA50"},{"values":[39636,12158,47448,51980,52342,3165,null,52174,51745,40665,40860,null,52362],"quality":"More is better","isMain":false,"metricName":"NGA75"},{"values":[1,1,1,1,1,3,null,1,1,1,1,20,1],"quality":"Less is better","isMain":false,"metricName":"LG50"},{"values":[1,2,1,1,1,5,null,1,1,1,1,null,1],"quality":"Less is better","isMain":false,"metricName":"LG75"},{"values":[1,1,1,1,1,3,3,1,1,1,1,10,1],"quality":"Less is better","isMain":false,"metricName":"LA50"},{"values":[1,2,1,1,1,5,5,1,1,1,1,19,1],"quality":"Less is better","isMain":false,"metricName":"LA75"},{"values":[1,1,1,1,1,3,null,1,1,1,1,20,1],"quality":"Less is better","isMain":true,"metricName":"LGA50"},{"values":[1,2,1,1,1,5,null,1,1,1,1,null,1],"quality":"Less is better","isMain":false,"metricName":"LGA75"}]],["Misassemblies",[{"values":[0,0,0,0,0,0,0,0,0,0,0,0,0],"quality":"Less is better","isMain":true,"metricName":"# misassemblies"},{"values":[0,0,0,0,0,0,0,0,0,0,0,0,0],"quality":"Less is better","isMain":false,"metricName":" # relocations"},{"values":[0,0,0,0,0,0,0,0,0,0,0,0,0],"quality":"Less is better","isMain":false,"metricName":" # translocations"},{"values":[0,0,0,0,0,0,0,0,0,0,0,0,0],"quality":"Less is better","isMain":false,"metricName":" # inversions"},{"values":[0,0,0,0,0,0,0,0,0,0,0,0,0],"quality":"Less is better","isMain":false,"metricName":"# misassembled contigs"},{"values":[0,0,0,0,0,0,0,0,0,0,0,0,0],"quality":"Less is better","isMain":true,"metricName":"Misassembled contigs length"},{"values":[0,0,0,0,1,0,0,1,1,0,0,0,1],"quality":"Less is better","isMain":false,"metricName":"# local misassemblies"},{"values":[0,0,0,0,0,0,0,0,0,0,0,0,0],"quality":"Less is better","isMain":false,"metricName":"# unaligned mis. contigs"}]],["Unaligned",[{"values":[0,0,0,0,0,0,0,0,0,0,0,0,0],"quality":"Less is better","isMain":false,"metricName":"# fully unaligned contigs"},{"values":[0,0,0,0,0,0,0,0,0,0,0,0,0],"quality":"Less is better","isMain":false,"metricName":"Fully unaligned length"},{"values":[0,0,0,0,0,0,0,0,0,0,0,0,0],"quality":"Less is better","isMain":false,"metricName":"# partially unaligned contigs"},{"values":[0,0,0,0,0,0,0,0,0,0,0,0,0],"quality":"Less is better","isMain":false,"metricName":"Partially unaligned length"}]],["Mismatches",[{"values":[31,0,2,1,1,4,0,0,190,31,0,1,0],"quality":"Less is better","isMain":false,"metricName":"# mismatches"},{"values":[0,0,0,0,0,0,0,0,9,0,0,0,1],"quality":"Less is better","isMain":false,"metricName":"# indels"},{"values":[0,0,0,0,0,0,0,0,10,0,0,0,1],"quality":"Less is better","isMain":false,"metricName":"Indels length"},{"values":["60.60","0.00","3.84","1.92","1.92","8.13","0.00","0.00","367.56","59.98","0.00","3.04","0.00"],"quality":"Less is better","isMain":true,"metricName":"# mismatches per 100 kbp"},{"values":["0.00","0.00","0.00","0.00","0.00","0.00","0.00","0.00","17.41","0.00","0.00","0.00","1.92"],"quality":"Less is better","isMain":true,"metricName":"# indels per 100 kbp"},{"values":[0,0,0,0,0,0,0,0,9,0,0,0,1],"quality":"Less is better","isMain":false,"metricName":" # indels (<= 5 bp)"},{"values":[0,0,0,0,0,0,0,0,0,0,0,0,0],"quality":"Less is better","isMain":false,"metricName":" # indels (> 5 bp)"},{"values":[0,0,0,0,2,0,0,0,0,0,0,0,446],"quality":"Less is better","isMain":false,"metricName":"# N's"},{"values":["0.00","0.00","0.00","0.00","3.76","0.00","0.00","0.00","0.00","0.00","0.00","0.00","851.73"],"quality":"Less is better","isMain":true,"metricName":"# N's per 100 kbp"}]],["Statistics without reference",[{"values":[3,4,3,1,2,11,6,1,1,3,2,31,1],"quality":"Equal","isMain":true,"metricName":"# contigs"},{"values":[3,3,2,1,1,10,0,1,1,2,2,12,1],"quality":"Equal","isMain":false,"metricName":"# contigs (>= 1000 bp)"},{"values":[2,3,1,1,1,3,0,1,1,2,2,0,1],"quality":"Equal","isMain":false,"metricName":"# contigs (>= 5000 bp)"},{"values":[2,3,1,1,1,1,0,1,1,2,2,0,1],"quality":"Equal","isMain":false,"metricName":"# contigs (>= 10000 bp)"},{"values":[1,1,1,1,1,0,0,1,1,1,1,0,1],"quality":"Equal","isMain":false,"metricName":"# contigs (>= 25000 bp)"},{"values":[0,0,0,1,1,0,0,1,1,0,0,0,1],"quality":"Equal","isMain":false,"metricName":"# contigs (>= 50000 bp)"},{"values":[39636,29441,47448,51980,52342,16781,801,52174,51942,40665,40860,3212,52364],"quality":"More is better","isMain":true,"metricName":"Largest contig"},{"values":[51155,52636,52344,51980,53242,52032,3817,52174,51942,51794,51324,33169,52364],"quality":"More is better","isMain":true,"metricName":"Total length"},{"values":[51155,52113,51696,51980,52342,51145,0,52174,51942,51129,51324,19333,52364],"quality":"More is better","isMain":true,"metricName":"Total length (>= 1000 bp)"},{"values":[49816,52113,47448,51980,52342,34023,0,52174,51942,51129,51324,0,52364],"quality":"More is better","isMain":false,"metricName":"Total length (>= 5000 bp)"},{"values":[49816,52113,47448,51980,52342,16781,0,52174,51942,51129,51324,0,52364],"quality":"More is better","isMain":true,"metricName":"Total length (>= 10000 bp)"},{"values":[39636,29441,47448,51980,52342,0,0,52174,51942,40665,40860,0,52364],"quality":"More is better","isMain":false,"metricName":"Total length (>= 25000 bp)"},{"values":[0,0,0,51980,52342,0,0,52174,51942,0,0,0,52364],"quality":"More is better","isMain":true,"metricName":"Total length (>= 50000 bp)"},{"values":[39636,29441,47448,51980,52342,8017,627,52174,51942,40665,40860,1107,52364],"quality":"More is better","isMain":false,"metricName":"N50"},{"values":[39636,12158,47448,51980,52342,3644,591,52174,51942,40665,40860,776,52364],"quality":"More is better","isMain":false,"metricName":"N75"},{"values":[1,1,1,1,1,3,3,1,1,1,1,10,1],"quality":"Less is better","isMain":false,"metricName":"L50"},{"values":[1,2,1,1,1,5,5,1,1,1,1,19,1],"quality":"Less is better","isMain":false,"metricName":"L75"},{"values":["62.88","62.84","62.88","62.88","62.85","62.78","63.82","62.88","62.92","62.89","62.88","62.80","62.86"],"quality":"Equal","isMain":false,"metricName":"GC (%)"}]],["Predicted genes",[]],["Similarity statistics",[{"values":[0,0,0,0,0,0,0,0,0,0,0,0,0],"quality":"Equal","isMain":false,"metricName":"# similar correct contigs"},{"values":[0,0,0,0,0,0,0,0,0,0,0,0,0],"quality":"Equal","isMain":false,"metricName":"# similar misassembled blocks"}]],["Reference statistics",[{"values":[52047,52047,52047,52047,52047,52047,52047,52047,52047,52047,52047,52047,52047],"quality":"Equal","isMain":false,"metricName":"Reference length"},{"values":[1,1,1,1,1,1,1,1,1,1,1,1,1],"quality":"Equal","isMain":false,"metricName":"Reference fragments"},{"values":["62.88","62.88","62.88","62.88","62.88","62.88","62.88","62.88","62.88","62.88","62.88","62.88","62.88"],"quality":"Equal","isMain":false,"metricName":"Reference GC (%)"}]]],"referenceName":"gi\_109392453\_ref\_NC","date":"18 July 2018, Wednesday, 16:06:26","order":[0,1,2,3,4,5,6,7,8,9,10,11,12],"assembliesNames":["ABySS\_63","CLC","IDBA\_UD","MEGAHIT","MIRA","Ray\_Meta","SOAPdenovo2","SPAdes","SPAdes\_meta","SPAdes\_sc","SPAdes\_sc\_careful","Velvet","Geneious"]},{"assembliesWithNs":null,"minContig":500,"report":[["Genome statistics",[{"values":["11.624","4.173","9.822","8.069","14.425","32.209","3.612","4.730","11.724","2.331","12.500","5.592","4.491","9.779"],"quality":"More is better","isMain":true,"metricName":"Genome fraction (%)"},{"values":["1.014","1.006","1.002","1.016","1.043","1.039","1.001","1.112","1.021","1.000","1.037","1.000","1.000","1.000"],"quality":"Less is better","isMain":true,"metricName":"Duplication ratio"},{"values":[1468,948,1944,3369,2668,6575,1052,1012,2778,868,10440,1012,3030,6738],"quality":"More is better","isMain":true,"metricName":"Largest alignment"},{"values":[18125,6454,15124,12607,23126,51466,5554,7273,18408,3584,19495,8598,6906,15036],"quality":"More is better","isMain":true,"metricName":"Total aligned length"},{"values":[798,722,1254,1127,1012,1908,986,543,1353,738,10440,738,824,5846],"quality":"More is better","isMain":false,"metricName":"NA50"},{"values":[612,625,820,672,641,1106,879,387,774,600,639,631,606,5846],"quality":"More is better","isMain":false,"metricName":"NA75"},{"values":[null,null,null,null,null,null,null,null,null,null,null,null,null,null],"quality":"More is better","isMain":true,"metricName":"NGA50"},{"values":[9,4,5,4,7,8,3,7,6,3,1,5,2,2],"quality":"Less is better","isMain":false,"metricName":"LA50"},{"values":[15,7,9,7,13,17,5,11,10,4,7,9,5,2],"quality":"Less is better","isMain":false,"metricName":"LA75"}]],["Misassemblies",[{"values":[0,0,0,0,0,0,0,0,0,0,0,0,0,0],"quality":"Less is better","isMain":true,"metricName":"# misassemblies"},{"values":[0,0,0,0,0,0,0,0,0,0,0,0,0,0],"quality":"Less is better","isMain":false,"metricName":" # relocations"},{"values":[0,0,0,0,0,0,0,0,0,0,0,0,0,0],"quality":"Less is better","isMain":false,"metricName":" # translocations"},{"values":[0,0,0,0,0,0,0,0,0,0,0,0,0,0],"quality":"Less is better","isMain":false,"metricName":" # inversions"},{"values":[0,0,0,0,0,0,0,0,0,0,0,0,0,0],"quality":"Less is better","isMain":false,"metricName":"# misassembled contigs"},{"values":[0,0,0,0,0,0,0,0,0,0,0,0,0,0],"quality":"Less is better","isMain":true,"metricName":"Misassembled contigs length"},{"values":[0,0,0,0,0,0,0,0,0,0,0,0,0,0],"quality":"Less is better","isMain":false,"metricName":"# local misassemblies"},{"values":[0,0,0,0,0,0,0,0,0,0,0,0,0,0],"quality":"Less is better","isMain":false,"metricName":"# unaligned mis. contigs"}]],["Unaligned",[{"values":[0,0,0,0,0,0,0,0,0,0,0,0,0,0],"quality":"Less is better","isMain":false,"metricName":"# fully unaligned contigs"},{"values":[0,0,0,0,0,0,0,0,0,0,0,0,0,0],"quality":"Less is better","isMain":false,"metricName":"Fully unaligned length"},{"values":[0,0,0,0,0,0,0,1,0,0,0,0,0,0],"quality":"Less is better","isMain":false,"metricName":"# partially unaligned contigs"},{"values":[0,0,0,0,0,0,0,654,0,0,0,0,0,0],"quality":"Less is better","isMain":false,"metricName":"Partially unaligned length"}]],["Mismatches",[{"values":[4,1,40,27,63,97,28,0,20,11,95,1,7,52],"quality":"Less is better","isMain":false,"metricName":"# mismatches"},{"values":[0,0,1,1,19,5,0,2,0,0,6,0,0,4],"quality":"Less is better","isMain":false,"metricName":"# indels"},{"values":[0,0,1,1,60,7,0,102,0,0,6,0,0,7],"quality":"Less is better","isMain":false,"metricName":"Indels length"},{"values":["22.38","15.59","264.85","217.60","284.03","195.85","504.14","0.00","110.94","306.92","494.28","11.63","101.36","345.81"],"quality":"Less is better","isMain":true,"metricName":"# mismatches per 100 kbp"},{"values":["0.00","0.00","6.62","8.06","85.66","10.10","0.00","27.50","0.00","0.00","31.22","0.00","0.00","26.60"],"quality":"Less is better","isMain":true,"metricName":"# indels per 100 kbp"},{"values":[0,0,1,1,17,5,0,0,0,0,6,0,0,4],"quality":"Less is better","isMain":false,"metricName":" # indels (<= 5 bp)"},{"values":[0,0,0,0,2,0,0,2,0,0,0,0,0,0],"quality":"Less is better","isMain":false,"metricName":" # indels (> 5 bp)"},{"values":[0,0,0,0,0,38,0,831,0,0,435,0,0,71],"quality":"Less is better","isMain":false,"metricName":"# N's"},{"values":["0.00","0.00","0.00","0.00","0.00","73.83","0.00","9504.75","0.00","0.00","2182.64","0.00","0.00","472.20"],"quality":"Less is better","isMain":true,"metricName":"# N's per 100 kbp"}]],["Statistics without reference",[{"values":[23,9,14,12,23,33,6,14,17,5,14,12,7,3],"quality":"Equal","isMain":true,"metricName":"# contigs"},{"values":[4,0,8,4,8,22,2,1,8,0,2,1,1,3],"quality":"Equal","isMain":false,"metricName":"# contigs (>= 1000 bp)"},{"values":[0,0,0,0,0,1,0,0,0,0,1,0,0,2],"quality":"Equal","isMain":false,"metricName":"# contigs (>= 5000 bp)"},{"values":[0,0,0,0,0,0,0,0,0,0,1,0,0,0],"quality":"Equal","isMain":false,"metricName":"# contigs (>= 10000 bp)"},{"values":[0,0,0,0,0,0,0,0,0,0,0,0,0,0],"quality":"Equal","isMain":false,"metricName":"# contigs (>= 25000 bp)"},{"values":[0,0,0,0,0,0,0,0,0,0,0,0,0,0],"quality":"Equal","isMain":false,"metricName":"# contigs (>= 50000 bp)"},{"values":[1468,948,1946,3369,2668,6575,1058,1012,2778,868,10875,1012,3030,6738],"quality":"More is better","isMain":true,"metricName":"Largest contig"},{"values":[18125,6454,15126,12607,23126,51467,5560,8743,18408,3584,19930,8598,6906,15036],"quality":"More is better","isMain":true,"metricName":"Total length"},{"values":[5220,0,11346,7259,13523,43904,2093,1012,12758,0,11887,1012,3030,15036],"quality":"More is better","isMain":true,"metricName":"Total length (>= 1000 bp)"},{"values":[0,0,0,0,0,6575,0,0,0,0,10875,0,0,12584],"quality":"More is better","isMain":false,"metricName":"Total length (>= 5000 bp)"},{"values":[0,0,0,0,0,0,0,0,0,0,10875,0,0,0],"quality":"More is better","isMain":true,"metricName":"Total length (>= 10000 bp)"},{"values":[0,0,0,0,0,0,0,0,0,0,0,0,0,0],"quality":"More is better","isMain":false,"metricName":"Total length (>= 25000 bp)"},{"values":[0,0,0,0,0,0,0,0,0,0,0,0,0,0],"quality":"More is better","isMain":true,"metricName":"Total length (>= 50000 bp)"},{"values":[798,722,1254,1127,1012,1908,986,558,1353,738,10875,738,824,5846],"quality":"More is better","isMain":false,"metricName":"N50"},{"values":[612,625,1012,672,641,1106,879,540,774,600,738,631,606,5846],"quality":"More is better","isMain":false,"metricName":"N75"},{"values":[9,4,5,4,7,8,3,6,6,3,1,5,2,2],"quality":"Less is better","isMain":false,"metricName":"L50"},{"values":[15,7,8,7,13,17,5,10,10,4,6,9,5,2],"quality":"Less is better","isMain":false,"metricName":"L75"},{"values":["64.88","63.99","64.86","64.67","65.21","64.80","64.68","64.83","64.97","62.64","64.57","64.48","65.90","66.52"],"quality":"Equal","isMain":false,"metricName":"GC (%)"}]],["Predicted genes",[]],["Similarity statistics",[{"values":[0,0,0,0,0,0,0,0,0,0,0,0,0,0],"quality":"Equal","isMain":false,"metricName":"# similar correct contigs"},{"values":[0,0,0,0,0,0,0,0,0,0,0,0,0,0],"quality":"Equal","isMain":false,"metricName":"# similar misassembled blocks"}]],["Reference statistics",[{"values":[153766,153766,153766,153766,153766,153766,153766,153766,153766,153766,153766,153766,153766,153766],"quality":"Equal","isMain":false,"metricName":"Reference length"},{"values":[1,1,1,1,1,1,1,1,1,1,1,1,1,1],"quality":"Equal","isMain":false,"metricName":"Reference fragments"},{"values":["64.74","64.74","64.74","64.74","64.74","64.74","64.74","64.74","64.74","64.74","64.74","64.74","64.74","64.74"],"quality":"Equal","isMain":false,"metricName":"Reference GC (%)"}]]],"referenceName":"gi\_109393216\_ref\_NC","date":"18 July 2018, Wednesday, 16:06:37","order":[0,1,2,3,4,5,6,7,8,9,10,11,12,13],"assembliesNames":["ABySS\_127","ABySS\_63","CLC","IDBA\_UD","MEGAHIT","MIRA","Ray\_Meta","SOAPdenovo2","SPAdes","SPAdes\_meta","SPAdes\_sc","SPAdes\_sc\_careful","Velvet","Geneious"]},{"assembliesWithNs":null,"minContig":500,"report":[["Genome statistics",[{"values":["27.478","20.725","88.287","80.430","80.009","47.841","83.747","87.876","88.590","88.603","88.603","17.384","10.510"],"quality":"More is better","isMain":true,"metricName":"Genome fraction (%)"},{"values":["1.001","1.000","1.001","1.000","1.000","1.008","1.003","1.001","1.001","1.001","1.001","1.002","1.000"],"quality":"Less is better","isMain":true,"metricName":"Duplication ratio"},{"values":[1743,1220,6874,5107,6705,3855,4851,6859,7004,6859,6859,5008,5155],"quality":"More is better","isMain":true,"metricName":"Largest alignment"},{"values":[21554,16257,69354,63108,62760,37825,65789,68989,69549,69559,69559,13636,8244],"quality":"More is better","isMain":true,"metricName":"Total aligned length"},{"values":[null,null,1844,1649,1655,null,1530,1993,1993,1993,1993,null,null],"quality":"More is better","isMain":false,"metricName":"NG50"},{"values":[null,null,926,717,658,null,690,1308,1153,1308,1308,null,null],"quality":"More is better","isMain":false,"metricName":"NG75"},{"values":[849,733,2057,1844,1861,1358,1727,2063,2063,2063,2063,2385,5155],"quality":"More is better","isMain":false,"metricName":"NA50"},{"values":[770,629,1474,1404,1381,974,1012,1602,1602,1602,1602,957,3089],"quality":"More is better","isMain":false,"metricName":"NA75"},{"values":[null,null,1844,1649,1655,null,1530,1993,1993,1993,1993,null,null],"quality":"More is better","isMain":true,"metricName":"NGA50"},{"values":[null,null,926,717,658,null,686,1308,1153,1308,1308,null,null],"quality":"More is better","isMain":false,"metricName":"NGA75"},{"values":[null,null,13,17,15,null,16,13,13,13,13,null,null],"quality":"Less is better","isMain":false,"metricName":"LG50"},{"values":[null,null,26,34,33,null,35,25,25,25,25,null,null],"quality":"Less is better","isMain":false,"metricName":"LG75"},{"values":[9,9,11,13,11,10,13,11,11,11,11,2,1],"quality":"Less is better","isMain":false,"metricName":"LA50"},{"values":[16,15,21,22,21,18,24,20,20,20,20,5,2],"quality":"Less is better","isMain":false,"metricName":"LA75"},{"values":[null,null,13,17,15,null,16,13,13,13,13,null,null],"quality":"Less is better","isMain":true,"metricName":"LGA50"},{"values":[null,null,26,34,33,null,36,25,25,25,25,null,null],"quality":"Less is better","isMain":false,"metricName":"LGA75"}]],["Misassemblies",[{"values":[0,0,0,0,0,0,0,0,0,0,0,0,0],"quality":"Less is better","isMain":true,"metricName":"# misassemblies"},{"values":[0,0,0,0,0,0,0,0,0,0,0,0,0],"quality":"Less is better","isMain":false,"metricName":" # relocations"},{"values":[0,0,0,0,0,0,0,0,0,0,0,0,0],"quality":"Less is better","isMain":false,"metricName":" # translocations"},{"values":[0,0,0,0,0,0,0,0,0,0,0,0,0],"quality":"Less is better","isMain":false,"metricName":" # inversions"},{"values":[0,0,0,0,0,0,0,0,0,0,0,0,0],"quality":"Less is better","isMain":false,"metricName":"# misassembled contigs"},{"values":[0,0,0,0,0,0,0,0,0,0,0,0,0],"quality":"Less is better","isMain":true,"metricName":"Misassembled contigs length"},{"values":[0,0,0,0,1,0,0,1,0,1,1,0,0],"quality":"Less is better","isMain":false,"metricName":"# local misassemblies"},{"values":[0,0,0,0,0,0,0,0,0,0,0,0,0],"quality":"Less is better","isMain":false,"metricName":"# unaligned mis. contigs"}]],["Unaligned",[{"values":[0,0,0,0,0,0,0,0,0,0,0,0,0],"quality":"Less is better","isMain":false,"metricName":"# fully unaligned contigs"},{"values":[0,0,0,0,0,0,0,0,0,0,0,0,0],"quality":"Less is better","isMain":false,"metricName":"Fully unaligned length"},{"values":[0,0,0,0,0,0,0,0,0,0,0,0,0],"quality":"Less is better","isMain":false,"metricName":"# partially unaligned contigs"},{"values":[0,0,0,0,0,0,0,0,0,0,0,0,0],"quality":"Less is better","isMain":false,"metricName":"Partially unaligned length"}]],["Mismatches",[{"values":[2,0,55,33,30,10,42,73,60,52,35,3,0],"quality":"Less is better","isMain":false,"metricName":"# mismatches"},{"values":[0,0,0,0,0,0,1,0,0,0,0,0,0],"quality":"Less is better","isMain":false,"metricName":"# indels"},{"values":[0,0,0,0,0,0,57,0,0,0,0,0,0],"quality":"Less is better","isMain":false,"metricName":"Indels length"},{"values":["9.28","0.00","79.42","52.31","47.80","26.65","63.93","105.90","86.34","74.82","50.36","22.00","0.00"],"quality":"Less is better","isMain":true,"metricName":"# mismatches per 100 kbp"},{"values":["0.00","0.00","0.00","0.00","0.00","0.00","1.52","0.00","0.00","0.00","0.00","0.00","0.00"],"quality":"Less is better","isMain":true,"metricName":"# indels per 100 kbp"},{"values":[0,0,0,0,0,0,0,0,0,0,0,0,0],"quality":"Less is better","isMain":false,"metricName":" # indels (<= 5 bp)"},{"values":[0,0,0,0,0,0,1,0,0,0,0,0,0],"quality":"Less is better","isMain":false,"metricName":" # indels (> 5 bp)"},{"values":[0,0,0,0,0,42,107,0,0,0,0,0,4],"quality":"Less is better","isMain":false,"metricName":"# N's"},{"values":["0.00","0.00","0.00","0.00","0.00","111.04","162.32","0.00","0.00","0.00","0.00","0.00","48.52"],"quality":"Less is better","isMain":true,"metricName":"# N's per 100 kbp"}]],["Statistics without reference",[{"values":[24,22,40,40,39,30,47,37,38,38,38,9,2],"quality":"Equal","isMain":true,"metricName":"# contigs"},{"values":[7,3,25,27,26,17,24,26,26,26,26,4,2],"quality":"Equal","isMain":false,"metricName":"# contigs (>= 1000 bp)"},{"values":[0,0,2,1,1,0,0,2,2,2,2,1,1],"quality":"Equal","isMain":false,"metricName":"# contigs (>= 5000 bp)"},{"values":[0,0,0,0,0,0,0,0,0,0,0,0,0],"quality":"Equal","isMain":false,"metricName":"# contigs (>= 10000 bp)"},{"values":[0,0,0,0,0,0,0,0,0,0,0,0,0],"quality":"Equal","isMain":false,"metricName":"# contigs (>= 25000 bp)"},{"values":[0,0,0,0,0,0,0,0,0,0,0,0,0],"quality":"Equal","isMain":false,"metricName":"# contigs (>= 50000 bp)"},{"values":[1743,1220,6874,5107,6705,3855,4874,6859,7004,6859,6859,5038,5155],"quality":"More is better","isMain":true,"metricName":"Largest contig"},{"values":[21583,16257,69354,63108,62760,37825,65919,68989,69549,69559,69559,13666,8244],"quality":"More is better","isMain":true,"metricName":"Total length"},{"values":[9302,3325,58209,53401,54050,27884,49856,60622,60612,60622,60622,10101,8244],"quality":"More is better","isMain":true,"metricName":"Total length (>= 1000 bp)"},{"values":[0,0,12150,5107,6705,0,0,12135,12280,12135,12135,5038,5155],"quality":"More is better","isMain":false,"metricName":"Total length (>= 5000 bp)"},{"values":[0,0,0,0,0,0,0,0,0,0,0,0,0],"quality":"More is better","isMain":true,"metricName":"Total length (>= 10000 bp)"},{"values":[0,0,0,0,0,0,0,0,0,0,0,0,0],"quality":"More is better","isMain":false,"metricName":"Total length (>= 25000 bp)"},{"values":[0,0,0,0,0,0,0,0,0,0,0,0,0],"quality":"More is better","isMain":true,"metricName":"Total length (>= 50000 bp)"},{"values":[849,733,2057,1844,1861,1358,1727,2063,2063,2063,2063,2385,5155],"quality":"More is better","isMain":false,"metricName":"N50"},{"values":[770,629,1474,1404,1381,974,1012,1602,1602,1602,1602,957,3089],"quality":"More is better","isMain":false,"metricName":"N75"},{"values":[9,9,11,13,11,10,13,11,11,11,11,2,1],"quality":"Less is better","isMain":false,"metricName":"L50"},{"values":[16,15,21,22,21,18,24,20,20,20,20,5,2],"quality":"Less is better","isMain":false,"metricName":"L75"},{"values":["57.65","57.53","56.82","56.81","56.80","57.20","56.88","56.81","56.81","56.82","56.82","58.02","56.98"],"quality":"Equal","isMain":false,"metricName":"GC (%)"}]],["Predicted genes",[]],["Similarity statistics",[{"values":[0,0,0,0,0,0,0,0,0,0,0,0,0],"quality":"Equal","isMain":false,"metricName":"# similar correct contigs"},{"values":[0,0,0,0,0,0,0,0,0,0,0,0,0],"quality":"Equal","isMain":false,"metricName":"# similar misassembled blocks"}]],["Reference statistics",[{"values":[78441,78441,78441,78441,78441,78441,78441,78441,78441,78441,78441,78441,78441],"quality":"Equal","isMain":false,"metricName":"Reference length"},{"values":[1,1,1,1,1,1,1,1,1,1,1,1,1],"quality":"Equal","isMain":false,"metricName":"Reference fragments"},{"values":["56.85","56.85","56.85","56.85","56.85","56.85","56.85","56.85","56.85","56.85","56.85","56.85","56.85"],"quality":"Equal","isMain":false,"metricName":"Reference GC (%)"}]]],"referenceName":"gi\_109521437\_ref\_NC","date":"18 July 2018, Wednesday, 16:06:48","order":[0,1,2,3,4,5,6,7,8,9,10,11,12],"assembliesNames":["ABySS\_127","ABySS\_63","CLC","IDBA\_UD","MEGAHIT","MIRA","SOAPdenovo2","SPAdes","SPAdes\_meta","SPAdes\_sc","SPAdes\_sc\_careful","Velvet","Geneious"]},{"assembliesWithNs":null,"minContig":500,"report":[["Genome statistics",[{"values":["99.695","100.000","99.068","99.824","99.836","28.120","100.000","6.311","99.881","100.000","99.876","99.881","93.499"],"quality":"More is better","isMain":true,"metricName":"Genome fraction (%)"},{"values":["1.008","1.007","1.007","1.009","1.011","1.002","1.132","1.000","1.041","1.001","1.008","1.011","1.004"],"quality":"Less is better","isMain":true,"metricName":"Duplication ratio"},{"values":[35003,35983,25891,32718,31553,2849,48398,712,39183,67243,48701,49006,6864],"quality":"More is better","isMain":true,"metricName":"Largest alignment"},{"values":[67536,67660,66992,67664,67834,18930,76051,4240,68886,67243,67230,67438,63014],"quality":"More is better","isMain":true,"metricName":"Total aligned length"},{"values":[35003,35983,10470,18305,18683,null,48398,null,40166,67243,49021,49326,2692],"quality":"More is better","isMain":false,"metricName":"NG50"},{"values":[32533,31677,8190,18305,9134,null,27653,null,23100,67243,18651,9910,1245],"quality":"More is better","isMain":false,"metricName":"NG75"},{"values":[35003,35983,10470,18305,18683,1276,48398,593,39183,67243,48701,49006,2692],"quality":"More is better","isMain":false,"metricName":"NA50"},{"values":[32533,31677,8190,18305,9134,920,27653,571,22331,67243,18529,9791,1695],"quality":"More is better","isMain":false,"metricName":"NA75"},{"values":[35003,35983,10470,18305,18683,null,48398,null,39183,67243,48701,49006,2692],"quality":"More is better","isMain":true,"metricName":"NGA50"},{"values":[32533,31677,8190,18305,9134,null,27653,null,22331,67243,18529,9791,1245],"quality":"More is better","isMain":false,"metricName":"NGA75"},{"values":[1,1,2,2,2,null,1,null,1,1,1,1,7],"quality":"Less is better","isMain":false,"metricName":"LG50"},{"values":[2,2,4,2,3,null,2,null,2,1,2,2,17],"quality":"Less is better","isMain":false,"metricName":"LG75"},{"values":[1,1,2,2,2,5,1,4,1,1,1,1,7],"quality":"Less is better","isMain":false,"metricName":"LA50"},{"values":[2,2,4,2,3,10,2,5,2,1,2,2,14],"quality":"Less is better","isMain":false,"metricName":"LA75"},{"values":[1,1,2,2,2,null,1,null,1,1,1,1,7],"quality":"Less is better","isMain":true,"metricName":"LGA50"},{"values":[2,2,4,2,3,null,2,null,2,1,2,2,17],"quality":"Less is better","isMain":false,"metricName":"LGA75"}]],["Misassemblies",[{"values":[0,0,0,0,0,0,0,0,0,0,0,0,0],"quality":"Less is better","isMain":true,"metricName":"# misassemblies"},{"values":[0,0,0,0,0,0,0,0,0,0,0,0,0],"quality":"Less is better","isMain":false,"metricName":" # relocations"},{"values":[0,0,0,0,0,0,0,0,0,0,0,0,0],"quality":"Less is better","isMain":false,"metricName":" # translocations"},{"values":[0,0,0,0,0,0,0,0,0,0,0,0,0],"quality":"Less is better","isMain":false,"metricName":" # inversions"},{"values":[0,0,0,0,0,0,0,0,0,0,0,0,0],"quality":"Less is better","isMain":false,"metricName":"# misassembled contigs"},{"values":[0,0,0,0,0,0,0,0,0,0,0,0,0],"quality":"Less is better","isMain":true,"metricName":"Misassembled contigs length"},{"values":[0,0,0,0,0,0,0,0,0,0,1,1,0],"quality":"Less is better","isMain":false,"metricName":"# local misassemblies"},{"values":[0,0,0,0,0,0,0,0,0,0,0,0,0],"quality":"Less is better","isMain":false,"metricName":"# unaligned mis. contigs"}]],["Unaligned",[{"values":[0,0,0,0,0,0,0,0,0,0,0,0,0],"quality":"Less is better","isMain":false,"metricName":"# fully unaligned contigs"},{"values":[0,0,0,0,0,0,0,0,0,0,0,0,0],"quality":"Less is better","isMain":false,"metricName":"Fully unaligned length"},{"values":[0,0,0,0,0,0,0,0,1,0,0,0,0],"quality":"Less is better","isMain":false,"metricName":"# partially unaligned contigs"},{"values":[0,0,0,0,0,0,0,0,769,0,0,0,0],"quality":"Less is better","isMain":false,"metricName":"Partially unaligned length"}]],["Mismatches",[{"values":[0,0,10,0,14,12,3,0,0,0,3,0,4],"quality":"Less is better","isMain":false,"metricName":"# mismatches"},{"values":[1,1,0,0,4,0,0,0,1,1,3,1,0],"quality":"Less is better","isMain":false,"metricName":"# indels"},{"values":[9,9,0,0,51,0,0,0,42,55,57,42,0],"quality":"Less is better","isMain":false,"metricName":"Indels length"},{"values":["0.00","0.00","15.02","0.00","20.87","63.52","4.47","0.00","0.00","0.00","4.47","0.00","6.37"],"quality":"Less is better","isMain":true,"metricName":"# mismatches per 100 kbp"},{"values":["1.49","1.49","0.00","0.00","5.96","0.00","0.00","0.00","1.49","1.49","4.47","1.49","0.00"],"quality":"Less is better","isMain":true,"metricName":"# indels per 100 kbp"},{"values":[0,0,0,0,1,0,0,0,0,0,0,0,0],"quality":"Less is better","isMain":false,"metricName":" # indels (<= 5 bp)"},{"values":[1,1,0,0,3,0,0,0,1,1,3,1,0],"quality":"Less is better","isMain":false,"metricName":" # indels (> 5 bp)"},{"values":[0,5,0,0,0,1,0,0,1752,0,122,118,0],"quality":"Less is better","isMain":false,"metricName":"# N's"},{"values":["0.00","7.39","0.00","0.00","0.00","5.28","0.00","0.00","2480.25","0.00","180.28","173.84","0.00"],"quality":"Less is better","isMain":true,"metricName":"# N's per 100 kbp"}]],["Statistics without reference",[{"values":[2,2,10,8,6,17,2,7,10,1,2,6,29],"quality":"Equal","isMain":true,"metricName":"# contigs"},{"values":[2,2,7,8,5,6,2,0,3,1,2,4,23],"quality":"Equal","isMain":false,"metricName":"# contigs (>= 1000 bp)"},{"values":[2,2,5,3,4,0,2,0,2,1,2,2,3],"quality":"Equal","isMain":false,"metricName":"# contigs (>= 5000 bp)"},{"values":[2,2,2,2,2,0,2,0,2,1,2,1,0],"quality":"Equal","isMain":false,"metricName":"# contigs (>= 10000 bp)"},{"values":[2,2,1,1,1,0,2,0,1,1,1,1,0],"quality":"Equal","isMain":false,"metricName":"# contigs (>= 25000 bp)"},{"values":[0,0,0,0,0,0,0,0,0,1,0,0,0],"quality":"Equal","isMain":false,"metricName":"# contigs (>= 50000 bp)"},{"values":[35003,35983,25891,32718,31553,2849,48398,712,40166,67243,49021,49326,6864],"quality":"More is better","isMain":true,"metricName":"Largest contig"},{"values":[67536,67660,67043,67664,67834,18930,76051,4240,70638,67243,67672,67877,63061],"quality":"More is better","isMain":true,"metricName":"Total length"},{"values":[67536,67660,65189,67664,66905,10613,76051,0,66122,67243,67672,66025,58395],"quality":"More is better","isMain":true,"metricName":"Total length (>= 1000 bp)"},{"values":[67536,67660,58138,59493,64495,0,76051,0,63266,67243,67672,59236,18236],"quality":"More is better","isMain":false,"metricName":"Total length (>= 5000 bp)"},{"values":[67536,67660,36361,51023,50236,0,76051,0,63266,67243,67672,49326,0],"quality":"More is better","isMain":true,"metricName":"Total length (>= 10000 bp)"},{"values":[67536,67660,25891,32718,31553,0,76051,0,40166,67243,49021,49326,0],"quality":"More is better","isMain":false,"metricName":"Total length (>= 25000 bp)"},{"values":[0,0,0,0,0,0,0,0,0,67243,0,0,0],"quality":"More is better","isMain":true,"metricName":"Total length (>= 50000 bp)"},{"values":[35003,35983,10470,18305,18683,1276,48398,593,40166,67243,49021,49326,2692],"quality":"More is better","isMain":false,"metricName":"N50"},{"values":[32533,31677,8190,18305,9134,920,27653,571,23100,67243,18651,9910,1695],"quality":"More is better","isMain":false,"metricName":"N75"},{"values":[1,1,2,2,2,5,1,4,1,1,1,1,7],"quality":"Less is better","isMain":false,"metricName":"L50"},{"values":[2,2,4,2,3,10,2,5,2,1,2,2,14],"quality":"Less is better","isMain":false,"metricName":"L75"},{"values":["69.04","68.96","69.00","68.95","68.98","68.84","69.06","69.15","68.93","68.97","68.97","68.98","68.91"],"quality":"Equal","isMain":false,"metricName":"GC (%)"}]],["Predicted genes",[]],["Similarity statistics",[{"values":[0,0,0,0,0,0,0,0,0,0,0,0,0],"quality":"Equal","isMain":false,"metricName":"# similar correct contigs"},{"values":[0,0,0,0,0,0,0,0,0,0,0,0,0],"quality":"Equal","isMain":false,"metricName":"# similar misassembled blocks"}]],["Reference statistics",[{"values":[67188,67188,67188,67188,67188,67188,67188,67188,67188,67188,67188,67188,67188],"quality":"Equal","isMain":false,"metricName":"Reference length"},{"values":[1,1,1,1,1,1,1,1,1,1,1,1,1],"quality":"Equal","isMain":false,"metricName":"Reference fragments"},{"values":["68.96","68.96","68.96","68.96","68.96","68.96","68.96","68.96","68.96","68.96","68.96","68.96","68.96"],"quality":"Equal","isMain":false,"metricName":"Reference GC (%)"}]]],"referenceName":"gi\_109521758\_ref\_NC","date":"18 July 2018, Wednesday, 16:06:58","order":[0,1,2,3,4,5,6,7,8,9,10,11,12],"assembliesNames":["ABySS\_127","ABySS\_63","CLC","IDBA\_UD","MEGAHIT","MIRA","Ray\_Meta","SOAPdenovo2","SPAdes","SPAdes\_meta","SPAdes\_sc","SPAdes\_sc\_careful","Velvet"]},{"assembliesWithNs":null,"minContig":500,"report":[["Genome statistics",[{"values":["16.290","1.932","3.105","10.582","8.926","71.316","3.112","12.549","6.046","3.462","11.836","0.882","4.056"],"quality":"More is better","isMain":true,"metricName":"Genome fraction (%)"},{"values":["1.000","1.000","1.000","1.000","1.010","1.100","1.000","1.025","1.000","1.000","1.064","1.000","1.000"],"quality":"Less is better","isMain":true,"metricName":"Duplication ratio"},{"values":[1102,673,1041,1898,1759,4134,1042,1926,1853,1854,5780,609,2801],"quality":"More is better","isMain":true,"metricName":"Largest alignment"},{"values":[11250,1334,2144,7308,6164,54155,2149,8832,4175,2391,8696,609,2801],"quality":"More is better","isMain":true,"metricName":"Total aligned length"},{"values":[null,null,null,null,null,1515,null,null,null,null,null,null,null],"quality":"More is better","isMain":false,"metricName":"NG50"},{"values":[null,null,null,null,null,925,null,null,null,null,null,null,null],"quality":"More is better","isMain":false,"metricName":"NG75"},{"values":[686,673,593,636,858,1838,595,672,657,1854,5780,609,2801],"quality":"More is better","isMain":false,"metricName":"NA50"},{"values":[594,661,593,545,619,1394,595,584,551,1854,1854,609,2801],"quality":"More is better","isMain":false,"metricName":"NA75"},{"values":[null,null,null,null,null,1515,null,null,null,null,null,null,null],"quality":"More is better","isMain":true,"metricName":"NGA50"},{"values":[null,null,null,null,null,925,null,null,null,null,null,null,null],"quality":"More is better","isMain":false,"metricName":"NGA75"},{"values":[null,null,null,null,null,16,null,null,null,null,null,null,null],"quality":"Less is better","isMain":false,"metricName":"LG50"},{"values":[null,null,null,null,null,30,null,null,null,null,null,null,null],"quality":"Less is better","isMain":false,"metricName":"LG75"},{"values":[7,1,2,4,3,11,2,4,2,1,1,1,1],"quality":"Less is better","isMain":false,"metricName":"LA50"},{"values":[11,2,2,7,5,20,2,8,4,1,2,1,1],"quality":"Less is better","isMain":false,"metricName":"LA75"},{"values":[null,null,null,null,null,16,null,null,null,null,null,null,null],"quality":"Less is better","isMain":true,"metricName":"LGA50"},{"values":[null,null,null,null,null,30,null,null,null,null,null,null,null],"quality":"Less is better","isMain":false,"metricName":"LGA75"}]],["Misassemblies",[{"values":[0,0,0,0,0,0,0,0,0,0,0,0,0],"quality":"Less is better","isMain":true,"metricName":"# misassemblies"},{"values":[0,0,0,0,0,0,0,0,0,0,0,0,0],"quality":"Less is better","isMain":false,"metricName":" # relocations"},{"values":[0,0,0,0,0,0,0,0,0,0,0,0,0],"quality":"Less is better","isMain":false,"metricName":" # translocations"},{"values":[0,0,0,0,0,0,0,0,0,0,0,0,0],"quality":"Less is better","isMain":false,"metricName":" # inversions"},{"values":[0,0,0,0,0,0,0,0,0,0,0,0,0],"quality":"Less is better","isMain":false,"metricName":"# misassembled contigs"},{"values":[0,0,0,0,0,0,0,0,0,0,0,0,0],"quality":"Less is better","isMain":true,"metricName":"Misassembled contigs length"},{"values":[0,0,0,0,0,0,0,0,0,0,0,0,0],"quality":"Less is better","isMain":false,"metricName":"# local misassemblies"},{"values":[0,0,0,0,0,0,0,0,0,0,0,0,0],"quality":"Less is better","isMain":false,"metricName":"# unaligned mis. contigs"}]],["Unaligned",[{"values":[0,0,0,0,0,0,0,0,0,0,0,0,0],"quality":"Less is better","isMain":false,"metricName":"# fully unaligned contigs"},{"values":[0,0,0,0,0,0,0,0,0,0,0,0,0],"quality":"Less is better","isMain":false,"metricName":"Fully unaligned length"},{"values":[0,0,0,0,0,0,0,0,0,0,0,0,0],"quality":"Less is better","isMain":false,"metricName":"# partially unaligned contigs"},{"values":[0,0,0,0,0,0,0,0,0,0,0,0,0],"quality":"Less is better","isMain":false,"metricName":"Partially unaligned length"}]],["Mismatches",[{"values":[5,0,0,0,13,154,0,11,3,0,91,0,7],"quality":"Less is better","isMain":false,"metricName":"# mismatches"},{"values":[0,0,0,0,0,5,0,0,0,0,1,0,0],"quality":"Less is better","isMain":false,"metricName":"# indels"},{"values":[0,0,0,0,0,6,0,0,0,0,3,0,0],"quality":"Less is better","isMain":false,"metricName":"Indels length"},{"values":["44.44","0.00","0.00","0.00","210.90","312.69","0.00","126.93","71.86","0.00","1113.29","0.00","249.91"],"quality":"Less is better","isMain":true,"metricName":"# mismatches per 100 kbp"},{"values":["0.00","0.00","0.00","0.00","0.00","10.15","0.00","0.00","0.00","0.00","12.23","0.00","0.00"],"quality":"Less is better","isMain":true,"metricName":"# indels per 100 kbp"},{"values":[0,0,0,0,0,5,0,0,0,0,1,0,0],"quality":"Less is better","isMain":false,"metricName":" # indels (<= 5 bp)"},{"values":[0,0,0,0,0,0,0,0,0,0,0,0,0],"quality":"Less is better","isMain":false,"metricName":" # indels (> 5 bp)"},{"values":[0,0,0,0,0,66,0,0,0,0,0,0,21],"quality":"Less is better","isMain":false,"metricName":"# N's"},{"values":["0.00","0.00","0.00","0.00","0.00","121.86","0.00","0.00","0.00","0.00","0.00","0.00","749.73"],"quality":"Less is better","isMain":true,"metricName":"# N's per 100 kbp"}]],["Statistics without reference",[{"values":[16,2,3,10,7,33,3,11,5,2,4,1,1],"quality":"Equal","isMain":true,"metricName":"# contigs"},{"values":[1,0,1,1,1,28,1,2,1,1,2,0,1],"quality":"Equal","isMain":false,"metricName":"# contigs (>= 1000 bp)"},{"values":[0,0,0,0,0,0,0,0,0,0,1,0,0],"quality":"Equal","isMain":false,"metricName":"# contigs (>= 5000 bp)"},{"values":[0,0,0,0,0,0,0,0,0,0,0,0,0],"quality":"Equal","isMain":false,"metricName":"# contigs (>= 10000 bp)"},{"values":[0,0,0,0,0,0,0,0,0,0,0,0,0],"quality":"Equal","isMain":false,"metricName":"# contigs (>= 25000 bp)"},{"values":[0,0,0,0,0,0,0,0,0,0,0,0,0],"quality":"Equal","isMain":false,"metricName":"# contigs (>= 50000 bp)"},{"values":[1102,673,1041,1898,1759,4134,1042,1926,1853,1854,5780,609,2801],"quality":"More is better","isMain":true,"metricName":"Largest contig"},{"values":[11250,1334,2144,7308,6225,54160,2149,8883,4175,2391,8696,609,2801],"quality":"More is better","isMain":true,"metricName":"Total length"},{"values":[1102,0,1041,1898,1759,50084,1042,3371,1853,1854,7634,0,2801],"quality":"More is better","isMain":true,"metricName":"Total length (>= 1000 bp)"},{"values":[0,0,0,0,0,0,0,0,0,0,5780,0,0],"quality":"More is better","isMain":false,"metricName":"Total length (>= 5000 bp)"},{"values":[0,0,0,0,0,0,0,0,0,0,0,0,0],"quality":"More is better","isMain":true,"metricName":"Total length (>= 10000 bp)"},{"values":[0,0,0,0,0,0,0,0,0,0,0,0,0],"quality":"More is better","isMain":false,"metricName":"Total length (>= 25000 bp)"},{"values":[0,0,0,0,0,0,0,0,0,0,0,0,0],"quality":"More is better","isMain":true,"metricName":"Total length (>= 50000 bp)"},{"values":[686,673,593,636,858,1838,595,672,657,1854,5780,609,2801],"quality":"More is better","isMain":false,"metricName":"N50"},{"values":[594,661,593,545,668,1394,595,596,551,1854,1854,609,2801],"quality":"More is better","isMain":false,"metricName":"N75"},{"values":[7,1,2,4,3,11,2,4,2,1,1,1,1],"quality":"Less is better","isMain":false,"metricName":"L50"},{"values":[11,2,2,7,5,20,2,7,4,1,2,1,1],"quality":"Less is better","isMain":false,"metricName":"L75"},{"values":["66.62","63.94","63.71","66.80","65.67","67.30","63.75","67.43","65.89","66.16","66.89","59.28","69.82"],"quality":"Equal","isMain":false,"metricName":"GC (%)"}]],["Predicted genes",[]],["Similarity statistics",[{"values":[0,0,0,0,0,0,0,0,0,0,0,0,0],"quality":"Equal","isMain":false,"metricName":"# similar correct contigs"},{"values":[0,0,0,0,0,0,0,0,0,0,0,0,0],"quality":"Equal","isMain":false,"metricName":"# similar misassembled blocks"}]],["Reference statistics",[{"values":[69059,69059,69059,69059,69059,69059,69059,69059,69059,69059,69059,69059,69059],"quality":"Equal","isMain":false,"metricName":"Reference length"},{"values":[1,1,1,1,1,1,1,1,1,1,1,1,1],"quality":"Equal","isMain":false,"metricName":"Reference fragments"},{"values":["67.34","67.34","67.34","67.34","67.34","67.34","67.34","67.34","67.34","67.34","67.34","67.34","67.34"],"quality":"Equal","isMain":false,"metricName":"Reference GC (%)"}]]],"referenceName":"gi\_109521840\_ref\_NC","date":"18 July 2018, Wednesday, 16:07:09","order":[0,1,2,3,4,5,6,7,8,9,10,11,12],"assembliesNames":["ABySS\_127","ABySS\_63","CLC","IDBA\_UD","MEGAHIT","MIRA","SOAPdenovo2","SPAdes","SPAdes\_meta","SPAdes\_sc","SPAdes\_sc\_careful","Velvet","Geneious"]},{"assembliesWithNs":null,"minContig":500,"report":[["Genome statistics",[{"values":["1.789","0.991","0.849","0.729","4.039","0.980","0.980"],"quality":"More is better","isMain":true,"metricName":"Genome fraction (%)"},{"values":["1.000","1.000","1.000","1.511","1.045","1.000","1.000"],"quality":"Less is better","isMain":true,"metricName":"Duplication ratio"},{"values":[1159,642,550,472,617,635,635],"quality":"More is better","isMain":true,"metricName":"Largest alignment"},{"values":[1159,642,550,472,2734,635,635],"quality":"More is better","isMain":true,"metricName":"Total aligned length"},{"values":[1159,642,550,472,523,635,635],"quality":"More is better","isMain":false,"metricName":"NA50"},{"values":[1159,642,550,null,520,635,635],"quality":"More is better","isMain":false,"metricName":"NA75"},{"values":[null,null,null,null,null,null,null],"quality":"More is better","isMain":true,"metricName":"NGA50"},{"values":[1,1,1,1,3,1,1],"quality":"Less is better","isMain":false,"metricName":"LA50"},{"values":[1,1,1,null,4,1,1],"quality":"Less is better","isMain":false,"metricName":"LA75"}]],["Misassemblies",[{"values":[0,0,0,0,0,0,0],"quality":"Less is better","isMain":true,"metricName":"# misassemblies"},{"values":[0,0,0,0,0,0,0],"quality":"Less is better","isMain":false,"metricName":" # relocations"},{"values":[0,0,0,0,0,0,0],"quality":"Less is better","isMain":false,"metricName":" # translocations"},{"values":[0,0,0,0,0,0,0],"quality":"Less is better","isMain":false,"metricName":" # inversions"},{"values":[0,0,0,0,0,0,0],"quality":"Less is better","isMain":false,"metricName":"# misassembled contigs"},{"values":[0,0,0,0,0,0,0],"quality":"Less is better","isMain":true,"metricName":"Misassembled contigs length"},{"values":[0,0,0,0,0,0,0],"quality":"Less is better","isMain":false,"metricName":"# local misassemblies"},{"values":[0,0,0,0,0,0,0],"quality":"Less is better","isMain":false,"metricName":"# unaligned mis. contigs"}]],["Unaligned",[{"values":[0,0,0,0,0,0,0],"quality":"Less is better","isMain":false,"metricName":"# fully unaligned contigs"},{"values":[0,0,0,0,0,0,0],"quality":"Less is better","isMain":false,"metricName":"Fully unaligned length"},{"values":[0,0,0,0,0,0,0],"quality":"Less is better","isMain":false,"metricName":"# partially unaligned contigs"},{"values":[0,0,0,0,0,0,0],"quality":"Less is better","isMain":false,"metricName":"Partially unaligned length"}]],["Mismatches",[{"values":[3,6,1,11,4,1,1],"quality":"Less is better","isMain":false,"metricName":"# mismatches"},{"values":[0,0,0,0,0,0,0],"quality":"Less is better","isMain":false,"metricName":"# indels"},{"values":[0,0,0,0,0,0,0],"quality":"Less is better","isMain":false,"metricName":"Indels length"},{"values":["258.84","934.58","181.82","2330.51","152.85","157.48","157.48"],"quality":"Less is better","isMain":true,"metricName":"# mismatches per 100 kbp"},{"values":["0.00","0.00","0.00","0.00","0.00","0.00","0.00"],"quality":"Less is better","isMain":true,"metricName":"# indels per 100 kbp"},{"values":[0,0,0,0,0,0,0],"quality":"Less is better","isMain":false,"metricName":" # indels (<= 5 bp)"},{"values":[0,0,0,0,0,0,0],"quality":"Less is better","isMain":false,"metricName":" # indels (> 5 bp)"},{"values":[0,0,0,47,0,0,0],"quality":"Less is better","isMain":false,"metricName":"# N's"},{"values":["0.00","0.00","0.00","6591.87","0.00","0.00","0.00"],"quality":"Less is better","isMain":true,"metricName":"# N's per 100 kbp"}]],["Statistics without reference",[{"values":[1,1,1,1,5,1,1],"quality":"Equal","isMain":true,"metricName":"# contigs"},{"values":[1,0,0,0,0,0,0],"quality":"Equal","isMain":false,"metricName":"# contigs (>= 1000 bp)"},{"values":[0,0,0,0,0,0,0],"quality":"Equal","isMain":false,"metricName":"# contigs (>= 5000 bp)"},{"values":[0,0,0,0,0,0,0],"quality":"Equal","isMain":false,"metricName":"# contigs (>= 10000 bp)"},{"values":[0,0,0,0,0,0,0],"quality":"Equal","isMain":false,"metricName":"# contigs (>= 25000 bp)"},{"values":[0,0,0,0,0,0,0],"quality":"Equal","isMain":false,"metricName":"# contigs (>= 50000 bp)"},{"values":[1159,642,550,713,617,635,635],"quality":"More is better","isMain":true,"metricName":"Largest contig"},{"values":[1159,642,550,713,2734,635,635],"quality":"More is better","isMain":true,"metricName":"Total length"},{"values":[1159,0,0,0,0,0,0],"quality":"More is better","isMain":true,"metricName":"Total length (>= 1000 bp)"},{"values":[0,0,0,0,0,0,0],"quality":"More is better","isMain":false,"metricName":"Total length (>= 5000 bp)"},{"values":[0,0,0,0,0,0,0],"quality":"More is better","isMain":true,"metricName":"Total length (>= 10000 bp)"},{"values":[0,0,0,0,0,0,0],"quality":"More is better","isMain":false,"metricName":"Total length (>= 25000 bp)"},{"values":[0,0,0,0,0,0,0],"quality":"More is better","isMain":true,"metricName":"Total length (>= 50000 bp)"},{"values":[1159,642,550,713,523,635,635],"quality":"More is better","isMain":false,"metricName":"N50"},{"values":[1159,642,550,713,520,635,635],"quality":"More is better","isMain":false,"metricName":"N75"},{"values":[1,1,1,1,3,1,1],"quality":"Less is better","isMain":false,"metricName":"L50"},{"values":[1,1,1,1,4,1,1],"quality":"Less is better","isMain":false,"metricName":"L75"},{"values":["57.81","58.88","63.45","60.36","58.19","57.32","57.32"],"quality":"Equal","isMain":false,"metricName":"GC (%)"}]],["Predicted genes",[]],["Similarity statistics",[{"values":[0,0,0,0,0,0,0],"quality":"Equal","isMain":false,"metricName":"# similar correct contigs"},{"values":[0,0,0,0,0,0,0],"quality":"Equal","isMain":false,"metricName":"# similar misassembled blocks"}]],["Reference statistics",[{"values":[64787,64787,64787,64787,64787,64787,64787],"quality":"Equal","isMain":false,"metricName":"Reference length"},{"values":[1,1,1,1,1,1,1],"quality":"Equal","isMain":false,"metricName":"Reference fragments"},{"values":["59.66","59.66","59.66","59.66","59.66","59.66","59.66"],"quality":"Equal","isMain":false,"metricName":"Reference GC (%)"}]]],"referenceName":"gi\_109522567\_ref\_NC","date":"18 July 2018, Wednesday, 16:07:16","order":[0,1,2,3,4,5,6],"assembliesNames":["ABySS\_127","CLC","MEGAHIT","SOAPdenovo2","SPAdes","SPAdes\_sc","SPAdes\_sc\_careful"]},{"assembliesWithNs":null,"minContig":500,"report":[["Genome statistics",[{"values":["13.854","7.201","13.373","0.919","9.660","14.914","14.775","14.593","14.782"],"quality":"More is better","isMain":true,"metricName":"Genome fraction (%)"},{"values":["1.000","1.000","1.000","1.000","1.000","1.000","1.000","1.000","1.000"],"quality":"Less is better","isMain":true,"metricName":"Duplication ratio"},{"values":[1968,1678,1624,1170,1380,1968,1968,1968,1968],"quality":"More is better","isMain":true,"metricName":"Largest alignment"},{"values":[30597,15905,29535,2030,21336,32939,32631,32230,32647],"quality":"More is better","isMain":true,"metricName":"Total aligned length"},{"values":[850,852,771,1170,718,874,850,850,850],"quality":"More is better","isMain":false,"metricName":"NA50"},{"values":[738,557,628,860,580,742,742,738,738],"quality":"More is better","isMain":false,"metricName":"NA75"},{"values":[null,null,null,null,null,null,null,null,null],"quality":"More is better","isMain":true,"metricName":"NGA50"},{"values":[14,8,16,1,11,15,15,15,15],"quality":"Less is better","isMain":false,"metricName":"LA50"},{"values":[24,15,26,2,20,25,25,25,25],"quality":"Less is better","isMain":false,"metricName":"LA75"}]],["Misassemblies",[{"values":[0,0,0,0,0,0,0,0,0],"quality":"Less is better","isMain":true,"metricName":"# misassemblies"},{"values":[0,0,0,0,0,0,0,0,0],"quality":"Less is better","isMain":false,"metricName":" # relocations"},{"values":[0,0,0,0,0,0,0,0,0],"quality":"Less is better","isMain":false,"metricName":" # translocations"},{"values":[0,0,0,0,0,0,0,0,0],"quality":"Less is better","isMain":false,"metricName":" # inversions"},{"values":[0,0,0,0,0,0,0,0,0],"quality":"Less is better","isMain":false,"metricName":"# misassembled contigs"},{"values":[0,0,0,0,0,0,0,0,0],"quality":"Less is better","isMain":true,"metricName":"Misassembled contigs length"},{"values":[0,0,1,0,0,0,0,0,0],"quality":"Less is better","isMain":false,"metricName":"# local misassemblies"},{"values":[0,0,0,0,0,0,0,0,0],"quality":"Less is better","isMain":false,"metricName":"# unaligned mis. contigs"}]],["Unaligned",[{"values":[0,0,0,0,0,0,0,0,0],"quality":"Less is better","isMain":false,"metricName":"# fully unaligned contigs"},{"values":[0,0,0,0,0,0,0,0,0],"quality":"Less is better","isMain":false,"metricName":"Fully unaligned length"},{"values":[0,1,0,0,0,0,0,0,0],"quality":"Less is better","isMain":false,"metricName":"# partially unaligned contigs"},{"values":[0,1456,0,0,0,0,0,0,0],"quality":"Less is better","isMain":false,"metricName":"Partially unaligned length"}]],["Mismatches",[{"values":[50,26,41,0,21,53,48,45,44],"quality":"Less is better","isMain":false,"metricName":"# mismatches"},{"values":[0,0,1,0,0,0,0,0,0],"quality":"Less is better","isMain":false,"metricName":"# indels"},{"values":[0,0,78,0,0,0,0,0,0],"quality":"Less is better","isMain":false,"metricName":"Indels length"},{"values":["163.41","163.47","138.82","0.00","98.43","160.90","147.10","139.62","134.78"],"quality":"Less is better","isMain":true,"metricName":"# mismatches per 100 kbp"},{"values":["0.00","0.00","3.39","0.00","0.00","0.00","0.00","0.00","0.00"],"quality":"Less is better","isMain":true,"metricName":"# indels per 100 kbp"},{"values":[0,0,0,0,0,0,0,0,0],"quality":"Less is better","isMain":false,"metricName":" # indels (<= 5 bp)"},{"values":[0,0,1,0,0,0,0,0,0],"quality":"Less is better","isMain":false,"metricName":" # indels (> 5 bp)"},{"values":[0,0,0,2,0,0,0,0,0],"quality":"Less is better","isMain":false,"metricName":"# N's"},{"values":["0.00","0.00","0.00","98.52","0.00","0.00","0.00","0.00","0.00"],"quality":"Less is better","isMain":true,"metricName":"# N's per 100 kbp"}]],["Statistics without reference",[{"values":[36,20,39,2,29,38,38,38,38],"quality":"Equal","isMain":true,"metricName":"# contigs"},{"values":[9,6,4,1,3,11,10,10,10],"quality":"Equal","isMain":false,"metricName":"# contigs (>= 1000 bp)"},{"values":[0,0,0,0,0,0,0,0,0],"quality":"Equal","isMain":false,"metricName":"# contigs (>= 5000 bp)"},{"values":[0,0,0,0,0,0,0,0,0],"quality":"Equal","isMain":false,"metricName":"# contigs (>= 10000 bp)"},{"values":[0,0,0,0,0,0,0,0,0],"quality":"Equal","isMain":false,"metricName":"# contigs (>= 25000 bp)"},{"values":[0,0,0,0,0,0,0,0,0],"quality":"Equal","isMain":false,"metricName":"# contigs (>= 50000 bp)"},{"values":[1968,1716,1624,1170,1380,1968,1968,1968,1968],"quality":"More is better","isMain":true,"metricName":"Largest contig"},{"values":[30597,17361,29535,2030,21336,32939,32632,32230,32647],"quality":"More is better","isMain":true,"metricName":"Total length"},{"values":[11335,7884,5108,1170,3713,13489,12464,12464,12464],"quality":"More is better","isMain":true,"metricName":"Total length (>= 1000 bp)"},{"values":[0,0,0,0,0,0,0,0,0],"quality":"More is better","isMain":false,"metricName":"Total length (>= 5000 bp)"},{"values":[0,0,0,0,0,0,0,0,0],"quality":"More is better","isMain":true,"metricName":"Total length (>= 10000 bp)"},{"values":[0,0,0,0,0,0,0,0,0],"quality":"More is better","isMain":false,"metricName":"Total length (>= 25000 bp)"},{"values":[0,0,0,0,0,0,0,0,0],"quality":"More is better","isMain":true,"metricName":"Total length (>= 50000 bp)"},{"values":[850,909,771,1170,718,874,850,850,850],"quality":"More is better","isMain":false,"metricName":"N50"},{"values":[738,695,628,860,580,742,742,738,738],"quality":"More is better","isMain":false,"metricName":"N75"},{"values":[14,7,16,1,11,15,15,15,15],"quality":"Less is better","isMain":false,"metricName":"L50"},{"values":[24,13,26,2,20,25,25,25,25],"quality":"Less is better","isMain":false,"metricName":"L75"},{"values":["54.71","53.37","55.68","62.82","54.49","55.19","55.15","54.96","54.98"],"quality":"Equal","isMain":false,"metricName":"GC (%)"}]],["Predicted genes",[]],["Similarity statistics",[{"values":[0,0,0,0,0,0,0,0,0],"quality":"Equal","isMain":false,"metricName":"# similar correct contigs"},{"values":[0,0,0,0,0,0,0,0,0],"quality":"Equal","isMain":false,"metricName":"# similar misassembled blocks"}]],["Reference statistics",[{"values":[220859,220859,220859,220859,220859,220859,220859,220859,220859],"quality":"Equal","isMain":false,"metricName":"Reference length"},{"values":[1,1,1,1,1,1,1,1,1],"quality":"Equal","isMain":false,"metricName":"Reference fragments"},{"values":["54.55","54.55","54.55","54.55","54.55","54.55","54.55","54.55","54.55"],"quality":"Equal","isMain":false,"metricName":"Reference GC (%)"}]]],"referenceName":"gi\_109638461\_ref\_NC","date":"18 July 2018, Wednesday, 16:07:24","order":[0,1,2,3,4,5,6,7,8],"assembliesNames":["CLC","IDBA\_UD","MEGAHIT","MIRA","SOAPdenovo2","SPAdes","SPAdes\_meta","SPAdes\_sc","SPAdes\_sc\_careful"]},{"assembliesWithNs":null,"minContig":500,"report":[["Genome statistics",[{"values":["2.560","0.255","49.530","34.524","43.893","7.275","38.526","51.300","51.329","51.561","51.562","0.258"],"quality":"More is better","isMain":true,"metricName":"Genome fraction (%)"},{"values":["1.010","1.000","1.002","1.003","1.001","1.000","1.000","1.003","1.004","1.003","1.003","1.000"],"quality":"Less is better","isMain":true,"metricName":"Duplication ratio"},{"values":[825,590,3177,2826,2775,1645,3177,3177,3177,3177,3177,598],"quality":"More is better","isMain":true,"metricName":"Largest alignment"},{"values":[5933,590,114863,80035,101767,16863,89323,118950,119461,119563,119561,598],"quality":"More is better","isMain":true,"metricName":"Total aligned length"},{"values":[null,null,null,null,null,null,null,541,535,547,547,null],"quality":"More is better","isMain":false,"metricName":"NG50"},{"values":[530,590,934,924,828,1067,853,939,938,941,941,null],"quality":"More is better","isMain":false,"metricName":"NA50"},{"values":[522,590,799,681,646,756,652,799,801,801,801,null],"quality":"More is better","isMain":false,"metricName":"NA75"},{"values":[null,null,null,null,null,null,null,535,535,535,535,null],"quality":"More is better","isMain":true,"metricName":"NGA50"},{"values":[null,null,null,null,null,null,null,117,118,117,117,null],"quality":"Less is better","isMain":false,"metricName":"LG50"},{"values":[5,1,44,33,45,7,37,46,45,44,44,null],"quality":"Less is better","isMain":false,"metricName":"LA50"},{"values":[8,1,77,59,80,12,67,80,79,78,78,null],"quality":"Less is better","isMain":false,"metricName":"LA75"},{"values":[null,null,null,null,null,null,null,119,118,118,118,null],"quality":"Less is better","isMain":true,"metricName":"LGA50"}]],["Misassemblies",[{"values":[0,0,0,1,0,0,0,0,0,0,0,0],"quality":"Less is better","isMain":true,"metricName":"# misassemblies"},{"values":[0,0,0,1,0,0,0,0,0,0,0,0],"quality":"Less is better","isMain":false,"metricName":" # relocations"},{"values":[0,0,0,0,0,0,0,0,0,0,0,0],"quality":"Less is better","isMain":false,"metricName":" # translocations"},{"values":[0,0,0,0,0,0,0,0,0,0,0,0],"quality":"Less is better","isMain":false,"metricName":" # inversions"},{"values":[0,0,0,1,0,0,0,0,0,0,0,0],"quality":"Less is better","isMain":false,"metricName":"# misassembled contigs"},{"values":[0,0,0,1415,0,0,0,0,0,0,0,0],"quality":"Less is better","isMain":true,"metricName":"Misassembled contigs length"},{"values":[0,0,0,0,1,0,0,0,4,3,3,0],"quality":"Less is better","isMain":false,"metricName":"# local misassemblies"},{"values":[0,0,0,0,0,0,0,0,0,0,0,0],"quality":"Less is better","isMain":false,"metricName":"# unaligned mis. contigs"}]],["Unaligned",[{"values":[0,0,0,0,0,0,0,0,0,0,0,0],"quality":"Less is better","isMain":false,"metricName":"# fully unaligned contigs"},{"values":[0,0,0,0,0,0,0,0,0,0,0,0],"quality":"Less is better","isMain":false,"metricName":"Fully unaligned length"},{"values":[0,0,0,1,0,0,0,1,1,0,0,1],"quality":"Less is better","isMain":false,"metricName":"# partially unaligned contigs"},{"values":[0,0,0,790,0,0,0,790,548,0,0,43951],"quality":"Less is better","isMain":false,"metricName":"Partially unaligned length"}]],["Mismatches",[{"values":[2,0,176,91,132,5,98,212,202,184,151,0],"quality":"Less is better","isMain":false,"metricName":"# mismatches"},{"values":[1,0,2,6,9,0,1,1,6,5,3,0],"quality":"Less is better","isMain":false,"metricName":"# indels"},{"values":[1,0,2,6,68,0,42,1,13,12,10,0],"quality":"Less is better","isMain":false,"metricName":"Indels length"},{"values":["33.70","0.00","153.30","113.71","129.74","29.65","109.74","178.28","169.78","153.95","126.34","0.00"],"quality":"Less is better","isMain":true,"metricName":"# mismatches per 100 kbp"},{"values":["16.85","0.00","1.74","7.50","8.85","0.00","1.12","0.84","5.04","4.18","2.51","0.00"],"quality":"Less is better","isMain":true,"metricName":"# indels per 100 kbp"},{"values":[1,0,2,6,7,0,0,1,5,4,2,0],"quality":"Less is better","isMain":false,"metricName":" # indels (<= 5 bp)"},{"values":[0,0,0,0,2,0,1,0,1,1,1,0],"quality":"Less is better","isMain":false,"metricName":" # indels (> 5 bp)"},{"values":[0,0,0,0,0,37,0,0,0,0,0,4],"quality":"Less is better","isMain":false,"metricName":"# N's"},{"values":["0.00","0.00","0.00","0.00","0.00","219.42","0.00","0.00","0.00","0.00","0.00","8.98"],"quality":"Less is better","isMain":true,"metricName":"# N's per 100 kbp"}]],["Statistics without reference",[{"values":[10,1,121,90,123,18,105,124,125,124,124,1],"quality":"Equal","isMain":true,"metricName":"# contigs"},{"values":[0,0,37,27,26,8,23,42,38,40,40,1],"quality":"Equal","isMain":false,"metricName":"# contigs (>= 1000 bp)"},{"values":[0,0,0,0,0,0,0,0,0,0,0,1],"quality":"Equal","isMain":false,"metricName":"# contigs (>= 5000 bp)"},{"values":[0,0,0,0,0,0,0,0,0,0,0,1],"quality":"Equal","isMain":false,"metricName":"# contigs (>= 10000 bp)"},{"values":[0,0,0,0,0,0,0,0,0,0,0,1],"quality":"Equal","isMain":false,"metricName":"# contigs (>= 25000 bp)"},{"values":[0,0,0,0,0,0,0,0,0,0,0,0],"quality":"Equal","isMain":false,"metricName":"# contigs (>= 50000 bp)"},{"values":[858,590,3177,2826,2775,1645,3177,3177,3177,3177,3177,44549],"quality":"More is better","isMain":true,"metricName":"Largest contig"},{"values":[5993,590,114989,81029,101799,16863,89323,120079,120009,119935,119933,44549],"quality":"More is better","isMain":true,"metricName":"Total length"},{"values":[0,0,51532,35703,34326,9739,32370,57969,54132,57016,57014,44549],"quality":"More is better","isMain":true,"metricName":"Total length (>= 1000 bp)"},{"values":[0,0,0,0,0,0,0,0,0,0,0,44549],"quality":"More is better","isMain":false,"metricName":"Total length (>= 5000 bp)"},{"values":[0,0,0,0,0,0,0,0,0,0,0,44549],"quality":"More is better","isMain":true,"metricName":"Total length (>= 10000 bp)"},{"values":[0,0,0,0,0,0,0,0,0,0,0,44549],"quality":"More is better","isMain":false,"metricName":"Total length (>= 25000 bp)"},{"values":[0,0,0,0,0,0,0,0,0,0,0,0],"quality":"More is better","isMain":true,"metricName":"Total length (>= 50000 bp)"},{"values":[530,590,938,952,828,1067,853,958,938,941,941,44549],"quality":"More is better","isMain":false,"metricName":"N50"},{"values":[522,590,799,707,646,756,652,805,801,801,801,44549],"quality":"More is better","isMain":false,"metricName":"N75"},{"values":[5,1,44,32,45,7,37,45,45,44,44,1],"quality":"Less is better","isMain":false,"metricName":"L50"},{"values":[8,1,77,58,80,12,67,79,79,78,78,1],"quality":"Less is better","isMain":false,"metricName":"L75"},{"values":["54.68","51.53","53.66","53.33","53.33","53.43","53.83","53.15","53.24","53.49","53.49","35.38"],"quality":"Equal","isMain":false,"metricName":"GC (%)"}]],["Predicted genes",[]],["Similarity statistics",[{"values":[0,0,0,0,0,0,0,0,0,0,0,0],"quality":"Equal","isMain":false,"metricName":"# similar correct contigs"},{"values":[0,0,0,0,0,0,0,0,0,0,0,0],"quality":"Equal","isMain":false,"metricName":"# similar misassembled blocks"}]],["Reference statistics",[{"values":[231801,231801,231801,231801,231801,231801,231801,231801,231801,231801,231801,231801],"quality":"Equal","isMain":false,"metricName":"Reference length"},{"values":[1,1,1,1,1,1,1,1,1,1,1,1],"quality":"Equal","isMain":false,"metricName":"Reference fragments"},{"values":["52.77","52.77","52.77","52.77","52.77","52.77","52.77","52.77","52.77","52.77","52.77","52.77"],"quality":"Equal","isMain":false,"metricName":"Reference GC (%)"}]]],"referenceName":"gi\_109638594\_ref\_NC","date":"18 July 2018, Wednesday, 16:07:34","order":[0,1,2,3,4,5,6,7,8,9,10,11],"assembliesNames":["ABySS\_127","ABySS\_63","CLC","IDBA\_UD","MEGAHIT","MIRA","SOAPdenovo2","SPAdes","SPAdes\_meta","SPAdes\_sc","SPAdes\_sc\_careful","Geneious"]},{"assembliesWithNs":null,"minContig":500,"report":[["Genome statistics",[{"values":["66.791","55.896","93.114","96.526","96.493","85.887","77.221","97.544","97.354","97.196","96.995","80.795","35.025"],"quality":"More is better","isMain":true,"metricName":"Genome fraction (%)"},{"values":["1.000","1.000","1.029","1.005","1.004","1.035","1.030","1.005","1.004","1.010","1.009","1.000","1.004"],"quality":"Less is better","isMain":true,"metricName":"Duplication ratio"},{"values":[3908,3017,10597,15484,11748,6222,6428,16545,16537,16432,16160,12870,7623],"quality":"More is better","isMain":true,"metricName":"Largest alignment"},{"values":[88176,73802,125501,127709,127843,116992,102921,128985,128563,129198,128950,106670,46200],"quality":"More is better","isMain":true,"metricName":"Total aligned length"},{"values":[820,586,4009,6137,6068,2468,1724,8667,6712,6668,6668,4218,null],"quality":"More is better","isMain":false,"metricName":"NG50"},{"values":[null,null,2707,3358,4046,1397,645,5113,4638,4638,4638,1364,null],"quality":"More is better","isMain":false,"metricName":"NG75"},{"values":[1144,960,4099,6137,6068,2617,1885,8667,6481,6665,6665,4432,4347],"quality":"More is better","isMain":false,"metricName":"NA50"},{"values":[820,728,2707,3354,4369,1736,1080,6121,4638,4638,4120,2434,3992],"quality":"More is better","isMain":false,"metricName":"NA75"},{"values":[820,586,4009,6137,6068,2468,1665,8667,6481,6665,6665,4218,null],"quality":"More is better","isMain":true,"metricName":"NGA50"},{"values":[null,null,2581,3354,4046,1294,568,4638,4046,4120,4120,1364,null],"quality":"More is better","isMain":false,"metricName":"NGA75"},{"values":[45,63,12,8,8,19,24,6,6,7,7,11,null],"quality":"Less is better","isMain":false,"metricName":"LG50"},{"values":[null,null,22,15,15,35,53,11,11,12,12,26,null],"quality":"Less is better","isMain":false,"metricName":"LG75"},{"values":[22,24,11,8,8,16,18,6,6,7,7,8,4],"quality":"Less is better","isMain":false,"metricName":"LA50"},{"values":[45,47,21,15,14,29,36,10,11,12,13,16,7],"quality":"Less is better","isMain":false,"metricName":"LA75"},{"values":[45,63,12,8,8,19,25,6,6,7,7,11,null],"quality":"Less is better","isMain":true,"metricName":"LGA50"},{"values":[null,null,22,15,15,36,60,11,12,13,13,26,null],"quality":"Less is better","isMain":false,"metricName":"LGA75"}]],["Misassemblies",[{"values":[0,0,0,0,0,0,0,0,0,0,0,0,0],"quality":"Less is better","isMain":true,"metricName":"# misassemblies"},{"values":[0,0,0,0,0,0,0,0,0,0,0,0,0],"quality":"Less is better","isMain":false,"metricName":" # relocations"},{"values":[0,0,0,0,0,0,0,0,0,0,0,0,0],"quality":"Less is better","isMain":false,"metricName":" # translocations"},{"values":[0,0,0,0,0,0,0,0,0,0,0,0,0],"quality":"Less is better","isMain":false,"metricName":" # inversions"},{"values":[0,0,0,0,0,0,0,0,0,0,0,0,0],"quality":"Less is better","isMain":false,"metricName":"# misassembled contigs"},{"values":[0,0,0,0,0,0,0,0,0,0,0,0,0],"quality":"Less is better","isMain":true,"metricName":"Misassembled contigs length"},{"values":[0,0,0,0,0,1,0,1,1,1,1,0,1],"quality":"Less is better","isMain":false,"metricName":"# local misassemblies"},{"values":[0,0,0,0,0,0,0,0,0,0,0,0,0],"quality":"Less is better","isMain":false,"metricName":"# unaligned mis. contigs"}]],["Unaligned",[{"values":[0,0,0,0,0,0,0,0,0,0,0,0,0],"quality":"Less is better","isMain":false,"metricName":"# fully unaligned contigs"},{"values":[0,0,0,0,0,0,0,0,0,0,0,0,0],"quality":"Less is better","isMain":false,"metricName":"Fully unaligned length"},{"values":[0,0,0,1,1,1,2,1,0,2,4,0,0],"quality":"Less is better","isMain":false,"metricName":"# partially unaligned contigs"},{"values":[0,0,0,741,672,1518,2009,537,0,1278,3400,0,0],"quality":"Less is better","isMain":false,"metricName":"Partially unaligned length"}]],["Mismatches",[{"values":[12,4,226,92,120,56,26,204,58,324,301,16,4],"quality":"Less is better","isMain":false,"metricName":"# mismatches"},{"values":[2,0,19,3,4,2,28,3,7,16,15,0,1],"quality":"Less is better","isMain":false,"metricName":"# indels"},{"values":[5,0,43,97,119,51,1312,68,39,152,145,0,31],"quality":"Less is better","isMain":false,"metricName":"Indels length"},{"values":["13.61","5.42","183.88","72.21","94.22","49.40","25.51","158.44","45.14","252.54","235.10","15.00","8.65"],"quality":"Less is better","isMain":true,"metricName":"# mismatches per 100 kbp"},{"values":["2.27","0.00","15.46","2.35","3.14","1.76","27.47","2.33","5.45","12.47","11.72","0.00","2.16"],"quality":"Less is better","isMain":true,"metricName":"# indels per 100 kbp"},{"values":[2,0,17,1,2,0,0,2,6,12,12,0,0],"quality":"Less is better","isMain":false,"metricName":" # indels (<= 5 bp)"},{"values":[0,0,2,2,2,2,28,1,1,4,3,0,1],"quality":"Less is better","isMain":false,"metricName":" # indels (> 5 bp)"},{"values":[0,0,0,0,0,127,3179,10,124,10,0,0,68],"quality":"Less is better","isMain":false,"metricName":"# N's"},{"values":["0.00","0.00","0.00","0.00","0.00","106.90","2970.31","7.69","96.09","7.64","0.00","0.00","146.55"],"quality":"Less is better","isMain":true,"metricName":"# N's per 100 kbp"}]],["Statistics without reference",[{"values":[78,77,46,31,28,56,67,22,24,26,26,35,11],"quality":"Equal","isMain":true,"metricName":"# contigs"},{"values":[31,23,31,28,26,42,42,19,22,23,23,29,11],"quality":"Equal","isMain":false,"metricName":"# contigs (>= 1000 bp)"},{"values":[0,0,7,10,10,2,2,11,10,11,11,5,3],"quality":"Equal","isMain":false,"metricName":"# contigs (>= 5000 bp)"},{"values":[0,0,1,2,2,0,0,3,3,3,3,1,0],"quality":"Equal","isMain":false,"metricName":"# contigs (>= 10000 bp)"},{"values":[0,0,0,0,0,0,0,0,0,0,0,0,0],"quality":"Equal","isMain":false,"metricName":"# contigs (>= 25000 bp)"},{"values":[0,0,0,0,0,0,0,0,0,0,0,0,0],"quality":"Equal","isMain":false,"metricName":"# contigs (>= 50000 bp)"},{"values":[3908,3017,10597,15484,11748,6222,6428,16545,16777,16432,17146,12870,7623],"quality":"More is better","isMain":true,"metricName":"Largest contig"},{"values":[88176,73802,126430,128816,128518,118805,107026,129996,129039,130914,132641,106673,46400],"quality":"More is better","isMain":true,"metricName":"Total length"},{"values":[53823,36498,116092,127042,127402,108235,90572,128152,127755,128708,130435,102599,46400],"quality":"More is better","isMain":true,"metricName":"Total length (>= 1000 bp)"},{"values":[0,0,47993,79327,80678,11540,11770,103260,94407,95222,95936,39674,19127],"quality":"More is better","isMain":false,"metricName":"Total length (>= 5000 bp)"},{"values":[0,0,10597,26070,23208,0,0,44778,45004,38510,39224,12870,0],"quality":"More is better","isMain":true,"metricName":"Total length (>= 10000 bp)"},{"values":[0,0,0,0,0,0,0,0,0,0,0,0,0],"quality":"More is better","isMain":false,"metricName":"Total length (>= 25000 bp)"},{"values":[0,0,0,0,0,0,0,0,0,0,0,0,0],"quality":"More is better","isMain":true,"metricName":"Total length (>= 50000 bp)"},{"values":[1144,960,4099,6137,6068,2621,2049,8667,6712,6668,6668,4432,4347],"quality":"More is better","isMain":false,"metricName":"N50"},{"values":[820,728,2872,3358,4369,1852,1303,6121,4638,4638,4638,2434,3992],"quality":"More is better","isMain":false,"metricName":"N75"},{"values":[22,24,11,8,8,16,17,6,6,7,7,8,4],"quality":"Less is better","isMain":false,"metricName":"L50"},{"values":[45,47,20,15,14,29,34,10,11,12,12,16,7],"quality":"Less is better","isMain":false,"metricName":"L75"},{"values":["54.56","54.76","54.82","54.93","55.19","54.93","54.64","55.02","55.11","54.92","54.88","54.78","54.61"],"quality":"Equal","isMain":false,"metricName":"GC (%)"}]],["Predicted genes",[]],["Similarity statistics",[{"values":[0,0,0,0,0,0,0,0,0,0,0,0,0],"quality":"Equal","isMain":false,"metricName":"# similar correct contigs"},{"values":[0,0,0,0,0,0,0,0,0,0,0,0,0],"quality":"Equal","isMain":false,"metricName":"# similar misassembled blocks"}]],["Reference statistics",[{"values":[131995,131995,131995,131995,131995,131995,131995,131995,131995,131995,131995,131995,131995],"quality":"Equal","isMain":false,"metricName":"Reference length"},{"values":[1,1,1,1,1,1,1,1,1,1,1,1,1],"quality":"Equal","isMain":false,"metricName":"Reference fragments"},{"values":["55.13","55.13","55.13","55.13","55.13","55.13","55.13","55.13","55.13","55.13","55.13","55.13","55.13"],"quality":"Equal","isMain":false,"metricName":"Reference GC (%)"}]]],"referenceName":"gi\_11038431\_ref\_NC","date":"18 July 2018, Wednesday, 16:07:45","order":[0,1,2,3,4,5,6,7,8,9,10,11,12],"assembliesNames":["ABySS\_127","ABySS\_63","CLC","IDBA\_UD","MEGAHIT","MIRA","SOAPdenovo2","SPAdes","SPAdes\_meta","SPAdes\_sc","SPAdes\_sc\_careful","Velvet","Geneious"]},{"assembliesWithNs":null,"minContig":500,"report":[["Genome statistics",[{"values":["18.477","11.254","71.537","62.884","66.211","31.571","66.185","76.773","74.965","75.681","76.727","7.885","7.102"],"quality":"More is better","isMain":true,"metricName":"Genome fraction (%)"},{"values":["1.000","1.000","1.007","1.009","1.000","1.032","1.005","1.003","1.004","1.009","1.005","1.000","1.063"],"quality":"Less is better","isMain":true,"metricName":"Duplication ratio"},{"values":[1466,1682,3647,3406,4353,1783,4182,4737,3647,4737,4737,3161,2884],"quality":"More is better","isMain":true,"metricName":"Largest alignment"},{"values":[18662,11366,72644,63534,66889,32705,67066,77574,76039,76467,77519,7964,7471],"quality":"More is better","isMain":true,"metricName":"Total aligned length"},{"values":[null,null,1093,973,788,null,806,1144,1144,1400,1400,null,null],"quality":"More is better","isMain":false,"metricName":"NG50"},{"values":[null,null,null,null,null,null,null,528,501,652,667,null,null],"quality":"More is better","isMain":false,"metricName":"NG75"},{"values":[631,841,1631,1548,1597,1163,1235,1665,1641,1660,1660,1543,null],"quality":"More is better","isMain":false,"metricName":"NA50"},{"values":[565,696,963,935,783,860,806,935,973,918,852,942,null],"quality":"More is better","isMain":false,"metricName":"NA75"},{"values":[null,null,1093,851,783,null,806,1144,1144,1144,1144,null,null],"quality":"More is better","isMain":true,"metricName":"NGA50"},{"values":[null,null,null,null,null,null,null,528,501,501,529,null,null],"quality":"More is better","isMain":false,"metricName":"NGA75"},{"values":[null,null,26,28,30,null,33,23,25,21,21,null,null],"quality":"Less is better","isMain":false,"metricName":"LG50"},{"values":[null,null,null,null,null,null,null,55,57,49,49,null,null],"quality":"Less is better","isMain":false,"metricName":"LG75"},{"values":[10,6,15,15,14,12,16,15,16,16,16,2,null],"quality":"Less is better","isMain":false,"metricName":"LA50"},{"values":[18,9,30,28,31,20,33,31,31,32,33,4,null],"quality":"Less is better","isMain":false,"metricName":"LA75"},{"values":[null,null,26,30,31,null,33,23,25,23,23,null,null],"quality":"Less is better","isMain":true,"metricName":"LGA50"},{"values":[null,null,null,null,null,null,null,55,57,55,54,null,null],"quality":"Less is better","isMain":false,"metricName":"LGA75"}]],["Misassemblies",[{"values":[0,0,0,0,0,1,0,0,0,0,0,0,0],"quality":"Less is better","isMain":true,"metricName":"# misassemblies"},{"values":[0,0,0,0,0,1,0,0,0,0,0,0,0],"quality":"Less is better","isMain":false,"metricName":" # relocations"},{"values":[0,0,0,0,0,0,0,0,0,0,0,0,0],"quality":"Less is better","isMain":false,"metricName":" # translocations"},{"values":[0,0,0,0,0,0,0,0,0,0,0,0,0],"quality":"Less is better","isMain":false,"metricName":" # inversions"},{"values":[0,0,0,0,0,1,0,0,0,0,0,0,0],"quality":"Less is better","isMain":false,"metricName":"# misassembled contigs"},{"values":[0,0,0,0,0,1183,0,0,0,0,0,0,0],"quality":"Less is better","isMain":true,"metricName":"Misassembled contigs length"},{"values":[0,0,0,0,0,1,0,0,0,0,0,0,1],"quality":"Less is better","isMain":false,"metricName":"# local misassemblies"},{"values":[0,0,0,0,0,0,0,0,0,0,0,0,0],"quality":"Less is better","isMain":false,"metricName":"# unaligned mis. contigs"}]],["Unaligned",[{"values":[0,0,0,0,0,0,0,0,0,0,0,0,0],"quality":"Less is better","isMain":false,"metricName":"# fully unaligned contigs"},{"values":[0,0,0,0,0,0,0,0,0,0,0,0,0],"quality":"Less is better","isMain":false,"metricName":"Fully unaligned length"},{"values":[0,0,0,1,1,0,0,0,0,1,1,0,1],"quality":"Less is better","isMain":false,"metricName":"# partially unaligned contigs"},{"values":[0,0,0,1669,513,0,0,0,0,2600,2600,0,9044],"quality":"Less is better","isMain":false,"metricName":"Partially unaligned length"}]],["Mismatches",[{"values":[5,1,79,67,45,51,38,70,64,66,71,1,30],"quality":"Less is better","isMain":false,"metricName":"# mismatches"},{"values":[0,0,2,1,0,8,2,0,0,2,5,0,0],"quality":"Less is better","isMain":false,"metricName":"# indels"},{"values":[0,0,2,1,0,10,100,0,0,3,12,0,0],"quality":"Less is better","isMain":false,"metricName":"Indels length"},{"values":["26.79","8.80","109.34","105.49","67.29","159.94","56.85","90.28","84.53","86.35","91.62","12.56","418.24"],"quality":"Less is better","isMain":true,"metricName":"# mismatches per 100 kbp"},{"values":["0.00","0.00","2.77","1.57","0.00","25.09","2.99","0.00","0.00","2.62","6.45","0.00","0.00"],"quality":"Less is better","isMain":true,"metricName":"# indels per 100 kbp"},{"values":[0,0,2,1,0,8,0,0,0,2,4,0,0],"quality":"Less is better","isMain":false,"metricName":" # indels (<= 5 bp)"},{"values":[0,0,0,0,0,0,2,0,0,0,1,0,0],"quality":"Less is better","isMain":false,"metricName":" # indels (> 5 bp)"},{"values":[0,0,0,0,0,74,184,0,0,0,0,0,107],"quality":"Less is better","isMain":false,"metricName":"# N's"},{"values":["0.00","0.00","0.00","0.00","0.00","224.77","273.80","0.00","0.00","0.00","0.00","0.00","641.83"],"quality":"Less is better","isMain":true,"metricName":"# N's per 100 kbp"}]],["Statistics without reference",[{"values":[26,14,56,49,56,30,59,58,57,56,57,6,4],"quality":"Equal","isMain":true,"metricName":"# contigs"},{"values":[4,3,27,25,21,17,23,28,30,30,30,3,4],"quality":"Equal","isMain":false,"metricName":"# contigs (>= 1000 bp)"},{"values":[0,0,0,0,0,0,0,0,0,0,0,0,1],"quality":"Equal","isMain":false,"metricName":"# contigs (>= 5000 bp)"},{"values":[0,0,0,0,0,0,0,0,0,0,0,0,0],"quality":"Equal","isMain":false,"metricName":"# contigs (>= 10000 bp)"},{"values":[0,0,0,0,0,0,0,0,0,0,0,0,0],"quality":"Equal","isMain":false,"metricName":"# contigs (>= 25000 bp)"},{"values":[0,0,0,0,0,0,0,0,0,0,0,0,0],"quality":"Equal","isMain":false,"metricName":"# contigs (>= 50000 bp)"},{"values":[1466,1682,3647,3406,4353,1849,4182,4737,3647,4737,4737,3161,9947],"quality":"More is better","isMain":true,"metricName":"Largest contig"},{"values":[18662,11366,72742,65722,67402,32922,67202,77786,76039,79702,80464,7964,16671],"quality":"More is better","isMain":true,"metricName":"Total length"},{"values":[5110,3818,52090,47964,43016,23219,41832,56321,56436,60954,60955,5797,16671],"quality":"More is better","isMain":true,"metricName":"Total length (>= 1000 bp)"},{"values":[0,0,0,0,0,0,0,0,0,0,0,0,9947],"quality":"More is better","isMain":false,"metricName":"Total length (>= 5000 bp)"},{"values":[0,0,0,0,0,0,0,0,0,0,0,0,0],"quality":"More is better","isMain":true,"metricName":"Total length (>= 10000 bp)"},{"values":[0,0,0,0,0,0,0,0,0,0,0,0,0],"quality":"More is better","isMain":false,"metricName":"Total length (>= 25000 bp)"},{"values":[0,0,0,0,0,0,0,0,0,0,0,0,0],"quality":"More is better","isMain":true,"metricName":"Total length (>= 50000 bp)"},{"values":[631,841,1631,1726,1597,1195,1235,1665,1641,1764,1764,1543,9947],"quality":"More is better","isMain":false,"metricName":"N50"},{"values":[565,696,963,974,788,908,806,935,973,1019,1007,942,2884],"quality":"More is better","isMain":false,"metricName":"N75"},{"values":[10,6,15,14,14,11,16,15,16,15,15,2,1],"quality":"Less is better","isMain":false,"metricName":"L50"},{"values":[18,9,30,27,30,19,33,31,31,29,30,4,2],"quality":"Less is better","isMain":false,"metricName":"L75"},{"values":["42.35","40.36","41.90","41.59","41.68","40.40","41.91","41.56","41.87","41.45","41.39","42.09","40.33"],"quality":"Equal","isMain":false,"metricName":"GC (%)"}]],["Predicted genes",[]],["Similarity statistics",[{"values":[0,0,0,0,0,0,0,0,0,0,0,0,0],"quality":"Equal","isMain":false,"metricName":"# similar correct contigs"},{"values":[0,0,0,0,0,0,0,0,0,0,0,0,0],"quality":"Equal","isMain":false,"metricName":"# similar misassembled blocks"}]],["Reference statistics",[{"values":[100999,100999,100999,100999,100999,100999,100999,100999,100999,100999,100999,100999,100999],"quality":"Equal","isMain":false,"metricName":"Reference length"},{"values":[1,1,1,1,1,1,1,1,1,1,1,1,1],"quality":"Equal","isMain":false,"metricName":"Reference fragments"},{"values":["40.69","40.69","40.69","40.69","40.69","40.69","40.69","40.69","40.69","40.69","40.69","40.69","40.69"],"quality":"Equal","isMain":false,"metricName":"Reference GC (%)"}]]],"referenceName":"gi\_11068003\_ref\_NC","date":"18 July 2018, Wednesday, 16:07:56","order":[0,1,2,3,4,5,6,7,8,9,10,11,12],"assembliesNames":["ABySS\_127","ABySS\_63","CLC","IDBA\_UD","MEGAHIT","MIRA","SOAPdenovo2","SPAdes","SPAdes\_meta","SPAdes\_sc","SPAdes\_sc\_careful","Velvet","Geneious"]},{"assembliesWithNs":null,"minContig":500,"report":[["Genome statistics",[{"values":["99.957","100.000","100.000","100.000","81.416","72.484","100.000","100.000","100.000","100.000","100.000"],"quality":"More is better","isMain":true,"metricName":"Genome fraction (%)"},{"values":["1.000","1.002","1.003","1.009","1.026","1.001","1.003","1.001","1.001","1.001","1.001"],"quality":"Less is better","isMain":true,"metricName":"Duplication ratio"},{"values":[43991,44109,44151,44402,12878,2436,44137,44065,44065,44065,39780],"quality":"More is better","isMain":true,"metricName":"Largest alignment"},{"values":[43991,44109,44151,44402,36747,31921,44137,44065,44065,44065,44061],"quality":"More is better","isMain":true,"metricName":"Total aligned length"},{"values":[44010,44109,44151,44402,7432,750,44137,44065,44065,44065,39780],"quality":"More is better","isMain":false,"metricName":"NG50"},{"values":[44010,44109,44151,44402,4051,null,44137,44065,44065,44065,39780],"quality":"More is better","isMain":false,"metricName":"NG75"},{"values":[43991,44109,44151,44402,9120,1219,44137,44065,44065,44065,39780],"quality":"More is better","isMain":false,"metricName":"NA50"},{"values":[43991,44109,44151,44402,7432,706,44137,44065,44065,44065,39780],"quality":"More is better","isMain":false,"metricName":"NA75"},{"values":[43991,44109,44151,44402,7432,750,44137,44065,44065,44065,39780],"quality":"More is better","isMain":true,"metricName":"NGA50"},{"values":[43991,44109,44151,44402,4051,null,44137,44065,44065,44065,39780],"quality":"More is better","isMain":false,"metricName":"NGA75"},{"values":[1,1,1,1,3,18,1,1,1,1,1],"quality":"Less is better","isMain":false,"metricName":"LG50"},{"values":[1,1,1,1,4,null,1,1,1,1,1],"quality":"Less is better","isMain":false,"metricName":"LG75"},{"values":[1,1,1,1,2,11,1,1,1,1,1],"quality":"Less is better","isMain":false,"metricName":"LA50"},{"values":[1,1,1,1,3,21,1,1,1,1,1],"quality":"Less is better","isMain":false,"metricName":"LA75"},{"values":[1,1,1,1,3,18,1,1,1,1,1],"quality":"Less is better","isMain":true,"metricName":"LGA50"},{"values":[1,1,1,1,4,null,1,1,1,1,1],"quality":"Less is better","isMain":false,"metricName":"LGA75"}]],["Misassemblies",[{"values":[0,0,0,0,0,0,0,0,0,0,0],"quality":"Less is better","isMain":true,"metricName":"# misassemblies"},{"values":[0,0,0,0,0,0,0,0,0,0,0],"quality":"Less is better","isMain":false,"metricName":" # relocations"},{"values":[0,0,0,0,0,0,0,0,0,0,0],"quality":"Less is better","isMain":false,"metricName":" # translocations"},{"values":[0,0,0,0,0,0,0,0,0,0,0],"quality":"Less is better","isMain":false,"metricName":" # inversions"},{"values":[0,0,0,0,0,0,0,0,0,0,0],"quality":"Less is better","isMain":false,"metricName":"# misassembled contigs"},{"values":[0,0,0,0,0,0,0,0,0,0,0],"quality":"Less is better","isMain":true,"metricName":"Misassembled contigs length"},{"values":[0,1,1,1,0,0,1,0,0,0,0],"quality":"Less is better","isMain":false,"metricName":"# local misassemblies"},{"values":[0,0,0,0,0,0,0,0,0,0,0],"quality":"Less is better","isMain":false,"metricName":"# unaligned mis. contigs"}]],["Unaligned",[{"values":[0,0,0,0,0,0,0,0,0,0,0],"quality":"Less is better","isMain":false,"metricName":"# fully unaligned contigs"},{"values":[0,0,0,0,0,0,0,0,0,0,0],"quality":"Less is better","isMain":false,"metricName":"Fully unaligned length"},{"values":[0,0,0,0,0,0,0,0,0,0,0],"quality":"Less is better","isMain":false,"metricName":"# partially unaligned contigs"},{"values":[0,0,0,0,0,0,0,0,0,0,0],"quality":"Less is better","isMain":false,"metricName":"Partially unaligned length"}]],["Mismatches",[{"values":[0,0,0,0,0,2,0,0,0,0,0],"quality":"Less is better","isMain":false,"metricName":"# mismatches"},{"values":[0,0,0,0,0,0,0,1,1,1,0],"quality":"Less is better","isMain":false,"metricName":"# indels"},{"values":[0,0,0,0,0,0,0,55,55,55,0],"quality":"Less is better","isMain":false,"metricName":"Indels length"},{"values":["0.00","0.00","0.00","0.00","0.00","6.27","0.00","0.00","0.00","0.00","0.00"],"quality":"Less is better","isMain":true,"metricName":"# mismatches per 100 kbp"},{"values":["0.00","0.00","0.00","0.00","0.00","0.00","0.00","2.27","2.27","2.27","0.00"],"quality":"Less is better","isMain":true,"metricName":"# indels per 100 kbp"},{"values":[0,0,0,0,0,0,0,0,0,0,0],"quality":"Less is better","isMain":false,"metricName":" # indels (<= 5 bp)"},{"values":[0,0,0,0,0,0,0,1,1,1,0],"quality":"Less is better","isMain":false,"metricName":" # indels (> 5 bp)"},{"values":[0,0,0,0,0,0,0,0,0,0,0],"quality":"Less is better","isMain":false,"metricName":"# N's"},{"values":["0.00","0.00","0.00","0.00","0.00","0.00","0.00","0.00","0.00","0.00","0.00"],"quality":"Less is better","isMain":true,"metricName":"# N's per 100 kbp"}]],["Statistics without reference",[{"values":[1,1,1,1,5,33,1,1,1,1,2],"quality":"Equal","isMain":true,"metricName":"# contigs"},{"values":[1,1,1,1,5,12,1,1,1,1,2],"quality":"Equal","isMain":false,"metricName":"# contigs (>= 1000 bp)"},{"values":[1,1,1,1,3,0,1,1,1,1,1],"quality":"Equal","isMain":false,"metricName":"# contigs (>= 5000 bp)"},{"values":[1,1,1,1,1,0,1,1,1,1,1],"quality":"Equal","isMain":false,"metricName":"# contigs (>= 10000 bp)"},{"values":[1,1,1,1,0,0,1,1,1,1,1],"quality":"Equal","isMain":false,"metricName":"# contigs (>= 25000 bp)"},{"values":[0,0,0,0,0,0,0,0,0,0,0],"quality":"Equal","isMain":false,"metricName":"# contigs (>= 50000 bp)"},{"values":[44010,44109,44151,44402,12878,2436,44137,44065,44065,44065,39780],"quality":"More is better","isMain":true,"metricName":"Largest contig"},{"values":[44010,44109,44151,44402,36747,31921,44137,44065,44065,44065,44061],"quality":"More is better","isMain":true,"metricName":"Total length"},{"values":[44010,44109,44151,44402,36747,17212,44137,44065,44065,44065,44061],"quality":"More is better","isMain":true,"metricName":"Total length (>= 1000 bp)"},{"values":[44010,44109,44151,44402,29430,0,44137,44065,44065,44065,39780],"quality":"More is better","isMain":false,"metricName":"Total length (>= 5000 bp)"},{"values":[44010,44109,44151,44402,12878,0,44137,44065,44065,44065,39780],"quality":"More is better","isMain":true,"metricName":"Total length (>= 10000 bp)"},{"values":[44010,44109,44151,44402,0,0,44137,44065,44065,44065,39780],"quality":"More is better","isMain":false,"metricName":"Total length (>= 25000 bp)"},{"values":[0,0,0,0,0,0,0,0,0,0,0],"quality":"More is better","isMain":true,"metricName":"Total length (>= 50000 bp)"},{"values":[44010,44109,44151,44402,9120,1219,44137,44065,44065,44065,39780],"quality":"More is better","isMain":false,"metricName":"N50"},{"values":[44010,44109,44151,44402,7432,706,44137,44065,44065,44065,39780],"quality":"More is better","isMain":false,"metricName":"N75"},{"values":[1,1,1,1,2,11,1,1,1,1,1],"quality":"Less is better","isMain":false,"metricName":"L50"},{"values":[1,1,1,1,3,21,1,1,1,1,1],"quality":"Less is better","isMain":false,"metricName":"L75"},{"values":["43.73","43.72","43.71","43.68","43.79","43.86","43.73","43.74","43.74","43.74","43.71"],"quality":"Equal","isMain":false,"metricName":"GC (%)"}]],["Predicted genes",[]],["Similarity statistics",[{"values":[0,0,0,0,0,0,0,0,0,0,0],"quality":"Equal","isMain":false,"metricName":"# similar correct contigs"},{"values":[0,0,0,0,0,0,0,0,0,0,0],"quality":"Equal","isMain":false,"metricName":"# similar misassembled blocks"}]],["Reference statistics",[{"values":[44010,44010,44010,44010,44010,44010,44010,44010,44010,44010,44010],"quality":"Equal","isMain":false,"metricName":"Reference length"},{"values":[1,1,1,1,1,1,1,1,1,1,1],"quality":"Equal","isMain":false,"metricName":"Reference fragments"},{"values":["43.72","43.72","43.72","43.72","43.72","43.72","43.72","43.72","43.72","43.72","43.72"],"quality":"Equal","isMain":false,"metricName":"Reference GC (%)"}]]],"referenceName":"gi\_111146921\_ref\_NC","date":"18 July 2018, Wednesday, 16:08:06","order":[0,1,2,3,4,5,6,7,8,9,10],"assembliesNames":["CLC","IDBA\_UD","MEGAHIT","MIRA","Ray\_Meta","SOAPdenovo2","SPAdes","SPAdes\_meta","SPAdes\_sc","SPAdes\_sc\_careful","Velvet"]},{"assembliesWithNs":null,"minContig":500,"report":[["Genome statistics",[{"values":["46.803","38.990","74.793","89.047","88.046","79.467","40.442","84.962","88.080","86.513","78.610","54.854","36.528"],"quality":"More is better","isMain":true,"metricName":"Genome fraction (%)"},{"values":["1.004","1.005","1.042","1.008","1.035","1.045","1.062","1.005","1.012","1.014","1.014","1.002","1.004"],"quality":"Less is better","isMain":true,"metricName":"Duplication ratio"},{"values":[3429,3422,5855,10014,7581,5869,2180,15821,14710,15405,11808,5276,10963],"quality":"More is better","isMain":true,"metricName":"Largest alignment"},{"values":[92950,77553,154157,177222,179942,164423,80652,168612,175822,172514,156664,108592,72256],"quality":"More is better","isMain":true,"metricName":"Total aligned length"},{"values":[null,null,2231,4397,2616,2356,null,5495,4862,3194,2048,654,null],"quality":"More is better","isMain":false,"metricName":"NG50"},{"values":[null,null,1013,2113,1515,992,null,2570,2290,1547,692,null,null],"quality":"More is better","isMain":false,"metricName":"NG75"},{"values":[971,828,1846,4541,2768,2581,811,6932,6933,3467,2571,1656,5660],"quality":"More is better","isMain":false,"metricName":"NA50"},{"values":[702,677,700,2321,1811,1725,611,3467,2576,2048,1498,983,3986],"quality":"More is better","isMain":false,"metricName":"NA75"},{"values":[null,null,1777,4397,2505,2356,null,5487,4844,3188,2048,651,null],"quality":"More is better","isMain":true,"metricName":"NGA50"},{"values":[null,null,578,1996,1515,992,null,2392,2057,1173,668,null,null],"quality":"More is better","isMain":false,"metricName":"NGA75"},{"values":[null,null,21,15,23,30,null,11,11,17,26,65,null],"quality":"Less is better","isMain":false,"metricName":"LG50"},{"values":[null,null,51,33,47,61,null,24,27,37,68,null,null],"quality":"Less is better","isMain":false,"metricName":"LG75"},{"values":[33,32,31,13,20,23,39,9,9,15,17,23,5],"quality":"Less is better","isMain":false,"metricName":"LA50"},{"values":[61,58,69,27,40,42,73,18,21,31,37,44,8],"quality":"Less is better","isMain":false,"metricName":"LA75"},{"values":[null,null,35,15,23,30,null,11,11,18,26,66,null],"quality":"Less is better","isMain":true,"metricName":"LGA50"},{"values":[null,null,89,34,47,61,null,25,27,41,70,null,null],"quality":"Less is better","isMain":false,"metricName":"LGA75"}]],["Misassemblies",[{"values":[0,0,0,0,0,0,0,0,0,0,0,0,0],"quality":"Less is better","isMain":true,"metricName":"# misassemblies"},{"values":[0,0,0,0,0,0,0,0,0,0,0,0,0],"quality":"Less is better","isMain":false,"metricName":" # relocations"},{"values":[0,0,0,0,0,0,0,0,0,0,0,0,0],"quality":"Less is better","isMain":false,"metricName":" # translocations"},{"values":[0,0,0,0,0,0,0,0,0,0,0,0,0],"quality":"Less is better","isMain":false,"metricName":" # inversions"},{"values":[0,0,0,0,0,0,0,0,0,0,0,0,0],"quality":"Less is better","isMain":false,"metricName":"# misassembled contigs"},{"values":[0,0,0,0,0,0,0,0,0,0,0,0,0],"quality":"Less is better","isMain":true,"metricName":"Misassembled contigs length"},{"values":[0,0,1,1,1,0,0,1,1,2,1,1,0],"quality":"Less is better","isMain":false,"metricName":"# local misassemblies"},{"values":[0,0,1,0,0,0,1,0,0,0,0,0,0],"quality":"Less is better","isMain":false,"metricName":"# unaligned mis. contigs"}]],["Unaligned",[{"values":[0,0,0,0,0,0,0,0,0,0,0,0,0],"quality":"Less is better","isMain":false,"metricName":"# fully unaligned contigs"},{"values":[0,0,0,0,0,0,0,0,0,0,0,0,0],"quality":"Less is better","isMain":false,"metricName":"Fully unaligned length"},{"values":[0,0,2,1,1,0,5,2,1,2,0,0,0],"quality":"Less is better","isMain":false,"metricName":"# partially unaligned contigs"},{"values":[0,0,26563,861,786,0,6824,1847,1061,3193,0,0,0],"quality":"Less is better","isMain":false,"metricName":"Partially unaligned length"}]],["Mismatches",[{"values":[58,43,570,424,539,284,127,541,588,538,447,153,162],"quality":"Less is better","isMain":false,"metricName":"# mismatches"},{"values":[3,0,87,42,59,44,36,54,64,61,47,5,27],"quality":"Less is better","isMain":false,"metricName":"# indels"},{"values":[8,0,315,124,187,139,1665,137,194,202,155,80,183],"quality":"Less is better","isMain":false,"metricName":"Indels length"},{"values":["62.57","55.69","384.80","240.42","309.10","180.45","158.56","321.51","337.07","314.00","287.11","140.83","223.93"],"quality":"Less is better","isMain":true,"metricName":"# mismatches per 100 kbp"},{"values":["3.24","0.00","58.73","23.82","33.83","27.96","44.95","32.09","36.69","35.60","30.19","4.60","37.32"],"quality":"Less is better","isMain":true,"metricName":"# indels per 100 kbp"},{"values":[3,0,78,38,54,38,1,51,58,54,42,3,21],"quality":"Less is better","isMain":false,"metricName":" # indels (<= 5 bp)"},{"values":[0,0,9,4,5,6,35,3,6,7,5,2,6],"quality":"Less is better","isMain":false,"metricName":" # indels (> 5 bp)"},{"values":[0,0,0,0,0,514,5683,0,422,1371,730,40,421],"quality":"Less is better","isMain":false,"metricName":"# N's"},{"values":["0.00","0.00","0.00","0.00","0.00","312.48","6182.21","0.00","237.73","774.97","462.39","36.74","579.90"],"quality":"Less is better","isMain":true,"metricName":"# N's per 100 kbp"}]],["Statistics without reference",[{"values":[99,91,98,59,80,83,98,37,48,71,83,82,16],"quality":"Equal","isMain":true,"metricName":"# contigs"},{"values":[32,20,51,49,60,59,28,36,41,48,49,44,16],"quality":"Equal","isMain":false,"metricName":"# contigs (>= 1000 bp)"},{"values":[0,0,5,11,6,2,0,12,10,8,6,1,7],"quality":"Equal","isMain":false,"metricName":"# contigs (>= 5000 bp)"},{"values":[0,0,1,1,0,0,0,4,4,1,1,0,1],"quality":"Equal","isMain":false,"metricName":"# contigs (>= 10000 bp)"},{"values":[0,0,1,0,0,0,0,0,0,0,0,0,0],"quality":"Equal","isMain":false,"metricName":"# contigs (>= 25000 bp)"},{"values":[0,0,0,0,0,0,0,0,0,0,0,0,0],"quality":"Equal","isMain":false,"metricName":"# contigs (>= 50000 bp)"},{"values":[3429,3422,30159,10014,7581,5869,3228,15821,14710,15405,11808,5276,10963],"quality":"More is better","isMain":true,"metricName":"Largest contig"},{"values":[93031,77587,180960,178713,181248,164488,91925,170962,177514,176909,157876,108859,72599],"quality":"More is better","isMain":true,"metricName":"Total length"},{"values":[46126,28653,148981,170826,166023,146727,42302,170346,172102,161281,133587,82361,72599],"quality":"More is better","isMain":true,"metricName":"Total length (>= 1000 bp)"},{"values":[0,0,52425,82427,39575,11050,0,107808,96297,66553,43343,5276,51070],"quality":"More is better","isMain":false,"metricName":"Total length (>= 5000 bp)"},{"values":[0,0,30159,10014,0,0,0,48840,49362,15405,11808,0,10963],"quality":"More is better","isMain":true,"metricName":"Total length (>= 10000 bp)"},{"values":[0,0,30159,0,0,0,0,0,0,0,0,0,0],"quality":"More is better","isMain":false,"metricName":"Total length (>= 25000 bp)"},{"values":[0,0,0,0,0,0,0,0,0,0,0,0,0],"quality":"More is better","isMain":true,"metricName":"Total length (>= 50000 bp)"},{"values":[971,828,2584,4541,2845,2581,914,6932,6933,3602,2724,1656,5660],"quality":"More is better","isMain":false,"metricName":"N50"},{"values":[702,689,1471,2321,1869,1725,708,3470,2739,2127,1498,1014,3986],"quality":"More is better","isMain":false,"metricName":"N75"},{"values":[33,32,17,13,20,23,32,9,9,14,17,23,5],"quality":"Less is better","isMain":false,"metricName":"L50"},{"values":[61,58,41,27,39,42,61,18,21,29,37,44,8],"quality":"Less is better","isMain":false,"metricName":"L75"},{"values":["33.31","33.49","32.42","33.16","33.29","33.22","33.18","33.27","33.31","33.20","33.25","33.15","33.29"],"quality":"Equal","isMain":false,"metricName":"GC (%)"}]],["Predicted genes",[]],["Similarity statistics",[{"values":[0,0,0,0,0,0,0,0,0,0,0,0,0],"quality":"Equal","isMain":false,"metricName":"# similar correct contigs"},{"values":[0,0,0,0,0,0,0,0,0,0,0,0,0],"quality":"Equal","isMain":false,"metricName":"# similar misassembled blocks"}]],["Reference statistics",[{"values":[198050,198050,198050,198050,198050,198050,198050,198050,198050,198050,198050,198050,198050],"quality":"Equal","isMain":false,"metricName":"Reference length"},{"values":[1,1,1,1,1,1,1,1,1,1,1,1,1],"quality":"Equal","isMain":false,"metricName":"Reference fragments"},{"values":["33.26","33.26","33.26","33.26","33.26","33.26","33.26","33.26","33.26","33.26","33.26","33.26","33.26"],"quality":"Equal","isMain":false,"metricName":"Reference GC (%)"}]]],"referenceName":"gi\_113195177\_ref\_NC","date":"18 July 2018, Wednesday, 16:08:18","order":[0,1,2,3,4,5,6,7,8,9,10,11,12],"assembliesNames":["ABySS\_127","ABySS\_63","CLC","IDBA\_UD","MEGAHIT","MIRA","SOAPdenovo2","SPAdes","SPAdes\_meta","SPAdes\_sc","SPAdes\_sc\_careful","Velvet","Geneious"]},{"assembliesWithNs":null,"minContig":500,"report":[["Genome statistics",[{"values":["18.954","12.201","76.896","71.552","76.463","41.590","69.176","83.007","83.825","83.656","83.788","9.004","1.112"],"quality":"More is better","isMain":true,"metricName":"Genome fraction (%)"},{"values":["1.001","1.001","1.018","1.004","1.001","1.005","1.004","1.004","1.001","1.003","1.004","1.000","1.000"],"quality":"Less is better","isMain":true,"metricName":"Duplication ratio"},{"values":[1309,1117,5124,4865,4799,2919,5061,5124,5124,5124,5124,3008,1868],"quality":"More is better","isMain":true,"metricName":"Largest alignment"},{"values":[31872,20518,130706,120379,128559,70248,116455,139525,141015,140601,140923,15130,1868],"quality":"More is better","isMain":true,"metricName":"Total aligned length"},{"values":[null,null,1259,1321,1360,null,994,1831,1653,1787,1882,null,null],"quality":"More is better","isMain":false,"metricName":"NG50"},{"values":[null,null,667,566,554,null,null,905,832,891,905,null,null],"quality":"More is better","isMain":false,"metricName":"NG75"},{"values":[713,743,1460,1699,1799,1199,1409,1914,1983,1945,1983,1667,1868],"quality":"More is better","isMain":false,"metricName":"NA50"},{"values":[587,631,868,1039,1031,794,891,1113,1255,1210,1210,1072,1868],"quality":"More is better","isMain":false,"metricName":"NA75"},{"values":[null,null,1121,1231,1289,null,966,1653,1653,1667,1667,null,null],"quality":"More is better","isMain":true,"metricName":"NGA50"},{"values":[null,null,572,null,554,null,null,828,832,832,854,null,null],"quality":"More is better","isMain":false,"metricName":"NGA75"},{"values":[null,null,34,34,37,null,47,29,31,30,30,null,null],"quality":"Less is better","isMain":false,"metricName":"LG50"},{"values":[null,null,81,80,87,null,null,61,67,63,61,null,null],"quality":"Less is better","isMain":false,"metricName":"LG75"},{"values":[18,12,25,25,25,22,26,25,24,25,25,4,1],"quality":"Less is better","isMain":false,"metricName":"LA50"},{"values":[30,19,56,49,48,40,51,50,47,49,49,7,1],"quality":"Less is better","isMain":false,"metricName":"LA75"},{"values":[null,null,38,39,38,null,48,32,31,31,31,null,null],"quality":"Less is better","isMain":true,"metricName":"LGA50"},{"values":[null,null,90,null,87,null,null,68,67,67,66,null,null],"quality":"Less is better","isMain":false,"metricName":"LGA75"}]],["Misassemblies",[{"values":[0,0,0,0,1,0,0,0,0,0,0,0,0],"quality":"Less is better","isMain":true,"metricName":"# misassemblies"},{"values":[0,0,0,0,1,0,0,0,0,0,0,0,0],"quality":"Less is better","isMain":false,"metricName":" # relocations"},{"values":[0,0,0,0,0,0,0,0,0,0,0,0,0],"quality":"Less is better","isMain":false,"metricName":" # translocations"},{"values":[0,0,0,0,0,0,0,0,0,0,0,0,0],"quality":"Less is better","isMain":false,"metricName":" # inversions"},{"values":[0,0,0,0,1,0,0,0,0,0,0,0,0],"quality":"Less is better","isMain":false,"metricName":"# misassembled contigs"},{"values":[0,0,0,0,1648,0,0,0,0,0,0,0,0],"quality":"Less is better","isMain":true,"metricName":"Misassembled contigs length"},{"values":[0,0,0,0,0,0,0,0,0,0,0,0,0],"quality":"Less is better","isMain":false,"metricName":"# local misassemblies"},{"values":[0,0,0,0,0,0,0,0,0,0,0,0,0],"quality":"Less is better","isMain":false,"metricName":"# unaligned mis. contigs"}]],["Unaligned",[{"values":[0,0,0,0,0,0,0,0,0,0,0,0,0],"quality":"Less is better","isMain":false,"metricName":"# fully unaligned contigs"},{"values":[0,0,0,0,0,0,0,0,0,0,0,0,0],"quality":"Less is better","isMain":false,"metricName":"Fully unaligned length"},{"values":[0,0,3,2,0,0,0,3,0,3,4,0,0],"quality":"Less is better","isMain":false,"metricName":"# partially unaligned contigs"},{"values":[0,0,4426,6381,0,0,0,5228,0,3361,4612,0,0],"quality":"Less is better","isMain":false,"metricName":"Partially unaligned length"}]],["Mismatches",[{"values":[5,3,180,198,96,21,92,180,134,146,165,4,0],"quality":"Less is better","isMain":false,"metricName":"# mismatches"},{"values":[0,0,2,2,0,0,4,2,0,2,4,0,0],"quality":"Less is better","isMain":false,"metricName":"# indels"},{"values":[0,0,2,2,0,0,183,3,0,3,5,0,0],"quality":"Less is better","isMain":false,"metricName":"Indels length"},{"values":["15.70","14.63","139.30","164.67","74.71","30.05","79.14","129.05","95.13","103.86","117.19","26.44","0.00"],"quality":"Less is better","isMain":true,"metricName":"# mismatches per 100 kbp"},{"values":["0.00","0.00","1.55","1.66","0.00","0.00","3.44","1.43","0.00","1.42","2.84","0.00","0.00"],"quality":"Less is better","isMain":true,"metricName":"# indels per 100 kbp"},{"values":[0,0,2,2,0,0,0,2,0,2,4,0,0],"quality":"Less is better","isMain":false,"metricName":" # indels (<= 5 bp)"},{"values":[0,0,0,0,0,0,4,0,0,0,0,0,0],"quality":"Less is better","isMain":false,"metricName":" # indels (> 5 bp)"},{"values":[0,0,0,0,0,94,383,0,0,0,0,0,3],"quality":"Less is better","isMain":false,"metricName":"# N's"},{"values":["0.00","0.00","0.00","0.00","0.00","133.81","328.16","0.00","0.00","0.00","0.00","0.00","160.60"],"quality":"Less is better","isMain":true,"metricName":"# N's per 100 kbp"}]],["Statistics without reference",[{"values":[44,28,98,81,91,67,93,87,88,87,87,12,1],"quality":"Equal","isMain":true,"metricName":"# contigs"},{"values":[7,2,47,51,49,27,46,56,56,57,57,7,1],"quality":"Equal","isMain":false,"metricName":"# contigs (>= 1000 bp)"},{"values":[0,0,3,1,0,0,1,3,2,3,3,0,0],"quality":"Equal","isMain":false,"metricName":"# contigs (>= 5000 bp)"},{"values":[0,0,0,0,0,0,0,0,0,0,0,0,0],"quality":"Equal","isMain":false,"metricName":"# contigs (>= 10000 bp)"},{"values":[0,0,0,0,0,0,0,0,0,0,0,0,0],"quality":"Equal","isMain":false,"metricName":"# contigs (>= 25000 bp)"},{"values":[0,0,0,0,0,0,0,0,0,0,0,0,0],"quality":"Equal","isMain":false,"metricName":"# contigs (>= 50000 bp)"},{"values":[1309,1117,5225,8204,4799,2919,5061,5225,5124,5225,5225,3008,1868],"quality":"More is better","isMain":true,"metricName":"Largest contig"},{"values":[31872,20518,135921,127072,128559,70248,116713,145300,141015,144344,145917,15130,1868],"quality":"More is better","isMain":true,"metricName":"Total length"},{"values":[8011,2146,98622,103951,98042,41693,83162,121415,116728,121219,122792,12140,1868],"quality":"More is better","isMain":true,"metricName":"Total length (>= 1000 bp)"},{"values":[0,0,15410,8204,0,0,5061,15410,10185,15410,15410,0,0],"quality":"More is better","isMain":false,"metricName":"Total length (>= 5000 bp)"},{"values":[0,0,0,0,0,0,0,0,0,0,0,0,0],"quality":"More is better","isMain":true,"metricName":"Total length (>= 10000 bp)"},{"values":[0,0,0,0,0,0,0,0,0,0,0,0,0],"quality":"More is better","isMain":false,"metricName":"Total length (>= 25000 bp)"},{"values":[0,0,0,0,0,0,0,0,0,0,0,0,0],"quality":"More is better","isMain":true,"metricName":"Total length (>= 50000 bp)"},{"values":[713,743,1653,1891,1799,1199,1409,2025,1983,2007,2025,1667,1868],"quality":"More is better","isMain":false,"metricName":"N50"},{"values":[587,631,928,1135,1031,794,891,1259,1255,1311,1311,1072,1868],"quality":"More is better","isMain":false,"metricName":"N75"},{"values":[18,12,23,21,25,22,26,23,24,24,24,4,1],"quality":"Less is better","isMain":false,"metricName":"L50"},{"values":[30,19,51,43,48,40,51,46,47,46,46,7,1],"quality":"Less is better","isMain":false,"metricName":"L75"},{"values":["48.14","47.70","48.49","48.76","48.57","48.58","48.42","48.62","48.52","48.45","48.51","48.29","45.36"],"quality":"Equal","isMain":false,"metricName":"GC (%)"}]],["Predicted genes",[]],["Similarity statistics",[{"values":[0,0,0,0,0,0,0,0,0,0,0,0,0],"quality":"Equal","isMain":false,"metricName":"# similar correct contigs"},{"values":[0,0,0,0,0,0,0,0,0,0,0,0,0],"quality":"Equal","isMain":false,"metricName":"# similar misassembled blocks"}]],["Reference statistics",[{"values":[168041,168041,168041,168041,168041,168041,168041,168041,168041,168041,168041,168041,168041],"quality":"Equal","isMain":false,"metricName":"Reference length"},{"values":[1,1,1,1,1,1,1,1,1,1,1,1,1],"quality":"Equal","isMain":false,"metricName":"Reference fragments"},{"values":["48.57","48.57","48.57","48.57","48.57","48.57","48.57","48.57","48.57","48.57","48.57","48.57","48.57"],"quality":"Equal","isMain":false,"metricName":"Reference GC (%)"}]]],"referenceName":"gi\_114679849\_ref\_NC","date":"18 July 2018, Wednesday, 16:08:30","order":[0,1,2,3,4,5,6,7,8,9,10,11,12],"assembliesNames":["ABySS\_127","ABySS\_63","CLC","IDBA\_UD","MEGAHIT","MIRA","SOAPdenovo2","SPAdes","SPAdes\_meta","SPAdes\_sc","SPAdes\_sc\_careful","Velvet","Geneious"]},{"assembliesWithNs":null,"minContig":500,"report":[["Genome statistics",[{"values":["20.269","14.013","19.970","2.629","7.303","22.118","18.030","21.348","21.014"],"quality":"More is better","isMain":true,"metricName":"Genome fraction (%)"},{"values":["1.083","1.008","1.002","1.000","1.001","1.020","1.019","1.031","1.031"],"quality":"Less is better","isMain":true,"metricName":"Duplication ratio"},{"values":[1629,1552,1516,1008,1497,1687,1687,2247,2247],"quality":"More is better","isMain":true,"metricName":"Largest alignment"},{"values":[28952,18857,26874,3534,9826,29739,24248,28693,28243],"quality":"More is better","isMain":true,"metricName":"Total aligned length"},{"values":[1041,844,1050,987,888,1041,860,1126,1123],"quality":"More is better","isMain":false,"metricName":"NA50"},{"values":[687,621,728,810,630,809,654,781,781],"quality":"More is better","isMain":false,"metricName":"NA75"},{"values":[null,null,null,null,null,null,null,null,null],"quality":"More is better","isMain":true,"metricName":"NGA50"},{"values":[12,9,11,2,5,12,12,11,11],"quality":"Less is better","isMain":false,"metricName":"LA50"},{"values":[21,16,19,3,8,21,19,19,19],"quality":"Less is better","isMain":false,"metricName":"LA75"}]],["Misassemblies",[{"values":[1,0,0,0,0,0,0,0,0],"quality":"Less is better","isMain":true,"metricName":"# misassemblies"},{"values":[1,0,0,0,0,0,0,0,0],"quality":"Less is better","isMain":false,"metricName":" # relocations"},{"values":[0,0,0,0,0,0,0,0,0],"quality":"Less is better","isMain":false,"metricName":" # translocations"},{"values":[0,0,0,0,0,0,0,0,0],"quality":"Less is better","isMain":false,"metricName":" # inversions"},{"values":[1,0,0,0,0,0,0,0,0],"quality":"Less is better","isMain":false,"metricName":"# misassembled contigs"},{"values":[723,0,0,0,0,0,0,0,0],"quality":"Less is better","isMain":true,"metricName":"Misassembled contigs length"},{"values":[0,0,0,0,0,1,0,1,1],"quality":"Less is better","isMain":false,"metricName":"# local misassemblies"},{"values":[0,0,0,0,0,0,0,0,0],"quality":"Less is better","isMain":false,"metricName":"# unaligned mis. contigs"}]],["Unaligned",[{"values":[0,0,0,0,0,0,0,0,0],"quality":"Less is better","isMain":false,"metricName":"# fully unaligned contigs"},{"values":[0,0,0,0,0,0,0,0,0],"quality":"Less is better","isMain":false,"metricName":"Fully unaligned length"},{"values":[1,1,0,0,0,1,0,1,1],"quality":"Less is better","isMain":false,"metricName":"# partially unaligned contigs"},{"values":[698,620,0,0,0,755,0,1565,1565],"quality":"Less is better","isMain":false,"metricName":"Partially unaligned length"}]],["Mismatches",[{"values":[354,189,343,12,114,422,184,388,372],"quality":"Less is better","isMain":false,"metricName":"# mismatches"},{"values":[9,8,19,0,2,19,5,19,19],"quality":"Less is better","isMain":false,"metricName":"# indels"},{"values":[31,31,47,0,9,62,18,57,57],"quality":"Less is better","isMain":false,"metricName":"Indels length"},{"values":["1299.32","1003.40","1277.80","339.56","1161.25","1419.39","759.20","1352.10","1317.00"],"quality":"Less is better","isMain":true,"metricName":"# mismatches per 100 kbp"},{"values":["33.03","42.47","70.78","0.00","20.37","63.91","20.63","66.21","67.27"],"quality":"Less is better","isMain":true,"metricName":"# indels per 100 kbp"},{"values":[7,6,17,0,1,16,3,16,16],"quality":"Less is better","isMain":false,"metricName":" # indels (<= 5 bp)"},{"values":[2,2,2,0,1,3,2,3,3],"quality":"Less is better","isMain":false,"metricName":" # indels (> 5 bp)"},{"values":[0,0,0,28,0,0,0,0,0],"quality":"Less is better","isMain":false,"metricName":"# N's"},{"values":["0.00","0.00","0.00","792.30","0.00","0.00","0.00","0.00","0.00"],"quality":"Less is better","isMain":true,"metricName":"# N's per 100 kbp"}]],["Statistics without reference",[{"values":[32,23,30,4,12,30,29,27,27],"quality":"Equal","isMain":true,"metricName":"# contigs"},{"values":[12,6,12,1,2,14,6,17,16],"quality":"Equal","isMain":false,"metricName":"# contigs (>= 1000 bp)"},{"values":[0,0,0,0,0,0,0,0,0],"quality":"Equal","isMain":false,"metricName":"# contigs (>= 5000 bp)"},{"values":[0,0,0,0,0,0,0,0,0],"quality":"Equal","isMain":false,"metricName":"# contigs (>= 10000 bp)"},{"values":[0,0,0,0,0,0,0,0,0],"quality":"Equal","isMain":false,"metricName":"# contigs (>= 25000 bp)"},{"values":[0,0,0,0,0,0,0,0,0],"quality":"Equal","isMain":false,"metricName":"# contigs (>= 50000 bp)"},{"values":[1629,1552,1516,1008,1497,1846,1687,2247,2247],"quality":"More is better","isMain":true,"metricName":"Largest contig"},{"values":[30194,19604,26884,3534,9826,31080,24688,31143,30693],"quality":"More is better","isMain":true,"metricName":"Total length"},{"values":[15571,7353,14634,1008,2806,18990,7611,23762,22460],"quality":"More is better","isMain":true,"metricName":"Total length (>= 1000 bp)"},{"values":[0,0,0,0,0,0,0,0,0],"quality":"More is better","isMain":false,"metricName":"Total length (>= 5000 bp)"},{"values":[0,0,0,0,0,0,0,0,0],"quality":"More is better","isMain":true,"metricName":"Total length (>= 10000 bp)"},{"values":[0,0,0,0,0,0,0,0,0],"quality":"More is better","isMain":false,"metricName":"Total length (>= 25000 bp)"},{"values":[0,0,0,0,0,0,0,0,0],"quality":"More is better","isMain":true,"metricName":"Total length (>= 50000 bp)"},{"values":[1041,963,1053,987,888,1123,866,1271,1264],"quality":"More is better","isMain":false,"metricName":"N50"},{"values":[809,651,728,810,630,866,716,1001,945],"quality":"More is better","isMain":false,"metricName":"N75"},{"values":[12,9,11,2,5,11,12,10,10],"quality":"Less is better","isMain":false,"metricName":"L50"},{"values":[20,15,19,3,8,19,19,17,17],"quality":"Less is better","isMain":false,"metricName":"L75"},{"values":["40.03","40.42","40.15","42.16","39.87","40.18","39.75","40.73","40.69"],"quality":"Equal","isMain":false,"metricName":"GC (%)"}]],["Predicted genes",[]],["Similarity statistics",[{"values":[0,0,0,0,0,0,0,0,0],"quality":"Equal","isMain":false,"metricName":"# similar correct contigs"},{"values":[0,0,0,0,0,0,0,0,0],"quality":"Equal","isMain":false,"metricName":"# similar misassembled blocks"}]],["Reference statistics",[{"values":[134417,134417,134417,134417,134417,134417,134417,134417,134417],"quality":"Equal","isMain":false,"metricName":"Reference length"},{"values":[1,1,1,1,1,1,1,1,1],"quality":"Equal","isMain":false,"metricName":"Reference fragments"},{"values":["40.69","40.69","40.69","40.69","40.69","40.69","40.69","40.69","40.69"],"quality":"Equal","isMain":false,"metricName":"Reference GC (%)"}]]],"referenceName":"gi\_114680053\_ref\_NC","date":"18 July 2018, Wednesday, 16:08:38","order":[0,1,2,3,4,5,6,7,8],"assembliesNames":["CLC","IDBA\_UD","MEGAHIT","MIRA","SOAPdenovo2","SPAdes","SPAdes\_meta","SPAdes\_sc","SPAdes\_sc\_careful"]},{"assembliesWithNs":null,"minContig":500,"report":[["Genome statistics",[{"values":["9.538","3.947","11.464","6.660","12.166","12.933","12.401","12.401"],"quality":"More is better","isMain":true,"metricName":"Genome fraction (%)"},{"values":["1.000","1.048","1.002","1.000","1.000","1.004","1.009","1.009"],"quality":"Less is better","isMain":true,"metricName":"Duplication ratio"},{"values":[1291,1147,1084,1291,1291,1897,1291,1291],"quality":"More is better","isMain":true,"metricName":"Largest alignment"},{"values":[14967,6202,18014,10451,19091,20345,19482,19482],"quality":"More is better","isMain":true,"metricName":"Total aligned length"},{"values":[751,536,694,682,751,751,751,751],"quality":"More is better","isMain":false,"metricName":"NA50"},{"values":[548,null,611,560,634,667,582,582],"quality":"More is better","isMain":false,"metricName":"NA75"},{"values":[null,null,null,null,null,null,null,null],"quality":"More is better","isMain":true,"metricName":"NGA50"},{"values":[9,7,12,7,12,11,12,12],"quality":"Less is better","isMain":false,"metricName":"LA50"},{"values":[15,null,19,11,19,18,20,20],"quality":"Less is better","isMain":false,"metricName":"LA75"}]],["Misassemblies",[{"values":[1,0,0,0,0,0,0,0],"quality":"Less is better","isMain":true,"metricName":"# misassemblies"},{"values":[1,0,0,0,0,0,0,0],"quality":"Less is better","isMain":false,"metricName":" # relocations"},{"values":[0,0,0,0,0,0,0,0],"quality":"Less is better","isMain":false,"metricName":" # translocations"},{"values":[0,0,0,0,0,0,0,0],"quality":"Less is better","isMain":false,"metricName":" # inversions"},{"values":[1,0,0,0,0,0,0,0],"quality":"Less is better","isMain":false,"metricName":"# misassembled contigs"},{"values":[504,0,0,0,0,0,0,0],"quality":"Less is better","isMain":true,"metricName":"Misassembled contigs length"},{"values":[0,0,1,0,0,1,0,0],"quality":"Less is better","isMain":false,"metricName":"# local misassemblies"},{"values":[0,0,0,0,0,0,0,0],"quality":"Less is better","isMain":false,"metricName":"# unaligned mis. contigs"}]],["Unaligned",[{"values":[0,0,0,0,0,0,0,0],"quality":"Less is better","isMain":false,"metricName":"# fully unaligned contigs"},{"values":[0,0,0,0,0,0,0,0],"quality":"Less is better","isMain":false,"metricName":"Fully unaligned length"},{"values":[1,2,1,0,2,0,2,2],"quality":"Less is better","isMain":false,"metricName":"# partially unaligned contigs"},{"values":[900,2092,747,0,1757,0,1757,1757],"quality":"Less is better","isMain":false,"metricName":"Partially unaligned length"}]],["Mismatches",[{"values":[51,12,40,19,53,47,66,61],"quality":"Less is better","isMain":false,"metricName":"# mismatches"},{"values":[5,1,1,0,2,5,1,1],"quality":"Less is better","isMain":false,"metricName":"# indels"},{"values":[6,8,2,0,2,29,22,22],"quality":"Less is better","isMain":false,"metricName":"Indels length"},{"values":["340.75","193.74","222.35","181.80","277.62","231.58","339.16","313.46"],"quality":"Less is better","isMain":true,"metricName":"# mismatches per 100 kbp"},{"values":["33.41","16.14","5.56","0.00","10.48","24.64","5.14","5.14"],"quality":"Less is better","isMain":true,"metricName":"# indels per 100 kbp"},{"values":[5,0,1,0,2,4,0,0],"quality":"Less is better","isMain":false,"metricName":" # indels (<= 5 bp)"},{"values":[0,1,0,0,0,1,1,1],"quality":"Less is better","isMain":false,"metricName":" # indels (> 5 bp)"},{"values":[0,0,0,0,0,0,0,0],"quality":"Less is better","isMain":false,"metricName":"# N's"},{"values":["0.00","0.00","0.00","0.00","0.00","0.00","0.00","0.00"],"quality":"Less is better","isMain":true,"metricName":"# N's per 100 kbp"}]],["Statistics without reference",[{"values":[20,10,26,15,26,26,27,27],"quality":"Equal","isMain":true,"metricName":"# contigs"},{"values":[3,3,3,1,5,3,4,4],"quality":"Equal","isMain":false,"metricName":"# contigs (>= 1000 bp)"},{"values":[0,0,0,0,0,0,0,0],"quality":"Equal","isMain":false,"metricName":"# contigs (>= 5000 bp)"},{"values":[0,0,0,0,0,0,0,0],"quality":"Equal","isMain":false,"metricName":"# contigs (>= 10000 bp)"},{"values":[0,0,0,0,0,0,0,0],"quality":"Equal","isMain":false,"metricName":"# contigs (>= 25000 bp)"},{"values":[0,0,0,0,0,0,0,0],"quality":"Equal","isMain":false,"metricName":"# contigs (>= 50000 bp)"},{"values":[1391,1716,1168,1291,1391,1897,1391,1391],"quality":"More is better","isMain":true,"metricName":"Largest contig"},{"values":[15871,8585,18764,10451,20848,20386,21399,21399],"quality":"More is better","isMain":true,"metricName":"Total length"},{"values":[3949,4254,3254,1291,5844,4190,4808,4808],"quality":"More is better","isMain":true,"metricName":"Total length (>= 1000 bp)"},{"values":[0,0,0,0,0,0,0,0],"quality":"More is better","isMain":false,"metricName":"Total length (>= 5000 bp)"},{"values":[0,0,0,0,0,0,0,0],"quality":"More is better","isMain":true,"metricName":"Total length (>= 10000 bp)"},{"values":[0,0,0,0,0,0,0,0],"quality":"More is better","isMain":false,"metricName":"Total length (>= 25000 bp)"},{"values":[0,0,0,0,0,0,0,0],"quality":"More is better","isMain":true,"metricName":"Total length (>= 50000 bp)"},{"values":[803,852,743,682,805,751,803,803],"quality":"More is better","isMain":false,"metricName":"N50"},{"values":[682,575,634,560,694,667,682,682],"quality":"More is better","isMain":false,"metricName":"N75"},{"values":[8,4,11,7,11,11,11,11],"quality":"Less is better","isMain":false,"metricName":"L50"},{"values":[13,7,18,11,18,18,18,18],"quality":"Less is better","isMain":false,"metricName":"L75"},{"values":["48.14","48.39","48.29","49.32","48.47","48.36","48.42","48.43"],"quality":"Equal","isMain":false,"metricName":"GC (%)"}]],["Predicted genes",[]],["Similarity statistics",[{"values":[0,0,0,0,0,0,0,0],"quality":"Equal","isMain":false,"metricName":"# similar correct contigs"},{"values":[0,0,0,0,0,0,0,0],"quality":"Equal","isMain":false,"metricName":"# similar misassembled blocks"}]],["Reference statistics",[{"values":[156922,156922,156922,156922,156922,156922,156922,156922],"quality":"Equal","isMain":false,"metricName":"Reference length"},{"values":[1,1,1,1,1,1,1,1],"quality":"Equal","isMain":false,"metricName":"Reference fragments"},{"values":["49.26","49.26","49.26","49.26","49.26","49.26","49.26","49.26"],"quality":"Equal","isMain":false,"metricName":"Reference GC (%)"}]]],"referenceName":"gi\_115298502\_ref\_NC","date":"18 July 2018, Wednesday, 16:08:47","order":[0,1,2,3,4,5,6,7],"assembliesNames":["CLC","IDBA\_UD","MEGAHIT","SOAPdenovo2","SPAdes","SPAdes\_meta","SPAdes\_sc","SPAdes\_sc\_careful"]},{"assembliesWithNs":null,"minContig":500,"report":[["Genome statistics",[{"values":["86.229","32.283","96.184","64.489","82.063","99.622","74.364","93.679","61.490","63.351","58.189","1.966","99.296"],"quality":"More is better","isMain":true,"metricName":"Genome fraction (%)"},{"values":["1.123","1.011","1.015","1.011","1.064","2.046","1.118","1.041","1.000","1.013","1.013","1.000","1.792"],"quality":"Less is better","isMain":true,"metricName":"Duplication ratio"},{"values":[6646,2810,12708,3885,4835,12301,5442,8814,11188,3705,5132,561,15257],"quality":"More is better","isMain":true,"metricName":"Largest alignment"},{"values":[27654,9314,27854,18603,24905,57991,23373,27750,17549,18299,16826,561,50779],"quality":"More is better","isMain":true,"metricName":"Total aligned length"},{"values":[8910,null,8334,955,1315,88168,2594,5656,3324,879,847,null,106226],"quality":"More is better","isMain":false,"metricName":"NG50"},{"values":[6646,null,2755,null,597,88168,2093,2966,null,null,null,null,106226],"quality":"More is better","isMain":false,"metricName":"NG75"},{"values":[997,2192,8334,1206,1436,902,2679,5656,11188,1454,3705,561,null],"quality":"More is better","isMain":false,"metricName":"NA50"},{"values":[null,780,8334,965,805,null,2412,2966,3324,879,893,561,null],"quality":"More is better","isMain":false,"metricName":"NA75"},{"values":[2862,null,8334,955,1315,10024,2594,5656,3324,879,847,null,15257],"quality":"More is better","isMain":true,"metricName":"NGA50"},{"values":[877,null,2755,null,597,10024,2093,2966,null,null,null,null,12301],"quality":"More is better","isMain":false,"metricName":"NGA75"},{"values":[2,null,2,10,7,1,4,2,2,8,7,null,1],"quality":"Less is better","isMain":false,"metricName":"LG50"},{"values":[3,null,3,null,16,1,7,4,null,null,null,null,1],"quality":"Less is better","isMain":false,"metricName":"LG75"},{"values":[6,2,2,5,6,11,3,2,1,4,2,1,null],"quality":"Less is better","isMain":false,"metricName":"LA50"},{"values":[null,5,2,9,12,null,5,4,2,8,5,1,null],"quality":"Less is better","isMain":false,"metricName":"LA75"},{"values":[4,null,2,10,7,2,4,2,2,8,7,null,1],"quality":"Less is better","isMain":true,"metricName":"LGA50"},{"values":[8,null,3,null,16,2,7,4,null,null,null,null,2],"quality":"Less is better","isMain":false,"metricName":"LGA75"}]],["Misassemblies",[{"values":[0,0,0,0,0,0,0,0,0,0,0,0,0],"quality":"Less is better","isMain":true,"metricName":"# misassemblies"},{"values":[0,0,0,0,0,0,0,0,0,0,0,0,0],"quality":"Less is better","isMain":false,"metricName":" # relocations"},{"values":[0,0,0,0,0,0,0,0,0,0,0,0,0],"quality":"Less is better","isMain":false,"metricName":" # translocations"},{"values":[0,0,0,0,0,0,0,0,0,0,0,0,0],"quality":"Less is better","isMain":false,"metricName":" # inversions"},{"values":[0,0,0,0,0,0,0,0,0,0,0,0,0],"quality":"Less is better","isMain":false,"metricName":"# misassembled contigs"},{"values":[0,0,0,0,0,0,0,0,0,0,0,0,0],"quality":"Less is better","isMain":true,"metricName":"Misassembled contigs length"},{"values":[0,0,0,0,0,1,0,0,0,0,0,0,0],"quality":"Less is better","isMain":false,"metricName":"# local misassemblies"},{"values":[2,0,0,0,0,1,0,0,0,0,0,0,1],"quality":"Less is better","isMain":false,"metricName":"# unaligned mis. contigs"}]],["Unaligned",[{"values":[0,0,0,0,0,0,0,0,0,0,0,0,0],"quality":"Less is better","isMain":false,"metricName":"# fully unaligned contigs"},{"values":[0,0,0,0,0,0,0,0,0,0,0,0,0],"quality":"Less is better","isMain":false,"metricName":"Fully unaligned length"},{"values":[2,0,0,0,0,1,0,0,0,0,0,0,1],"quality":"Less is better","isMain":false,"metricName":"# partially unaligned contigs"},{"values":[11477,0,0,0,0,46964,0,0,0,0,0,0,55454],"quality":"Less is better","isMain":false,"metricName":"Partially unaligned length"}]],["Mismatches",[{"values":[15,0,21,0,22,309,30,8,196,124,6,0,133],"quality":"Less is better","isMain":false,"metricName":"# mismatches"},{"values":[0,0,0,0,0,9,0,0,3,2,0,0,7],"quality":"Less is better","isMain":false,"metricName":"# indels"},{"values":[0,0,0,0,0,15,0,0,3,2,0,0,13],"quality":"Less is better","isMain":false,"metricName":"Indels length"},{"values":["60.96","0.00","76.51","0.00","93.94","1086.88","141.36","29.92","1116.94","685.88","36.13","0.00","469.35"],"quality":"Less is better","isMain":true,"metricName":"# mismatches per 100 kbp"},{"values":["0.00","0.00","0.00","0.00","0.00","31.66","0.00","0.00","17.10","11.06","0.00","0.00","24.70"],"quality":"Less is better","isMain":true,"metricName":"# indels per 100 kbp"},{"values":[0,0,0,0,0,9,0,0,3,2,0,0,7],"quality":"Less is better","isMain":false,"metricName":" # indels (<= 5 bp)"},{"values":[0,0,0,0,0,0,0,0,0,0,0,0,0],"quality":"Less is better","isMain":false,"metricName":" # indels (> 5 bp)"},{"values":[111,0,0,0,0,1,0,0,0,0,0,0,50],"quality":"Less is better","isMain":false,"metricName":"# N's"},{"values":["283.71","0.00","0.00","0.00","0.00","0.95","0.00","0.00","0.00","0.00","0.00","0.00","47.07"],"quality":"Less is better","isMain":true,"metricName":"# N's per 100 kbp"}]],["Statistics without reference",[{"values":[14,8,6,16,22,9,8,10,4,13,11,1,1],"quality":"Equal","isMain":true,"metricName":"# contigs"},{"values":[5,2,4,8,9,4,8,6,4,7,4,0,1],"quality":"Equal","isMain":false,"metricName":"# contigs (>= 1000 bp)"},{"values":[3,0,2,0,0,2,1,2,1,0,1,0,1],"quality":"Equal","isMain":false,"metricName":"# contigs (>= 5000 bp)"},{"values":[1,0,1,0,0,1,0,0,1,0,0,0,1],"quality":"Equal","isMain":false,"metricName":"# contigs (>= 10000 bp)"},{"values":[0,0,0,0,0,1,0,0,0,0,0,0,1],"quality":"Equal","isMain":false,"metricName":"# contigs (>= 25000 bp)"},{"values":[0,0,0,0,0,1,0,0,0,0,0,0,1],"quality":"Equal","isMain":false,"metricName":"# contigs (>= 50000 bp)"},{"values":[10316,2810,12708,3885,4835,88168,5442,8814,11188,3705,5132,561,106226],"quality":"More is better","isMain":true,"metricName":"Largest contig"},{"values":[39124,9314,27854,18603,24911,105131,23724,27830,17549,18307,16826,561,106226],"quality":"More is better","isMain":true,"metricName":"Total length"},{"values":[32703,5002,26541,13082,16585,101012,23724,25457,17549,13490,11837,0,106226],"quality":"More is better","isMain":true,"metricName":"Total length (>= 1000 bp)"},{"values":[25872,0,21042,0,0,97965,5442,14470,11188,0,5132,0,106226],"quality":"More is better","isMain":false,"metricName":"Total length (>= 5000 bp)"},{"values":[10316,0,12708,0,0,88168,0,0,11188,0,0,0,106226],"quality":"More is better","isMain":true,"metricName":"Total length (>= 10000 bp)"},{"values":[0,0,0,0,0,88168,0,0,0,0,0,0,106226],"quality":"More is better","isMain":false,"metricName":"Total length (>= 25000 bp)"},{"values":[0,0,0,0,0,88168,0,0,0,0,0,0,106226],"quality":"More is better","isMain":true,"metricName":"Total length (>= 50000 bp)"},{"values":[6646,2192,8334,1206,1436,88168,3030,5656,11188,1454,3705,561,106226],"quality":"More is better","isMain":false,"metricName":"N50"},{"values":[3969,780,8334,965,805,88168,2412,2966,3324,879,893,561,106226],"quality":"More is better","isMain":false,"metricName":"N75"},{"values":[3,2,2,5,6,1,3,2,1,4,2,1,1],"quality":"Less is better","isMain":false,"metricName":"L50"},{"values":[4,5,2,9,12,1,5,4,2,8,5,1,1],"quality":"Less is better","isMain":false,"metricName":"L75"},{"values":["34.64","33.55","34.70","35.03","34.56","34.80","34.41","34.56","34.30","34.31","34.43","27.99","34.35"],"quality":"Equal","isMain":false,"metricName":"GC (%)"}]],["Predicted genes",[]],["Similarity statistics",[{"values":[0,0,0,0,0,0,0,0,0,0,0,0,0],"quality":"Equal","isMain":false,"metricName":"# similar correct contigs"},{"values":[0,0,0,0,0,0,0,0,0,0,0,0,0],"quality":"Equal","isMain":false,"metricName":"# similar misassembled blocks"}]],["Reference statistics",[{"values":[28538,28538,28538,28538,28538,28538,28538,28538,28538,28538,28538,28538,28538],"quality":"Equal","isMain":false,"metricName":"Reference length"},{"values":[1,1,1,1,1,1,1,1,1,1,1,1,1],"quality":"Equal","isMain":false,"metricName":"Reference fragments"},{"values":["34.68","34.68","34.68","34.68","34.68","34.68","34.68","34.68","34.68","34.68","34.68","34.68","34.68"],"quality":"Equal","isMain":false,"metricName":"Reference GC (%)"}]]],"referenceName":"gi\_115304210\_ref\_NC","date":"18 July 2018, Wednesday, 16:08:57","order":[0,1,2,3,4,5,6,7,8,9,10,11,12],"assembliesNames":["ABySS\_127","ABySS\_63","CLC","IDBA\_UD","MEGAHIT","MIRA","Ray\_Meta","SPAdes","SPAdes\_meta","SPAdes\_sc","SPAdes\_sc\_careful","Velvet","Geneious"]},{"assembliesWithNs":null,"minContig":500,"report":[["Genome statistics",[{"values":["99.634","100.000","100.000","100.000","12.865","92.358","100.000","100.000","100.000","100.000","100.000"],"quality":"More is better","isMain":true,"metricName":"Genome fraction (%)"},{"values":["1.004","1.004","1.005","1.007","1.001","1.014","1.005","1.002","1.002","1.002","1.001"],"quality":"Less is better","isMain":true,"metricName":"Duplication ratio"},{"values":[26440,26636,26678,26736,862,24861,26664,26592,26592,26592,26567],"quality":"More is better","isMain":true,"metricName":"Largest alignment"},{"values":[26440,26636,26678,26736,3418,24861,26664,26592,26592,26592,26567],"quality":"More is better","isMain":true,"metricName":"Total aligned length"},{"values":[26537,26636,26678,26736,null,27370,26664,26592,26592,26592,26567],"quality":"More is better","isMain":false,"metricName":"NG50"},{"values":[26537,26636,26678,26736,null,27370,26664,26592,26592,26592,26567],"quality":"More is better","isMain":false,"metricName":"NG75"},{"values":[26440,26636,26678,26736,614,24861,26664,26592,26592,26592,26567],"quality":"More is better","isMain":false,"metricName":"NA50"},{"values":[26440,26636,26678,26736,575,24861,26664,26592,26592,26592,26567],"quality":"More is better","isMain":false,"metricName":"NA75"},{"values":[26440,26636,26678,26736,null,24861,26664,26592,26592,26592,26567],"quality":"More is better","isMain":true,"metricName":"NGA50"},{"values":[26440,26636,26678,26736,null,24861,26664,26592,26592,26592,26567],"quality":"More is better","isMain":false,"metricName":"NGA75"},{"values":[1,1,1,1,null,1,1,1,1,1,1],"quality":"Less is better","isMain":false,"metricName":"LG50"},{"values":[1,1,1,1,null,1,1,1,1,1,1],"quality":"Less is better","isMain":false,"metricName":"LG75"},{"values":[1,1,1,1,3,1,1,1,1,1,1],"quality":"Less is better","isMain":false,"metricName":"LA50"},{"values":[1,1,1,1,4,1,1,1,1,1,1],"quality":"Less is better","isMain":false,"metricName":"LA75"},{"values":[1,1,1,1,null,1,1,1,1,1,1],"quality":"Less is better","isMain":true,"metricName":"LGA50"},{"values":[1,1,1,1,null,1,1,1,1,1,1],"quality":"Less is better","isMain":false,"metricName":"LGA75"}]],["Misassemblies",[{"values":[0,0,0,0,0,0,0,0,0,0,0],"quality":"Less is better","isMain":true,"metricName":"# misassemblies"},{"values":[0,0,0,0,0,0,0,0,0,0,0],"quality":"Less is better","isMain":false,"metricName":" # relocations"},{"values":[0,0,0,0,0,0,0,0,0,0,0],"quality":"Less is better","isMain":false,"metricName":" # translocations"},{"values":[0,0,0,0,0,0,0,0,0,0,0],"quality":"Less is better","isMain":false,"metricName":" # inversions"},{"values":[0,0,0,0,0,0,0,0,0,0,0],"quality":"Less is better","isMain":false,"metricName":"# misassembled contigs"},{"values":[0,0,0,0,0,0,0,0,0,0,0],"quality":"Less is better","isMain":true,"metricName":"Misassembled contigs length"},{"values":[0,1,1,1,0,1,1,0,0,0,0],"quality":"Less is better","isMain":false,"metricName":"# local misassemblies"},{"values":[0,0,0,0,0,0,0,0,0,0,0],"quality":"Less is better","isMain":false,"metricName":"# unaligned mis. contigs"}]],["Unaligned",[{"values":[0,0,0,0,0,0,0,0,0,0,0],"quality":"Less is better","isMain":false,"metricName":"# fully unaligned contigs"},{"values":[0,0,0,0,0,0,0,0,0,0,0],"quality":"Less is better","isMain":false,"metricName":"Fully unaligned length"},{"values":[0,0,0,0,0,1,0,0,0,0,0],"quality":"Less is better","isMain":false,"metricName":"# partially unaligned contigs"},{"values":[0,0,0,0,0,2509,0,0,0,0,0],"quality":"Less is better","isMain":false,"metricName":"Partially unaligned length"}]],["Mismatches",[{"values":[0,0,0,0,0,0,0,0,0,0,0],"quality":"Less is better","isMain":false,"metricName":"# mismatches"},{"values":[0,0,0,0,0,13,0,1,1,1,1],"quality":"Less is better","isMain":false,"metricName":"# indels"},{"values":[0,0,0,0,0,655,0,55,55,55,30],"quality":"Less is better","isMain":false,"metricName":"Indels length"},{"values":["0.00","0.00","0.00","0.00","0.00","0.00","0.00","0.00","0.00","0.00","0.00"],"quality":"Less is better","isMain":true,"metricName":"# mismatches per 100 kbp"},{"values":["0.00","0.00","0.00","0.00","0.00","53.04","0.00","3.77","3.77","3.77","3.77"],"quality":"Less is better","isMain":true,"metricName":"# indels per 100 kbp"},{"values":[0,0,0,0,0,0,0,0,0,0,0],"quality":"Less is better","isMain":false,"metricName":" # indels (<= 5 bp)"},{"values":[0,0,0,0,0,13,0,1,1,1,1],"quality":"Less is better","isMain":false,"metricName":" # indels (> 5 bp)"},{"values":[0,0,0,0,0,1437,0,0,0,0,0],"quality":"Less is better","isMain":false,"metricName":"# N's"},{"values":["0.00","0.00","0.00","0.00","0.00","5250.27","0.00","0.00","0.00","0.00","0.00"],"quality":"Less is better","isMain":true,"metricName":"# N's per 100 kbp"}]],["Statistics without reference",[{"values":[1,1,1,1,5,1,1,1,1,1,1],"quality":"Equal","isMain":true,"metricName":"# contigs"},{"values":[1,1,1,1,0,1,1,1,1,1,1],"quality":"Equal","isMain":false,"metricName":"# contigs (>= 1000 bp)"},{"values":[1,1,1,1,0,1,1,1,1,1,1],"quality":"Equal","isMain":false,"metricName":"# contigs (>= 5000 bp)"},{"values":[1,1,1,1,0,1,1,1,1,1,1],"quality":"Equal","isMain":false,"metricName":"# contigs (>= 10000 bp)"},{"values":[1,1,1,1,0,1,1,1,1,1,1],"quality":"Equal","isMain":false,"metricName":"# contigs (>= 25000 bp)"},{"values":[0,0,0,0,0,0,0,0,0,0,0],"quality":"Equal","isMain":false,"metricName":"# contigs (>= 50000 bp)"},{"values":[26537,26636,26678,26736,862,27370,26664,26592,26592,26592,26567],"quality":"More is better","isMain":true,"metricName":"Largest contig"},{"values":[26537,26636,26678,26736,3418,27370,26664,26592,26592,26592,26567],"quality":"More is better","isMain":true,"metricName":"Total length"},{"values":[26537,26636,26678,26736,0,27370,26664,26592,26592,26592,26567],"quality":"More is better","isMain":true,"metricName":"Total length (>= 1000 bp)"},{"values":[26537,26636,26678,26736,0,27370,26664,26592,26592,26592,26567],"quality":"More is better","isMain":false,"metricName":"Total length (>= 5000 bp)"},{"values":[26537,26636,26678,26736,0,27370,26664,26592,26592,26592,26567],"quality":"More is better","isMain":true,"metricName":"Total length (>= 10000 bp)"},{"values":[26537,26636,26678,26736,0,27370,26664,26592,26592,26592,26567],"quality":"More is better","isMain":false,"metricName":"Total length (>= 25000 bp)"},{"values":[0,0,0,0,0,0,0,0,0,0,0],"quality":"More is better","isMain":true,"metricName":"Total length (>= 50000 bp)"},{"values":[26537,26636,26678,26736,614,27370,26664,26592,26592,26592,26567],"quality":"More is better","isMain":false,"metricName":"N50"},{"values":[26537,26636,26678,26736,575,27370,26664,26592,26592,26592,26567],"quality":"More is better","isMain":false,"metricName":"N75"},{"values":[1,1,1,1,3,1,1,1,1,1,1],"quality":"Less is better","isMain":false,"metricName":"L50"},{"values":[1,1,1,1,4,1,1,1,1,1,1],"quality":"Less is better","isMain":false,"metricName":"L75"},{"values":["37.10","37.13","37.11","37.10","41.37","37.10","37.14","37.12","37.12","37.12","37.10"],"quality":"Equal","isMain":false,"metricName":"GC (%)"}]],["Predicted genes",[]],["Similarity statistics",[{"values":[0,0,0,0,0,0,0,0,0,0,0],"quality":"Equal","isMain":false,"metricName":"# similar correct contigs"},{"values":[0,0,0,0,0,0,0,0,0,0,0],"quality":"Equal","isMain":false,"metricName":"# similar misassembled blocks"}]],["Reference statistics",[{"values":[26537,26537,26537,26537,26537,26537,26537,26537,26537,26537,26537],"quality":"Equal","isMain":false,"metricName":"Reference length"},{"values":[1,1,1,1,1,1,1,1,1,1,1],"quality":"Equal","isMain":false,"metricName":"Reference fragments"},{"values":["37.10","37.10","37.10","37.10","37.10","37.10","37.10","37.10","37.10","37.10","37.10"],"quality":"Equal","isMain":false,"metricName":"Reference GC (%)"}]]],"referenceName":"gi\_115304270\_ref\_NC","date":"18 July 2018, Wednesday, 16:09:07","order":[0,1,2,3,4,5,6,7,8,9,10],"assembliesNames":["CLC","IDBA\_UD","MEGAHIT","MIRA","Ray\_Meta","SOAPdenovo2","SPAdes","SPAdes\_meta","SPAdes\_sc","SPAdes\_sc\_careful","Velvet"]},{"assembliesWithNs":null,"minContig":500,"report":[["Genome statistics",[{"values":["94.182","69.862","83.183","81.704","85.529","79.695","79.797","92.199","47.584","71.872","78.614","19.613","100.000"],"quality":"More is better","isMain":true,"metricName":"Genome fraction (%)"},{"values":["1.095","1.000","1.005","1.017","1.016","1.248","1.043","1.018","0.999","1.006","1.000","1.000","1.494"],"quality":"Less is better","isMain":true,"metricName":"Duplication ratio"},{"values":[6178,4853,4680,4565,4264,8004,4605,7066,5499,4511,5573,1048,8352],"quality":"More is better","isMain":true,"metricName":"Largest alignment"},{"values":[31456,21315,25496,25344,26513,29783,25396,28638,14500,22039,23986,5984,45591],"quality":"More is better","isMain":true,"metricName":"Total aligned length"},{"values":[5634,2113,3943,1915,3032,8576,4039,4642,5054,2860,3236,null,106226],"quality":"More is better","isMain":false,"metricName":"NG50"},{"values":[4117,null,2794,953,1379,4739,2584,3828,null,null,1169,null,106226],"quality":"More is better","isMain":false,"metricName":"NG75"},{"values":[5634,3442,4064,2210,3109,4739,4429,4642,5054,3358,3358,661,null],"quality":"More is better","isMain":false,"metricName":"NA50"},{"values":[4117,2113,3368,1702,2532,981,3532,4274,3947,2860,2860,622,null],"quality":"More is better","isMain":false,"metricName":"NA75"},{"values":[5634,2113,3943,1915,3032,7526,4039,4642,null,2860,3236,null,8004],"quality":"More is better","isMain":true,"metricName":"NGA50"},{"values":[4117,null,2794,953,1379,2862,2584,3828,null,null,1169,null,7340],"quality":"More is better","isMain":false,"metricName":"NGA75"},{"values":[3,5,4,5,5,2,4,3,3,5,4,null,1],"quality":"Less is better","isMain":false,"metricName":"LG50"},{"values":[5,null,6,10,8,3,6,5,null,null,8,null,1],"quality":"Less is better","isMain":false,"metricName":"LG75"},{"values":[3,3,3,4,4,3,3,3,2,3,3,4,null],"quality":"Less is better","isMain":false,"metricName":"LA50"},{"values":[5,5,5,7,6,8,5,4,3,5,5,6,null],"quality":"Less is better","isMain":false,"metricName":"LA75"},{"values":[3,5,4,5,5,2,4,3,null,5,4,null,2],"quality":"Less is better","isMain":true,"metricName":"LGA50"},{"values":[5,null,6,10,8,4,6,5,null,null,8,null,3],"quality":"Less is better","isMain":false,"metricName":"LGA75"}]],["Misassemblies",[{"values":[0,0,0,0,0,0,0,0,0,0,0,0,0],"quality":"Less is better","isMain":true,"metricName":"# misassemblies"},{"values":[0,0,0,0,0,0,0,0,0,0,0,0,0],"quality":"Less is better","isMain":false,"metricName":" # relocations"},{"values":[0,0,0,0,0,0,0,0,0,0,0,0,0],"quality":"Less is better","isMain":false,"metricName":" # translocations"},{"values":[0,0,0,0,0,0,0,0,0,0,0,0,0],"quality":"Less is better","isMain":false,"metricName":" # inversions"},{"values":[0,0,0,0,0,0,0,0,0,0,0,0,0],"quality":"Less is better","isMain":false,"metricName":"# misassembled contigs"},{"values":[0,0,0,0,0,0,0,0,0,0,0,0,0],"quality":"Less is better","isMain":true,"metricName":"Misassembled contigs length"},{"values":[0,0,0,0,0,1,0,0,1,0,0,0,0],"quality":"Less is better","isMain":false,"metricName":"# local misassemblies"},{"values":[0,0,0,0,0,0,0,0,0,0,0,0,2],"quality":"Less is better","isMain":false,"metricName":"# unaligned mis. contigs"}]],["Unaligned",[{"values":[0,0,0,0,0,0,0,0,0,0,0,0,0],"quality":"Less is better","isMain":false,"metricName":"# fully unaligned contigs"},{"values":[0,0,0,0,0,0,0,0,0,0,0,0,0],"quality":"Less is better","isMain":false,"metricName":"Fully unaligned length"},{"values":[0,0,0,0,0,2,0,0,2,0,0,0,3],"quality":"Less is better","isMain":false,"metricName":"# partially unaligned contigs"},{"values":[0,0,0,0,0,6686,0,0,3923,0,0,0,114320],"quality":"Less is better","isMain":false,"metricName":"Partially unaligned length"}]],["Mismatches",[{"values":[22,21,38,0,7,211,0,29,204,52,126,0,202],"quality":"Less is better","isMain":false,"metricName":"# mismatches"},{"values":[0,0,0,0,0,10,0,0,14,1,1,0,8],"quality":"Less is better","isMain":false,"metricName":"# indels"},{"values":[0,0,0,0,0,16,0,0,24,1,1,0,19],"quality":"Less is better","isMain":false,"metricName":"Indels length"},{"values":["76.56","98.52","149.73","0.00","26.83","867.78","0.00","103.09","1405.15","237.14","525.33","0.00","662.08"],"quality":"Less is better","isMain":true,"metricName":"# mismatches per 100 kbp"},{"values":["0.00","0.00","0.00","0.00","0.00","41.13","0.00","0.00","96.43","4.56","4.17","0.00","26.22"],"quality":"Less is better","isMain":true,"metricName":"# indels per 100 kbp"},{"values":[0,0,0,0,0,10,0,0,14,1,1,0,7],"quality":"Less is better","isMain":false,"metricName":" # indels (<= 5 bp)"},{"values":[0,0,0,0,0,0,0,0,0,0,0,0,1],"quality":"Less is better","isMain":false,"metricName":" # indels (> 5 bp)"},{"values":[0,0,0,0,0,9,0,0,0,0,0,0,286],"quality":"Less is better","isMain":false,"metricName":"# N's"},{"values":["0.00","0.00","0.00","0.00","0.00","24.30","0.00","0.00","0.00","0.00","0.00","0.00","178.85"],"quality":"Less is better","isMain":true,"metricName":"# N's per 100 kbp"}]],["Statistics without reference",[{"values":[7,11,8,13,11,10,7,8,3,8,8,8,4],"quality":"Equal","isMain":true,"metricName":"# contigs"},{"values":[6,5,8,9,8,7,7,6,3,7,8,2,4],"quality":"Equal","isMain":false,"metricName":"# contigs (>= 1000 bp)"},{"values":[3,0,0,0,0,2,0,2,3,0,1,0,3],"quality":"Equal","isMain":false,"metricName":"# contigs (>= 5000 bp)"},{"values":[0,0,0,0,0,1,0,0,0,0,0,0,2],"quality":"Equal","isMain":false,"metricName":"# contigs (>= 10000 bp)"},{"values":[0,0,0,0,0,0,0,0,0,0,0,0,2],"quality":"Equal","isMain":false,"metricName":"# contigs (>= 25000 bp)"},{"values":[0,0,0,0,0,0,0,0,0,0,0,0,1],"quality":"Equal","isMain":false,"metricName":"# contigs (>= 50000 bp)"},{"values":[6178,4853,4680,4565,4264,10863,4605,7066,7121,4513,5573,1048,106226],"quality":"More is better","isMain":true,"metricName":"Largest contig"},{"values":[31456,21315,25501,25344,26513,37041,25396,28638,18423,22051,23986,5984,159911],"quality":"More is better","isMain":true,"metricName":"Total length"},{"values":[30913,17106,25501,22174,23857,34701,25396,27514,18423,21455,23986,2074,159911],"quality":"More is better","isMain":true,"metricName":"Total length (>= 1000 bp)"},{"values":[17924,0,0,0,0,19439,0,13761,18423,0,5573,0,158562],"quality":"More is better","isMain":false,"metricName":"Total length (>= 5000 bp)"},{"values":[0,0,0,0,0,10863,0,0,0,0,0,0,153194],"quality":"More is better","isMain":true,"metricName":"Total length (>= 10000 bp)"},{"values":[0,0,0,0,0,0,0,0,0,0,0,0,153194],"quality":"More is better","isMain":false,"metricName":"Total length (>= 25000 bp)"},{"values":[0,0,0,0,0,0,0,0,0,0,0,0,106226],"quality":"More is better","isMain":true,"metricName":"Total length (>= 50000 bp)"},{"values":[5634,3442,4064,2210,3109,8576,4429,4642,6248,3358,3358,661,106226],"quality":"More is better","isMain":false,"metricName":"N50"},{"values":[4117,2113,3368,1702,2532,4523,3532,4274,5054,2860,2860,622,46968],"quality":"More is better","isMain":false,"metricName":"N75"},{"values":[3,3,3,4,4,2,3,3,2,3,3,4,1],"quality":"Less is better","isMain":false,"metricName":"L50"},{"values":[5,5,5,7,6,4,5,4,3,5,5,6,2],"quality":"Less is better","isMain":false,"metricName":"L75"},{"values":["33.70","34.06","33.98","33.91","33.96","33.72","33.38","33.93","33.82","33.94","34.16","33.77","34.45"],"quality":"Equal","isMain":false,"metricName":"GC (%)"}]],["Predicted genes",[]],["Similarity statistics",[{"values":[0,0,0,0,0,0,0,0,0,0,0,0,0],"quality":"Equal","isMain":false,"metricName":"# similar correct contigs"},{"values":[0,0,0,0,0,0,0,0,0,0,0,0,0],"quality":"Equal","isMain":false,"metricName":"# similar misassembled blocks"}]],["Reference statistics",[{"values":[30510,30510,30510,30510,30510,30510,30510,30510,30510,30510,30510,30510,30510],"quality":"Equal","isMain":false,"metricName":"Reference length"},{"values":[1,1,1,1,1,1,1,1,1,1,1,1,1],"quality":"Equal","isMain":false,"metricName":"Reference fragments"},{"values":["33.88","33.88","33.88","33.88","33.88","33.88","33.88","33.88","33.88","33.88","33.88","33.88","33.88"],"quality":"Equal","isMain":false,"metricName":"Reference GC (%)"}]]],"referenceName":"gi\_115315572\_ref\_NC","date":"18 July 2018, Wednesday, 16:09:18","order":[0,1,2,3,4,5,6,7,8,9,10,11,12],"assembliesNames":["ABySS\_127","ABySS\_63","CLC","IDBA\_UD","MEGAHIT","MIRA","Ray\_Meta","SPAdes","SPAdes\_meta","SPAdes\_sc","SPAdes\_sc\_careful","Velvet","Geneious"]},{"assembliesWithNs":null,"minContig":500,"report":[["Genome statistics",[{"values":["79.878","32.867","73.500","55.225","74.068","100.000","62.561","89.287","57.943","52.519","56.861","5.526","17.437"],"quality":"More is better","isMain":true,"metricName":"Genome fraction (%)"},{"values":["1.044","1.006","1.065","1.003","1.063","1.667","1.039","1.078","1.027","1.016","1.015","1.000","1.006"],"quality":"Less is better","isMain":true,"metricName":"Duplication ratio"},{"values":[4015,2848,3417,2533,4362,28432,7607,3249,4573,2873,2660,944,4815],"quality":"More is better","isMain":true,"metricName":"Largest alignment"},{"values":[22892,9077,21449,15213,21602,45635,17839,26417,16343,14648,15841,1517,4815],"quality":"More is better","isMain":true,"metricName":"Total aligned length"},{"values":[4015,null,1002,593,1017,88168,2686,1908,3665,519,595,null,null],"quality":"More is better","isMain":false,"metricName":"NG50"},{"values":[915,null,517,null,536,88168,null,890,1446,null,null,null,null],"quality":"More is better","isMain":false,"metricName":"NG75"},{"values":[889,928,1284,1184,1632,null,4793,1908,1817,1072,2200,944,4815],"quality":"More is better","isMain":false,"metricName":"NA50"},{"values":[500,632,957,834,749,null,2686,972,null,810,1072,573,4815],"quality":"More is better","isMain":false,"metricName":"NA75"},{"values":[915,null,964,593,1017,28432,2686,1908,1446,519,595,null,null],"quality":"More is better","isMain":true,"metricName":"NGA50"},{"values":[604,null,517,null,536,28432,null,890,null,null,null,null,null],"quality":"More is better","isMain":false,"metricName":"NGA75"},{"values":[2,null,8,12,7,1,3,6,3,12,9,null,null],"quality":"Less is better","isMain":false,"metricName":"LG50"},{"values":[6,null,17,null,18,1,null,12,6,null,null,null,null],"quality":"Less is better","isMain":false,"metricName":"LG75"},{"values":[8,3,5,5,5,null,2,6,5,4,4,1,1],"quality":"Less is better","isMain":false,"metricName":"LA50"},{"values":[19,6,10,8,10,null,3,11,null,8,6,2,1],"quality":"Less is better","isMain":false,"metricName":"LA75"},{"values":[6,null,8,12,7,1,3,6,6,12,9,null,null],"quality":"Less is better","isMain":true,"metricName":"LGA50"},{"values":[15,null,17,null,18,1,null,12,null,null,null,null,null],"quality":"Less is better","isMain":false,"metricName":"LGA75"}]],["Misassemblies",[{"values":[0,0,0,0,0,0,0,0,0,0,0,0,0],"quality":"Less is better","isMain":true,"metricName":"# misassemblies"},{"values":[0,0,0,0,0,0,0,0,0,0,0,0,0],"quality":"Less is better","isMain":false,"metricName":" # relocations"},{"values":[0,0,0,0,0,0,0,0,0,0,0,0,0],"quality":"Less is better","isMain":false,"metricName":" # translocations"},{"values":[0,0,0,0,0,0,0,0,0,0,0,0,0],"quality":"Less is better","isMain":false,"metricName":" # inversions"},{"values":[0,0,0,0,0,0,0,0,0,0,0,0,0],"quality":"Less is better","isMain":false,"metricName":"# misassembled contigs"},{"values":[0,0,0,0,0,0,0,0,0,0,0,0,0],"quality":"Less is better","isMain":true,"metricName":"Misassembled contigs length"},{"values":[0,0,0,0,0,1,0,0,0,0,0,0,0],"quality":"Less is better","isMain":false,"metricName":"# local misassemblies"},{"values":[0,0,0,0,0,0,0,0,1,0,0,0,0],"quality":"Less is better","isMain":false,"metricName":"# unaligned mis. contigs"}]],["Unaligned",[{"values":[0,0,0,0,0,0,0,0,0,0,0,0,0],"quality":"Less is better","isMain":false,"metricName":"# fully unaligned contigs"},{"values":[0,0,0,0,0,0,0,0,0,0,0,0,0],"quality":"Less is better","isMain":false,"metricName":"Fully unaligned length"},{"values":[1,0,0,0,0,2,0,0,2,0,0,0,1],"quality":"Less is better","isMain":false,"metricName":"# partially unaligned contigs"},{"values":[7406,0,0,0,0,81435,0,0,5776,0,0,0,553],"quality":"Less is better","isMain":false,"metricName":"Partially unaligned length"}]],["Mismatches",[{"values":[0,0,42,0,49,273,5,3,134,48,19,0,70],"quality":"Less is better","isMain":false,"metricName":"# mismatches"},{"values":[0,0,2,0,3,13,2,0,3,1,0,0,18],"quality":"Less is better","isMain":false,"metricName":"# indels"},{"values":[0,0,21,0,3,26,2,0,5,1,0,0,32],"quality":"Less is better","isMain":false,"metricName":"Indels length"},{"values":["0.00","0.00","208.15","0.00","240.98","994.43","29.11","12.24","842.40","332.92","121.72","0.00","1462.29"],"quality":"Less is better","isMain":true,"metricName":"# mismatches per 100 kbp"},{"values":["0.00","0.00","9.91","0.00","14.75","47.35","11.64","0.00","18.86","6.94","0.00","0.00","376.02"],"quality":"Less is better","isMain":true,"metricName":"# indels per 100 kbp"},{"values":[0,0,1,0,3,12,2,0,3,1,0,0,18],"quality":"Less is better","isMain":false,"metricName":" # indels (<= 5 bp)"},{"values":[0,0,1,0,0,1,0,0,0,0,0,0,0],"quality":"Less is better","isMain":false,"metricName":" # indels (> 5 bp)"},{"values":[109,0,0,0,0,3,0,0,0,0,0,0,19],"quality":"Less is better","isMain":false,"metricName":"# N's"},{"values":["359.76","0.00","0.00","0.00","0.00","2.36","0.00","0.00","0.00","0.00","0.00","0.00","353.95"],"quality":"Less is better","isMain":true,"metricName":"# N's per 100 kbp"}]],["Statistics without reference",[{"values":[19,10,18,14,19,8,5,20,7,13,12,2,1],"quality":"Equal","isMain":true,"metricName":"# contigs"},{"values":[4,1,8,6,7,6,4,10,7,7,7,0,1],"quality":"Equal","isMain":false,"metricName":"# contigs (>= 1000 bp)"},{"values":[1,0,0,0,0,2,1,0,1,0,0,0,1],"quality":"Equal","isMain":false,"metricName":"# contigs (>= 5000 bp)"},{"values":[1,0,0,0,0,2,0,0,0,0,0,0,0],"quality":"Equal","isMain":false,"metricName":"# contigs (>= 10000 bp)"},{"values":[0,0,0,0,0,2,0,0,0,0,0,0,0],"quality":"Equal","isMain":false,"metricName":"# contigs (>= 25000 bp)"},{"values":[0,0,0,0,0,1,0,0,0,0,0,0,0],"quality":"Equal","isMain":false,"metricName":"# contigs (>= 50000 bp)"},{"values":[10316,2848,3417,2533,4362,88168,7607,3249,7121,2873,2660,944,5368],"quality":"More is better","isMain":true,"metricName":"Largest contig"},{"values":[30298,9077,21489,15213,21606,127207,17839,26417,22119,14648,15841,1517,5368],"quality":"More is better","isMain":true,"metricName":"Total length"},{"values":[19520,2848,14501,9764,13908,125899,17146,19519,22119,10935,12945,0,5368],"quality":"More is better","isMain":true,"metricName":"Total length (>= 1000 bp)"},{"values":[10316,0,0,0,0,116737,7607,0,7121,0,0,0,5368],"quality":"More is better","isMain":false,"metricName":"Total length (>= 5000 bp)"},{"values":[10316,0,0,0,0,116737,0,0,0,0,0,0,0],"quality":"More is better","isMain":true,"metricName":"Total length (>= 10000 bp)"},{"values":[0,0,0,0,0,116737,0,0,0,0,0,0,0],"quality":"More is better","isMain":false,"metricName":"Total length (>= 25000 bp)"},{"values":[0,0,0,0,0,88168,0,0,0,0,0,0,0],"quality":"More is better","isMain":true,"metricName":"Total length (>= 50000 bp)"},{"values":[2923,928,1284,1184,1632,88168,4793,1908,4573,1072,2200,944,5368],"quality":"More is better","isMain":false,"metricName":"N50"},{"values":[889,632,957,834,749,28569,2686,972,2336,810,1072,573,5368],"quality":"More is better","isMain":false,"metricName":"N75"},{"values":[3,3,5,5,5,1,2,6,2,4,4,1,1],"quality":"Less is better","isMain":false,"metricName":"L50"},{"values":[8,6,10,8,10,2,3,11,4,8,6,2,1],"quality":"Less is better","isMain":false,"metricName":"L75"},{"values":["35.52","34.46","34.83","35.12","34.94","34.66","34.70","35.14","33.49","34.70","34.99","36.85","32.98"],"quality":"Equal","isMain":false,"metricName":"GC (%)"}]],["Predicted genes",[]],["Similarity statistics",[{"values":[0,0,0,0,0,0,0,0,0,0,0,0,0],"quality":"Equal","isMain":false,"metricName":"# similar correct contigs"},{"values":[0,0,0,0,0,0,0,0,0,0,0,0,0],"quality":"Equal","isMain":false,"metricName":"# similar misassembled blocks"}]],["Reference statistics",[{"values":[27453,27453,27453,27453,27453,27453,27453,27453,27453,27453,27453,27453,27453],"quality":"Equal","isMain":false,"metricName":"Reference length"},{"values":[1,1,1,1,1,1,1,1,1,1,1,1,1],"quality":"Equal","isMain":false,"metricName":"Reference fragments"},{"values":["34.94","34.94","34.94","34.94","34.94","34.94","34.94","34.94","34.94","34.94","34.94","34.94","34.94"],"quality":"Equal","isMain":false,"metricName":"Reference GC (%)"}]]],"referenceName":"gi\_115315628\_ref\_NC","date":"18 July 2018, Wednesday, 16:09:30","order":[0,1,2,3,4,5,6,7,8,9,10,11,12],"assembliesNames":["ABySS\_127","ABySS\_63","CLC","IDBA\_UD","MEGAHIT","MIRA","Ray\_Meta","SPAdes","SPAdes\_meta","SPAdes\_sc","SPAdes\_sc\_careful","Velvet","Geneious"]},{"assembliesWithNs":null,"minContig":500,"report":[["Genome statistics",[{"values":["38.331","18.173","32.343","37.270","43.205","67.637","2.178","15.965","40.069","20.793","30.650","30.650","20.467","16.259"],"quality":"More is better","isMain":true,"metricName":"Genome fraction (%)"},{"values":["1.002","1.004","1.008","1.000","1.007","1.169","1.000","1.066","1.000","1.000","1.003","1.003","1.004","1.000"],"quality":"Less is better","isMain":true,"metricName":"Duplication ratio"},{"values":[3355,1901,2400,4131,7534,5304,682,1700,8006,3930,3874,3874,3851,4817],"quality":"More is better","isMain":true,"metricName":"Largest alignment"},{"values":[23137,10989,19483,22451,26212,47628,1312,9666,24139,12526,18510,18510,12382,9793],"quality":"More is better","isMain":true,"metricName":"Total aligned length"},{"values":[null,null,null,null,null,153388,null,null,null,null,null,null,null,4817],"quality":"More is better","isMain":false,"metricName":"NG50"},{"values":[null,null,null,null,null,153388,null,null,null,null,null,null,null,null],"quality":"More is better","isMain":false,"metricName":"NG75"},{"values":[1263,930,1477,1204,1309,null,682,799,1994,3419,1387,1387,1367,null],"quality":"More is better","isMain":false,"metricName":"NA50"},{"values":[692,613,995,750,909,null,630,203,1387,1387,931,931,792,null],"quality":"More is better","isMain":false,"metricName":"NA75"},{"values":[null,null,null,null,null,1520,null,null,null,null,null,null,null,null],"quality":"More is better","isMain":true,"metricName":"NGA50"},{"values":[null,null,null,null,null,725,null,null,null,null,null,null,null,null],"quality":"More is better","isMain":false,"metricName":"NGA75"},{"values":[null,null,null,null,null,1,null,null,null,null,null,null,null,2],"quality":"Less is better","isMain":false,"metricName":"LG50"},{"values":[null,null,null,null,null,1,null,null,null,null,null,null,null,null],"quality":"Less is better","isMain":false,"metricName":"LG75"},{"values":[6,5,6,6,5,null,1,6,4,2,5,5,3,null],"quality":"Less is better","isMain":false,"metricName":"LA50"},{"values":[13,8,10,12,11,null,2,12,7,4,9,9,6,null],"quality":"Less is better","isMain":false,"metricName":"LA75"},{"values":[null,null,null,null,null,15,null,null,null,null,null,null,null,null],"quality":"Less is better","isMain":true,"metricName":"LGA50"},{"values":[null,null,null,null,null,28,null,null,null,null,null,null,null,null],"quality":"Less is better","isMain":false,"metricName":"LGA75"}]],["Misassemblies",[{"values":[0,0,0,0,0,0,0,0,0,0,0,0,0,0],"quality":"Less is better","isMain":true,"metricName":"# misassemblies"},{"values":[0,0,0,0,0,0,0,0,0,0,0,0,0,0],"quality":"Less is better","isMain":false,"metricName":" # relocations"},{"values":[0,0,0,0,0,0,0,0,0,0,0,0,0,0],"quality":"Less is better","isMain":false,"metricName":" # translocations"},{"values":[0,0,0,0,0,0,0,0,0,0,0,0,0,0],"quality":"Less is better","isMain":false,"metricName":" # inversions"},{"values":[0,0,0,0,0,0,0,0,0,0,0,0,0,0],"quality":"Less is better","isMain":false,"metricName":"# misassembled contigs"},{"values":[0,0,0,0,0,0,0,0,0,0,0,0,0,0],"quality":"Less is better","isMain":true,"metricName":"Misassembled contigs length"},{"values":[0,0,0,0,0,2,0,0,0,1,0,0,0,0],"quality":"Less is better","isMain":false,"metricName":"# local misassemblies"},{"values":[0,0,0,0,0,0,0,0,0,0,0,0,0,0],"quality":"Less is better","isMain":false,"metricName":"# unaligned mis. contigs"}]],["Unaligned",[{"values":[0,0,0,0,0,0,0,0,0,0,0,0,0,0],"quality":"Less is better","isMain":false,"metricName":"# fully unaligned contigs"},{"values":[0,0,0,0,0,0,0,0,0,0,0,0,0,0],"quality":"Less is better","isMain":false,"metricName":"Fully unaligned length"},{"values":[0,0,0,0,0,2,0,1,1,1,0,0,0,1],"quality":"Less is better","isMain":false,"metricName":"# partially unaligned contigs"},{"values":[0,0,0,0,0,152415,0,2569,720,894,0,0,0,22770],"quality":"Less is better","isMain":false,"metricName":"Partially unaligned length"}]],["Mismatches",[{"values":[4,0,31,12,10,183,0,0,27,22,6,6,2,43],"quality":"Less is better","isMain":false,"metricName":"# mismatches"},{"values":[0,0,0,2,0,7,0,3,2,1,0,0,0,1],"quality":"Less is better","isMain":false,"metricName":"# indels"},{"values":[0,0,0,2,0,7,0,158,2,1,0,0,0,1],"quality":"Less is better","isMain":false,"metricName":"Indels length"},{"values":["17.32","0.00","159.11","53.45","38.42","449.16","0.00","0.00","111.86","175.65","32.50","32.50","16.22","439.04"],"quality":"Less is better","isMain":true,"metricName":"# mismatches per 100 kbp"},{"values":["0.00","0.00","0.00","8.91","0.00","17.18","0.00","31.19","8.29","7.98","0.00","0.00","0.00","10.21"],"quality":"Less is better","isMain":true,"metricName":"# indels per 100 kbp"},{"values":[0,0,0,2,0,7,0,0,2,1,0,0,0,1],"quality":"Less is better","isMain":false,"metricName":" # indels (<= 5 bp)"},{"values":[0,0,0,0,0,0,0,3,0,0,0,0,0,0],"quality":"Less is better","isMain":false,"metricName":" # indels (> 5 bp)"},{"values":[0,0,0,0,0,24,0,1035,0,0,0,0,0,48],"quality":"Less is better","isMain":false,"metricName":"# N's"},{"values":["0.00","0.00","0.00","0.00","0.00","12.00","0.00","8070.80","0.00","0.00","0.00","0.00","0.00","147.41"],"quality":"Less is better","isMain":true,"metricName":"# N's per 100 kbp"}]],["Statistics without reference",[{"values":[22,13,15,21,20,31,2,12,13,6,15,15,10,3],"quality":"Equal","isMain":true,"metricName":"# contigs"},{"values":[8,3,9,6,8,23,0,4,9,4,7,7,5,3],"quality":"Equal","isMain":false,"metricName":"# contigs (>= 1000 bp)"},{"values":[0,0,0,0,1,2,0,0,1,0,0,0,0,1],"quality":"Equal","isMain":false,"metricName":"# contigs (>= 5000 bp)"},{"values":[0,0,0,0,0,1,0,0,0,0,0,0,0,1],"quality":"Equal","isMain":false,"metricName":"# contigs (>= 10000 bp)"},{"values":[0,0,0,0,0,1,0,0,0,0,0,0,0,1],"quality":"Equal","isMain":false,"metricName":"# contigs (>= 25000 bp)"},{"values":[0,0,0,0,0,1,0,0,0,0,0,0,0,0],"quality":"Equal","isMain":false,"metricName":"# contigs (>= 50000 bp)"},{"values":[3355,1901,2406,4131,7534,153388,682,3422,8006,3930,3874,3874,3851,26427],"quality":"More is better","isMain":true,"metricName":"Largest contig"},{"values":[23137,10989,19635,22451,26212,200046,1312,12824,24859,13420,18510,18510,12382,32563],"quality":"More is better","isMain":true,"metricName":"Total length"},{"values":[13943,4454,14682,12257,17461,193768,0,7508,22070,11930,12853,12853,8894,32563],"quality":"More is better","isMain":true,"metricName":"Total length (>= 1000 bp)"},{"values":[0,0,0,0,7534,158692,0,0,8006,0,0,0,0,26427],"quality":"More is better","isMain":false,"metricName":"Total length (>= 5000 bp)"},{"values":[0,0,0,0,0,153388,0,0,0,0,0,0,0,26427],"quality":"More is better","isMain":true,"metricName":"Total length (>= 10000 bp)"},{"values":[0,0,0,0,0,153388,0,0,0,0,0,0,0,26427],"quality":"More is better","isMain":false,"metricName":"Total length (>= 25000 bp)"},{"values":[0,0,0,0,0,153388,0,0,0,0,0,0,0,0],"quality":"More is better","isMain":true,"metricName":"Total length (>= 50000 bp)"},{"values":[1263,930,1477,1204,1309,153388,682,1243,2306,3419,1387,1387,1367,26427],"quality":"More is better","isMain":false,"metricName":"N50"},{"values":[692,613,995,750,909,153388,630,705,1387,3194,931,931,792,26427],"quality":"More is better","isMain":false,"metricName":"N75"},{"values":[6,5,6,6,5,1,1,3,3,2,5,5,3,1],"quality":"Less is better","isMain":false,"metricName":"L50"},{"values":[13,8,10,12,11,1,2,7,7,3,9,9,6,1],"quality":"Less is better","isMain":false,"metricName":"L75"},{"values":["50.28","50.52","49.92","49.95","50.43","50.74","38.34","45.88","50.03","49.28","50.05","50.05","49.59","50.39"],"quality":"Equal","isMain":false,"metricName":"GC (%)"}]],["Predicted genes",[]],["Similarity statistics",[{"values":[0,0,0,0,0,0,0,0,0,0,0,0,0,0],"quality":"Equal","isMain":false,"metricName":"# similar correct contigs"},{"values":[0,0,0,0,0,0,0,0,0,0,0,0,0,0],"quality":"Equal","isMain":false,"metricName":"# similar misassembled blocks"}]],["Reference statistics",[{"values":[60238,60238,60238,60238,60238,60238,60238,60238,60238,60238,60238,60238,60238,60238],"quality":"Equal","isMain":false,"metricName":"Reference length"},{"values":[1,1,1,1,1,1,1,1,1,1,1,1,1,1],"quality":"Equal","isMain":false,"metricName":"Reference fragments"},{"values":["49.07","49.07","49.07","49.07","49.07","49.07","49.07","49.07","49.07","49.07","49.07","49.07","49.07","49.07"],"quality":"Equal","isMain":false,"metricName":"Reference GC (%)"}]]],"referenceName":"gi\_116221992\_ref\_NC","date":"18 July 2018, Wednesday, 16:09:42","order":[0,1,2,3,4,5,6,7,8,9,10,11,12,13],"assembliesNames":["ABySS\_127","ABySS\_63","CLC","IDBA\_UD","MEGAHIT","MIRA","Ray\_Meta","SOAPdenovo2","SPAdes","SPAdes\_meta","SPAdes\_sc","SPAdes\_sc\_careful","Velvet","Geneious"]},{"assembliesWithNs":null,"minContig":500,"report":[["Genome statistics",[{"values":["99.679","92.646","95.437","93.156","88.644","100.000","93.445","2.564","97.620","99.006","95.115","86.138","46.783","100.000"],"quality":"More is better","isMain":true,"metricName":"Genome fraction (%)"},{"values":["1.039","1.015","1.010","1.043","1.067","1.844","1.134","1.121","1.050","1.013","1.030","1.024","1.003","1.730"],"quality":"Less is better","isMain":true,"metricName":"Duplication ratio"},{"values":[18611,25403,16673,6770,8156,81430,7501,749,13139,24149,8302,7311,3244,81430],"quality":"More is better","isMain":true,"metricName":"Largest alignment"},{"values":[171874,155959,159961,161118,156869,305566,175643,4254,170069,165626,162150,146272,77833,287014],"quality":"More is better","isMain":true,"metricName":"Total aligned length"},{"values":[9253,4083,6721,1873,2421,167653,2816,null,5296,11824,2371,1530,null,171313],"quality":"More is better","isMain":false,"metricName":"NG50"},{"values":[5010,1872,3045,1179,1233,167653,1955,null,2084,6810,1047,785,null,171313],"quality":"More is better","isMain":false,"metricName":"NG75"},{"values":[9021,4163,6721,1896,2452,17862,2758,608,5085,11295,2371,1838,878,26703],"quality":"More is better","isMain":false,"metricName":"NA50"},{"values":[4709,2068,3047,1210,1338,4878,1829,502,1840,6637,1125,920,671,11496],"quality":"More is better","isMain":false,"metricName":"NA75"},{"values":[9253,4083,6721,1873,2321,33704,2811,null,5296,11295,2371,1390,null,48260],"quality":"More is better","isMain":true,"metricName":"NGA50"},{"values":[5010,1872,3045,1179,1204,26703,1955,null,1984,6637,1046,755,null,48260],"quality":"More is better","isMain":false,"metricName":"NGA75"},{"values":[6,11,8,28,20,1,20,null,10,6,19,27,null,1],"quality":"Less is better","isMain":false,"metricName":"LG50"},{"values":[12,28,18,56,45,1,38,null,22,11,47,66,null,1],"quality":"Less is better","isMain":false,"metricName":"LG75"},{"values":[7,10,8,27,20,5,22,4,11,6,19,23,30,4],"quality":"Less is better","isMain":false,"metricName":"LA50"},{"values":[13,24,17,53,43,12,42,6,25,11,46,52,56,8],"quality":"Less is better","isMain":false,"metricName":"LA75"},{"values":[6,11,8,28,22,2,20,null,10,6,19,28,null,2],"quality":"Less is better","isMain":true,"metricName":"LGA50"},{"values":[12,28,18,56,47,3,38,null,23,11,48,68,null,2],"quality":"Less is better","isMain":false,"metricName":"LGA75"}]],["Misassemblies",[{"values":[0,0,0,0,1,7,0,0,0,0,0,0,0,9],"quality":"Less is better","isMain":true,"metricName":"# misassemblies"},{"values":[0,0,0,0,1,7,0,0,0,0,0,0,0,9],"quality":"Less is better","isMain":false,"metricName":" # relocations"},{"values":[0,0,0,0,0,0,0,0,0,0,0,0,0,0],"quality":"Less is better","isMain":false,"metricName":" # translocations"},{"values":[0,0,0,0,0,0,0,0,0,0,0,0,0,0],"quality":"Less is better","isMain":false,"metricName":" # inversions"},{"values":[0,0,0,0,1,4,0,0,0,0,0,0,0,2],"quality":"Less is better","isMain":false,"metricName":"# misassembled contigs"},{"values":[0,0,0,0,11039,279796,0,0,0,0,0,0,0,340604],"quality":"Less is better","isMain":true,"metricName":"Misassembled contigs length"},{"values":[0,0,0,0,0,19,1,0,0,1,1,0,0,25],"quality":"Less is better","isMain":false,"metricName":"# local misassemblies"},{"values":[0,0,0,0,0,0,0,0,0,0,0,0,0,0],"quality":"Less is better","isMain":false,"metricName":"# unaligned mis. contigs"}]],["Unaligned",[{"values":[0,0,0,0,0,0,0,0,0,0,0,0,0,0],"quality":"Less is better","isMain":false,"metricName":"# fully unaligned contigs"},{"values":[0,0,0,0,0,0,0,0,0,0,0,0,0,0],"quality":"Less is better","isMain":false,"metricName":"Fully unaligned length"},{"values":[0,0,0,0,1,4,0,0,2,2,1,2,0,2],"quality":"Less is better","isMain":false,"metricName":"# partially unaligned contigs"},{"values":[0,0,0,0,1117,45531,0,0,1407,1384,847,1904,0,53590],"quality":"Less is better","isMain":false,"metricName":"Partially unaligned length"}]],["Mismatches",[{"values":[2,29,9,11,279,1086,199,0,4,131,126,41,53,1443],"quality":"Less is better","isMain":false,"metricName":"# mismatches"},{"values":[0,0,0,0,8,32,8,4,0,8,3,3,2,46],"quality":"Less is better","isMain":false,"metricName":"# indels"},{"values":[0,0,0,0,13,37,12,204,0,20,52,52,2,80],"quality":"Less is better","isMain":false,"metricName":"Indels length"},{"values":["1.21","18.87","5.68","7.12","189.73","654.65","128.37","0.00","2.47","79.76","79.85","28.69","68.29","869.85"],"quality":"Less is better","isMain":true,"metricName":"# mismatches per 100 kbp"},{"values":["0.00","0.00","0.00","0.00","5.44","19.29","5.16","94.03","0.00","4.87","1.90","2.10","2.58","27.73"],"quality":"Less is better","isMain":true,"metricName":"# indels per 100 kbp"},{"values":[0,0,0,0,7,32,8,0,0,7,2,2,2,43],"quality":"Less is better","isMain":false,"metricName":" # indels (<= 5 bp)"},{"values":[0,0,0,0,1,0,0,4,0,1,1,1,0,3],"quality":"Less is better","isMain":false,"metricName":" # indels (> 5 bp)"},{"values":[0,17,0,0,0,14,0,515,1407,2085,1238,1904,0,253],"quality":"Less is better","isMain":false,"metricName":"# N's"},{"values":["0.00","10.90","0.00","0.00","0.00","3.98","0.00","10798.91","820.52","1243.33","757.63","1284.68","0.00","74.28"],"quality":"Less is better","isMain":true,"metricName":"# N's per 100 kbp"}]],["Statistics without reference",[{"values":[28,65,44,102,86,28,81,7,69,28,97,103,89,2],"quality":"Equal","isMain":true,"metricName":"# contigs"},{"values":[27,32,35,65,53,18,65,0,43,21,49,47,19,2],"quality":"Equal","isMain":false,"metricName":"# contigs (>= 1000 bp)"},{"values":[12,7,11,3,4,6,6,0,11,13,6,3,0,2],"quality":"Equal","isMain":false,"metricName":"# contigs (>= 5000 bp)"},{"values":[5,1,4,0,1,6,0,0,3,6,0,0,0,2],"quality":"Equal","isMain":false,"metricName":"# contigs (>= 10000 bp)"},{"values":[0,1,0,0,0,4,0,0,0,0,0,0,0,2],"quality":"Equal","isMain":false,"metricName":"# contigs (>= 25000 bp)"},{"values":[0,0,0,0,0,2,0,0,0,0,0,0,0,2],"quality":"Equal","isMain":false,"metricName":"# contigs (>= 50000 bp)"},{"values":[18611,25403,16673,6770,11039,167653,7501,967,13139,24149,8302,7311,3244,171313],"quality":"More is better","isMain":true,"metricName":"Largest contig"},{"values":[171874,155959,159961,161118,157988,351354,175839,4769,171476,167695,163405,148208,77834,340604],"quality":"More is better","isMain":true,"metricName":"Total length"},{"values":[171354,132757,153957,134679,133310,344425,163168,0,153963,162330,127368,108393,28851,340604],"quality":"More is better","isMain":true,"metricName":"Total length (>= 1000 bp)"},{"values":[127404,66787,101424,18472,33535,311543,36425,0,89821,140930,39730,18854,0,340604],"quality":"More is better","isMain":false,"metricName":"Total length (>= 5000 bp)"},{"values":[75975,25403,54325,0,11039,311543,0,0,36498,91348,0,0,0,340604],"quality":"More is better","isMain":true,"metricName":"Total length (>= 10000 bp)"},{"values":[0,25403,0,0,0,279796,0,0,0,0,0,0,0,340604],"quality":"More is better","isMain":false,"metricName":"Total length (>= 25000 bp)"},{"values":[0,0,0,0,0,221525,0,0,0,0,0,0,0,340604],"quality":"More is better","isMain":true,"metricName":"Total length (>= 50000 bp)"},{"values":[9021,4163,6721,1896,2452,53872,2763,718,5085,11824,2423,1892,878,171313],"quality":"More is better","isMain":false,"metricName":"N50"},{"values":[4709,2068,3047,1210,1342,28058,1840,544,1840,6810,1131,941,671,169291],"quality":"More is better","isMain":false,"metricName":"N75"},{"values":[7,10,8,27,19,2,22,3,11,6,18,22,30,1],"quality":"Less is better","isMain":false,"metricName":"L50"},{"values":[13,24,17,53,41,4,42,5,25,11,45,50,56,2],"quality":"Less is better","isMain":false,"metricName":"L75"},{"values":["35.36","35.34","35.37","35.40","35.12","35.46","35.29","34.88","35.31","35.30","35.37","35.41","35.26","35.32"],"quality":"Equal","isMain":false,"metricName":"GC (%)"}]],["Predicted genes",[]],["Similarity statistics",[{"values":[0,0,0,0,0,0,0,0,0,0,0,0,0,0],"quality":"Equal","isMain":false,"metricName":"# similar correct contigs"},{"values":[0,0,0,0,0,0,0,0,0,0,0,0,0,0],"quality":"Equal","isMain":false,"metricName":"# similar misassembled blocks"}]],["Reference statistics",[{"values":[165890,165890,165890,165890,165890,165890,165890,165890,165890,165890,165890,165890,165890,165890],"quality":"Equal","isMain":false,"metricName":"Reference length"},{"values":[1,1,1,1,1,1,1,1,1,1,1,1,1,1],"quality":"Equal","isMain":false,"metricName":"Reference fragments"},{"values":["35.34","35.34","35.34","35.34","35.34","35.34","35.34","35.34","35.34","35.34","35.34","35.34","35.34","35.34"],"quality":"Equal","isMain":false,"metricName":"Reference GC (%)"}]]],"referenceName":"gi\_116326222\_ref\_NC","date":"18 July 2018, Wednesday, 16:09:57","order":[0,1,2,3,4,5,6,7,8,9,10,11,12,13],"assembliesNames":["ABySS\_127","ABySS\_63","CLC","IDBA\_UD","MEGAHIT","MIRA","Ray\_Meta","SOAPdenovo2","SPAdes","SPAdes\_meta","SPAdes\_sc","SPAdes\_sc\_careful","Velvet","Geneious"]},{"assembliesWithNs":null,"minContig":500,"report":[["Genome statistics",[{"values":["11.368","9.389","71.051","62.583","67.595","29.429","62.189","76.880","75.162","75.590","75.590","4.523","6.853"],"quality":"More is better","isMain":true,"metricName":"Genome fraction (%)"},{"values":["1.000","1.000","1.012","1.000","1.001","1.027","1.002","1.002","1.005","1.002","1.002","1.000","1.000"],"quality":"Less is better","isMain":true,"metricName":"Duplication ratio"},{"values":[2257,1547,5191,5789,5789,3341,2991,5937,5937,5387,5387,1749,4203],"quality":"More is better","isMain":true,"metricName":"Largest alignment"},{"values":[19787,16343,124587,108966,117794,52595,108478,133889,131452,131770,131770,7872,11928],"quality":"More is better","isMain":true,"metricName":"Total aligned length"},{"values":[null,null,973,879,834,null,762,1380,1266,1325,1325,null,null],"quality":"More is better","isMain":false,"metricName":"NG50"},{"values":[null,null,null,null,null,null,null,599,563,563,563,null,null],"quality":"More is better","isMain":false,"metricName":"NG75"},{"values":[796,725,1437,1505,1492,1145,1202,1630,1589,1644,1644,1188,null],"quality":"More is better","isMain":false,"metricName":"NA50"},{"values":[639,599,854,1037,821,953,841,1037,990,1037,1037,686,null],"quality":"More is better","isMain":false,"metricName":"NA75"},{"values":[null,null,973,879,834,null,762,1351,1266,1292,1292,null,null],"quality":"More is better","isMain":true,"metricName":"NGA50"},{"values":[null,null,null,null,null,null,null,592,563,563,563,null,null],"quality":"More is better","isMain":false,"metricName":"NGA75"},{"values":[null,null,47,52,50,null,66,40,41,40,40,null,null],"quality":"Less is better","isMain":false,"metricName":"LG50"},{"values":[null,null,null,null,null,null,null,88,94,92,92,null,null],"quality":"Less is better","isMain":false,"metricName":"LG75"},{"values":[9,8,27,24,26,17,32,27,26,26,26,3,null],"quality":"Less is better","isMain":false,"metricName":"LA50"},{"values":[17,14,55,46,51,29,59,53,52,51,51,5,null],"quality":"Less is better","isMain":false,"metricName":"LA75"},{"values":[null,null,47,52,50,null,66,40,41,41,41,null,null],"quality":"Less is better","isMain":true,"metricName":"LGA50"},{"values":[null,null,null,null,null,null,null,90,95,92,92,null,null],"quality":"Less is better","isMain":false,"metricName":"LGA75"}]],["Misassemblies",[{"values":[0,0,1,0,0,0,0,0,1,0,0,0,0],"quality":"Less is better","isMain":true,"metricName":"# misassemblies"},{"values":[0,0,1,0,0,0,0,0,1,0,0,0,0],"quality":"Less is better","isMain":false,"metricName":" # relocations"},{"values":[0,0,0,0,0,0,0,0,0,0,0,0,0],"quality":"Less is better","isMain":false,"metricName":" # translocations"},{"values":[0,0,0,0,0,0,0,0,0,0,0,0,0],"quality":"Less is better","isMain":false,"metricName":" # inversions"},{"values":[0,0,1,0,0,0,0,0,1,0,0,0,0],"quality":"Less is better","isMain":false,"metricName":"# misassembled contigs"},{"values":[0,0,2159,0,0,0,0,0,4928,0,0,0,0],"quality":"Less is better","isMain":true,"metricName":"Misassembled contigs length"},{"values":[0,0,0,0,0,0,0,0,0,0,0,0,0],"quality":"Less is better","isMain":false,"metricName":"# local misassemblies"},{"values":[0,0,0,0,0,0,0,0,0,0,0,0,0],"quality":"Less is better","isMain":false,"metricName":"# unaligned mis. contigs"}]],["Unaligned",[{"values":[0,0,0,0,0,0,0,0,0,0,0,0,0],"quality":"Less is better","isMain":false,"metricName":"# fully unaligned contigs"},{"values":[0,0,0,0,0,0,0,0,0,0,0,0,0],"quality":"Less is better","isMain":false,"metricName":"Fully unaligned length"},{"values":[0,0,0,0,0,0,0,1,0,0,0,0,2],"quality":"Less is better","isMain":false,"metricName":"# partially unaligned contigs"},{"values":[0,0,0,0,0,0,0,1580,0,0,0,0,25262],"quality":"Less is better","isMain":false,"metricName":"Partially unaligned length"}]],["Mismatches",[{"values":[2,0,136,97,91,22,82,192,174,143,116,2,0],"quality":"Less is better","isMain":false,"metricName":"# mismatches"},{"values":[0,0,0,0,0,0,1,1,0,0,0,0,0],"quality":"Less is better","isMain":false,"metricName":"# indels"},{"values":[0,0,0,0,0,0,46,48,0,0,0,0,0],"quality":"Less is better","isMain":false,"metricName":"Indels length"},{"values":["10.11","0.00","109.97","89.05","77.34","42.95","75.75","143.48","133.00","108.69","88.16","25.41","0.00"],"quality":"Less is better","isMain":true,"metricName":"# mismatches per 100 kbp"},{"values":["0.00","0.00","0.00","0.00","0.00","0.00","0.92","0.75","0.00","0.00","0.00","0.00","0.00"],"quality":"Less is better","isMain":true,"metricName":"# indels per 100 kbp"},{"values":[0,0,0,0,0,0,0,0,0,0,0,0,0],"quality":"Less is better","isMain":false,"metricName":" # indels (<= 5 bp)"},{"values":[0,0,0,0,0,0,1,1,0,0,0,0,0],"quality":"Less is better","isMain":false,"metricName":" # indels (> 5 bp)"},{"values":[0,0,0,0,0,78,60,0,0,0,0,0,39],"quality":"Less is better","isMain":false,"metricName":"# N's"},{"values":["0.00","0.00","0.00","0.00","0.00","148.30","55.31","0.00","0.00","0.00","0.00","0.00","104.87"],"quality":"Less is better","isMain":true,"metricName":"# N's per 100 kbp"}]],["Statistics without reference",[{"values":[25,21,99,83,95,47,99,96,95,94,94,8,5],"quality":"Equal","isMain":true,"metricName":"# contigs"},{"values":[3,4,45,47,42,24,44,55,50,52,52,3,5],"quality":"Equal","isMain":false,"metricName":"# contigs (>= 1000 bp)"},{"values":[0,0,1,1,1,0,0,1,1,1,1,0,1],"quality":"Equal","isMain":false,"metricName":"# contigs (>= 5000 bp)"},{"values":[0,0,0,0,0,0,0,0,0,0,0,0,1],"quality":"Equal","isMain":false,"metricName":"# contigs (>= 10000 bp)"},{"values":[0,0,0,0,0,0,0,0,0,0,0,0,1],"quality":"Equal","isMain":false,"metricName":"# contigs (>= 25000 bp)"},{"values":[0,0,0,0,0,0,0,0,0,0,0,0,0],"quality":"Equal","isMain":false,"metricName":"# contigs (>= 50000 bp)"},{"values":[2257,1547,5191,5789,5789,3341,2991,5937,5937,5387,5387,1749,28523],"quality":"More is better","isMain":true,"metricName":"Largest contig"},{"values":[19787,16343,125157,108982,117796,52595,108478,135631,131452,131843,131843,7872,37190],"quality":"More is better","isMain":true,"metricName":"Total length"},{"values":[4822,5101,85885,82816,80102,34585,68546,105596,98068,100854,100854,4563,37190],"quality":"More is better","isMain":true,"metricName":"Total length (>= 1000 bp)"},{"values":[0,0,5191,5789,5789,0,0,5937,5937,5387,5387,0,28523],"quality":"More is better","isMain":false,"metricName":"Total length (>= 5000 bp)"},{"values":[0,0,0,0,0,0,0,0,0,0,0,0,28523],"quality":"More is better","isMain":true,"metricName":"Total length (>= 10000 bp)"},{"values":[0,0,0,0,0,0,0,0,0,0,0,0,28523],"quality":"More is better","isMain":false,"metricName":"Total length (>= 25000 bp)"},{"values":[0,0,0,0,0,0,0,0,0,0,0,0,0],"quality":"More is better","isMain":true,"metricName":"Total length (>= 50000 bp)"},{"values":[796,725,1437,1505,1492,1145,1202,1644,1589,1644,1644,1188,28523],"quality":"More is better","isMain":false,"metricName":"N50"},{"values":[639,599,870,1037,821,953,841,1082,990,1037,1037,686,28523],"quality":"More is better","isMain":false,"metricName":"N75"},{"values":[9,8,27,24,26,17,32,27,26,26,26,3,1],"quality":"Less is better","isMain":false,"metricName":"L50"},{"values":[17,14,54,46,51,29,59,52,51,51,51,5,1],"quality":"Less is better","isMain":false,"metricName":"L75"},{"values":["36.38","35.61","35.22","35.32","35.28","35.58","34.97","35.31","35.22","35.21","35.21","34.20","39.29"],"quality":"Equal","isMain":false,"metricName":"GC (%)"}]],["Predicted genes",[]],["Similarity statistics",[{"values":[0,0,0,0,0,0,0,0,0,0,0,0,0],"quality":"Equal","isMain":false,"metricName":"# similar correct contigs"},{"values":[0,0,0,0,0,0,0,0,0,0,0,0,0],"quality":"Equal","isMain":false,"metricName":"# similar misassembled blocks"}]],["Reference statistics",[{"values":[174059,174059,174059,174059,174059,174059,174059,174059,174059,174059,174059,174059,174059],"quality":"Equal","isMain":false,"metricName":"Reference length"},{"values":[1,1,1,1,1,1,1,1,1,1,1,1,1],"quality":"Equal","isMain":false,"metricName":"Reference fragments"},{"values":["35.23","35.23","35.23","35.23","35.23","35.23","35.23","35.23","35.23","35.23","35.23","35.23","35.23"],"quality":"Equal","isMain":false,"metricName":"Reference GC (%)"}]]],"referenceName":"gi\_116326687\_ref\_NC","date":"18 July 2018, Wednesday, 16:10:09","order":[0,1,2,3,4,5,6,7,8,9,10,11,12],"assembliesNames":["ABySS\_127","ABySS\_63","CLC","IDBA\_UD","MEGAHIT","MIRA","SOAPdenovo2","SPAdes","SPAdes\_meta","SPAdes\_sc","SPAdes\_sc\_careful","Velvet","Geneious"]},{"assembliesWithNs":null,"minContig":500,"report":[["Genome statistics",[{"values":["95.449","92.019","99.468","99.688","99.697","97.950","97.499","99.722","99.924","99.827","99.803","99.474","89.002"],"quality":"More is better","isMain":true,"metricName":"Genome fraction (%)"},{"values":["1.002","1.001","1.009","1.002","1.002","1.011","1.018","1.002","1.000","0.999","1.000","1.001","1.001"],"quality":"Less is better","isMain":true,"metricName":"Duplication ratio"},{"values":[7915,8093,39627,104015,54462,31005,38886,104015,104249,104249,104249,47498,41621],"quality":"More is better","isMain":true,"metricName":"Largest alignment"},{"values":[155009,149324,162370,161646,161653,160408,158503,161668,161944,161729,161745,161351,144280],"quality":"More is better","isMain":true,"metricName":"Total aligned length"},{"values":[2982,2855,31670,104300,49581,18565,21819,104300,104249,104249,104249,22465,22466],"quality":"More is better","isMain":false,"metricName":"NG50"},{"values":[1752,1502,22779,18568,24324,16984,7466,33275,33317,33102,33118,22146,18232],"quality":"More is better","isMain":false,"metricName":"NG75"},{"values":[3149,3085,31385,104015,49581,18565,21524,104015,104249,104249,104249,22465,31005],"quality":"More is better","isMain":false,"metricName":"NA50"},{"values":[1826,1715,22779,18568,24324,16984,7315,33275,33317,33102,33118,22146,18565],"quality":"More is better","isMain":false,"metricName":"NA75"},{"values":[2982,2855,31385,104015,49581,18565,21524,104015,104249,104249,104249,22465,22466],"quality":"More is better","isMain":true,"metricName":"NGA50"},{"values":[1752,1502,22779,18568,24324,16984,7026,33275,33317,33102,33118,22146,18232],"quality":"More is better","isMain":false,"metricName":"NGA75"},{"values":[17,18,3,1,2,4,3,1,1,1,1,3,3],"quality":"Less is better","isMain":false,"metricName":"LG50"},{"values":[35,38,4,2,3,6,7,2,2,2,2,4,5],"quality":"Less is better","isMain":false,"metricName":"LG75"},{"values":[16,16,3,1,2,4,3,1,1,1,1,3,2],"quality":"Less is better","isMain":false,"metricName":"LA50"},{"values":[32,32,4,2,3,6,7,2,2,2,2,4,4],"quality":"Less is better","isMain":false,"metricName":"LA75"},{"values":[17,18,3,1,2,4,3,1,1,1,1,3,3],"quality":"Less is better","isMain":true,"metricName":"LGA50"},{"values":[35,38,4,2,3,6,8,2,2,2,2,4,5],"quality":"Less is better","isMain":false,"metricName":"LGA75"}]],["Misassemblies",[{"values":[0,0,0,0,0,0,0,0,0,0,0,0,0],"quality":"Less is better","isMain":true,"metricName":"# misassemblies"},{"values":[0,0,0,0,0,0,0,0,0,0,0,0,0],"quality":"Less is better","isMain":false,"metricName":" # relocations"},{"values":[0,0,0,0,0,0,0,0,0,0,0,0,0],"quality":"Less is better","isMain":false,"metricName":" # translocations"},{"values":[0,0,0,0,0,0,0,0,0,0,0,0,0],"quality":"Less is better","isMain":false,"metricName":" # inversions"},{"values":[0,0,0,0,0,0,0,0,0,0,0,0,0],"quality":"Less is better","isMain":false,"metricName":"# misassembled contigs"},{"values":[0,0,0,0,0,0,0,0,0,0,0,0,0],"quality":"Less is better","isMain":true,"metricName":"Misassembled contigs length"},{"values":[0,0,0,0,0,0,0,0,0,0,1,0,0],"quality":"Less is better","isMain":false,"metricName":"# local misassemblies"},{"values":[0,0,0,0,0,0,0,0,0,0,0,0,0],"quality":"Less is better","isMain":false,"metricName":"# unaligned mis. contigs"}]],["Unaligned",[{"values":[0,0,0,0,0,0,0,0,0,0,0,0,0],"quality":"Less is better","isMain":false,"metricName":"# fully unaligned contigs"},{"values":[0,0,0,0,0,0,0,0,0,0,0,0,0],"quality":"Less is better","isMain":false,"metricName":"Fully unaligned length"},{"values":[0,0,0,0,0,0,0,0,0,0,0,0,0],"quality":"Less is better","isMain":false,"metricName":"# partially unaligned contigs"},{"values":[0,0,0,0,0,0,0,0,0,0,0,0,0],"quality":"Less is better","isMain":false,"metricName":"Partially unaligned length"}]],["Mismatches",[{"values":[17,5,6,5,6,0,6,21,13,9,16,15,0],"quality":"Less is better","isMain":false,"metricName":"# mismatches"},{"values":[0,1,2,0,0,1,31,1,1,4,9,8,1],"quality":"Less is better","isMain":false,"metricName":"# indels"},{"values":[0,51,2,0,0,42,1479,42,51,269,130,82,42],"quality":"Less is better","isMain":false,"metricName":"Indels length"},{"values":["10.99","3.35","3.72","3.09","3.71","0.00","3.80","12.99","8.03","5.56","9.89","9.30","0.00"],"quality":"Less is better","isMain":true,"metricName":"# mismatches per 100 kbp"},{"values":["0.00","0.67","1.24","0.00","0.00","0.63","19.61","0.62","0.62","2.47","5.56","4.96","0.69"],"quality":"Less is better","isMain":true,"metricName":"# indels per 100 kbp"},{"values":[0,0,2,0,0,0,0,0,0,0,7,7,0],"quality":"Less is better","isMain":false,"metricName":" # indels (<= 5 bp)"},{"values":[0,1,0,0,0,1,31,1,1,4,2,1,1],"quality":"Less is better","isMain":false,"metricName":" # indels (> 5 bp)"},{"values":[0,0,0,0,0,41,2817,0,0,10,0,0,33],"quality":"Less is better","isMain":false,"metricName":"# N's"},{"values":["0.00","0.00","0.00","0.00","0.00","25.54","1751.59","0.00","0.00","6.18","0.00","0.00","22.85"],"quality":"Less is better","isMain":true,"metricName":"# N's per 100 kbp"}]],["Statistics without reference",[{"values":[67,67,13,5,5,17,17,3,3,3,3,11,7],"quality":"Equal","isMain":true,"metricName":"# contigs"},{"values":[51,49,11,5,5,15,16,3,3,3,3,10,7],"quality":"Equal","isMain":false,"metricName":"# contigs (>= 1000 bp)"},{"values":[6,6,6,5,5,7,11,3,3,3,3,7,7],"quality":"Equal","isMain":false,"metricName":"# contigs (>= 5000 bp)"},{"values":[0,0,5,4,5,6,3,3,3,3,3,5,5],"quality":"Equal","isMain":false,"metricName":"# contigs (>= 10000 bp)"},{"values":[0,0,3,1,2,1,2,2,2,2,2,2,2],"quality":"Equal","isMain":false,"metricName":"# contigs (>= 25000 bp)"},{"values":[0,0,0,1,1,0,0,1,1,1,1,0,0],"quality":"Equal","isMain":false,"metricName":"# contigs (>= 50000 bp)"},{"values":[7915,8093,39627,104300,54718,31143,39085,104300,104249,104249,104249,47498,41621],"quality":"More is better","isMain":true,"metricName":"Largest contig"},{"values":[155009,149324,162655,161931,161909,160546,160825,161953,161944,161729,161745,161351,144418],"quality":"More is better","isMain":true,"metricName":"Total length"},{"values":[143613,136342,161038,161931,161909,159327,160301,161953,161944,161729,161745,160766,144418],"quality":"More is better","isMain":true,"metricName":"Total length (>= 1000 bp)"},{"values":[40139,39457,146782,161931,161909,137783,147848,161953,161944,161729,161745,155279,144418],"quality":"More is better","isMain":false,"metricName":"Total length (>= 5000 bp)"},{"values":[0,0,140917,152083,161909,132194,91911,161953,161944,161729,161745,138963,132027],"quality":"More is better","isMain":true,"metricName":"Total length (>= 10000 bp)"},{"values":[0,0,103564,104300,104299,31143,70092,137575,137566,137351,137367,79770,72764],"quality":"More is better","isMain":false,"metricName":"Total length (>= 25000 bp)"},{"values":[0,0,0,104300,54718,0,0,104300,104249,104249,104249,0,0],"quality":"More is better","isMain":true,"metricName":"Total length (>= 50000 bp)"},{"values":[3149,3085,31670,104300,49581,18565,21819,104300,104249,104249,104249,22465,31143],"quality":"More is better","isMain":false,"metricName":"N50"},{"values":[1826,1715,22779,18568,24324,16984,7466,33275,33317,33102,33118,22146,18565],"quality":"More is better","isMain":false,"metricName":"N75"},{"values":[16,16,3,1,2,4,3,1,1,1,1,3,2],"quality":"Less is better","isMain":false,"metricName":"L50"},{"values":[32,32,4,2,3,6,7,2,2,2,2,4,4],"quality":"Less is better","isMain":false,"metricName":"L75"},{"values":["45.94","45.95","45.91","45.95","45.96","46.00","46.05","45.95","45.96","45.94","45.95","45.96","46.24"],"quality":"Equal","isMain":false,"metricName":"GC (%)"}]],["Predicted genes",[]],["Similarity statistics",[{"values":[0,0,1,1,1,1,0,1,0,1,0,1,1],"quality":"Equal","isMain":false,"metricName":"# similar correct contigs"},{"values":[0,0,0,0,0,0,0,0,0,0,0,0,0],"quality":"Equal","isMain":false,"metricName":"# similar misassembled blocks"}]],["Reference statistics",[{"values":[162109,162109,162109,162109,162109,162109,162109,162109,162109,162109,162109,162109,162109],"quality":"Equal","isMain":false,"metricName":"Reference length"},{"values":[1,1,1,1,1,1,1,1,1,1,1,1,1],"quality":"Equal","isMain":false,"metricName":"Reference fragments"},{"values":["45.95","45.95","45.95","45.95","45.95","45.95","45.95","45.95","45.95","45.95","45.95","45.95","45.95"],"quality":"Equal","isMain":false,"metricName":"Reference GC (%)"}]]],"referenceName":"gi\_117530171\_ref\_NC","date":"18 July 2018, Wednesday, 16:10:21","order":[0,1,2,3,4,5,6,7,8,9,10,11,12],"assembliesNames":["ABySS\_127","ABySS\_63","CLC","IDBA\_UD","MEGAHIT","MIRA","SOAPdenovo2","SPAdes","SPAdes\_meta","SPAdes\_sc","SPAdes\_sc\_careful","Velvet","Geneious"]},{"assembliesWithNs":null,"minContig":500,"report":[["Genome statistics",[{"values":["29.339","11.733","28.614","20.946","21.430","94.540","18.606","31.988","13.592","20.978","17.652","19.320"],"quality":"More is better","isMain":true,"metricName":"Genome fraction (%)"},{"values":["1.000","1.000","1.000","1.000","1.000","1.504","1.051","1.011","1.000","1.000","1.000","1.000"],"quality":"Less is better","isMain":true,"metricName":"Duplication ratio"},{"values":[3365,3099,3667,3744,3784,19280,3620,3772,2769,2769,2769,3639],"quality":"More is better","isMain":true,"metricName":"Largest alignment"},{"values":[11043,4416,10770,7884,8066,53507,7088,12167,5116,7896,6644,7272],"quality":"More is better","isMain":true,"metricName":"Total aligned length"},{"values":[null,null,null,null,null,40228,null,null,null,null,null,null],"quality":"More is better","isMain":false,"metricName":"NG50"},{"values":[null,null,null,null,null,40228,null,null,null,null,null,null],"quality":"More is better","isMain":false,"metricName":"NG75"},{"values":[1128,3099,2829,2291,2184,15622,2225,2419,2769,2347,2347,3639],"quality":"More is better","isMain":false,"metricName":"NA50"},{"values":[876,746,2274,2291,822,1501,2225,785,2347,931,931,2282],"quality":"More is better","isMain":false,"metricName":"NA75"},{"values":[null,null,null,null,null,19280,null,null,null,null,null,null],"quality":"More is better","isMain":true,"metricName":"NGA50"},{"values":[null,null,null,null,null,15622,null,null,null,null,null,null],"quality":"More is better","isMain":false,"metricName":"NGA75"},{"values":[null,null,null,null,null,1,null,null,null,null,null,null],"quality":"Less is better","isMain":false,"metricName":"LG50"},{"values":[null,null,null,null,null,1,null,null,null,null,null,null],"quality":"Less is better","isMain":false,"metricName":"LG75"},{"values":[3,1,2,2,2,2,2,2,1,2,2,1],"quality":"Less is better","isMain":false,"metricName":"LA50"},{"values":[5,2,3,2,3,6,2,5,2,4,3,2],"quality":"Less is better","isMain":false,"metricName":"LA75"},{"values":[null,null,null,null,null,1,null,null,null,null,null,null],"quality":"Less is better","isMain":true,"metricName":"LGA50"},{"values":[null,null,null,null,null,2,null,null,null,null,null,null],"quality":"Less is better","isMain":false,"metricName":"LGA75"}]],["Misassemblies",[{"values":[0,0,0,0,0,1,0,0,0,0,0,0],"quality":"Less is better","isMain":true,"metricName":"# misassemblies"},{"values":[0,0,0,0,0,1,0,0,0,0,0,0],"quality":"Less is better","isMain":false,"metricName":" # relocations"},{"values":[0,0,0,0,0,0,0,0,0,0,0,0],"quality":"Less is better","isMain":false,"metricName":" # translocations"},{"values":[0,0,0,0,0,0,0,0,0,0,0,0],"quality":"Less is better","isMain":false,"metricName":" # inversions"},{"values":[0,0,0,0,0,1,0,0,0,0,0,0],"quality":"Less is better","isMain":false,"metricName":"# misassembled contigs"},{"values":[0,0,0,0,0,40228,0,0,0,0,0,0],"quality":"Less is better","isMain":true,"metricName":"Misassembled contigs length"},{"values":[0,0,0,0,0,2,0,0,0,0,0,0],"quality":"Less is better","isMain":false,"metricName":"# local misassemblies"},{"values":[0,0,0,0,0,0,0,0,0,0,0,0],"quality":"Less is better","isMain":false,"metricName":"# unaligned mis. contigs"}]],["Unaligned",[{"values":[0,0,0,0,0,0,0,0,0,0,0,0],"quality":"Less is better","isMain":false,"metricName":"# fully unaligned contigs"},{"values":[0,0,0,0,0,0,0,0,0,0,0,0],"quality":"Less is better","isMain":false,"metricName":"Fully unaligned length"},{"values":[0,0,0,0,0,1,0,0,0,1,1,0],"quality":"Less is better","isMain":false,"metricName":"# partially unaligned contigs"},{"values":[0,0,0,0,0,5326,0,0,0,1151,1151,0],"quality":"Less is better","isMain":false,"metricName":"Partially unaligned length"}]],["Mismatches",[{"values":[4,0,1,0,2,484,0,2,0,0,0,3],"quality":"Less is better","isMain":false,"metricName":"# mismatches"},{"values":[0,0,0,0,0,15,4,0,0,0,0,0],"quality":"Less is better","isMain":false,"metricName":"# indels"},{"values":[0,0,0,0,0,24,174,0,0,0,0,0],"quality":"Less is better","isMain":false,"metricName":"Indels length"},{"values":["36.22","0.00","9.29","0.00","24.80","1360.16","0.00","16.61","0.00","0.00","0.00","41.25"],"quality":"Less is better","isMain":true,"metricName":"# mismatches per 100 kbp"},{"values":["0.00","0.00","0.00","0.00","0.00","42.15","57.12","0.00","0.00","0.00","0.00","0.00"],"quality":"Less is better","isMain":true,"metricName":"# indels per 100 kbp"},{"values":[0,0,0,0,0,14,0,0,0,0,0,0],"quality":"Less is better","isMain":false,"metricName":" # indels (<= 5 bp)"},{"values":[0,0,0,0,0,1,4,0,0,0,0,0],"quality":"Less is better","isMain":false,"metricName":" # indels (> 5 bp)"},{"values":[0,0,0,0,0,11,364,0,0,0,0,0],"quality":"Less is better","isMain":false,"metricName":"# N's"},{"values":["0.00","0.00","0.00","0.00","0.00","18.70","4945.65","0.00","0.00","0.00","0.00","0.00"],"quality":"Less is better","isMain":true,"metricName":"# N's per 100 kbp"}]],["Statistics without reference",[{"values":[9,3,6,5,5,13,4,9,2,5,4,3],"quality":"Equal","isMain":true,"metricName":"# contigs"},{"values":[3,1,3,2,2,9,2,3,2,4,3,3],"quality":"Equal","isMain":false,"metricName":"# contigs (>= 1000 bp)"},{"values":[0,0,0,0,0,1,0,0,0,0,0,0],"quality":"Equal","isMain":false,"metricName":"# contigs (>= 5000 bp)"},{"values":[0,0,0,0,0,1,0,0,0,0,0,0],"quality":"Equal","isMain":false,"metricName":"# contigs (>= 10000 bp)"},{"values":[0,0,0,0,0,1,0,0,0,0,0,0],"quality":"Equal","isMain":false,"metricName":"# contigs (>= 25000 bp)"},{"values":[0,0,0,0,0,0,0,0,0,0,0,0],"quality":"Equal","isMain":false,"metricName":"# contigs (>= 50000 bp)"},{"values":[3365,3099,3667,3744,3784,40228,3755,3772,2769,2769,2769,3639],"quality":"More is better","isMain":true,"metricName":"Largest contig"},{"values":[11043,4416,10770,7884,8066,58833,7360,12167,5116,9047,7795,7272],"quality":"More is better","isMain":true,"metricName":"Total length"},{"values":[6587,3099,8770,6035,5968,55658,5980,8033,5116,8506,7198,7272],"quality":"More is better","isMain":true,"metricName":"Total length (>= 1000 bp)"},{"values":[0,0,0,0,0,40228,0,0,0,0,0,0],"quality":"More is better","isMain":false,"metricName":"Total length (>= 5000 bp)"},{"values":[0,0,0,0,0,40228,0,0,0,0,0,0],"quality":"More is better","isMain":true,"metricName":"Total length (>= 10000 bp)"},{"values":[0,0,0,0,0,40228,0,0,0,0,0,0],"quality":"More is better","isMain":false,"metricName":"Total length (>= 25000 bp)"},{"values":[0,0,0,0,0,0,0,0,0,0,0,0],"quality":"More is better","isMain":true,"metricName":"Total length (>= 50000 bp)"},{"values":[1128,3099,2829,2291,2184,40228,3755,2419,2769,2347,2347,3639],"quality":"More is better","isMain":false,"metricName":"N50"},{"values":[876,746,2274,2291,822,2632,2225,785,2347,2082,2082,2282],"quality":"More is better","isMain":false,"metricName":"N75"},{"values":[3,1,2,2,2,1,1,2,1,2,2,1],"quality":"Less is better","isMain":false,"metricName":"L50"},{"values":[5,2,3,2,3,3,2,5,2,3,3,2],"quality":"Less is better","isMain":false,"metricName":"L75"},{"values":["61.18","56.88","61.16","59.16","59.00","64.92","57.00","63.41","55.55","57.06","56.36","59.19"],"quality":"Equal","isMain":false,"metricName":"GC (%)"}]],["Predicted genes",[]],["Similarity statistics",[{"values":[0,0,0,0,0,0,0,0,0,0,0,0],"quality":"Equal","isMain":false,"metricName":"# similar correct contigs"},{"values":[0,0,0,0,0,0,0,0,0,0,0,0],"quality":"Equal","isMain":false,"metricName":"# similar misassembled blocks"}]],["Reference statistics",[{"values":[37639,37639,37639,37639,37639,37639,37639,37639,37639,37639,37639,37639],"quality":"Equal","isMain":false,"metricName":"Reference length"},{"values":[1,1,1,1,1,1,1,1,1,1,1,1],"quality":"Equal","isMain":false,"metricName":"Reference fragments"},{"values":["64.82","64.82","64.82","64.82","64.82","64.82","64.82","64.82","64.82","64.82","64.82","64.82"],"quality":"Equal","isMain":false,"metricName":"Reference GC (%)"}]]],"referenceName":"gi\_117676329\_ref\_NC","date":"18 July 2018, Wednesday, 16:10:31","order":[0,1,2,3,4,5,6,7,8,9,10,11],"assembliesNames":["ABySS\_127","ABySS\_63","CLC","IDBA\_UD","MEGAHIT","MIRA","SOAPdenovo2","SPAdes","SPAdes\_meta","SPAdes\_sc","SPAdes\_sc\_careful","Velvet"]},{"assembliesWithNs":null,"minContig":500,"report":[["Genome statistics",[{"values":["97.698","97.068","99.758","99.758","99.474","98.850","2.111","98.538","99.758","99.758","99.758","99.758","99.758","71.774"],"quality":"More is better","isMain":true,"metricName":"Genome fraction (%)"},{"values":["1.002","1.001","1.002","1.000","1.000","1.008","1.000","1.022","1.000","1.001","1.000","1.000","1.000","1.000"],"quality":"Less is better","isMain":true,"metricName":"Duplication ratio"},{"values":[17852,13705,58223,58223,58174,49102,796,33621,58223,58223,58223,58223,58223,50003],"quality":"More is better","isMain":true,"metricName":"Largest alignment"},{"values":[149200,148106,152304,152004,151571,151763,3216,150792,152004,152156,152004,152004,152004,109364],"quality":"More is better","isMain":true,"metricName":"Total aligned length"},{"values":[6106,7071,44938,44938,44832,44661,null,11445,44938,44938,44938,44938,44938,44661],"quality":"More is better","isMain":false,"metricName":"NG50"},{"values":[3844,4223,17667,33843,33683,26163,null,8540,33843,26502,33843,33843,33843,null],"quality":"More is better","isMain":false,"metricName":"NG75"},{"values":[6846,7646,44938,44938,44832,44661,596,11262,44938,44938,44938,44938,44938,44661],"quality":"More is better","isMain":false,"metricName":"NA50"},{"values":[4074,4724,17667,33843,33683,26163,586,8442,33843,26502,33843,33843,33843,44661],"quality":"More is better","isMain":false,"metricName":"NA75"},{"values":[6106,7071,44938,44938,44832,44661,null,11262,44938,44938,44938,44938,44938,44661],"quality":"More is better","isMain":true,"metricName":"NGA50"},{"values":[3844,4223,17667,33843,33683,26163,null,8442,33843,26502,33843,33843,33843,null],"quality":"More is better","isMain":false,"metricName":"NGA75"},{"values":[8,9,2,2,2,2,null,4,2,2,2,2,2,2],"quality":"Less is better","isMain":false,"metricName":"LG50"},{"values":[16,15,3,3,3,3,null,8,3,3,3,3,3,null],"quality":"Less is better","isMain":false,"metricName":"LG75"},{"values":[7,8,2,2,2,2,3,4,2,2,2,2,2,2],"quality":"Less is better","isMain":false,"metricName":"LA50"},{"values":[15,14,3,3,3,3,4,8,3,3,3,3,3,2],"quality":"Less is better","isMain":false,"metricName":"LA75"},{"values":[8,9,2,2,2,2,null,4,2,2,2,2,2,2],"quality":"Less is better","isMain":true,"metricName":"LGA50"},{"values":[16,15,3,3,3,3,null,8,3,3,3,3,3,null],"quality":"Less is better","isMain":false,"metricName":"LGA75"}]],["Misassemblies",[{"values":[0,0,0,0,0,0,0,0,0,0,0,0,0,0],"quality":"Less is better","isMain":true,"metricName":"# misassemblies"},{"values":[0,0,0,0,0,0,0,0,0,0,0,0,0,0],"quality":"Less is better","isMain":false,"metricName":" # relocations"},{"values":[0,0,0,0,0,0,0,0,0,0,0,0,0,0],"quality":"Less is better","isMain":false,"metricName":" # translocations"},{"values":[0,0,0,0,0,0,0,0,0,0,0,0,0,0],"quality":"Less is better","isMain":false,"metricName":" # inversions"},{"values":[0,0,0,0,0,0,0,0,0,0,0,0,0,0],"quality":"Less is better","isMain":false,"metricName":"# misassembled contigs"},{"values":[0,0,0,0,0,0,0,0,0,0,0,0,0,0],"quality":"Less is better","isMain":true,"metricName":"Misassembled contigs length"},{"values":[0,0,0,0,0,0,0,0,0,0,0,0,0,0],"quality":"Less is better","isMain":false,"metricName":"# local misassemblies"},{"values":[0,0,0,0,0,0,0,0,0,0,0,0,0,0],"quality":"Less is better","isMain":false,"metricName":"# unaligned mis. contigs"}]],["Unaligned",[{"values":[0,0,0,0,0,0,0,0,0,0,0,0,0,0],"quality":"Less is better","isMain":false,"metricName":"# fully unaligned contigs"},{"values":[0,0,0,0,0,0,0,0,0,0,0,0,0,0],"quality":"Less is better","isMain":false,"metricName":"Fully unaligned length"},{"values":[0,0,0,0,0,0,0,0,0,0,0,0,0,0],"quality":"Less is better","isMain":false,"metricName":"# partially unaligned contigs"},{"values":[0,0,0,0,0,0,0,0,0,0,0,0,0,0],"quality":"Less is better","isMain":false,"metricName":"Partially unaligned length"}]],["Mismatches",[{"values":[9,2,3,2,5,0,0,1,3,5,2,2,1,0],"quality":"Less is better","isMain":false,"metricName":"# mismatches"},{"values":[0,0,0,0,0,0,0,31,0,0,0,0,0,0],"quality":"Less is better","isMain":false,"metricName":"# indels"},{"values":[0,0,0,0,0,0,0,1517,0,0,0,0,0,0],"quality":"Less is better","isMain":false,"metricName":"Indels length"},{"values":["6.05","1.35","1.97","1.32","3.30","0.00","0.00","0.67","1.97","3.29","1.32","1.32","0.66","0.00"],"quality":"Less is better","isMain":true,"metricName":"# mismatches per 100 kbp"},{"values":["0.00","0.00","0.00","0.00","0.00","0.00","0.00","20.65","0.00","0.00","0.00","0.00","0.00","0.00"],"quality":"Less is better","isMain":true,"metricName":"# indels per 100 kbp"},{"values":[0,0,0,0,0,0,0,0,0,0,0,0,0,0],"quality":"Less is better","isMain":false,"metricName":" # indels (<= 5 bp)"},{"values":[0,0,0,0,0,0,0,31,0,0,0,0,0,0],"quality":"Less is better","isMain":false,"metricName":" # indels (> 5 bp)"},{"values":[0,0,0,0,0,15,0,3033,0,0,0,0,0,14],"quality":"Less is better","isMain":false,"metricName":"# N's"},{"values":["0.00","0.00","0.00","0.00","0.00","9.88","0.00","1976.55","0.00","0.00","0.00","0.00","0.00","12.80"],"quality":"Less is better","isMain":true,"metricName":"# N's per 100 kbp"}]],["Statistics without reference",[{"values":[31,29,5,4,4,8,5,16,4,5,4,4,4,3],"quality":"Equal","isMain":true,"metricName":"# contigs"},{"values":[28,27,5,4,4,7,0,16,4,5,4,4,4,3],"quality":"Equal","isMain":false,"metricName":"# contigs (>= 1000 bp)"},{"values":[9,13,5,4,4,6,0,10,4,5,4,4,4,3],"quality":"Equal","isMain":false,"metricName":"# contigs (>= 5000 bp)"},{"values":[3,2,5,4,4,4,0,5,4,4,4,4,4,3],"quality":"Equal","isMain":false,"metricName":"# contigs (>= 10000 bp)"},{"values":[0,0,2,3,3,3,0,1,3,3,3,3,3,2],"quality":"Equal","isMain":false,"metricName":"# contigs (>= 25000 bp)"},{"values":[0,0,1,1,1,0,0,0,1,1,1,1,1,1],"quality":"Equal","isMain":false,"metricName":"# contigs (>= 50000 bp)"},{"values":[17852,13705,58223,58223,58174,49102,796,34220,58223,58223,58223,58223,58223,50003],"quality":"More is better","isMain":true,"metricName":"Largest contig"},{"values":[149200,148106,152304,152004,151571,151763,3216,153449,152004,152156,152004,152004,152004,109364],"quality":"More is better","isMain":true,"metricName":"Total length"},{"values":[146949,146465,152304,152004,151571,150770,0,153449,152004,152156,152004,152004,152004,109364],"quality":"More is better","isMain":true,"metricName":"Total length (>= 1000 bp)"},{"values":[86994,106729,152304,152004,151571,149718,0,135326,152004,152156,152004,152004,152004,109364],"quality":"More is better","isMain":false,"metricName":"Total length (>= 5000 bp)"},{"values":[45067,25950,152304,152004,151571,134626,0,94694,152004,144663,152004,152004,152004,109364],"quality":"More is better","isMain":true,"metricName":"Total length (>= 10000 bp)"},{"values":[0,0,103161,137004,136689,119926,0,34220,137004,129663,137004,137004,137004,94664],"quality":"More is better","isMain":false,"metricName":"Total length (>= 25000 bp)"},{"values":[0,0,58223,58223,58174,0,0,0,58223,58223,58223,58223,58223,50003],"quality":"More is better","isMain":true,"metricName":"Total length (>= 50000 bp)"},{"values":[6846,7646,44938,44938,44832,44661,596,11445,44938,44938,44938,44938,44938,44661],"quality":"More is better","isMain":false,"metricName":"N50"},{"values":[4074,4724,17667,33843,33683,26163,586,8540,33843,26502,33843,33843,33843,44661],"quality":"More is better","isMain":false,"metricName":"N75"},{"values":[7,8,2,2,2,2,3,4,2,2,2,2,2,2],"quality":"Less is better","isMain":false,"metricName":"L50"},{"values":[15,14,3,3,3,3,4,8,3,3,3,3,3,2],"quality":"Less is better","isMain":false,"metricName":"L75"},{"values":["32.65","32.65","32.58","32.59","32.60","32.61","32.18","32.57","32.59","32.58","32.59","32.59","32.59","32.52"],"quality":"Equal","isMain":false,"metricName":"GC (%)"}]],["Predicted genes",[]],["Similarity statistics",[{"values":[0,0,3,3,3,2,0,1,3,3,3,3,3,2],"quality":"Equal","isMain":false,"metricName":"# similar correct contigs"},{"values":[0,0,0,0,0,0,0,0,0,0,0,0,0,0],"quality":"Equal","isMain":false,"metricName":"# similar misassembled blocks"}]],["Reference statistics",[{"values":[152372,152372,152372,152372,152372,152372,152372,152372,152372,152372,152372,152372,152372,152372],"quality":"Equal","isMain":false,"metricName":"Reference length"},{"values":[1,1,1,1,1,1,1,1,1,1,1,1,1,1],"quality":"Equal","isMain":false,"metricName":"Reference fragments"},{"values":["32.59","32.59","32.59","32.59","32.59","32.59","32.59","32.59","32.59","32.59","32.59","32.59","32.59","32.59"],"quality":"Equal","isMain":false,"metricName":"Reference GC (%)"}]]],"referenceName":"gi\_118197620\_ref\_NC","date":"18 July 2018, Wednesday, 16:10:43","order":[0,1,2,3,4,5,6,7,8,9,10,11,12,13],"assembliesNames":["ABySS\_127","ABySS\_63","CLC","IDBA\_UD","MEGAHIT","MIRA","Ray\_Meta","SOAPdenovo2","SPAdes","SPAdes\_meta","SPAdes\_sc","SPAdes\_sc\_careful","Velvet","Geneious"]},{"assembliesWithNs":null,"minContig":500,"report":[["Genome statistics",[{"values":["100.000","100.000","100.000","100.000","100.000","8.346","89.853","100.000","100.000","100.000","100.000","100.000"],"quality":"More is better","isMain":true,"metricName":"Genome fraction (%)"},{"values":["1.017","1.000","1.015","1.021","1.020","1.000","1.048","1.019","1.008","1.008","1.008","1.005"],"quality":"Less is better","isMain":true,"metricName":"Duplication ratio"},{"values":[6776,6662,6761,6803,6798,556,5986,6789,6717,6717,6717,6692],"quality":"More is better","isMain":true,"metricName":"Largest alignment"},{"values":[6776,6662,6761,6803,6798,556,5986,6789,6717,6717,6717,6692],"quality":"More is better","isMain":true,"metricName":"Total aligned length"},{"values":[6776,6662,6761,6803,6798,null,6271,6789,6717,6717,6717,6692],"quality":"More is better","isMain":false,"metricName":"NG50"},{"values":[6776,6662,6761,6803,6798,null,6271,6789,6717,6717,6717,6692],"quality":"More is better","isMain":false,"metricName":"NG75"},{"values":[6776,6662,6761,6803,6798,556,5986,6789,6717,6717,6717,6692],"quality":"More is better","isMain":false,"metricName":"NA50"},{"values":[6776,6662,6761,6803,6798,556,5986,6789,6717,6717,6717,6692],"quality":"More is better","isMain":false,"metricName":"NA75"},{"values":[6776,6662,6761,6803,6798,null,5986,6789,6717,6717,6717,6692],"quality":"More is better","isMain":true,"metricName":"NGA50"},{"values":[6776,6662,6761,6803,6798,null,5986,6789,6717,6717,6717,6692],"quality":"More is better","isMain":false,"metricName":"NGA75"},{"values":[1,1,1,1,1,null,1,1,1,1,1,1],"quality":"Less is better","isMain":false,"metricName":"LG50"},{"values":[1,1,1,1,1,null,1,1,1,1,1,1],"quality":"Less is better","isMain":false,"metricName":"LG75"},{"values":[1,1,1,1,1,1,1,1,1,1,1,1],"quality":"Less is better","isMain":false,"metricName":"LA50"},{"values":[1,1,1,1,1,1,1,1,1,1,1,1],"quality":"Less is better","isMain":false,"metricName":"LA75"},{"values":[1,1,1,1,1,null,1,1,1,1,1,1],"quality":"Less is better","isMain":true,"metricName":"LGA50"},{"values":[1,1,1,1,1,null,1,1,1,1,1,1],"quality":"Less is better","isMain":false,"metricName":"LGA75"}]],["Misassemblies",[{"values":[0,0,0,0,0,0,0,0,0,0,0,0],"quality":"Less is better","isMain":true,"metricName":"# misassemblies"},{"values":[0,0,0,0,0,0,0,0,0,0,0,0],"quality":"Less is better","isMain":false,"metricName":" # relocations"},{"values":[0,0,0,0,0,0,0,0,0,0,0,0],"quality":"Less is better","isMain":false,"metricName":" # translocations"},{"values":[0,0,0,0,0,0,0,0,0,0,0,0],"quality":"Less is better","isMain":false,"metricName":" # inversions"},{"values":[0,0,0,0,0,0,0,0,0,0,0,0],"quality":"Less is better","isMain":false,"metricName":"# misassembled contigs"},{"values":[0,0,0,0,0,0,0,0,0,0,0,0],"quality":"Less is better","isMain":true,"metricName":"Misassembled contigs length"},{"values":[1,0,1,1,1,0,0,1,0,0,0,0],"quality":"Less is better","isMain":false,"metricName":"# local misassemblies"},{"values":[0,0,0,0,0,0,0,0,0,0,0,0],"quality":"Less is better","isMain":false,"metricName":"# unaligned mis. contigs"}]],["Unaligned",[{"values":[0,0,0,0,0,0,0,0,0,0,0,0],"quality":"Less is better","isMain":false,"metricName":"# fully unaligned contigs"},{"values":[0,0,0,0,0,0,0,0,0,0,0,0],"quality":"Less is better","isMain":false,"metricName":"Fully unaligned length"},{"values":[0,0,0,0,0,0,0,0,0,0,0,0],"quality":"Less is better","isMain":false,"metricName":"# partially unaligned contigs"},{"values":[0,0,0,0,0,0,0,0,0,0,0,0],"quality":"Less is better","isMain":false,"metricName":"Partially unaligned length"}]],["Mismatches",[{"values":[0,0,0,0,0,0,0,0,0,0,0,0],"quality":"Less is better","isMain":false,"metricName":"# mismatches"},{"values":[0,0,0,0,0,0,2,0,1,1,1,1],"quality":"Less is better","isMain":false,"metricName":"# indels"},{"values":[0,0,0,0,0,0,101,0,55,55,55,30],"quality":"Less is better","isMain":false,"metricName":"Indels length"},{"values":["0.00","0.00","0.00","0.00","0.00","0.00","0.00","0.00","0.00","0.00","0.00","0.00"],"quality":"Less is better","isMain":true,"metricName":"# mismatches per 100 kbp"},{"values":["0.00","0.00","0.00","0.00","0.00","0.00","33.41","0.00","15.01","15.01","15.01","15.01"],"quality":"Less is better","isMain":true,"metricName":"# indels per 100 kbp"},{"values":[0,0,0,0,0,0,0,0,0,0,0,0],"quality":"Less is better","isMain":false,"metricName":" # indels (<= 5 bp)"},{"values":[0,0,0,0,0,0,2,0,1,1,1,1],"quality":"Less is better","isMain":false,"metricName":" # indels (> 5 bp)"},{"values":[0,0,0,0,0,0,285,0,0,0,0,0],"quality":"Less is better","isMain":false,"metricName":"# N's"},{"values":["0.00","0.00","0.00","0.00","0.00","0.00","4544.73","0.00","0.00","0.00","0.00","0.00"],"quality":"Less is better","isMain":true,"metricName":"# N's per 100 kbp"}]],["Statistics without reference",[{"values":[1,1,1,1,1,1,1,1,1,1,1,1],"quality":"Equal","isMain":true,"metricName":"# contigs"},{"values":[1,1,1,1,1,0,1,1,1,1,1,1],"quality":"Equal","isMain":false,"metricName":"# contigs (>= 1000 bp)"},{"values":[1,1,1,1,1,0,1,1,1,1,1,1],"quality":"Equal","isMain":false,"metricName":"# contigs (>= 5000 bp)"},{"values":[0,0,0,0,0,0,0,0,0,0,0,0],"quality":"Equal","isMain":false,"metricName":"# contigs (>= 10000 bp)"},{"values":[0,0,0,0,0,0,0,0,0,0,0,0],"quality":"Equal","isMain":false,"metricName":"# contigs (>= 25000 bp)"},{"values":[0,0,0,0,0,0,0,0,0,0,0,0],"quality":"Equal","isMain":false,"metricName":"# contigs (>= 50000 bp)"},{"values":[6776,6662,6761,6803,6798,556,6271,6789,6717,6717,6717,6692],"quality":"More is better","isMain":true,"metricName":"Largest contig"},{"values":[6776,6662,6761,6803,6798,556,6271,6789,6717,6717,6717,6692],"quality":"More is better","isMain":true,"metricName":"Total length"},{"values":[6776,6662,6761,6803,6798,0,6271,6789,6717,6717,6717,6692],"quality":"More is better","isMain":true,"metricName":"Total length (>= 1000 bp)"},{"values":[6776,6662,6761,6803,6798,0,6271,6789,6717,6717,6717,6692],"quality":"More is better","isMain":false,"metricName":"Total length (>= 5000 bp)"},{"values":[0,0,0,0,0,0,0,0,0,0,0,0],"quality":"More is better","isMain":true,"metricName":"Total length (>= 10000 bp)"},{"values":[0,0,0,0,0,0,0,0,0,0,0,0],"quality":"More is better","isMain":false,"metricName":"Total length (>= 25000 bp)"},{"values":[0,0,0,0,0,0,0,0,0,0,0,0],"quality":"More is better","isMain":true,"metricName":"Total length (>= 50000 bp)"},{"values":[6776,6662,6761,6803,6798,556,6271,6789,6717,6717,6717,6692],"quality":"More is better","isMain":false,"metricName":"N50"},{"values":[6776,6662,6761,6803,6798,556,6271,6789,6717,6717,6717,6692],"quality":"More is better","isMain":false,"metricName":"N75"},{"values":[1,1,1,1,1,1,1,1,1,1,1,1],"quality":"Less is better","isMain":false,"metricName":"L50"},{"values":[1,1,1,1,1,1,1,1,1,1,1,1],"quality":"Less is better","isMain":false,"metricName":"L75"},{"values":["62.60","62.61","62.56","62.52","62.59","62.77","62.68","62.69","62.66","62.66","62.66","62.61"],"quality":"Equal","isMain":false,"metricName":"GC (%)"}]],["Predicted genes",[]],["Similarity statistics",[{"values":[0,0,0,0,0,0,0,0,0,0,0,0],"quality":"Equal","isMain":false,"metricName":"# similar correct contigs"},{"values":[0,0,0,0,0,0,0,0,0,0,0,0],"quality":"Equal","isMain":false,"metricName":"# similar misassembled blocks"}]],["Reference statistics",[{"values":[6662,6662,6662,6662,6662,6662,6662,6662,6662,6662,6662,6662],"quality":"Equal","isMain":false,"metricName":"Reference length"},{"values":[1,1,1,1,1,1,1,1,1,1,1,1],"quality":"Equal","isMain":false,"metricName":"Reference fragments"},{"values":["62.61","62.61","62.61","62.61","62.61","62.61","62.61","62.61","62.61","62.61","62.61","62.61"],"quality":"Equal","isMain":false,"metricName":"Reference GC (%)"}]]],"referenceName":"gi\_118430711\_ref\_NC","date":"18 July 2018, Wednesday, 16:10:52","order":[0,1,2,3,4,5,6,7,8,9,10,11],"assembliesNames":["ABySS\_127","CLC","IDBA\_UD","MEGAHIT","MIRA","Ray\_Meta","SOAPdenovo2","SPAdes","SPAdes\_meta","SPAdes\_sc","SPAdes\_sc\_careful","Velvet"]},{"assembliesWithNs":null,"minContig":500,"report":[["Genome statistics",[{"values":["13.103","10.865","9.335","10.838","10.351","23.180","4.368","12.150","20.098","14.459","10.511","6.845","30.616"],"quality":"More is better","isMain":true,"metricName":"Genome fraction (%)"},{"values":["1.056","1.013","1.005","1.021","1.032","1.000","1.000","1.024","1.019","1.000","1.000","1.010","1.540"],"quality":"Less is better","isMain":true,"metricName":"Duplication ratio"},{"values":[1060,990,1583,1060,1072,3011,1079,1076,2687,2158,1488,958,3851],"quality":"More is better","isMain":true,"metricName":"Largest alignment"},{"values":[5969,4748,4048,4773,4605,9997,1884,5367,8830,6236,4533,2982,20337],"quality":"More is better","isMain":true,"metricName":"Total aligned length"},{"values":[null,null,null,null,null,null,null,null,null,null,null,null,36887],"quality":"More is better","isMain":false,"metricName":"NG50"},{"values":[null,null,null,null,null,null,null,null,null,null,null,null,36887],"quality":"More is better","isMain":false,"metricName":"NG75"},{"values":[764,635,1017,612,764,2812,1079,764,1256,1921,1060,849,null],"quality":"More is better","isMain":false,"metricName":"NA50"},{"values":[612,612,833,537,612,1447,805,612,833,1097,889,604,null],"quality":"More is better","isMain":false,"metricName":"NA75"},{"values":[null,null,null,null,null,null,null,null,null,null,null,null,null],"quality":"More is better","isMain":true,"metricName":"NGA50"},{"values":[null,null,null,null,null,null,null,null,null,null,null,null,1],"quality":"Less is better","isMain":false,"metricName":"LG50"},{"values":[null,null,null,null,null,null,null,null,null,null,null,null,1],"quality":"Less is better","isMain":false,"metricName":"LG75"},{"values":[4,3,2,3,3,2,1,3,3,2,2,2,null],"quality":"Less is better","isMain":false,"metricName":"LA50"},{"values":[6,5,3,5,4,4,2,5,6,3,3,3,null],"quality":"Less is better","isMain":false,"metricName":"LA75"}]],["Misassemblies",[{"values":[0,0,0,0,0,0,0,0,0,0,0,0,0],"quality":"Less is better","isMain":true,"metricName":"# misassemblies"},{"values":[0,0,0,0,0,0,0,0,0,0,0,0,0],"quality":"Less is better","isMain":false,"metricName":" # relocations"},{"values":[0,0,0,0,0,0,0,0,0,0,0,0,0],"quality":"Less is better","isMain":false,"metricName":" # translocations"},{"values":[0,0,0,0,0,0,0,0,0,0,0,0,0],"quality":"Less is better","isMain":false,"metricName":" # inversions"},{"values":[0,0,0,0,0,0,0,0,0,0,0,0,0],"quality":"Less is better","isMain":false,"metricName":"# misassembled contigs"},{"values":[0,0,0,0,0,0,0,0,0,0,0,0,0],"quality":"Less is better","isMain":true,"metricName":"Misassembled contigs length"},{"values":[0,0,0,0,0,1,0,0,0,0,0,0,1],"quality":"Less is better","isMain":false,"metricName":"# local misassemblies"},{"values":[0,0,0,0,0,0,0,0,0,0,0,0,2],"quality":"Less is better","isMain":false,"metricName":"# unaligned mis. contigs"}]],["Unaligned",[{"values":[0,0,0,0,0,0,0,0,0,0,0,0,0],"quality":"Less is better","isMain":false,"metricName":"# fully unaligned contigs"},{"values":[0,0,0,0,0,0,0,0,0,0,0,0,0],"quality":"Less is better","isMain":false,"metricName":"Fully unaligned length"},{"values":[0,0,0,0,0,1,0,0,2,0,0,0,4],"quality":"Less is better","isMain":false,"metricName":"# partially unaligned contigs"},{"values":[0,0,0,0,0,1555,0,0,1455,0,0,0,58581],"quality":"Less is better","isMain":false,"metricName":"Partially unaligned length"}]],["Mismatches",[{"values":[0,4,2,0,0,130,0,0,107,44,4,0,178],"quality":"Less is better","isMain":false,"metricName":"# mismatches"},{"values":[0,0,0,0,0,4,0,0,0,0,0,0,9],"quality":"Less is better","isMain":false,"metricName":"# indels"},{"values":[0,0,0,0,0,4,0,0,0,0,0,0,9],"quality":"Less is better","isMain":false,"metricName":"Indels length"},{"values":["0.00","85.36","49.68","0.00","0.00","1300.39","0.00","0.00","1234.43","705.58","88.24","0.00","1348.08"],"quality":"Less is better","isMain":true,"metricName":"# mismatches per 100 kbp"},{"values":["0.00","0.00","0.00","0.00","0.00","40.01","0.00","0.00","0.00","0.00","0.00","0.00","68.16"],"quality":"Less is better","isMain":true,"metricName":"# indels per 100 kbp"},{"values":[0,0,0,0,0,4,0,0,0,0,0,0,9],"quality":"Less is better","isMain":false,"metricName":" # indels (<= 5 bp)"},{"values":[0,0,0,0,0,0,0,0,0,0,0,0,0],"quality":"Less is better","isMain":false,"metricName":" # indels (> 5 bp)"},{"values":[0,0,0,0,0,2,0,0,0,0,0,0,543],"quality":"Less is better","isMain":false,"metricName":"# N's"},{"values":["0.00","0.00","0.00","0.00","0.00","17.31","0.00","0.00","0.00","0.00","0.00","0.00","688.06"],"quality":"Less is better","isMain":true,"metricName":"# N's per 100 kbp"}]],["Statistics without reference",[{"values":[8,7,4,7,6,5,2,7,7,4,5,4,4],"quality":"Equal","isMain":true,"metricName":"# contigs"},{"values":[1,0,2,1,2,4,1,2,5,4,2,0,4],"quality":"Equal","isMain":false,"metricName":"# contigs (>= 1000 bp)"},{"values":[0,0,0,0,0,0,0,0,0,0,0,0,3],"quality":"Equal","isMain":false,"metricName":"# contigs (>= 5000 bp)"},{"values":[0,0,0,0,0,0,0,0,0,0,0,0,2],"quality":"Equal","isMain":false,"metricName":"# contigs (>= 10000 bp)"},{"values":[0,0,0,0,0,0,0,0,0,0,0,0,2],"quality":"Equal","isMain":false,"metricName":"# contigs (>= 25000 bp)"},{"values":[0,0,0,0,0,0,0,0,0,0,0,0,0],"quality":"Equal","isMain":false,"metricName":"# contigs (>= 50000 bp)"},{"values":[1060,990,1583,1060,1072,4566,1079,1076,2687,2158,1488,958,36887],"quality":"More is better","isMain":true,"metricName":"Largest contig"},{"values":[5969,4748,4048,4773,4605,11552,1884,5367,10285,6236,4533,2982,78918],"quality":"More is better","isMain":true,"metricName":"Total length"},{"values":[1060,0,2600,1060,2132,10560,1079,2136,8882,6236,2548,0,78918],"quality":"More is better","isMain":true,"metricName":"Total length (>= 1000 bp)"},{"values":[0,0,0,0,0,0,0,0,0,0,0,0,74352],"quality":"More is better","isMain":false,"metricName":"Total length (>= 5000 bp)"},{"values":[0,0,0,0,0,0,0,0,0,0,0,0,65450],"quality":"More is better","isMain":true,"metricName":"Total length (>= 10000 bp)"},{"values":[0,0,0,0,0,0,0,0,0,0,0,0,65450],"quality":"More is better","isMain":false,"metricName":"Total length (>= 25000 bp)"},{"values":[0,0,0,0,0,0,0,0,0,0,0,0,0],"quality":"More is better","isMain":true,"metricName":"Total length (>= 50000 bp)"},{"values":[764,635,1017,612,764,2812,1079,764,1926,1921,1060,849,28563],"quality":"More is better","isMain":false,"metricName":"N50"},{"values":[612,612,833,537,612,1735,805,612,1097,1097,889,604,28563],"quality":"More is better","isMain":false,"metricName":"N75"},{"values":[4,3,2,3,3,2,1,3,3,2,2,2,2],"quality":"Less is better","isMain":false,"metricName":"L50"},{"values":[6,5,3,5,4,3,2,5,4,3,3,3,2],"quality":"Less is better","isMain":false,"metricName":"L75"},{"values":["33.42","32.18","32.56","32.96","32.62","33.17","32.75","33.67","33.00","32.63","32.19","32.06","34.90"],"quality":"Equal","isMain":false,"metricName":"GC (%)"}]],["Predicted genes",[]],["Similarity statistics",[{"values":[0,0,0,0,0,0,0,0,0,0,0,0,0],"quality":"Equal","isMain":false,"metricName":"# similar correct contigs"},{"values":[0,0,0,0,0,0,0,0,0,0,0,0,0],"quality":"Equal","isMain":false,"metricName":"# similar misassembled blocks"}]],["Reference statistics",[{"values":[43128,43128,43128,43128,43128,43128,43128,43128,43128,43128,43128,43128,43128],"quality":"Equal","isMain":false,"metricName":"Reference length"},{"values":[1,1,1,1,1,1,1,1,1,1,1,1,1],"quality":"Equal","isMain":false,"metricName":"Reference fragments"},{"values":["34.16","34.16","34.16","34.16","34.16","34.16","34.16","34.16","34.16","34.16","34.16","34.16","34.16"],"quality":"Equal","isMain":false,"metricName":"Reference GC (%)"}]]],"referenceName":"gi\_118430724\_ref\_NC","date":"18 July 2018, Wednesday, 16:11:03","order":[0,1,2,3,4,5,6,7,8,9,10,11,12],"assembliesNames":["ABySS\_127","ABySS\_63","CLC","IDBA\_UD","MEGAHIT","MIRA","Ray\_Meta","SPAdes","SPAdes\_meta","SPAdes\_sc","SPAdes\_sc\_careful","Velvet","Geneious"]},{"assembliesWithNs":null,"minContig":500,"report":[["Genome statistics",[{"values":["67.704","55.269","93.614","93.596","94.572","84.278","84.921","97.020","95.773","95.291","94.835","78.507","34.885"],"quality":"More is better","isMain":true,"metricName":"Genome fraction (%)"},{"values":["1.002","1.002","1.012","1.003","1.002","1.029","1.012","1.002","1.003","1.002","1.002","1.001","0.999"],"quality":"Less is better","isMain":true,"metricName":"Duplication ratio"},{"values":[5757,3008,21238,21238,21219,9286,9978,27427,27049,26741,26742,19469,13833],"quality":"More is better","isMain":true,"metricName":"Largest alignment"},{"values":[217977,177932,303941,301643,304261,278574,274094,312278,308447,306340,304835,252377,111981],"quality":"More is better","isMain":true,"metricName":"Total aligned length"},{"values":[930,596,4632,5135,5542,2247,2573,7417,6355,7311,7301,3821,null],"quality":"More is better","isMain":false,"metricName":"NG50"},{"values":[null,null,2267,2688,3096,1206,1165,3708,3671,3671,3671,991,null],"quality":"More is better","isMain":false,"metricName":"NG75"},{"values":[1402,1098,5024,5652,5677,2352,2914,7417,6355,7301,7301,4913,5512],"quality":"More is better","isMain":false,"metricName":"NA50"},{"values":[910,797,2607,3340,3484,1679,1655,3821,3788,3788,3788,2396,4598],"quality":"More is better","isMain":false,"metricName":"NA75"},{"values":[929,596,4632,5135,5542,2220,2573,7417,6337,6355,6355,3821,null],"quality":"More is better","isMain":true,"metricName":"NGA50"},{"values":[null,null,2267,2688,3096,1206,1052,3708,3627,3542,3540,959,null],"quality":"More is better","isMain":false,"metricName":"NGA75"},{"values":[99,143,20,18,18,48,38,12,14,13,13,25,null],"quality":"Less is better","isMain":false,"metricName":"LG50"},{"values":[null,null,44,38,36,94,83,28,31,29,29,66,null],"quality":"Less is better","isMain":false,"metricName":"LG75"},{"values":[54,57,18,16,16,39,30,12,14,13,13,17,8],"quality":"Less is better","isMain":false,"metricName":"LA50"},{"values":[102,104,39,34,33,73,61,26,29,28,28,34,13],"quality":"Less is better","isMain":false,"metricName":"LA75"},{"values":[99,143,20,18,18,48,38,12,15,14,14,25,null],"quality":"Less is better","isMain":true,"metricName":"LGA50"},{"values":[null,null,44,38,36,95,85,28,32,31,31,67,null],"quality":"Less is better","isMain":false,"metricName":"LGA75"}]],["Misassemblies",[{"values":[1,0,1,1,0,1,0,0,1,3,3,1,0],"quality":"Less is better","isMain":true,"metricName":"# misassemblies"},{"values":[1,0,1,1,0,1,0,0,1,3,3,1,0],"quality":"Less is better","isMain":false,"metricName":" # relocations"},{"values":[0,0,0,0,0,0,0,0,0,0,0,0,0],"quality":"Less is better","isMain":false,"metricName":" # translocations"},{"values":[0,0,0,0,0,0,0,0,0,0,0,0,0],"quality":"Less is better","isMain":false,"metricName":" # inversions"},{"values":[1,0,1,1,0,1,0,0,1,2,2,1,0],"quality":"Less is better","isMain":false,"metricName":"# misassembled contigs"},{"values":[1114,0,1095,1851,0,2579,0,0,10601,30895,30895,2203,0],"quality":"Less is better","isMain":true,"metricName":"Misassembled contigs length"},{"values":[0,0,0,2,0,2,0,3,1,7,7,2,2],"quality":"Less is better","isMain":false,"metricName":"# local misassemblies"},{"values":[0,0,0,0,0,0,0,0,0,0,0,0,0],"quality":"Less is better","isMain":false,"metricName":"# unaligned mis. contigs"}]],["Unaligned",[{"values":[0,0,0,0,0,0,0,0,0,0,0,0,0],"quality":"Less is better","isMain":false,"metricName":"# fully unaligned contigs"},{"values":[0,0,0,0,0,0,0,0,0,0,0,0,0],"quality":"Less is better","isMain":false,"metricName":"Fully unaligned length"},{"values":[0,0,0,0,0,0,1,0,0,0,0,0,1],"quality":"Less is better","isMain":false,"metricName":"# partially unaligned contigs"},{"values":[0,0,0,0,0,0,728,0,0,0,0,0,5532],"quality":"Less is better","isMain":false,"metricName":"Partially unaligned length"}]],["Mismatches",[{"values":[77,29,160,191,90,72,75,225,225,178,145,92,64],"quality":"Less is better","isMain":false,"metricName":"# mismatches"},{"values":[1,0,5,4,1,1,31,4,13,6,6,4,1],"quality":"Less is better","isMain":false,"metricName":"# indels"},{"values":[12,0,66,133,21,84,1538,174,330,146,150,74,84],"quality":"Less is better","isMain":false,"metricName":"Indels length"},{"values":["35.40","16.33","53.20","63.53","29.62","26.59","27.49","72.19","73.13","58.15","47.60","36.48","57.11"],"quality":"Less is better","isMain":true,"metricName":"# mismatches per 100 kbp"},{"values":["0.46","0.00","1.66","1.33","0.33","0.37","11.36","1.28","4.23","1.96","1.97","1.59","0.89"],"quality":"Less is better","isMain":true,"metricName":"# indels per 100 kbp"},{"values":[0,0,3,0,0,0,0,0,5,0,0,2,0],"quality":"Less is better","isMain":false,"metricName":" # indels (<= 5 bp)"},{"values":[1,0,2,4,1,1,31,4,8,6,6,2,1],"quality":"Less is better","isMain":false,"metricName":" # indels (> 5 bp)"},{"values":[0,0,0,0,0,214,3164,0,0,348,316,10,96],"quality":"Less is better","isMain":false,"metricName":"# N's"},{"values":["0.00","0.00","0.00","0.00","0.00","76.82","1143.45","0.00","0.00","113.46","103.54","3.96","81.69"],"quality":"Less is better","isMain":true,"metricName":"# N's per 100 kbp"}]],["Statistics without reference",[{"values":[177,174,105,81,79,140,129,65,68,69,68,82,22],"quality":"Equal","isMain":true,"metricName":"# contigs"},{"values":[94,72,67,68,65,99,89,56,60,56,56,64,22],"quality":"Equal","isMain":false,"metricName":"# contigs (>= 1000 bp)"},{"values":[1,0,18,19,22,5,11,20,21,19,19,16,12],"quality":"Equal","isMain":false,"metricName":"# contigs (>= 5000 bp)"},{"values":[0,0,4,4,5,0,0,9,6,7,7,3,1],"quality":"Equal","isMain":false,"metricName":"# contigs (>= 10000 bp)"},{"values":[0,0,0,0,0,0,0,1,1,1,1,0,0],"quality":"Equal","isMain":false,"metricName":"# contigs (>= 25000 bp)"},{"values":[0,0,0,0,0,0,0,0,0,0,0,0,0],"quality":"Equal","isMain":false,"metricName":"# contigs (>= 50000 bp)"},{"values":[5757,3008,21238,21238,21219,9286,9978,27427,27049,26751,26751,19469,13833],"quality":"More is better","isMain":true,"metricName":"Largest contig"},{"values":[217977,177932,304268,301684,304271,278575,276706,312280,308568,306726,305209,252393,117513],"quality":"More is better","isMain":true,"metricName":"Total length"},{"values":[156489,105377,276747,292935,295132,247074,247960,305885,303101,297481,296684,239023,117513],"quality":"More is better","isMain":true,"metricName":"Total length (>= 1000 bp)"},{"values":[5757,0,154947,168947,187740,33019,73327,210685,201596,200130,200139,123935,87484],"quality":"More is better","isMain":false,"metricName":"Total length (>= 5000 bp)"},{"values":[0,0,61195,62198,75789,0,0,140021,97362,116743,116738,41073,13833],"quality":"More is better","isMain":true,"metricName":"Total length (>= 10000 bp)"},{"values":[0,0,0,0,0,0,0,27427,27049,26751,26751,0,0],"quality":"More is better","isMain":false,"metricName":"Total length (>= 25000 bp)"},{"values":[0,0,0,0,0,0,0,0,0,0,0,0,0],"quality":"More is better","isMain":true,"metricName":"Total length (>= 50000 bp)"},{"values":[1402,1098,5024,5652,5677,2358,2914,7417,7417,7417,7417,4913,6348],"quality":"More is better","isMain":false,"metricName":"N50"},{"values":[914,797,2624,3340,3484,1734,1678,3821,3821,3821,3821,2396,4812],"quality":"More is better","isMain":false,"metricName":"N75"},{"values":[54,57,18,16,16,39,30,12,13,12,12,17,7],"quality":"Less is better","isMain":false,"metricName":"L50"},{"values":[102,104,39,34,33,72,60,26,28,26,26,34,13],"quality":"Less is better","isMain":false,"metricName":"L75"},{"values":["44.68","44.50","44.44","44.49","44.54","44.48","44.27","44.55","44.51","44.45","44.42","44.30","44.47"],"quality":"Equal","isMain":false,"metricName":"GC (%)"}]],["Predicted genes",[]],["Similarity statistics",[{"values":[0,0,2,2,2,0,0,2,2,2,2,0,0],"quality":"Equal","isMain":false,"metricName":"# similar correct contigs"},{"values":[0,0,0,0,0,0,0,0,0,0,0,0,0],"quality":"Equal","isMain":false,"metricName":"# similar misassembled blocks"}]],["Reference statistics",[{"values":[321240,321240,321240,321240,321240,321240,321240,321240,321240,321240,321240,321240,321240],"quality":"Equal","isMain":false,"metricName":"Reference length"},{"values":[1,1,1,1,1,1,1,1,1,1,1,1,1],"quality":"Equal","isMain":false,"metricName":"Reference fragments"},{"values":["44.61","44.61","44.61","44.61","44.61","44.61","44.61","44.61","44.61","44.61","44.61","44.61","44.61"],"quality":"Equal","isMain":false,"metricName":"Reference GC (%)"}]]],"referenceName":"gi\_118496614\_ref\_NC","date":"18 July 2018, Wednesday, 16:11:15","order":[0,1,2,3,4,5,6,7,8,9,10,11,12],"assembliesNames":["ABySS\_127","ABySS\_63","CLC","IDBA\_UD","MEGAHIT","MIRA","SOAPdenovo2","SPAdes","SPAdes\_meta","SPAdes\_sc","SPAdes\_sc\_careful","Velvet","Geneious"]},{"assembliesWithNs":null,"minContig":500,"report":[["Genome statistics",[{"values":["46.540","25.975","56.676","51.592","58.296","80.597","3.116","9.103","67.080","34.647","38.994","42.616","23.767","74.592"],"quality":"More is better","isMain":true,"metricName":"Genome fraction (%)"},{"values":["1.018","1.007","1.034","1.026","1.055","1.393","1.000","1.181","1.034","1.082","1.012","1.018","1.002","1.076"],"quality":"Less is better","isMain":true,"metricName":"Duplication ratio"},{"values":[6995,2031,2827,3938,3501,9299,1373,2055,10579,5071,4638,5195,1769,14321],"quality":"More is better","isMain":true,"metricName":"Largest alignment"},{"values":[20876,11459,25616,23315,26872,48603,1373,4011,30382,16038,17325,19107,10498,34860],"quality":"More is better","isMain":true,"metricName":"Total aligned length"},{"values":[null,null,579,544,1103,6565,null,null,1439,null,null,null,null,28563],"quality":"More is better","isMain":false,"metricName":"NG50"},{"values":[null,null,null,null,null,4478,null,null,null,null,null,null,null,28444],"quality":"More is better","isMain":false,"metricName":"NG75"},{"values":[1467,1819,1284,1425,1467,3274,1373,777,2549,3800,1889,1357,1059,null],"quality":"More is better","isMain":false,"metricName":"NA50"},{"values":[1117,989,900,1012,1215,594,1373,null,1439,1351,1258,859,811,null],"quality":"More is better","isMain":false,"metricName":"NA75"},{"values":[null,null,579,544,1103,5855,null,null,1439,null,null,null,null,5863],"quality":"More is better","isMain":true,"metricName":"NGA50"},{"values":[null,null,null,null,null,2708,null,null,null,null,null,null,null,747],"quality":"More is better","isMain":false,"metricName":"NGA75"},{"values":[null,null,18,15,12,3,null,null,7,null,null,null,null,1],"quality":"Less is better","isMain":false,"metricName":"LG50"},{"values":[null,null,null,null,null,5,null,null,null,null,null,null,null,2],"quality":"Less is better","isMain":false,"metricName":"LG75"},{"values":[4,3,8,5,6,5,1,3,3,2,4,4,4,null],"quality":"Less is better","isMain":false,"metricName":"LA50"},{"values":[7,6,14,9,11,18,1,null,7,5,7,9,7,null],"quality":"Less is better","isMain":false,"metricName":"LA75"},{"values":[null,null,18,15,12,3,null,null,7,null,null,null,null,3],"quality":"Less is better","isMain":true,"metricName":"LGA50"},{"values":[null,null,null,null,null,6,null,null,null,null,null,null,null,7],"quality":"Less is better","isMain":false,"metricName":"LGA75"}]],["Misassemblies",[{"values":[0,0,0,0,0,0,0,0,0,1,0,0,0,0],"quality":"Less is better","isMain":true,"metricName":"# misassemblies"},{"values":[0,0,0,0,0,0,0,0,0,1,0,0,0,0],"quality":"Less is better","isMain":false,"metricName":" # relocations"},{"values":[0,0,0,0,0,0,0,0,0,0,0,0,0,0],"quality":"Less is better","isMain":false,"metricName":" # translocations"},{"values":[0,0,0,0,0,0,0,0,0,0,0,0,0,0],"quality":"Less is better","isMain":false,"metricName":" # inversions"},{"values":[0,0,0,0,0,0,0,0,0,1,0,0,0,0],"quality":"Less is better","isMain":false,"metricName":"# misassembled contigs"},{"values":[0,0,0,0,0,0,0,0,0,6917,0,0,0,0],"quality":"Less is better","isMain":true,"metricName":"Misassembled contigs length"},{"values":[0,0,0,0,0,4,0,1,0,0,0,0,0,2],"quality":"Less is better","isMain":false,"metricName":"# local misassemblies"},{"values":[0,0,0,0,0,1,0,0,0,0,0,0,0,2],"quality":"Less is better","isMain":false,"metricName":"# unaligned mis. contigs"}]],["Unaligned",[{"values":[0,0,0,0,0,0,0,0,0,0,0,0,0,0],"quality":"Less is better","isMain":false,"metricName":"# fully unaligned contigs"},{"values":[0,0,0,0,0,0,0,0,0,0,0,0,0,0],"quality":"Less is better","isMain":false,"metricName":"Fully unaligned length"},{"values":[0,0,0,0,0,7,0,2,0,0,1,1,0,5],"quality":"Less is better","isMain":false,"metricName":"# partially unaligned contigs"},{"values":[0,0,0,0,0,12948,0,2771,0,0,1690,1690,0,49785],"quality":"Less is better","isMain":false,"metricName":"Partially unaligned length"}]],["Mismatches",[{"values":[0,0,17,19,33,165,0,0,14,173,59,61,3,341],"quality":"Less is better","isMain":false,"metricName":"# mismatches"},{"values":[0,0,1,0,0,0,0,8,0,8,2,1,0,6],"quality":"Less is better","isMain":false,"metricName":"# indels"},{"values":[0,0,1,0,0,0,0,386,0,8,4,3,0,11],"quality":"Less is better","isMain":false,"metricName":"Indels length"},{"values":["0.00","0.00","68.08","83.58","128.47","464.63","0.00","0.00","47.37","1133.24","343.40","324.87","28.65","1037.55"],"quality":"Less is better","isMain":true,"metricName":"# mismatches per 100 kbp"},{"values":["0.00","0.00","4.00","0.00","0.00","0.00","0.00","199.45","0.00","52.40","11.64","5.33","0.00","18.26"],"quality":"Less is better","isMain":true,"metricName":"# indels per 100 kbp"},{"values":[0,0,1,0,0,0,0,0,0,8,2,1,0,5],"quality":"Less is better","isMain":false,"metricName":" # indels (<= 5 bp)"},{"values":[0,0,0,0,0,0,0,8,0,0,0,0,0,1],"quality":"Less is better","isMain":false,"metricName":" # indels (> 5 bp)"},{"values":[0,0,0,0,0,20,0,2164,0,0,0,0,0,925],"quality":"Less is better","isMain":false,"metricName":"# N's"},{"values":["0.00","0.00","0.00","0.00","0.00","32.03","0.00","28826.43","0.00","0.00","0.00","0.00","0.00","1086.41"],"quality":"Less is better","isMain":true,"metricName":"# N's per 100 kbp"}]],["Statistics without reference",[{"values":[14,9,24,17,19,21,1,4,16,7,11,14,11,7],"quality":"Equal","isMain":true,"metricName":"# contigs"},{"values":[7,5,12,9,12,15,1,4,9,4,7,7,4,7],"quality":"Equal","isMain":false,"metricName":"# contigs (>= 1000 bp)"},{"values":[1,0,0,0,0,4,0,0,1,1,0,1,0,4],"quality":"Equal","isMain":false,"metricName":"# contigs (>= 5000 bp)"},{"values":[0,0,0,0,0,0,0,0,1,0,0,0,0,3],"quality":"Equal","isMain":false,"metricName":"# contigs (>= 10000 bp)"},{"values":[0,0,0,0,0,0,0,0,0,0,0,0,0,2],"quality":"Equal","isMain":false,"metricName":"# contigs (>= 25000 bp)"},{"values":[0,0,0,0,0,0,0,0,0,0,0,0,0,0],"quality":"Equal","isMain":false,"metricName":"# contigs (>= 50000 bp)"},{"values":[6995,2031,2939,3938,3501,9755,1373,3304,10744,6917,4639,5195,1769,28563],"quality":"More is better","isMain":true,"metricName":"Largest contig"},{"values":[20876,11520,25817,23315,27086,62433,1373,7507,30547,16512,19070,20797,10498,85143],"quality":"More is better","isMain":true,"metricName":"Total length"},{"values":[15731,8556,17714,17939,22740,58260,1373,7507,25850,13971,16335,16148,5637,85143],"quality":"More is better","isMain":true,"metricName":"Total length (>= 1000 bp)"},{"values":[6995,0,0,0,0,31835,0,0,10744,6917,0,5195,0,77901],"quality":"More is better","isMain":false,"metricName":"Total length (>= 5000 bp)"},{"values":[0,0,0,0,0,0,0,0,10744,0,0,0,0,71328],"quality":"More is better","isMain":true,"metricName":"Total length (>= 10000 bp)"},{"values":[0,0,0,0,0,0,0,0,0,0,0,0,0,57007],"quality":"More is better","isMain":false,"metricName":"Total length (>= 25000 bp)"},{"values":[0,0,0,0,0,0,0,0,0,0,0,0,0,0],"quality":"More is better","isMain":true,"metricName":"Total length (>= 50000 bp)"},{"values":[1467,1880,1284,1425,1508,5832,1373,1698,2549,3800,2032,2032,1059,28444],"quality":"More is better","isMain":false,"metricName":"N50"},{"values":[1117,989,900,1012,1219,2708,1373,1336,1439,1898,1300,1218,811,14321],"quality":"More is better","isMain":false,"metricName":"N75"},{"values":[4,3,8,5,5,4,1,2,3,2,3,3,4,2],"quality":"Less is better","isMain":false,"metricName":"L50"},{"values":[7,6,14,9,10,9,1,3,7,3,6,7,7,3],"quality":"Less is better","isMain":false,"metricName":"L75"},{"values":["32.22","29.74","32.65","32.12","32.37","33.44","26.80","29.12","32.56","32.34","32.20","32.42","29.80","34.64"],"quality":"Equal","isMain":false,"metricName":"GC (%)"}]],["Predicted genes",[]],["Similarity statistics",[{"values":[0,0,0,0,0,0,0,0,0,0,0,0,0,0],"quality":"Equal","isMain":false,"metricName":"# similar correct contigs"},{"values":[0,0,0,0,0,0,0,0,0,0,0,0,0,0],"quality":"Equal","isMain":false,"metricName":"# similar misassembled blocks"}]],["Reference statistics",[{"values":[44061,44061,44061,44061,44061,44061,44061,44061,44061,44061,44061,44061,44061,44061],"quality":"Equal","isMain":false,"metricName":"Reference length"},{"values":[1,1,1,1,1,1,1,1,1,1,1,1,1,1],"quality":"Equal","isMain":false,"metricName":"Reference fragments"},{"values":["32.99","32.99","32.99","32.99","32.99","32.99","32.99","32.99","32.99","32.99","32.99","32.99","32.99","32.99"],"quality":"Equal","isMain":false,"metricName":"Reference GC (%)"}]]],"referenceName":"gi\_118725053\_ref\_NC","date":"18 July 2018, Wednesday, 16:11:26","order":[0,1,2,3,4,5,6,7,8,9,10,11,12,13],"assembliesNames":["ABySS\_127","ABySS\_63","CLC","IDBA\_UD","MEGAHIT","MIRA","Ray\_Meta","SOAPdenovo2","SPAdes","SPAdes\_meta","SPAdes\_sc","SPAdes\_sc\_careful","Velvet","Geneious"]},{"assembliesWithNs":null,"minContig":500,"report":[["Genome statistics",[{"values":["19.326","4.385","27.956","16.631","29.804","57.871","1.155","39.795","7.796","24.219","28.299","28.705","16.709"],"quality":"More is better","isMain":true,"metricName":"Genome fraction (%)"},{"values":["1.000","1.000","1.005","1.000","1.042","1.294","1.201","1.000","1.000","1.000","1.000","1.002","1.191"],"quality":"Less is better","isMain":true,"metricName":"Duplication ratio"},{"values":[3020,783,2484,3547,3304,3689,518,7111,2751,5052,7111,6282,3698],"quality":"More is better","isMain":true,"metricName":"Largest alignment"},{"values":[8669,1967,12607,7460,13933,33176,518,17852,3498,10863,12697,12905,8927],"quality":"More is better","isMain":true,"metricName":"Total aligned length"},{"values":[null,null,null,null,null,9674,null,null,null,null,null,null,42152],"quality":"More is better","isMain":false,"metricName":"NG50"},{"values":[null,null,null,null,null,8776,null,null,null,null,null,null,42152],"quality":"More is better","isMain":false,"metricName":"NG75"},{"values":[734,677,1344,807,885,565,518,2504,2751,1104,7111,2746,null],"quality":"More is better","isMain":false,"metricName":"NA50"},{"values":[592,507,602,680,577,null,518,1295,747,675,693,962,null],"quality":"More is better","isMain":false,"metricName":"NA75"},{"values":[null,null,null,null,null,995,null,null,null,null,null,null,null],"quality":"More is better","isMain":true,"metricName":"NGA50"},{"values":[null,null,null,null,null,2,null,null,null,null,null,null,1],"quality":"Less is better","isMain":false,"metricName":"LG50"},{"values":[null,null,null,null,null,3,null,null,null,null,null,null,1],"quality":"Less is better","isMain":false,"metricName":"LG75"},{"values":[3,2,4,2,4,23,1,2,1,2,1,2,null],"quality":"Less is better","isMain":false,"metricName":"LA50"},{"values":[6,3,8,4,9,null,1,5,2,5,5,3,null],"quality":"Less is better","isMain":false,"metricName":"LA75"},{"values":[null,null,null,null,null,12,null,null,null,null,null,null,null],"quality":"Less is better","isMain":true,"metricName":"LGA50"}]],["Misassemblies",[{"values":[0,0,0,0,0,0,0,0,0,0,0,0,0],"quality":"Less is better","isMain":true,"metricName":"# misassemblies"},{"values":[0,0,0,0,0,0,0,0,0,0,0,0,0],"quality":"Less is better","isMain":false,"metricName":" # relocations"},{"values":[0,0,0,0,0,0,0,0,0,0,0,0,0],"quality":"Less is better","isMain":false,"metricName":" # translocations"},{"values":[0,0,0,0,0,0,0,0,0,0,0,0,0],"quality":"Less is better","isMain":false,"metricName":" # inversions"},{"values":[0,0,0,0,0,0,0,0,0,0,0,0,0],"quality":"Less is better","isMain":false,"metricName":"# misassembled contigs"},{"values":[0,0,0,0,0,0,0,0,0,0,0,0,0],"quality":"Less is better","isMain":true,"metricName":"Misassembled contigs length"},{"values":[0,0,0,0,0,0,0,0,1,0,0,0,0],"quality":"Less is better","isMain":false,"metricName":"# local misassemblies"},{"values":[0,0,0,0,0,2,0,0,0,0,0,0,2],"quality":"Less is better","isMain":false,"metricName":"# unaligned mis. contigs"}]],["Unaligned",[{"values":[0,0,0,0,0,0,0,0,0,0,0,0,0],"quality":"Less is better","isMain":false,"metricName":"# fully unaligned contigs"},{"values":[0,0,0,0,0,0,0,0,0,0,0,0,0],"quality":"Less is better","isMain":false,"metricName":"Fully unaligned length"},{"values":[0,0,0,0,0,4,0,0,1,0,0,0,2],"quality":"Less is better","isMain":false,"metricName":"# partially unaligned contigs"},{"values":[0,0,0,0,0,26859,0,0,555,0,0,0,69953],"quality":"Less is better","isMain":false,"metricName":"Partially unaligned length"}]],["Mismatches",[{"values":[0,0,16,0,3,144,0,3,10,11,7,4,30],"quality":"Less is better","isMain":false,"metricName":"# mismatches"},{"values":[0,0,1,0,0,5,1,1,1,1,1,0,0],"quality":"Less is better","isMain":false,"metricName":"# indels"},{"values":[0,0,1,0,0,5,48,1,1,1,1,0,0],"quality":"Less is better","isMain":false,"metricName":"Indels length"},{"values":["0.00","0.00","127.59","0.00","22.44","554.72","0.00","16.81","285.96","101.25","55.14","31.07","400.27"],"quality":"Less is better","isMain":true,"metricName":"# mismatches per 100 kbp"},{"values":["0.00","0.00","7.97","0.00","0.00","19.26","193.05","5.60","28.60","9.20","7.88","0.00","0.00"],"quality":"Less is better","isMain":true,"metricName":"# indels per 100 kbp"},{"values":[0,0,1,0,0,5,0,1,1,1,1,0,0],"quality":"Less is better","isMain":false,"metricName":" # indels (<= 5 bp)"},{"values":[0,0,0,0,0,0,1,0,0,0,0,0,0],"quality":"Less is better","isMain":false,"metricName":" # indels (> 5 bp)"},{"values":[0,0,0,0,0,9,104,0,0,0,0,0,468],"quality":"Less is better","isMain":false,"metricName":"# N's"},{"values":["0.00","0.00","0.00","0.00","0.00","14.89","16720.26","0.00","0.00","0.00","0.00","0.00","593.31"],"quality":"Less is better","isMain":true,"metricName":"# N's per 100 kbp"}]],["Statistics without reference",[{"values":[10,3,13,7,15,26,1,9,2,9,9,8,3],"quality":"Equal","isMain":true,"metricName":"# contigs"},{"values":[1,0,5,1,3,11,0,7,1,2,1,2,3],"quality":"Equal","isMain":false,"metricName":"# contigs (>= 1000 bp)"},{"values":[0,0,0,0,0,3,0,1,0,1,1,1,2],"quality":"Equal","isMain":false,"metricName":"# contigs (>= 5000 bp)"},{"values":[0,0,0,0,0,1,0,0,0,0,0,0,2],"quality":"Equal","isMain":false,"metricName":"# contigs (>= 10000 bp)"},{"values":[0,0,0,0,0,0,0,0,0,0,0,0,2],"quality":"Equal","isMain":false,"metricName":"# contigs (>= 25000 bp)"},{"values":[0,0,0,0,0,0,0,0,0,0,0,0,0],"quality":"Equal","isMain":false,"metricName":"# contigs (>= 50000 bp)"},{"values":[3020,783,2484,3547,3304,16098,622,7111,3306,5052,7111,6282,42152],"quality":"More is better","isMain":true,"metricName":"Largest contig"},{"values":[8669,1967,12607,7460,13933,60449,622,17852,4053,10863,12697,12905,78880],"quality":"More is better","isMain":true,"metricName":"Total length"},{"values":[3020,0,7828,3547,6327,50131,0,16410,3306,6156,7111,9028,78880],"quality":"More is better","isMain":true,"metricName":"Total length (>= 1000 bp)"},{"values":[0,0,0,0,0,34548,0,7111,0,5052,7111,6282,75182],"quality":"More is better","isMain":false,"metricName":"Total length (>= 5000 bp)"},{"values":[0,0,0,0,0,16098,0,0,0,0,0,0,75182],"quality":"More is better","isMain":true,"metricName":"Total length (>= 10000 bp)"},{"values":[0,0,0,0,0,0,0,0,0,0,0,0,75182],"quality":"More is better","isMain":false,"metricName":"Total length (>= 25000 bp)"},{"values":[0,0,0,0,0,0,0,0,0,0,0,0,0],"quality":"More is better","isMain":true,"metricName":"Total length (>= 50000 bp)"},{"values":[734,677,1344,807,885,8776,622,2504,3306,1104,7111,2746,42152],"quality":"More is better","isMain":false,"metricName":"N50"},{"values":[592,507,602,680,577,1596,622,1295,3306,675,693,962,33030],"quality":"More is better","isMain":false,"metricName":"N75"},{"values":[3,2,4,2,4,3,1,2,1,2,1,2,1],"quality":"Less is better","isMain":false,"metricName":"L50"},{"values":[6,3,8,4,9,8,1,5,1,5,5,3,2],"quality":"Less is better","isMain":false,"metricName":"L75"},{"values":["33.78","31.77","33.10","33.91","34.06","33.57","31.85","33.89","32.35","33.97","33.24","33.37","34.51"],"quality":"Equal","isMain":false,"metricName":"GC (%)"}]],["Predicted genes",[]],["Similarity statistics",[{"values":[0,0,0,0,0,0,0,0,0,0,0,0,0],"quality":"Equal","isMain":false,"metricName":"# similar correct contigs"},{"values":[0,0,0,0,0,0,0,0,0,0,0,0,0],"quality":"Equal","isMain":false,"metricName":"# similar misassembled blocks"}]],["Reference statistics",[{"values":[44857,44857,44857,44857,44857,44857,44857,44857,44857,44857,44857,44857,44857],"quality":"Equal","isMain":false,"metricName":"Reference length"},{"values":[1,1,1,1,1,1,1,1,1,1,1,1,1],"quality":"Equal","isMain":false,"metricName":"Reference fragments"},{"values":["33.47","33.47","33.47","33.47","33.47","33.47","33.47","33.47","33.47","33.47","33.47","33.47","33.47"],"quality":"Equal","isMain":false,"metricName":"Reference GC (%)"}]]],"referenceName":"gi\_119443652\_ref\_NC","date":"18 July 2018, Wednesday, 16:11:37","order":[0,1,2,3,4,5,6,7,8,9,10,11,12],"assembliesNames":["ABySS\_127","ABySS\_63","CLC","IDBA\_UD","MEGAHIT","MIRA","SOAPdenovo2","SPAdes","SPAdes\_meta","SPAdes\_sc","SPAdes\_sc\_careful","Velvet","Geneious"]},{"assembliesWithNs":null,"minContig":500,"report":[["Genome statistics",[{"values":["100.000","100.000","100.000","99.766","83.011","62.060","100.000","100.000","100.000","100.000","100.000"],"quality":"More is better","isMain":true,"metricName":"Genome fraction (%)"},{"values":["1.000","1.001","1.002","1.005","1.019","1.020","1.002","1.001","1.001","1.001","1.000"],"quality":"Less is better","isMain":true,"metricName":"Duplication ratio"},{"values":[70153,70252,70294,69989,3577,21931,70280,70208,70208,70208,70183],"quality":"More is better","isMain":true,"metricName":"Largest alignment"},{"values":[70153,70252,70294,69989,59353,44093,70280,70208,70208,70208,70183],"quality":"More is better","isMain":true,"metricName":"Total aligned length"},{"values":[70153,70252,70294,70325,1387,29197,70280,70208,70208,70208,70183],"quality":"More is better","isMain":false,"metricName":"NG50"},{"values":[70153,70252,70294,70325,785,29197,70280,70208,70208,70208,70183],"quality":"More is better","isMain":false,"metricName":"NG75"},{"values":[70153,70252,70294,69989,1729,1808,70280,70208,70208,70208,70183],"quality":"More is better","isMain":false,"metricName":"NA50"},{"values":[70153,70252,70294,69989,903,null,70280,70208,70208,70208,70183],"quality":"More is better","isMain":false,"metricName":"NA75"},{"values":[70153,70252,70294,69989,1387,1808,70280,70208,70208,70208,70183],"quality":"More is better","isMain":true,"metricName":"NGA50"},{"values":[70153,70252,70294,69989,785,null,70280,70208,70208,70208,70183],"quality":"More is better","isMain":false,"metricName":"NGA75"},{"values":[1,1,1,1,16,2,1,1,1,1,1],"quality":"Less is better","isMain":false,"metricName":"LG50"},{"values":[1,1,1,1,34,2,1,1,1,1,1],"quality":"Less is better","isMain":false,"metricName":"LG75"},{"values":[1,1,1,1,12,5,1,1,1,1,1],"quality":"Less is better","isMain":false,"metricName":"LA50"},{"values":[1,1,1,1,24,null,1,1,1,1,1],"quality":"Less is better","isMain":false,"metricName":"LA75"},{"values":[1,1,1,1,16,5,1,1,1,1,1],"quality":"Less is better","isMain":true,"metricName":"LGA50"},{"values":[1,1,1,1,34,null,1,1,1,1,1],"quality":"Less is better","isMain":false,"metricName":"LGA75"}]],["Misassemblies",[{"values":[0,0,0,0,0,0,0,0,0,0,0],"quality":"Less is better","isMain":true,"metricName":"# misassemblies"},{"values":[0,0,0,0,0,0,0,0,0,0,0],"quality":"Less is better","isMain":false,"metricName":" # relocations"},{"values":[0,0,0,0,0,0,0,0,0,0,0],"quality":"Less is better","isMain":false,"metricName":" # translocations"},{"values":[0,0,0,0,0,0,0,0,0,0,0],"quality":"Less is better","isMain":false,"metricName":" # inversions"},{"values":[0,0,0,0,0,0,0,0,0,0,0],"quality":"Less is better","isMain":false,"metricName":"# misassembled contigs"},{"values":[0,0,0,0,0,0,0,0,0,0,0],"quality":"Less is better","isMain":true,"metricName":"Misassembled contigs length"},{"values":[0,1,1,0,0,2,1,0,0,0,0],"quality":"Less is better","isMain":false,"metricName":"# local misassemblies"},{"values":[0,0,0,0,0,1,0,0,0,0,0],"quality":"Less is better","isMain":false,"metricName":"# unaligned mis. contigs"}]],["Unaligned",[{"values":[0,0,0,0,0,0,0,0,0,0,0],"quality":"Less is better","isMain":false,"metricName":"# fully unaligned contigs"},{"values":[0,0,0,0,0,0,0,0,0,0,0],"quality":"Less is better","isMain":false,"metricName":"Fully unaligned length"},{"values":[0,0,0,0,0,2,0,0,0,0,0],"quality":"Less is better","isMain":false,"metricName":"# partially unaligned contigs"},{"values":[0,0,0,0,0,25703,0,0,0,0,0],"quality":"Less is better","isMain":false,"metricName":"Partially unaligned length"}]],["Mismatches",[{"values":[0,0,0,0,0,0,0,0,0,0,0],"quality":"Less is better","isMain":false,"metricName":"# mismatches"},{"values":[0,0,0,0,0,24,0,1,1,1,1],"quality":"Less is better","isMain":false,"metricName":"# indels"},{"values":[0,0,0,0,0,1122,0,55,55,55,30],"quality":"Less is better","isMain":false,"metricName":"Indels length"},{"values":["0.00","0.00","0.00","0.00","0.00","0.00","0.00","0.00","0.00","0.00","0.00"],"quality":"Less is better","isMain":true,"metricName":"# mismatches per 100 kbp"},{"values":["0.00","0.00","0.00","0.00","0.00","55.13","0.00","1.43","1.43","1.43","1.43"],"quality":"Less is better","isMain":true,"metricName":"# indels per 100 kbp"},{"values":[0,0,0,0,0,0,0,0,0,0,0],"quality":"Less is better","isMain":false,"metricName":" # indels (<= 5 bp)"},{"values":[0,0,0,0,0,24,0,1,1,1,1],"quality":"Less is better","isMain":false,"metricName":" # indels (> 5 bp)"},{"values":[0,0,0,0,0,5409,0,0,0,0,0],"quality":"Less is better","isMain":false,"metricName":"# N's"},{"values":["0.00","0.00","0.00","0.00","0.00","7716.23","0.00","0.00","0.00","0.00","0.00"],"quality":"Less is better","isMain":true,"metricName":"# N's per 100 kbp"}]],["Statistics without reference",[{"values":[1,1,1,1,44,8,1,1,1,1,1],"quality":"Equal","isMain":true,"metricName":"# contigs"},{"values":[1,1,1,1,23,4,1,1,1,1,1],"quality":"Equal","isMain":false,"metricName":"# contigs (>= 1000 bp)"},{"values":[1,1,1,1,0,3,1,1,1,1,1],"quality":"Equal","isMain":false,"metricName":"# contigs (>= 5000 bp)"},{"values":[1,1,1,1,0,2,1,1,1,1,1],"quality":"Equal","isMain":false,"metricName":"# contigs (>= 10000 bp)"},{"values":[1,1,1,1,0,2,1,1,1,1,1],"quality":"Equal","isMain":false,"metricName":"# contigs (>= 25000 bp)"},{"values":[1,1,1,1,0,0,1,1,1,1,1],"quality":"Equal","isMain":false,"metricName":"# contigs (>= 50000 bp)"},{"values":[70153,70252,70294,70325,3577,29941,70280,70208,70208,70208,70183],"quality":"More is better","isMain":true,"metricName":"Largest contig"},{"values":[70153,70252,70294,70325,59353,70099,70280,70208,70208,70208,70183],"quality":"More is better","isMain":true,"metricName":"Total length"},{"values":[70153,70252,70294,70325,43875,67413,70280,70208,70208,70208,70183],"quality":"More is better","isMain":true,"metricName":"Total length (>= 1000 bp)"},{"values":[70153,70252,70294,70325,0,65512,70280,70208,70208,70208,70183],"quality":"More is better","isMain":false,"metricName":"Total length (>= 5000 bp)"},{"values":[70153,70252,70294,70325,0,59138,70280,70208,70208,70208,70183],"quality":"More is better","isMain":true,"metricName":"Total length (>= 10000 bp)"},{"values":[70153,70252,70294,70325,0,59138,70280,70208,70208,70208,70183],"quality":"More is better","isMain":false,"metricName":"Total length (>= 25000 bp)"},{"values":[70153,70252,70294,70325,0,0,70280,70208,70208,70208,70183],"quality":"More is better","isMain":true,"metricName":"Total length (>= 50000 bp)"},{"values":[70153,70252,70294,70325,1729,29197,70280,70208,70208,70208,70183],"quality":"More is better","isMain":false,"metricName":"N50"},{"values":[70153,70252,70294,70325,903,29197,70280,70208,70208,70208,70183],"quality":"More is better","isMain":false,"metricName":"N75"},{"values":[1,1,1,1,12,2,1,1,1,1,1],"quality":"Less is better","isMain":false,"metricName":"L50"},{"values":[1,1,1,1,24,2,1,1,1,1,1],"quality":"Less is better","isMain":false,"metricName":"L75"},{"values":["41.30","41.31","41.31","41.29","41.31","41.22","41.30","41.30","41.30","41.30","41.30"],"quality":"Equal","isMain":false,"metricName":"GC (%)"}]],["Predicted genes",[]],["Similarity statistics",[{"values":[0,0,0,0,0,0,0,0,0,0,0],"quality":"Equal","isMain":false,"metricName":"# similar correct contigs"},{"values":[0,0,0,0,0,0,0,0,0,0,0],"quality":"Equal","isMain":false,"metricName":"# similar misassembled blocks"}]],["Reference statistics",[{"values":[70153,70153,70153,70153,70153,70153,70153,70153,70153,70153,70153],"quality":"Equal","isMain":false,"metricName":"Reference length"},{"values":[1,1,1,1,1,1,1,1,1,1,1],"quality":"Equal","isMain":false,"metricName":"Reference fragments"},{"values":["41.30","41.30","41.30","41.30","41.30","41.30","41.30","41.30","41.30","41.30","41.30"],"quality":"Equal","isMain":false,"metricName":"Reference GC (%)"}]]],"referenceName":"gi\_119952179\_ref\_NC","date":"18 July 2018, Wednesday, 16:11:47","order":[0,1,2,3,4,5,6,7,8,9,10],"assembliesNames":["CLC","IDBA\_UD","MEGAHIT","MIRA","Ray\_Meta","SOAPdenovo2","SPAdes","SPAdes\_meta","SPAdes\_sc","SPAdes\_sc\_careful","Velvet"]},{"assembliesWithNs":null,"minContig":500,"report":[["Genome statistics",[{"values":["69.500","49.452","86.142","69.493","81.962","100.000","1.398","26.285","91.578","24.348","53.086","55.046","32.213","99.988"],"quality":"More is better","isMain":true,"metricName":"Genome fraction (%)"},{"values":["1.010","1.005","1.000","1.010","1.018","1.372","1.000","1.273","1.000","1.000","1.006","1.008","1.003","1.507"],"quality":"Less is better","isMain":true,"metricName":"Duplication ratio"},{"values":[13127,3051,16636,5102,7697,40776,607,2499,31176,7763,3407,3463,2182,50789],"quality":"More is better","isMain":true,"metricName":"Largest alignment"},{"values":[30473,21586,37403,30473,36231,59576,607,11488,39763,10570,23184,24090,14022,65421],"quality":"More is better","isMain":true,"metricName":"Total aligned length"},{"values":[2366,null,13566,1515,5208,44069,null,null,31176,null,607,675,null,56062],"quality":"More is better","isMain":false,"metricName":"NG50"},{"values":[null,null,1927,null,1088,44069,null,null,2430,null,null,null,null,56062],"quality":"More is better","isMain":false,"metricName":"NG75"},{"values":[5165,2006,13566,2579,5198,5524,607,211,31176,7763,1842,1842,1512,50789],"quality":"More is better","isMain":false,"metricName":"NA50"},{"values":[2366,1392,13566,1015,1745,null,607,null,31176,2807,1434,935,921,null],"quality":"More is better","isMain":false,"metricName":"NA75"},{"values":[2366,null,13566,1515,5198,40776,null,null,31176,null,607,675,null,50789],"quality":"More is better","isMain":true,"metricName":"NGA50"},{"values":[null,null,1927,null,864,40776,null,null,2430,null,null,null,null,50789],"quality":"More is better","isMain":false,"metricName":"NGA75"},{"values":[4,null,2,8,4,1,null,null,1,null,14,13,null,1],"quality":"Less is better","isMain":false,"metricName":"LG50"},{"values":[null,null,4,null,8,1,null,null,2,null,null,null,null,1],"quality":"Less is better","isMain":false,"metricName":"LG75"},{"values":[2,5,2,4,4,2,1,9,1,1,5,5,4,1],"quality":"Less is better","isMain":false,"metricName":"LA50"},{"values":[4,8,2,9,7,null,1,null,1,2,8,9,7,null],"quality":"Less is better","isMain":false,"metricName":"LA75"},{"values":[4,null,2,8,4,1,null,null,1,null,14,13,null,1],"quality":"Less is better","isMain":true,"metricName":"LGA50"},{"values":[null,null,4,null,10,1,null,null,2,null,null,null,null,1],"quality":"Less is better","isMain":false,"metricName":"LGA75"}]],["Misassemblies",[{"values":[0,0,0,0,0,0,0,0,0,0,0,0,0,0],"quality":"Less is better","isMain":true,"metricName":"# misassemblies"},{"values":[0,0,0,0,0,0,0,0,0,0,0,0,0,0],"quality":"Less is better","isMain":false,"metricName":" # relocations"},{"values":[0,0,0,0,0,0,0,0,0,0,0,0,0,0],"quality":"Less is better","isMain":false,"metricName":" # translocations"},{"values":[0,0,0,0,0,0,0,0,0,0,0,0,0,0],"quality":"Less is better","isMain":false,"metricName":" # inversions"},{"values":[0,0,0,0,0,0,0,0,0,0,0,0,0,0],"quality":"Less is better","isMain":false,"metricName":"# misassembled contigs"},{"values":[0,0,0,0,0,0,0,0,0,0,0,0,0,0],"quality":"Less is better","isMain":true,"metricName":"Misassembled contigs length"},{"values":[0,0,0,0,0,2,0,0,0,0,0,0,0,2],"quality":"Less is better","isMain":false,"metricName":"# local misassemblies"},{"values":[0,0,0,0,0,1,0,3,0,0,0,0,0,1],"quality":"Less is better","isMain":false,"metricName":"# unaligned mis. contigs"}]],["Unaligned",[{"values":[0,0,0,0,0,0,0,0,0,0,0,0,0,0],"quality":"Less is better","isMain":false,"metricName":"# fully unaligned contigs"},{"values":[0,0,0,0,0,0,0,0,0,0,0,0,0,0],"quality":"Less is better","isMain":false,"metricName":"Fully unaligned length"},{"values":[0,0,0,0,1,2,0,4,0,0,0,0,0,2],"quality":"Less is better","isMain":false,"metricName":"# partially unaligned contigs"},{"values":[0,0,0,0,2509,23641,0,6325,0,0,0,0,0,25621],"quality":"Less is better","isMain":false,"metricName":"Partially unaligned length"}]],["Mismatches",[{"values":[2,0,48,2,23,397,0,0,1,95,2,1,0,498],"quality":"Less is better","isMain":false,"metricName":"# mismatches"},{"values":[0,0,0,0,0,8,0,12,0,10,0,0,0,11],"quality":"Less is better","isMain":false,"metricName":"# indels"},{"values":[0,0,0,0,0,8,0,601,0,10,0,0,0,11],"quality":"Less is better","isMain":false,"metricName":"Indels length"},{"values":["6.63","0.00","128.33","6.63","64.63","914.33","0.00","0.00","2.51","898.60","8.68","4.18","0.00","1147.07"],"quality":"Less is better","isMain":true,"metricName":"# mismatches per 100 kbp"},{"values":["0.00","0.00","0.00","0.00","0.00","18.42","0.00","105.14","0.00","94.59","0.00","0.00","0.00","25.34"],"quality":"Less is better","isMain":true,"metricName":"# indels per 100 kbp"},{"values":[0,0,0,0,0,8,0,0,0,10,0,0,0,11],"quality":"Less is better","isMain":false,"metricName":" # indels (<= 5 bp)"},{"values":[0,0,0,0,0,0,0,12,0,0,0,0,0,0],"quality":"Less is better","isMain":false,"metricName":" # indels (> 5 bp)"},{"values":[0,0,0,0,0,0,0,4935,0,0,0,0,0,182],"quality":"Less is better","isMain":false,"metricName":"# N's"},{"values":["0.00","0.00","0.00","0.00","0.00","0.00","0.00","23669.06","0.00","0.00","0.00","0.00","0.00","199.91"],"quality":"Less is better","isMain":true,"metricName":"# N's per 100 kbp"}]],["Statistics without reference",[{"values":[8,15,7,18,15,6,1,13,7,2,16,17,12,2],"quality":"Equal","isMain":true,"metricName":"# contigs"},{"values":[8,8,6,9,8,5,0,9,6,2,8,8,6,2],"quality":"Equal","isMain":false,"metricName":"# contigs (>= 1000 bp)"},{"values":[2,0,2,2,4,2,0,0,1,1,0,0,0,2],"quality":"Equal","isMain":false,"metricName":"# contigs (>= 5000 bp)"},{"values":[1,0,2,0,0,2,0,0,1,0,0,0,0,2],"quality":"Equal","isMain":false,"metricName":"# contigs (>= 10000 bp)"},{"values":[0,0,0,0,0,2,0,0,1,0,0,0,0,2],"quality":"Equal","isMain":false,"metricName":"# contigs (>= 25000 bp)"},{"values":[0,0,0,0,0,0,0,0,0,0,0,0,0,1],"quality":"Equal","isMain":false,"metricName":"# contigs (>= 50000 bp)"},{"values":[13127,3051,16636,5102,7707,44069,607,4335,31176,7763,3407,3463,2182,56062],"quality":"More is better","isMain":true,"metricName":"Largest contig"},{"values":[30473,21586,37403,30473,38740,83217,607,20850,39763,10570,23184,24090,14022,91042],"quality":"More is better","isMain":true,"metricName":"Total length"},{"values":[30473,16743,36827,23764,33585,82611,0,17826,39195,10570,17618,17894,9775,91042],"quality":"More is better","isMain":true,"metricName":"Total length (>= 1000 bp)"},{"values":[18292,0,30202,10118,26345,73818,0,0,31176,7763,0,0,0,91042],"quality":"More is better","isMain":false,"metricName":"Total length (>= 5000 bp)"},{"values":[13127,0,30202,0,0,73818,0,0,31176,0,0,0,0,91042],"quality":"More is better","isMain":true,"metricName":"Total length (>= 10000 bp)"},{"values":[0,0,0,0,0,73818,0,0,31176,0,0,0,0,91042],"quality":"More is better","isMain":false,"metricName":"Total length (>= 25000 bp)"},{"values":[0,0,0,0,0,0,0,0,0,0,0,0,0,56062],"quality":"More is better","isMain":true,"metricName":"Total length (>= 50000 bp)"},{"values":[5165,2006,13566,2579,5733,44069,607,1746,31176,7763,1842,1842,1512,56062],"quality":"More is better","isMain":false,"metricName":"N50"},{"values":[2366,1392,13566,1015,2011,29749,607,1226,31176,2807,1434,935,921,34980],"quality":"More is better","isMain":false,"metricName":"N75"},{"values":[2,5,2,4,3,1,1,4,1,1,5,5,4,1],"quality":"Less is better","isMain":false,"metricName":"L50"},{"values":[4,8,2,9,6,2,1,7,1,2,8,9,7,2],"quality":"Less is better","isMain":false,"metricName":"L75"},{"values":["34.18","33.64","34.75","34.16","34.78","34.82","33.94","34.11","34.61","33.84","33.70","33.81","32.98","34.75"],"quality":"Equal","isMain":false,"metricName":"GC (%)"}]],["Predicted genes",[]],["Similarity statistics",[{"values":[0,0,0,0,0,0,0,0,0,0,0,0,0,0],"quality":"Equal","isMain":false,"metricName":"# similar correct contigs"},{"values":[0,0,0,0,0,0,0,0,0,0,0,0,0,0],"quality":"Equal","isMain":false,"metricName":"# similar misassembled blocks"}]],["Reference statistics",[{"values":[43420,43420,43420,43420,43420,43420,43420,43420,43420,43420,43420,43420,43420,43420],"quality":"Equal","isMain":false,"metricName":"Reference length"},{"values":[1,1,1,1,1,1,1,1,1,1,1,1,1,1],"quality":"Equal","isMain":false,"metricName":"Reference fragments"},{"values":["34.68","34.68","34.68","34.68","34.68","34.68","34.68","34.68","34.68","34.68","34.68","34.68","34.68","34.68"],"quality":"Equal","isMain":false,"metricName":"Reference GC (%)"}]]],"referenceName":"gi\_119953678\_ref\_NC","date":"18 July 2018, Wednesday, 16:11:58","order":[0,1,2,3,4,5,6,7,8,9,10,11,12,13],"assembliesNames":["ABySS\_127","ABySS\_63","CLC","IDBA\_UD","MEGAHIT","MIRA","Ray\_Meta","SOAPdenovo2","SPAdes","SPAdes\_meta","SPAdes\_sc","SPAdes\_sc\_careful","Velvet","Geneious"]},{"assembliesWithNs":null,"minContig":500,"report":[["Genome statistics",[{"values":["97.144","93.464","99.019","99.611","99.694","98.658","93.835","99.209","99.578","98.811","98.811","96.999","55.799"],"quality":"More is better","isMain":true,"metricName":"Genome fraction (%)"},{"values":["1.001","1.002","1.006","1.001","1.000","1.023","1.032","1.000","1.001","1.002","1.002","1.001","1.001"],"quality":"Less is better","isMain":true,"metricName":"Duplication ratio"},{"values":[17271,10518,40294,47952,46206,42314,15485,64369,64436,56631,56568,17366,42314],"quality":"More is better","isMain":true,"metricName":"Largest alignment"},{"values":[280075,269854,286934,287122,287301,290731,272258,285787,287038,285179,285114,279694,160679],"quality":"More is better","isMain":true,"metricName":"Total aligned length"},{"values":[4547,3618,17425,32128,34853,28866,6641,42592,38869,33433,33431,9319,14561],"quality":"More is better","isMain":false,"metricName":"NG50"},{"values":[2659,1806,10065,20464,29113,13465,3661,39059,29161,29161,29161,4452,null],"quality":"More is better","isMain":false,"metricName":"NG75"},{"values":[4983,3822,17425,32128,34853,28866,6504,42592,38846,33433,33431,9968,32545],"quality":"More is better","isMain":false,"metricName":"NA50"},{"values":[3039,2454,10065,20464,29113,13465,3600,39036,29161,29161,29161,4737,30087],"quality":"More is better","isMain":false,"metricName":"NA75"},{"values":[4547,3618,17425,32128,34853,28866,6417,42592,38846,33433,33431,9319,14561],"quality":"More is better","isMain":true,"metricName":"NGA50"},{"values":[2659,1806,10065,20464,29113,13465,3481,39036,29161,29161,29161,4452,null],"quality":"More is better","isMain":false,"metricName":"NGA75"},{"values":[18,27,6,4,4,4,15,3,3,4,4,12,5],"quality":"Less is better","isMain":false,"metricName":"LG50"},{"values":[37,53,11,7,6,9,29,5,6,6,6,22,null],"quality":"Less is better","isMain":false,"metricName":"LG75"},{"values":[17,25,6,4,4,4,15,3,3,4,4,11,3],"quality":"Less is better","isMain":false,"metricName":"LA50"},{"values":[35,47,11,7,6,9,29,5,6,6,6,21,4],"quality":"Less is better","isMain":false,"metricName":"LA75"},{"values":[18,27,6,4,4,4,16,3,3,4,4,12,5],"quality":"Less is better","isMain":true,"metricName":"LGA50"},{"values":[37,53,11,7,6,9,30,5,6,6,6,22,null],"quality":"Less is better","isMain":false,"metricName":"LGA75"}]],["Misassemblies",[{"values":[0,0,0,0,0,0,0,0,0,0,0,0,0],"quality":"Less is better","isMain":true,"metricName":"# misassemblies"},{"values":[0,0,0,0,0,0,0,0,0,0,0,0,0],"quality":"Less is better","isMain":false,"metricName":" # relocations"},{"values":[0,0,0,0,0,0,0,0,0,0,0,0,0],"quality":"Less is better","isMain":false,"metricName":" # translocations"},{"values":[0,0,0,0,0,0,0,0,0,0,0,0,0],"quality":"Less is better","isMain":false,"metricName":" # inversions"},{"values":[0,0,0,0,0,0,0,0,0,0,0,0,0],"quality":"Less is better","isMain":false,"metricName":"# misassembled contigs"},{"values":[0,0,0,0,0,0,0,0,0,0,0,0,0],"quality":"Less is better","isMain":true,"metricName":"Misassembled contigs length"},{"values":[0,0,0,0,1,2,0,1,1,5,5,0,1],"quality":"Less is better","isMain":false,"metricName":"# local misassemblies"},{"values":[0,0,0,0,0,0,1,0,0,0,0,0,0],"quality":"Less is better","isMain":false,"metricName":"# unaligned mis. contigs"}]],["Unaligned",[{"values":[0,0,0,0,0,0,0,0,0,0,0,0,0],"quality":"Less is better","isMain":false,"metricName":"# fully unaligned contigs"},{"values":[0,0,0,0,0,0,0,0,0,0,0,0,0],"quality":"Less is better","isMain":false,"metricName":"Fully unaligned length"},{"values":[0,0,0,0,0,0,5,0,0,2,2,0,0],"quality":"Less is better","isMain":false,"metricName":"# partially unaligned contigs"},{"values":[0,0,0,0,0,0,4132,0,0,1506,1506,0,0],"quality":"Less is better","isMain":false,"metricName":"Partially unaligned length"}]],["Mismatches",[{"values":[21,5,17,8,9,7,9,26,54,35,25,9,1],"quality":"Less is better","isMain":false,"metricName":"# mismatches"},{"values":[0,0,0,1,1,3,89,0,4,5,4,4,2],"quality":"Less is better","isMain":false,"metricName":"# indels"},{"values":[0,0,0,27,1,121,4315,0,27,91,52,90,120],"quality":"Less is better","isMain":false,"metricName":"Indels length"},{"values":["7.50","1.86","5.96","2.79","3.13","2.46","3.33","9.10","18.83","12.30","8.78","3.22","0.62"],"quality":"Less is better","isMain":true,"metricName":"# mismatches per 100 kbp"},{"values":["0.00","0.00","0.00","0.35","0.35","1.06","32.93","0.00","1.39","1.76","1.41","1.43","1.24"],"quality":"Less is better","isMain":true,"metricName":"# indels per 100 kbp"},{"values":[0,0,0,0,1,1,0,0,2,2,2,1,0],"quality":"Less is better","isMain":false,"metricName":" # indels (<= 5 bp)"},{"values":[0,0,0,1,0,2,89,0,2,3,2,3,2],"quality":"Less is better","isMain":false,"metricName":" # indels (> 5 bp)"},{"values":[0,0,0,0,0,59,9754,0,100,1529,1499,10,136],"quality":"Less is better","isMain":false,"metricName":"# N's"},{"values":["0.00","0.00","0.00","0.00","0.00","20.29","3445.69","0.00","34.82","533.30","522.95","3.57","84.50"],"quality":"Less is better","isMain":true,"metricName":"# N's per 100 kbp"}]],["Statistics without reference",[{"values":[83,108,28,17,15,28,64,8,10,16,16,51,6],"quality":"Equal","isMain":true,"metricName":"# contigs"},{"values":[67,73,24,14,15,21,54,8,10,11,11,44,6],"quality":"Equal","isMain":false,"metricName":"# contigs (>= 1000 bp)"},{"values":[16,11,19,11,11,13,25,8,10,11,11,20,5],"quality":"Equal","isMain":false,"metricName":"# contigs (>= 5000 bp)"},{"values":[4,1,11,9,8,10,4,7,8,7,7,10,5],"quality":"Equal","isMain":false,"metricName":"# contigs (>= 10000 bp)"},{"values":[0,0,3,6,6,4,0,6,6,6,6,0,4],"quality":"Equal","isMain":false,"metricName":"# contigs (>= 25000 bp)"},{"values":[0,0,0,0,0,0,0,1,1,1,1,0,0],"quality":"Equal","isMain":false,"metricName":"# contigs (>= 50000 bp)"},{"values":[17271,10518,40294,47952,46206,42314,16065,64369,64536,56631,56568,17366,42314],"quality":"More is better","isMain":true,"metricName":"Largest contig"},{"values":[280098,269854,286966,287122,287301,290731,283078,285810,287161,286708,286643,279733,160942],"quality":"More is better","isMain":true,"metricName":"Total length"},{"values":[268340,244224,283798,285151,287301,285519,275665,285810,287161,283326,283261,275015,160942],"quality":"More is better","isMain":true,"metricName":"Total length (>= 1000 bp)"},{"values":[137140,79001,274432,278158,277869,264903,202590,285810,287161,283326,283261,207625,156169],"quality":"More is better","isMain":false,"metricName":"Total length (>= 5000 bp)"},{"values":[51140,10518,216218,259153,250482,240355,57118,276497,268026,248468,248403,133751,156169],"quality":"More is better","isMain":true,"metricName":"Total length (>= 10000 bp)"},{"values":[0,0,104979,214251,223698,149270,0,262740,243085,234711,234646,0,141608],"quality":"More is better","isMain":false,"metricName":"Total length (>= 25000 bp)"},{"values":[0,0,0,0,0,0,0,64369,64536,56631,56568,0,0],"quality":"More is better","isMain":true,"metricName":"Total length (>= 50000 bp)"},{"values":[4983,3822,17425,32128,34853,28866,6641,42592,38869,33433,33431,9968,32545],"quality":"More is better","isMain":false,"metricName":"N50"},{"values":[3039,2454,10065,20464,29113,13465,4044,39059,29161,29161,29161,4737,30087],"quality":"More is better","isMain":false,"metricName":"N75"},{"values":[17,25,6,4,4,4,15,3,3,4,4,11,3],"quality":"Less is better","isMain":false,"metricName":"L50"},{"values":[35,47,11,7,6,9,28,5,6,6,6,21,4],"quality":"Less is better","isMain":false,"metricName":"L75"},{"values":["49.39","49.49","49.40","49.40","49.40","49.42","49.25","49.38","49.40","49.37","49.37","49.24","49.79"],"quality":"Equal","isMain":false,"metricName":"GC (%)"}]],["Predicted genes",[]],["Similarity statistics",[{"values":[0,0,1,2,2,2,1,2,2,2,2,1,0],"quality":"Equal","isMain":false,"metricName":"# similar correct contigs"},{"values":[0,0,0,0,0,0,0,0,0,0,0,0,0],"quality":"Equal","isMain":false,"metricName":"# similar misassembled blocks"}]],["Reference statistics",[{"values":[288047,288047,288047,288047,288047,288047,288047,288047,288047,288047,288047,288047,288047],"quality":"Equal","isMain":false,"metricName":"Reference length"},{"values":[1,1,1,1,1,1,1,1,1,1,1,1,1],"quality":"Equal","isMain":false,"metricName":"Reference fragments"},{"values":["49.40","49.40","49.40","49.40","49.40","49.40","49.40","49.40","49.40","49.40","49.40","49.40","49.40"],"quality":"Equal","isMain":false,"metricName":"Reference GC (%)"}]]],"referenceName":"gi\_119953744\_ref\_NC","date":"18 July 2018, Wednesday, 16:12:10","order":[0,1,2,3,4,5,6,7,8,9,10,11,12],"assembliesNames":["ABySS\_127","ABySS\_63","CLC","IDBA\_UD","MEGAHIT","MIRA","SOAPdenovo2","SPAdes","SPAdes\_meta","SPAdes\_sc","SPAdes\_sc\_careful","Velvet","Geneious"]},{"assembliesWithNs":null,"minContig":500,"report":[["Genome statistics",[{"values":["99.816","99.535","100.000","95.422","99.850","100.000","98.281","4.444","99.180","100.000","97.255","92.814","40.603","100.000"],"quality":"More is better","isMain":true,"metricName":"Genome fraction (%)"},{"values":["1.003","1.037","1.042","1.034","1.090","1.810","1.032","1.000","1.023","1.004","1.028","1.019","1.002","1.530"],"quality":"Less is better","isMain":true,"metricName":"Duplication ratio"},{"values":[43960,18760,32356,5864,15070,27981,15661,714,34309,16086,14052,14349,3047,33206],"quality":"More is better","isMain":true,"metricName":"Largest alignment"},{"values":[43960,45438,45906,43438,47915,79703,44664,1957,44696,44206,43492,41426,17915,67385],"quality":"More is better","isMain":true,"metricName":"Total aligned length"},{"values":[44091,11654,32356,2856,7707,44069,12142,null,34309,14480,9843,4062,null,56062],"quality":"More is better","isMain":false,"metricName":"NG50"},{"values":[44091,9558,6371,1593,5599,44069,11700,null,34309,13640,3673,1375,null,56062],"quality":"More is better","isMain":false,"metricName":"NG75"},{"values":[43960,11654,32356,3642,5764,8042,12142,704,34309,14480,9283,4062,1053,17964],"quality":"More is better","isMain":false,"metricName":"NA50"},{"values":[43960,9558,6371,1786,2458,758,11700,539,34309,13640,3673,1627,820,null],"quality":"More is better","isMain":false,"metricName":"NA75"},{"values":[43960,11654,32356,2856,5764,27981,12142,null,34309,14480,9283,4062,null,33206],"quality":"More is better","isMain":true,"metricName":"NGA50"},{"values":[43960,9558,6371,1593,3922,15660,11700,null,34309,13640,3673,1375,null,33206],"quality":"More is better","isMain":false,"metricName":"NGA75"},{"values":[1,2,1,6,2,1,2,null,1,2,2,3,null,1],"quality":"Less is better","isMain":false,"metricName":"LG50"},{"values":[1,3,2,11,4,1,3,null,1,3,4,8,null,1],"quality":"Less is better","isMain":false,"metricName":"LG75"},{"values":[1,2,1,5,3,3,2,2,1,2,2,3,5,2],"quality":"Less is better","isMain":false,"metricName":"LA50"},{"values":[1,3,2,10,6,13,3,3,1,3,4,7,10,null],"quality":"Less is better","isMain":false,"metricName":"LA75"},{"values":[1,2,1,6,3,1,2,null,1,2,2,3,null,1],"quality":"Less is better","isMain":true,"metricName":"LGA50"},{"values":[1,3,2,11,5,2,3,null,1,3,4,8,null,1],"quality":"Less is better","isMain":false,"metricName":"LGA75"}]],["Misassemblies",[{"values":[0,0,0,0,0,1,0,0,0,0,0,0,0,1],"quality":"Less is better","isMain":true,"metricName":"# misassemblies"},{"values":[0,0,0,0,0,1,0,0,0,0,0,0,0,1],"quality":"Less is better","isMain":false,"metricName":" # relocations"},{"values":[0,0,0,0,0,0,0,0,0,0,0,0,0,0],"quality":"Less is better","isMain":false,"metricName":" # translocations"},{"values":[0,0,0,0,0,0,0,0,0,0,0,0,0,0],"quality":"Less is better","isMain":false,"metricName":" # inversions"},{"values":[0,0,0,0,0,1,0,0,0,0,0,0,0,1],"quality":"Less is better","isMain":false,"metricName":"# misassembled contigs"},{"values":[0,0,0,0,0,44069,0,0,0,0,0,0,0,56062],"quality":"Less is better","isMain":true,"metricName":"Misassembled contigs length"},{"values":[0,0,0,0,1,2,0,0,0,0,0,0,0,3],"quality":"Less is better","isMain":false,"metricName":"# local misassemblies"},{"values":[0,0,0,0,0,0,0,0,0,0,0,0,0,0],"quality":"Less is better","isMain":false,"metricName":"# unaligned mis. contigs"}]],["Unaligned",[{"values":[0,0,0,0,0,0,0,0,0,0,0,0,0,0],"quality":"Less is better","isMain":false,"metricName":"# fully unaligned contigs"},{"values":[0,0,0,0,0,0,0,0,0,0,0,0,0,0],"quality":"Less is better","isMain":false,"metricName":"Fully unaligned length"},{"values":[0,0,0,0,1,2,0,0,0,0,0,0,0,2],"quality":"Less is better","isMain":false,"metricName":"# partially unaligned contigs"},{"values":[0,0,0,0,1943,22135,0,0,0,0,0,0,0,23657],"quality":"Less is better","isMain":false,"metricName":"Partially unaligned length"}]],["Mismatches",[{"values":[0,0,0,0,167,398,1,2,0,1,0,2,2,194],"quality":"Less is better","isMain":false,"metricName":"# mismatches"},{"values":[0,0,0,0,0,7,0,0,0,0,0,1,0,5],"quality":"Less is better","isMain":false,"metricName":"# indels"},{"values":[0,0,0,0,0,7,0,0,0,0,0,1,0,5],"quality":"Less is better","isMain":false,"metricName":"Indels length"},{"values":["0.00","0.00","0.00","0.00","379.76","903.70","2.31","102.20","0.00","2.27","0.00","4.89","11.18","440.50"],"quality":"Less is better","isMain":true,"metricName":"# mismatches per 100 kbp"},{"values":["0.00","0.00","0.00","0.00","0.00","15.89","0.00","0.00","0.00","0.00","0.00","2.45","0.00","11.35"],"quality":"Less is better","isMain":true,"metricName":"# indels per 100 kbp"},{"values":[0,0,0,0,0,7,0,0,0,0,0,1,0,5],"quality":"Less is better","isMain":false,"metricName":" # indels (<= 5 bp)"},{"values":[0,0,0,0,0,0,0,0,0,0,0,0,0,0],"quality":"Less is better","isMain":false,"metricName":" # indels (> 5 bp)"},{"values":[0,8,0,0,0,1,0,0,0,0,560,204,0,182],"quality":"Less is better","isMain":false,"metricName":"# N's"},{"values":["0.00","17.61","0.00","0.00","0.00","0.98","0.00","0.00","0.00","0.00","1271.22","490.00","0.00","199.91"],"quality":"Less is better","isMain":true,"metricName":"# N's per 100 kbp"}]],["Statistics without reference",[{"values":[1,5,8,20,13,17,4,3,10,3,14,19,17,2],"quality":"Equal","isMain":true,"metricName":"# contigs"},{"values":[1,4,6,16,11,8,4,0,7,3,8,9,5,2],"quality":"Equal","isMain":false,"metricName":"# contigs (>= 1000 bp)"},{"values":[1,3,2,1,4,3,4,0,1,3,3,2,0,2],"quality":"Equal","isMain":false,"metricName":"# contigs (>= 5000 bp)"},{"values":[1,2,1,0,1,2,3,0,1,3,1,1,0,2],"quality":"Equal","isMain":false,"metricName":"# contigs (>= 10000 bp)"},{"values":[1,0,1,0,0,2,0,0,1,0,0,0,0,2],"quality":"Equal","isMain":false,"metricName":"# contigs (>= 25000 bp)"},{"values":[0,0,0,0,0,0,0,0,0,0,0,0,0,1],"quality":"Equal","isMain":false,"metricName":"# contigs (>= 50000 bp)"},{"values":[44091,18760,32356,5864,15070,44069,15663,714,34309,16086,14052,14349,3047,56062],"quality":"More is better","isMain":true,"metricName":"Largest contig"},{"values":[44091,45438,45906,43438,49858,101838,44666,1957,44696,44206,44052,41633,17915,91042],"quality":"More is better","isMain":true,"metricName":"Total length"},{"values":[44091,44910,44742,40455,48466,95382,44666,0,42924,44206,39661,35163,9104,91042],"quality":"More is better","isMain":true,"metricName":"Total length (>= 1000 bp)"},{"values":[44091,39972,38727,5864,34176,80282,44666,0,34309,44206,30298,20752,0,91042],"quality":"More is better","isMain":false,"metricName":"Total length (>= 5000 bp)"},{"values":[44091,30414,32356,0,15070,73818,39505,0,34309,44206,14052,14349,0,91042],"quality":"More is better","isMain":true,"metricName":"Total length (>= 10000 bp)"},{"values":[44091,0,32356,0,0,73818,0,0,34309,0,0,0,0,91042],"quality":"More is better","isMain":false,"metricName":"Total length (>= 25000 bp)"},{"values":[0,0,0,0,0,0,0,0,0,0,0,0,0,56062],"quality":"More is better","isMain":true,"metricName":"Total length (>= 50000 bp)"},{"values":[44091,11654,32356,3642,5800,29749,12142,704,34309,14480,9843,4062,1053,56062],"quality":"More is better","isMain":false,"metricName":"N50"},{"values":[44091,9558,6371,1786,3922,6464,11700,539,34309,13640,3673,1627,820,34980],"quality":"More is better","isMain":false,"metricName":"N75"},{"values":[1,2,1,5,3,2,2,2,1,2,2,3,5,1],"quality":"Less is better","isMain":false,"metricName":"L50"},{"values":[1,3,2,10,5,3,3,3,1,3,4,7,10,2],"quality":"Less is better","isMain":false,"metricName":"L75"},{"values":["34.91","34.93","34.74","34.82","34.92","34.73","34.99","34.90","34.92","34.93","34.85","34.82","34.07","34.75"],"quality":"Equal","isMain":false,"metricName":"GC (%)"}]],["Predicted genes",[]],["Similarity statistics",[{"values":[0,0,0,0,0,0,0,0,0,0,0,0,0,0],"quality":"Equal","isMain":false,"metricName":"# similar correct contigs"},{"values":[0,0,0,0,0,0,0,0,0,0,0,0,0,0],"quality":"Equal","isMain":false,"metricName":"# similar misassembled blocks"}]],["Reference statistics",[{"values":[44041,44041,44041,44041,44041,44041,44041,44041,44041,44041,44041,44041,44041,44041],"quality":"Equal","isMain":false,"metricName":"Reference length"},{"values":[1,1,1,1,1,1,1,1,1,1,1,1,1,1],"quality":"Equal","isMain":false,"metricName":"Reference fragments"},{"values":["34.91","34.91","34.91","34.91","34.91","34.91","34.91","34.91","34.91","34.91","34.91","34.91","34.91","34.91"],"quality":"Equal","isMain":false,"metricName":"Reference GC (%)"}]]],"referenceName":"gi\_119967833\_ref\_NC","date":"18 July 2018, Wednesday, 16:12:22","order":[0,1,2,3,4,5,6,7,8,9,10,11,12,13],"assembliesNames":["ABySS\_127","ABySS\_63","CLC","IDBA\_UD","MEGAHIT","MIRA","Ray\_Meta","SOAPdenovo2","SPAdes","SPAdes\_meta","SPAdes\_sc","SPAdes\_sc\_careful","Velvet","Geneious"]},{"assembliesWithNs":null,"minContig":500,"report":[["Genome statistics",[{"values":["100.000","100.000","100.000","100.000","100.000","100.000","100.000"],"quality":"More is better","isMain":true,"metricName":"Genome fraction (%)"},{"values":["1.000","243.432","1.028","1.012","1.012","1.012","1.670"],"quality":"Less is better","isMain":true,"metricName":"Duplication ratio"},{"values":[4594,1847,4721,4649,4649,4649,4127],"quality":"More is better","isMain":true,"metricName":"Largest alignment"},{"values":[4594,1116731,4721,4649,4649,4649,7670],"quality":"More is better","isMain":true,"metricName":"Total aligned length"},{"values":[4594,1502,4721,4649,4649,4649,7670],"quality":"More is better","isMain":false,"metricName":"NG50"},{"values":[4594,1458,4721,4649,4649,4649,7670],"quality":"More is better","isMain":false,"metricName":"NG75"},{"values":[4594,719,4721,4649,4649,4649,4127],"quality":"More is better","isMain":false,"metricName":"NA50"},{"values":[4594,576,4721,4649,4649,4649,3543],"quality":"More is better","isMain":false,"metricName":"NA75"},{"values":[4594,1502,4721,4649,4649,4649,4127],"quality":"More is better","isMain":true,"metricName":"NGA50"},{"values":[4594,1458,4721,4649,4649,4649,4127],"quality":"More is better","isMain":false,"metricName":"NGA75"},{"values":[1,2,1,1,1,1,1],"quality":"Less is better","isMain":false,"metricName":"LG50"},{"values":[1,3,1,1,1,1,1],"quality":"Less is better","isMain":false,"metricName":"LG75"},{"values":[1,634,1,1,1,1,1],"quality":"Less is better","isMain":false,"metricName":"LA50"},{"values":[1,1085,1,1,1,1,2],"quality":"Less is better","isMain":false,"metricName":"LA75"},{"values":[1,2,1,1,1,1,1],"quality":"Less is better","isMain":true,"metricName":"LGA50"},{"values":[1,3,1,1,1,1,1],"quality":"Less is better","isMain":false,"metricName":"LGA75"}]],["Misassemblies",[{"values":[0,0,0,0,0,0,1],"quality":"Less is better","isMain":true,"metricName":"# misassemblies"},{"values":[0,0,0,0,0,0,1],"quality":"Less is better","isMain":false,"metricName":" # relocations"},{"values":[0,0,0,0,0,0,0],"quality":"Less is better","isMain":false,"metricName":" # translocations"},{"values":[0,0,0,0,0,0,0],"quality":"Less is better","isMain":false,"metricName":" # inversions"},{"values":[0,0,0,0,0,0,1],"quality":"Less is better","isMain":false,"metricName":"# misassembled contigs"},{"values":[0,0,0,0,0,0,7670],"quality":"Less is better","isMain":true,"metricName":"Misassembled contigs length"},{"values":[0,0,1,0,0,0,0],"quality":"Less is better","isMain":false,"metricName":"# local misassemblies"},{"values":[0,0,0,0,0,0,0],"quality":"Less is better","isMain":false,"metricName":"# unaligned mis. contigs"}]],["Unaligned",[{"values":[0,0,0,0,0,0,0],"quality":"Less is better","isMain":false,"metricName":"# fully unaligned contigs"},{"values":[0,0,0,0,0,0,0],"quality":"Less is better","isMain":false,"metricName":"Fully unaligned length"},{"values":[0,0,0,0,0,0,0],"quality":"Less is better","isMain":false,"metricName":"# partially unaligned contigs"},{"values":[0,0,0,0,0,0,0],"quality":"Less is better","isMain":false,"metricName":"Partially unaligned length"}]],["Mismatches",[{"values":[0,12,0,0,0,0,2],"quality":"Less is better","isMain":false,"metricName":"# mismatches"},{"values":[0,0,0,1,1,1,0],"quality":"Less is better","isMain":false,"metricName":"# indels"},{"values":[0,0,0,55,55,55,0],"quality":"Less is better","isMain":false,"metricName":"Indels length"},{"values":["0.00","261.21","0.00","0.00","0.00","0.00","43.54"],"quality":"Less is better","isMain":true,"metricName":"# mismatches per 100 kbp"},{"values":["0.00","0.00","0.00","21.77","21.77","21.77","0.00"],"quality":"Less is better","isMain":true,"metricName":"# indels per 100 kbp"},{"values":[0,0,0,0,0,0,0],"quality":"Less is better","isMain":false,"metricName":" # indels (<= 5 bp)"},{"values":[0,0,0,1,1,1,0],"quality":"Less is better","isMain":false,"metricName":" # indels (> 5 bp)"},{"values":[0,37,0,0,0,0,0],"quality":"Less is better","isMain":false,"metricName":"# N's"},{"values":["0.00","3.31","0.00","0.00","0.00","0.00","0.00"],"quality":"Less is better","isMain":true,"metricName":"# N's per 100 kbp"}]],["Statistics without reference",[{"values":[1,1589,1,1,1,1,1],"quality":"Equal","isMain":true,"metricName":"# contigs"},{"values":[1,127,1,1,1,1,1],"quality":"Equal","isMain":false,"metricName":"# contigs (>= 1000 bp)"},{"values":[0,0,0,0,0,0,1],"quality":"Equal","isMain":false,"metricName":"# contigs (>= 5000 bp)"},{"values":[0,0,0,0,0,0,0],"quality":"Equal","isMain":false,"metricName":"# contigs (>= 10000 bp)"},{"values":[0,0,0,0,0,0,0],"quality":"Equal","isMain":false,"metricName":"# contigs (>= 25000 bp)"},{"values":[0,0,0,0,0,0,0],"quality":"Equal","isMain":false,"metricName":"# contigs (>= 50000 bp)"},{"values":[4594,1847,4721,4649,4649,4649,7670],"quality":"More is better","isMain":true,"metricName":"Largest contig"},{"values":[4594,1118327,4721,4649,4649,4649,7670],"quality":"More is better","isMain":true,"metricName":"Total length"},{"values":[4594,143797,4721,4649,4649,4649,7670],"quality":"More is better","isMain":true,"metricName":"Total length (>= 1000 bp)"},{"values":[0,0,0,0,0,0,7670],"quality":"More is better","isMain":false,"metricName":"Total length (>= 5000 bp)"},{"values":[0,0,0,0,0,0,0],"quality":"More is better","isMain":true,"metricName":"Total length (>= 10000 bp)"},{"values":[0,0,0,0,0,0,0],"quality":"More is better","isMain":false,"metricName":"Total length (>= 25000 bp)"},{"values":[0,0,0,0,0,0,0],"quality":"More is better","isMain":true,"metricName":"Total length (>= 50000 bp)"},{"values":[4594,721,4721,4649,4649,4649,7670],"quality":"More is better","isMain":false,"metricName":"N50"},{"values":[4594,577,4721,4649,4649,4649,7670],"quality":"More is better","isMain":false,"metricName":"N75"},{"values":[1,634,1,1,1,1,1],"quality":"Less is better","isMain":false,"metricName":"L50"},{"values":[1,1083,1,1,1,1,1],"quality":"Less is better","isMain":false,"metricName":"L75"},{"values":["46.10","46.07","45.88","46.14","46.14","46.14","46.48"],"quality":"Equal","isMain":false,"metricName":"GC (%)"}]],["Predicted genes",[]],["Similarity statistics",[{"values":[0,0,0,0,0,0,0],"quality":"Equal","isMain":false,"metricName":"# similar correct contigs"},{"values":[0,0,0,0,0,0,0],"quality":"Equal","isMain":false,"metricName":"# similar misassembled blocks"}]],["Reference statistics",[{"values":[4594,4594,4594,4594,4594,4594,4594],"quality":"Equal","isMain":false,"metricName":"Reference length"},{"values":[1,1,1,1,1,1,1],"quality":"Equal","isMain":false,"metricName":"Reference fragments"},{"values":["46.10","46.10","46.10","46.10","46.10","46.10","46.10"],"quality":"Equal","isMain":false,"metricName":"Reference GC (%)"}]]],"referenceName":"gi\_12085135\_ref\_NC","date":"18 July 2018, Wednesday, 16:12:49","order":[0,1,2,3,4,5,6],"assembliesNames":["CLC","MIRA","SPAdes","SPAdes\_meta","SPAdes\_sc","SPAdes\_sc\_careful","Geneious"]},{"assembliesWithNs":null,"minContig":500,"report":[["Genome statistics",[{"values":["100.000","63.240","2.513","100.000","100.000","100.000","100.000","100.000","100.000","2.499","100.000"],"quality":"More is better","isMain":true,"metricName":"Genome fraction (%)"},{"values":["1.000","1.007","1.000","325.750","1.002","1.006","1.003","1.003","1.003","1.000","2.981"],"quality":"Less is better","isMain":true,"metricName":"Duplication ratio"},{"values":[21129,1310,531,21602,21178,21256,21184,21184,21184,528,21129],"quality":"More is better","isMain":true,"metricName":"Largest alignment"},{"values":[21129,13461,531,6881197,21178,21256,21184,21184,21184,528,62994],"quality":"More is better","isMain":true,"metricName":"Total aligned length"},{"values":[21129,607,null,26186,21178,21256,21184,21184,21184,null,63664],"quality":"More is better","isMain":false,"metricName":"NG50"},{"values":[21129,null,null,26186,21178,21256,21184,21184,21184,null,63664],"quality":"More is better","isMain":false,"metricName":"NG75"},{"values":[21129,828,531,3759,21178,21256,21184,21184,21184,528,21129],"quality":"More is better","isMain":false,"metricName":"NA50"},{"values":[21129,690,531,2339,21178,21256,21184,21184,21184,528,15647],"quality":"More is better","isMain":false,"metricName":"NA75"},{"values":[21129,607,null,21602,21178,21256,21184,21184,21184,null,21129],"quality":"More is better","isMain":true,"metricName":"NGA50"},{"values":[21129,null,null,21602,21178,21256,21184,21184,21184,null,21129],"quality":"More is better","isMain":false,"metricName":"NGA75"},{"values":[1,12,null,1,1,1,1,1,1,null,1],"quality":"Less is better","isMain":false,"metricName":"LG50"},{"values":[1,null,null,1,1,1,1,1,1,null,1],"quality":"Less is better","isMain":false,"metricName":"LG75"},{"values":[1,7,1,508,1,1,1,1,1,1,2],"quality":"Less is better","isMain":false,"metricName":"LA50"},{"values":[1,11,1,1098,1,1,1,1,1,1,3],"quality":"Less is better","isMain":false,"metricName":"LA75"},{"values":[1,12,null,1,1,1,1,1,1,null,1],"quality":"Less is better","isMain":true,"metricName":"LGA50"},{"values":[1,null,null,1,1,1,1,1,1,null,1],"quality":"Less is better","isMain":false,"metricName":"LGA75"}]],["Misassemblies",[{"values":[0,0,0,8,0,0,0,0,0,0,3],"quality":"Less is better","isMain":true,"metricName":"# misassemblies"},{"values":[0,0,0,8,0,0,0,0,0,0,3],"quality":"Less is better","isMain":false,"metricName":" # relocations"},{"values":[0,0,0,0,0,0,0,0,0,0,0],"quality":"Less is better","isMain":false,"metricName":" # translocations"},{"values":[0,0,0,0,0,0,0,0,0,0,0],"quality":"Less is better","isMain":false,"metricName":" # inversions"},{"values":[0,0,0,8,0,0,0,0,0,0,1],"quality":"Less is better","isMain":false,"metricName":"# misassembled contigs"},{"values":[0,0,0,189753,0,0,0,0,0,0,63664],"quality":"Less is better","isMain":true,"metricName":"Misassembled contigs length"},{"values":[0,0,0,1,0,1,0,0,0,0,0],"quality":"Less is better","isMain":false,"metricName":"# local misassemblies"},{"values":[0,0,0,0,0,0,0,0,0,0,0],"quality":"Less is better","isMain":false,"metricName":"# unaligned mis. contigs"}]],["Unaligned",[{"values":[0,0,0,0,0,0,0,0,0,0,0],"quality":"Less is better","isMain":false,"metricName":"# fully unaligned contigs"},{"values":[0,0,0,0,0,0,0,0,0,0,0],"quality":"Less is better","isMain":false,"metricName":"Fully unaligned length"},{"values":[0,0,0,0,0,0,0,0,0,0,1],"quality":"Less is better","isMain":false,"metricName":"# partially unaligned contigs"},{"values":[0,0,0,0,0,0,0,0,0,0,670],"quality":"Less is better","isMain":false,"metricName":"Partially unaligned length"}]],["Mismatches",[{"values":[0,0,3,68,0,1,2,1,0,0,96],"quality":"Less is better","isMain":false,"metricName":"# mismatches"},{"values":[0,0,0,6,1,0,1,1,1,0,0],"quality":"Less is better","isMain":false,"metricName":"# indels"},{"values":[0,0,0,6,49,0,55,55,55,0,0],"quality":"Less is better","isMain":false,"metricName":"Indels length"},{"values":["0.00","0.00","564.97","321.83","0.00","4.73","9.47","4.73","0.00","0.00","454.35"],"quality":"Less is better","isMain":true,"metricName":"# mismatches per 100 kbp"},{"values":["0.00","0.00","0.00","28.40","4.73","0.00","4.73","4.73","4.73","0.00","0.00"],"quality":"Less is better","isMain":true,"metricName":"# indels per 100 kbp"},{"values":[0,0,0,6,0,0,0,0,0,0,0],"quality":"Less is better","isMain":false,"metricName":" # indels (<= 5 bp)"},{"values":[0,0,0,0,1,0,1,1,1,0,0],"quality":"Less is better","isMain":false,"metricName":" # indels (> 5 bp)"},{"values":[0,0,0,2570,0,0,0,0,1,0,20],"quality":"Less is better","isMain":false,"metricName":"# N's"},{"values":["0.00","0.00","0.00","37.34","0.00","0.00","0.00","0.00","4.72","0.00","31.41"],"quality":"Less is better","isMain":true,"metricName":"# N's per 100 kbp"}]],["Statistics without reference",[{"values":[1,17,1,2042,1,1,1,1,1,1,1],"quality":"Equal","isMain":true,"metricName":"# contigs"},{"values":[1,2,0,2020,1,1,1,1,1,0,1],"quality":"Equal","isMain":false,"metricName":"# contigs (>= 1000 bp)"},{"values":[1,0,0,298,1,1,1,1,1,0,1],"quality":"Equal","isMain":false,"metricName":"# contigs (>= 5000 bp)"},{"values":[1,0,0,72,1,1,1,1,1,0,1],"quality":"Equal","isMain":false,"metricName":"# contigs (>= 10000 bp)"},{"values":[0,0,0,2,0,0,0,0,0,0,1],"quality":"Equal","isMain":false,"metricName":"# contigs (>= 25000 bp)"},{"values":[0,0,0,0,0,0,0,0,0,0,1],"quality":"Equal","isMain":false,"metricName":"# contigs (>= 50000 bp)"},{"values":[21129,1310,531,26186,21178,21256,21184,21184,21184,528,63664],"quality":"More is better","isMain":true,"metricName":"Largest contig"},{"values":[21129,13461,531,6882766,21178,21256,21184,21184,21184,528,63664],"quality":"More is better","isMain":true,"metricName":"Total length"},{"values":[21129,2574,0,6864388,21178,21256,21184,21184,21184,0,63664],"quality":"More is better","isMain":true,"metricName":"Total length (>= 1000 bp)"},{"values":[21129,0,0,2580192,21178,21256,21184,21184,21184,0,63664],"quality":"More is better","isMain":false,"metricName":"Total length (>= 5000 bp)"},{"values":[21129,0,0,1040959,21178,21256,21184,21184,21184,0,63664],"quality":"More is better","isMain":true,"metricName":"Total length (>= 10000 bp)"},{"values":[0,0,0,51538,0,0,0,0,0,0,63664],"quality":"More is better","isMain":false,"metricName":"Total length (>= 25000 bp)"},{"values":[0,0,0,0,0,0,0,0,0,0,63664],"quality":"More is better","isMain":true,"metricName":"Total length (>= 50000 bp)"},{"values":[21129,828,531,3768,21178,21256,21184,21184,21184,528,63664],"quality":"More is better","isMain":false,"metricName":"N50"},{"values":[21129,690,531,2340,21178,21256,21184,21184,21184,528,63664],"quality":"More is better","isMain":false,"metricName":"N75"},{"values":[1,7,1,500,1,1,1,1,1,1,1],"quality":"Less is better","isMain":false,"metricName":"L50"},{"values":[1,11,1,1089,1,1,1,1,1,1,1],"quality":"Less is better","isMain":false,"metricName":"L75"},{"values":["34.66","34.13","34.84","34.78","34.68","34.69","34.63","34.63","34.63","35.04","34.75"],"quality":"Equal","isMain":false,"metricName":"GC (%)"}]],["Predicted genes",[]],["Similarity statistics",[{"values":[0,0,0,0,0,0,0,0,0,0,0],"quality":"Equal","isMain":false,"metricName":"# similar correct contigs"},{"values":[0,0,0,0,0,0,0,0,0,0,0],"quality":"Equal","isMain":false,"metricName":"# similar misassembled blocks"}]],["Reference statistics",[{"values":[21129,21129,21129,21129,21129,21129,21129,21129,21129,21129,21129],"quality":"Equal","isMain":false,"metricName":"Reference length"},{"values":[1,1,1,1,1,1,1,1,1,1,1],"quality":"Equal","isMain":false,"metricName":"Reference fragments"},{"values":["34.65","34.65","34.65","34.65","34.65","34.65","34.65","34.65","34.65","34.65","34.65"],"quality":"Equal","isMain":false,"metricName":"Reference GC (%)"}]]],"referenceName":"gi\_12248100\_ref\_NC","date":"18 July 2018, Wednesday, 16:13:40","order":[0,1,2,3,4,5,6,7,8,9,10],"assembliesNames":["CLC","IDBA\_UD","MEGAHIT","MIRA","Ray\_Meta","SPAdes","SPAdes\_meta","SPAdes\_sc","SPAdes\_sc\_careful","Velvet","Geneious"]},{"assembliesWithNs":null,"minContig":500,"report":[["Genome statistics",[{"values":["3.962","5.390","5.732","4.560","22.062","20.351","8.168","1.692","1.692","3.220","5.173","35.842"],"quality":"More is better","isMain":true,"metricName":"Genome fraction (%)"},{"values":["1.000","1.217","1.000","1.001","1.000","1.000","1.000","1.000","1.000","1.000","1.000","1.360"],"quality":"Less is better","isMain":true,"metricName":"Duplication ratio"},{"values":[606,749,787,749,2243,4874,777,732,732,732,1197,3720],"quality":"More is better","isMain":true,"metricName":"Largest alignment"},{"values":[1714,2837,2481,1974,9546,8805,3534,732,732,1393,2238,21089],"quality":"More is better","isMain":true,"metricName":"Total aligned length"},{"values":[null,null,null,null,null,null,null,null,null,null,null,36887],"quality":"More is better","isMain":false,"metricName":"NG50"},{"values":[null,null,null,null,null,null,null,null,null,null,null,36887],"quality":"More is better","isMain":false,"metricName":"NG75"},{"values":[571,507,648,632,1113,4874,749,732,732,732,1197,null],"quality":"More is better","isMain":false,"metricName":"NA50"},{"values":[537,506,531,593,850,2936,692,732,732,661,536,null],"quality":"More is better","isMain":false,"metricName":"NA75"},{"values":[null,null,null,null,null,null,null,null,null,null,null,null],"quality":"More is better","isMain":true,"metricName":"NGA50"},{"values":[null,null,null,null,null,null,null,null,null,null,null,1],"quality":"Less is better","isMain":false,"metricName":"LG50"},{"values":[null,null,null,null,null,null,null,null,null,null,null,1],"quality":"Less is better","isMain":false,"metricName":"LG75"},{"values":[2,3,2,2,3,1,3,1,1,1,1,null],"quality":"Less is better","isMain":false,"metricName":"LA50"},{"values":[3,4,3,3,6,2,4,1,1,2,2,null],"quality":"Less is better","isMain":false,"metricName":"LA75"}]],["Misassemblies",[{"values":[0,0,0,0,0,0,0,0,0,0,0,0],"quality":"Less is better","isMain":true,"metricName":"# misassemblies"},{"values":[0,0,0,0,0,0,0,0,0,0,0,0],"quality":"Less is better","isMain":false,"metricName":" # relocations"},{"values":[0,0,0,0,0,0,0,0,0,0,0,0],"quality":"Less is better","isMain":false,"metricName":" # translocations"},{"values":[0,0,0,0,0,0,0,0,0,0,0,0],"quality":"Less is better","isMain":false,"metricName":" # inversions"},{"values":[0,0,0,0,0,0,0,0,0,0,0,0],"quality":"Less is better","isMain":false,"metricName":"# misassembled contigs"},{"values":[0,0,0,0,0,0,0,0,0,0,0,0],"quality":"Less is better","isMain":true,"metricName":"Misassembled contigs length"},{"values":[0,0,0,0,0,0,0,0,0,0,0,0],"quality":"Less is better","isMain":false,"metricName":"# local misassemblies"},{"values":[0,0,0,0,0,0,0,0,0,0,0,3],"quality":"Less is better","isMain":false,"metricName":"# unaligned mis. contigs"}]],["Unaligned",[{"values":[0,0,0,0,0,0,0,0,0,0,0,0],"quality":"Less is better","isMain":false,"metricName":"# fully unaligned contigs"},{"values":[0,0,0,0,0,0,0,0,0,0,0,0],"quality":"Less is better","isMain":false,"metricName":"Fully unaligned length"},{"values":[0,0,0,0,1,0,0,0,0,0,0,3],"quality":"Less is better","isMain":false,"metricName":"# partially unaligned contigs"},{"values":[0,0,0,0,850,0,0,0,0,0,0,40441],"quality":"Less is better","isMain":false,"metricName":"Partially unaligned length"}]],["Mismatches",[{"values":[0,7,6,9,121,121,0,0,0,0,0,259],"quality":"Less is better","isMain":false,"metricName":"# mismatches"},{"values":[0,1,1,1,1,0,0,0,0,0,0,3],"quality":"Less is better","isMain":false,"metricName":"# indels"},{"values":[0,1,1,1,1,0,0,0,0,0,0,3],"quality":"Less is better","isMain":false,"metricName":"Indels length"},{"values":["0.00","300.17","241.94","456.16","1267.68","1374.22","0.00","0.00","0.00","0.00","0.00","1670.21"],"quality":"Less is better","isMain":true,"metricName":"# mismatches per 100 kbp"},{"values":["0.00","42.88","40.32","50.68","10.48","0.00","0.00","0.00","0.00","0.00","0.00","19.35"],"quality":"Less is better","isMain":true,"metricName":"# indels per 100 kbp"},{"values":[0,1,1,1,1,0,0,0,0,0,0,3],"quality":"Less is better","isMain":false,"metricName":" # indels (<= 5 bp)"},{"values":[0,0,0,0,0,0,0,0,0,0,0,0],"quality":"Less is better","isMain":false,"metricName":" # indels (> 5 bp)"},{"values":[0,0,0,0,3,0,0,0,0,0,0,280],"quality":"Less is better","isMain":false,"metricName":"# N's"},{"values":["0.00","0.00","0.00","0.00","28.86","0.00","0.00","0.00","0.00","0.00","0.00","455.06"],"quality":"Less is better","isMain":true,"metricName":"# N's per 100 kbp"}]],["Statistics without reference",[{"values":[3,5,4,3,8,3,5,1,1,2,3,3],"quality":"Equal","isMain":true,"metricName":"# contigs"},{"values":[0,0,0,0,6,2,0,0,0,0,1,3],"quality":"Equal","isMain":false,"metricName":"# contigs (>= 1000 bp)"},{"values":[0,0,0,0,0,0,0,0,0,0,0,2],"quality":"Equal","isMain":false,"metricName":"# contigs (>= 5000 bp)"},{"values":[0,0,0,0,0,0,0,0,0,0,0,2],"quality":"Equal","isMain":false,"metricName":"# contigs (>= 10000 bp)"},{"values":[0,0,0,0,0,0,0,0,0,0,0,1],"quality":"Equal","isMain":false,"metricName":"# contigs (>= 25000 bp)"},{"values":[0,0,0,0,0,0,0,0,0,0,0,0],"quality":"Equal","isMain":false,"metricName":"# contigs (>= 50000 bp)"},{"values":[606,749,787,749,2243,4874,777,732,732,732,1197,36887],"quality":"More is better","isMain":true,"metricName":"Largest contig"},{"values":[1714,2837,2481,1974,10396,8805,3534,732,732,1393,2238,61530],"quality":"More is better","isMain":true,"metricName":"Total length"},{"values":[0,0,0,0,8766,7810,0,0,0,0,1197,61530],"quality":"More is better","isMain":true,"metricName":"Total length (>= 1000 bp)"},{"values":[0,0,0,0,0,0,0,0,0,0,0,58257],"quality":"More is better","isMain":false,"metricName":"Total length (>= 5000 bp)"},{"values":[0,0,0,0,0,0,0,0,0,0,0,58257],"quality":"More is better","isMain":true,"metricName":"Total length (>= 10000 bp)"},{"values":[0,0,0,0,0,0,0,0,0,0,0,36887],"quality":"More is better","isMain":false,"metricName":"Total length (>= 25000 bp)"},{"values":[0,0,0,0,0,0,0,0,0,0,0,0],"quality":"More is better","isMain":true,"metricName":"Total length (>= 50000 bp)"},{"values":[571,507,648,632,1386,4874,749,732,732,732,1197,36887],"quality":"More is better","isMain":false,"metricName":"N50"},{"values":[537,506,531,593,1037,2936,692,732,732,661,536,21370],"quality":"More is better","isMain":false,"metricName":"N75"},{"values":[2,3,2,2,3,1,3,1,1,1,1,1],"quality":"Less is better","isMain":false,"metricName":"L50"},{"values":[3,4,3,3,6,2,4,1,1,2,2,2],"quality":"Less is better","isMain":false,"metricName":"L75"},{"values":["36.87","29.82","31.92","31.16","35.36","33.02","33.90","28.42","28.42","30.44","30.03","34.26"],"quality":"Equal","isMain":false,"metricName":"GC (%)"}]],["Predicted genes",[]],["Similarity statistics",[{"values":[0,0,0,0,0,0,0,0,0,0,0,0],"quality":"Equal","isMain":false,"metricName":"# similar correct contigs"},{"values":[0,0,0,0,0,0,0,0,0,0,0,0],"quality":"Equal","isMain":false,"metricName":"# similar misassembled blocks"}]],["Reference statistics",[{"values":[43265,43265,43265,43265,43265,43265,43265,43265,43265,43265,43265,43265],"quality":"Equal","isMain":false,"metricName":"Reference length"},{"values":[1,1,1,1,1,1,1,1,1,1,1,1],"quality":"Equal","isMain":false,"metricName":"Reference fragments"},{"values":["34.28","34.28","34.28","34.28","34.28","34.28","34.28","34.28","34.28","34.28","34.28","34.28"],"quality":"Equal","isMain":false,"metricName":"Reference GC (%)"}]]],"referenceName":"gi\_122891714\_ref\_NC","date":"18 July 2018, Wednesday, 16:13:50","order":[0,1,2,3,4,5,6,7,8,9,10,11],"assembliesNames":["ABySS\_127","CLC","IDBA\_UD","MEGAHIT","MIRA","Ray\_Meta","SPAdes","SPAdes\_meta","SPAdes\_sc","SPAdes\_sc\_careful","Velvet","Geneious"]},{"assembliesWithNs":null,"minContig":500,"report":[["Genome statistics",[{"values":["67.943","47.486","53.184","51.093","60.263","83.473","62.737","10.372","69.703","45.040","59.688","61.028","25.579","58.560"],"quality":"More is better","isMain":true,"metricName":"Genome fraction (%)"},{"values":["1.005","1.003","1.013","1.014","1.016","1.250","1.040","1.005","1.013","1.000","1.004","1.004","1.000","1.121"],"quality":"Less is better","isMain":true,"metricName":"Duplication ratio"},{"values":[11172,6492,6958,6740,6820,11765,4672,1275,10511,6569,9250,9250,2877,15800],"quality":"More is better","isMain":true,"metricName":"Largest alignment"},{"values":[29556,20615,23305,22414,26506,45146,28236,4510,30550,19494,25945,26524,11071,28403],"quality":"More is better","isMain":true,"metricName":"Total aligned length"},{"values":[3466,null,636,682,2058,47368,1582,null,2219,null,1921,1904,null,60961],"quality":"More is better","isMain":false,"metricName":"NG50"},{"values":[null,null,null,null,null,47368,null,null,null,null,null,null,null,60961],"quality":"More is better","isMain":false,"metricName":"NG75"},{"values":[4839,4066,2794,2481,2565,null,2653,674,3777,4885,4258,4203,2014,null],"quality":"More is better","isMain":false,"metricName":"NA50"},{"values":[3466,2234,1245,1218,1250,null,1582,609,2219,1921,1921,1904,1836,null],"quality":"More is better","isMain":false,"metricName":"NA75"},{"values":[3466,null,545,682,1250,4107,1582,null,2219,null,1921,1904,null,2885],"quality":"More is better","isMain":true,"metricName":"NGA50"},{"values":[null,null,null,null,null,2910,null,null,null,null,null,null,null,null],"quality":"More is better","isMain":false,"metricName":"NGA75"},{"values":[4,null,11,11,6,1,7,null,5,null,5,5,null,1],"quality":"Less is better","isMain":false,"metricName":"LG50"},{"values":[null,null,null,null,null,1,null,null,null,null,null,null,null,1],"quality":"Less is better","isMain":false,"metricName":"LG75"},{"values":[2,2,3,3,4,null,4,3,3,2,2,3,3,null],"quality":"Less is better","isMain":false,"metricName":"LA50"},{"values":[4,4,7,6,8,null,7,5,5,4,5,5,4,null],"quality":"Less is better","isMain":false,"metricName":"LA75"},{"values":[4,null,11,11,8,4,7,null,5,null,5,5,null,3],"quality":"Less is better","isMain":true,"metricName":"LGA50"},{"values":[null,null,null,null,null,7,null,null,null,null,null,null,null,null],"quality":"Less is better","isMain":false,"metricName":"LGA75"}]],["Misassemblies",[{"values":[0,0,0,0,0,0,0,0,0,0,0,0,0,0],"quality":"Less is better","isMain":true,"metricName":"# misassemblies"},{"values":[0,0,0,0,0,0,0,0,0,0,0,0,0,0],"quality":"Less is better","isMain":false,"metricName":" # relocations"},{"values":[0,0,0,0,0,0,0,0,0,0,0,0,0,0],"quality":"Less is better","isMain":false,"metricName":" # translocations"},{"values":[0,0,0,0,0,0,0,0,0,0,0,0,0,0],"quality":"Less is better","isMain":false,"metricName":" # inversions"},{"values":[0,0,0,0,0,0,0,0,0,0,0,0,0,0],"quality":"Less is better","isMain":false,"metricName":"# misassembled contigs"},{"values":[0,0,0,0,0,0,0,0,0,0,0,0,0,0],"quality":"Less is better","isMain":true,"metricName":"Misassembled contigs length"},{"values":[0,0,0,0,0,0,0,0,0,0,1,1,0,1],"quality":"Less is better","isMain":false,"metricName":"# local misassemblies"},{"values":[0,0,0,0,0,2,0,0,0,0,0,0,0,3],"quality":"Less is better","isMain":false,"metricName":"# unaligned mis. contigs"}]],["Unaligned",[{"values":[0,0,0,0,0,0,0,0,0,0,0,0,0,0],"quality":"Less is better","isMain":false,"metricName":"# fully unaligned contigs"},{"values":[0,0,0,0,0,0,0,0,0,0,0,0,0,0],"quality":"Less is better","isMain":false,"metricName":"Fully unaligned length"},{"values":[0,0,1,0,1,7,0,0,0,1,1,1,0,6],"quality":"Less is better","isMain":false,"metricName":"# partially unaligned contigs"},{"values":[0,0,606,0,3068,60522,0,0,0,1072,969,969,0,136217],"quality":"Less is better","isMain":false,"metricName":"Partially unaligned length"}]],["Mismatches",[{"values":[0,1,15,2,30,129,6,0,11,129,28,8,1,95],"quality":"Less is better","isMain":false,"metricName":"# mismatches"},{"values":[0,0,0,0,0,1,0,0,0,2,0,1,0,5],"quality":"Less is better","isMain":false,"metricName":"# indels"},{"values":[0,0,0,0,0,1,0,0,0,2,0,1,0,6],"quality":"Less is better","isMain":false,"metricName":"Indels length"},{"values":["0.00","4.87","65.16","9.04","115.02","357.05","22.10","0.00","36.46","661.74","108.38","30.29","9.03","374.81"],"quality":"Less is better","isMain":true,"metricName":"# mismatches per 100 kbp"},{"values":["0.00","0.00","0.00","0.00","0.00","2.77","0.00","0.00","0.00","10.26","0.00","3.79","0.00","19.73"],"quality":"Less is better","isMain":true,"metricName":"# indels per 100 kbp"},{"values":[0,0,0,0,0,1,0,0,0,2,0,1,0,5],"quality":"Less is better","isMain":false,"metricName":" # indels (<= 5 bp)"},{"values":[0,0,0,0,0,0,0,0,0,0,0,0,0,0],"quality":"Less is better","isMain":false,"metricName":" # indels (> 5 bp)"},{"values":[0,3,0,0,0,12,0,0,0,0,0,0,0,931],"quality":"Less is better","isMain":false,"metricName":"# N's"},{"values":["0.00","14.55","0.00","0.00","0.00","11.36","0.00","0.00","0.00","0.00","0.00","0.00","0.00","565.54"],"quality":"Less is better","isMain":true,"metricName":"# N's per 100 kbp"}]],["Statistics without reference",[{"values":[11,9,15,12,15,18,12,6,13,6,9,11,6,6],"quality":"Equal","isMain":true,"metricName":"# contigs"},{"values":[5,5,7,7,8,13,10,1,7,6,6,5,5,6],"quality":"Equal","isMain":false,"metricName":"# contigs (>= 1000 bp)"},{"values":[1,1,1,1,1,5,0,0,1,1,1,1,0,5],"quality":"Equal","isMain":false,"metricName":"# contigs (>= 5000 bp)"},{"values":[1,0,0,0,0,2,0,0,1,0,1,1,0,4],"quality":"Equal","isMain":false,"metricName":"# contigs (>= 10000 bp)"},{"values":[0,0,0,0,0,1,0,0,0,0,0,0,0,3],"quality":"Equal","isMain":false,"metricName":"# contigs (>= 25000 bp)"},{"values":[0,0,0,0,0,0,0,0,0,0,0,0,0,1],"quality":"Equal","isMain":false,"metricName":"# contigs (>= 50000 bp)"},{"values":[11172,6492,6958,6740,6820,47368,4672,1275,10511,6569,10219,10219,2877,60961],"quality":"More is better","isMain":true,"metricName":"Largest contig"},{"values":[29556,20615,23933,22414,29576,105668,28236,4510,30550,20569,26914,27493,11071,164620],"quality":"More is better","isMain":true,"metricName":"Total length"},{"values":[25751,17876,19145,18782,25310,102171,26469,1275,26817,20569,24854,23078,10431,164620],"quality":"More is better","isMain":true,"metricName":"Total length (>= 1000 bp)"},{"values":[11172,6492,6958,6740,6820,79319,0,0,10511,6569,10219,10219,0,161816],"quality":"More is better","isMain":false,"metricName":"Total length (>= 5000 bp)"},{"values":[11172,0,0,0,0,60062,0,0,10511,0,10219,10219,0,156102],"quality":"More is better","isMain":true,"metricName":"Total length (>= 10000 bp)"},{"values":[0,0,0,0,0,47368,0,0,0,0,0,0,0,131708],"quality":"More is better","isMain":false,"metricName":"Total length (>= 25000 bp)"},{"values":[0,0,0,0,0,0,0,0,0,0,0,0,0,60961],"quality":"More is better","isMain":true,"metricName":"Total length (>= 50000 bp)"},{"values":[4839,4066,2794,2481,3037,12694,2653,674,3777,4885,4258,4403,2014,42152],"quality":"More is better","isMain":false,"metricName":"N50"},{"values":[3466,2234,1245,1218,2058,5222,1582,609,2219,1926,2349,2349,1836,28595],"quality":"More is better","isMain":false,"metricName":"N75"},{"values":[2,2,3,3,4,2,4,3,3,2,2,2,3,2],"quality":"Less is better","isMain":false,"metricName":"L50"},{"values":[4,4,7,6,6,5,7,5,5,4,4,4,4,3],"quality":"Less is better","isMain":false,"metricName":"L75"},{"values":["34.59","34.23","35.06","34.86","34.88","35.40","35.32","34.61","34.79","34.98","34.95","35.42","33.68","34.97"],"quality":"Equal","isMain":false,"metricName":"GC (%)"}]],["Predicted genes",[]],["Similarity statistics",[{"values":[0,0,0,0,0,0,0,0,0,0,0,0,0,0],"quality":"Equal","isMain":false,"metricName":"# similar correct contigs"},{"values":[0,0,0,0,0,0,0,0,0,0,0,0,0,0],"quality":"Equal","isMain":false,"metricName":"# similar misassembled blocks"}]],["Reference statistics",[{"values":[43282,43282,43282,43282,43282,43282,43282,43282,43282,43282,43282,43282,43282,43282],"quality":"Equal","isMain":false,"metricName":"Reference length"},{"values":[1,1,1,1,1,1,1,1,1,1,1,1,1,1],"quality":"Equal","isMain":false,"metricName":"Reference fragments"},{"values":["34.90","34.90","34.90","34.90","34.90","34.90","34.90","34.90","34.90","34.90","34.90","34.90","34.90","34.90"],"quality":"Equal","isMain":false,"metricName":"Reference GC (%)"}]]],"referenceName":"gi\_122891784\_ref\_NC","date":"18 July 2018, Wednesday, 16:14:01","order":[0,1,2,3,4,5,6,7,8,9,10,11,12,13],"assembliesNames":["ABySS\_127","ABySS\_63","CLC","IDBA\_UD","MEGAHIT","MIRA","Ray\_Meta","SOAPdenovo2","SPAdes","SPAdes\_meta","SPAdes\_sc","SPAdes\_sc\_careful","Velvet","Geneious"]},{"assembliesWithNs":null,"minContig":500,"report":[["Genome statistics",[{"values":["87.022","72.734","87.851","84.724","85.467","100.000","8.490","22.943","90.120","86.676","75.653","80.698","29.731","100.000"],"quality":"More is better","isMain":true,"metricName":"Genome fraction (%)"},{"values":["1.010","1.015","1.001","1.028","1.023","0.993","1.039","1.454","1.007","1.000","1.008","1.010","1.003","1.006"],"quality":"Less is better","isMain":true,"metricName":"Duplication ratio"},{"values":[26287,7528,21461,15582,15398,70134,995,1222,29553,45080,7661,11729,3275,75680],"quality":"More is better","isMain":true,"metricName":"Largest alignment"},{"values":[121139,101753,121151,119992,120498,149016,12116,31681,125056,119426,105150,112357,41113,150898],"quality":"More is better","isMain":true,"metricName":"Total aligned length"},{"values":[8265,2117,10979,5746,6692,124838,null,749,9987,24870,2444,2392,null,125392],"quality":"More is better","isMain":false,"metricName":"NG50"},{"values":[3480,null,5765,2207,3537,124838,null,null,7865,9194,730,861,null,125392],"quality":"More is better","isMain":false,"metricName":"NG75"},{"values":[9354,3147,11660,7034,7118,35474,745,null,10520,24870,3863,3060,921,21924],"quality":"More is better","isMain":false,"metricName":"NA50"},{"values":[5984,2104,7760,3256,5501,null,594,null,9356,20431,2103,1907,676,null],"quality":"More is better","isMain":false,"metricName":"NA75"},{"values":[8265,2117,10979,5746,6692,70134,null,null,9987,24870,2444,2392,null,75680],"quality":"More is better","isMain":true,"metricName":"NGA50"},{"values":[3480,null,5765,2207,3537,35474,null,null,7865,9194,730,861,null,35475],"quality":"More is better","isMain":false,"metricName":"NGA75"},{"values":[6,18,5,8,8,1,null,49,5,2,16,15,nu
[truncated: 5,764,648 more chars]
