# Supplementary material for: Choice of assembly software has a critical impact on virome characterisation
Source: Microbiome. 2019 Jan 28;7:12. doi: 10.1186/s40168-019-0626-5 (PMC6350398; doi:10.1186/s40168-019-0626-5)
Supplement: Supplementary file 6 — Figure S1. Analysis of recovered genome fraction and indel/mismatch counts for mock communities A and B. Triangles represent N/A values for mismatches and indels caused by assembly failures. (PDF 293 kb) [file 40168_2019_626_MOESM6_ESM.pdf]

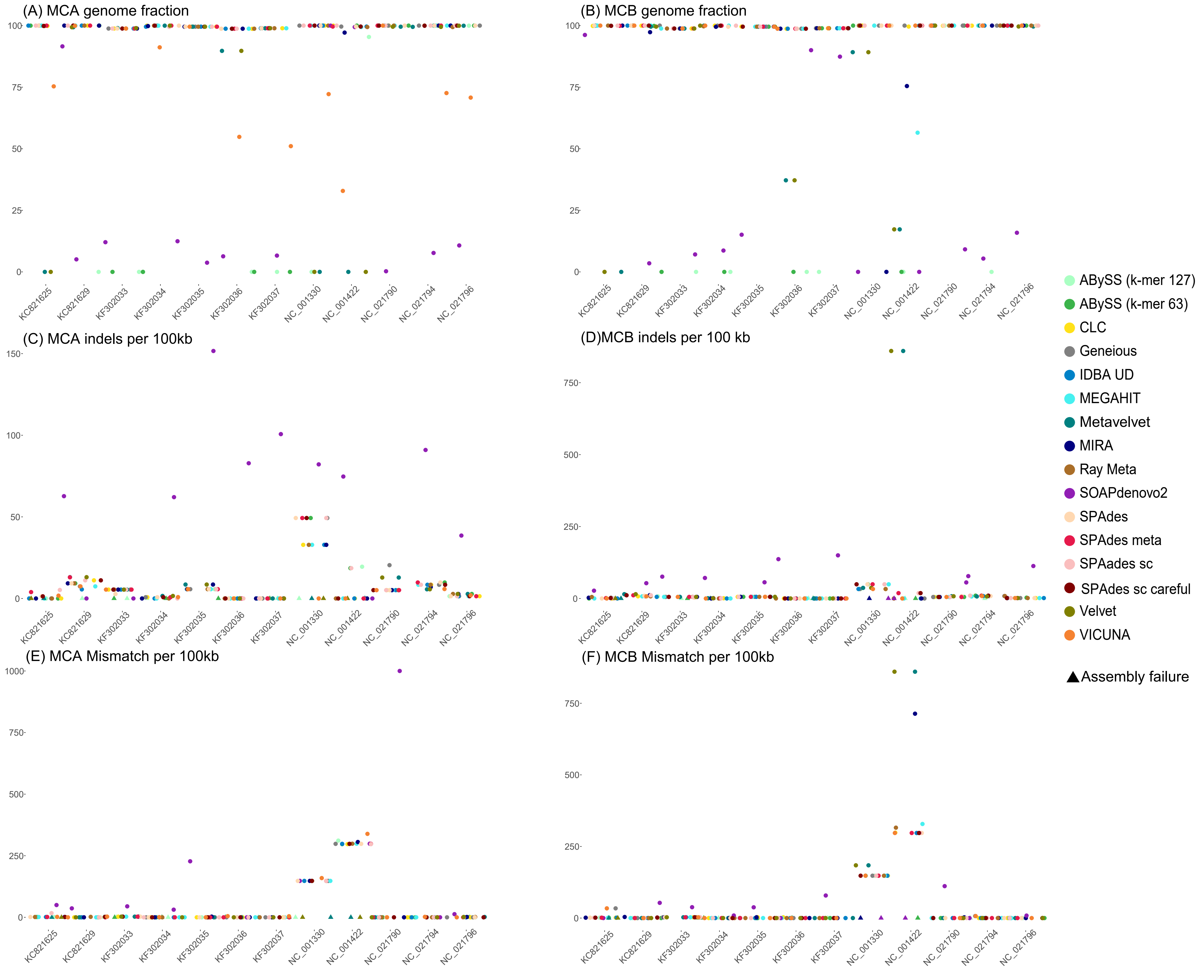
(A) MCA genome fraction
(B) MCB genome fraction
(C) MCA indels per 100kb
(D)MCB indels per 100 kb
(E) MCA Mismatch per 100kb
(F) MCB Mismatch per 100kb
KC821625
KC821629
KF302033
KF302034
KF302035
KF302036
KF302037
NC_001330
NC_001422
NC_021790
NC_021794
NC_021796
ABySS (k-mer 127)
ABySS (k-mer 63)
CLC
Geneious
IDBA UD
MEGAHIT
Metavelvet
MIRA
Ray Meta
SOAPdenovo2
SPAdes
SPAdes meta
SPAades sc
SPAdes sc careful
Velvet
VICUNA
Assembly failure
